# Cancer incidence and competing mortality risk following 15 presenting symptoms in primary care: a population-based cohort study using electronic healthcare records

**DOI:** 10.1136/bmjonc-2024-000500

**Published:** 2024-11-21

**Authors:** Matthew E Barclay, Cristina Renzi, Hannah Harrison, Ana Torralbo, Becky White, Samantha Hiu Yan Ip, Juliet Usher-Smith, Jane Lange, Nora Pashayan, Spiros Denaxas, Angela M Wood, Antonis Antoniou, Georgios Lyratzopoulos

**Affiliations:** 1 Department of Behavioural Science and Health, Institute of Epidemiology and Healthcare, University College London, London, UK; 2 Faculty of Medicine, University Vita-Salute San Raffaele, Milan, Italy; 3 Department of Public Health and Primary Care, School of Clinical Medicine, University of Cambridge, Cambridge, UK; 4 Institute of Health Informatics, University College London, London, UK; 5 Victor Phillip Dahdaleh Heart and Lung Research Institute, University of Cambridge, Cambridge, UK; 6 Cancer Early Detection Advanced Research Center, Oregon Health & Science University, Portland, Oregon, USA; 7 Department of Applied Health Research, Institute of Epidemiology and Healthcare, University College London, London, UK; 8 British Heart Foundation Centre of Research Excellence, University of Cambridge, Cambridge, UK; 9 National Institute for Health and Care Research Blood and Transplant Research Unit in Donor Health and Behaviour, University of Cambridge, Cambridge, UK; 10 Health Data Research UK Cambridge, Wellcome Genome Campus and University of Cambridge, Cambridge, UK; 11 Cambridge Centre for Artificial Intelligence in Medicine, University of Cambridge, Cambridge, UK

**Keywords:** Epidemiology

## Abstract

**Objectives:**

Assessment of age, sex and smoking-specific risk of cancer diagnosis and non-cancer mortality following primary care consultation for 15 new-onset symptoms.

**Methods and analysis:**

Data on patients aged 30–99 in 2007–2017 were extracted from a UK primary care database (CPRD Gold), comprising a randomly selected reference group and a symptomatic cohort of patients presenting with one of 15 new onset symptoms (abdominal pain, abdominal bloating, rectal bleed, change in bowel habit, dyspepsia, dysphagia, dyspnoea, haemoptysis, haematuria, fatigue, night sweats, weight loss, jaundice, breast lump and post-menopausal bleed).

Time-to-event models were used to estimate outcome-specific hazards for site-specific cancer diagnosis and non-cancer mortality and to estimate cumulative incidence up to 12 months following index consultation.

**Results:**

Data included 1 622 419 patients, of whom 36 802 had a cancer diagnosis and 28 857 died without a cancer diagnosis within 12 months of the index.

The risk of specific cancers exceeded the UK urgent referral risk threshold of 3% from a relatively young age for patients with red flag symptoms. For non-organ-specific symptoms, the risk of cancer at individual sites either did not reach the threshold at any age or reached it only in older patients.

**Conclusion:**

Patients with new-onset symptoms in primary care often have comparable risks of cancer diagnosis and non-cancer mortality. Non-organ-specific symptoms, in particular, are associated with elevated risk of cancer at multiple different sites. Management of symptomatic patients in primary care should be informed by the risk of different cancer types alongside mortality risk.

**WHAT IS ALREADY KNOWN ON THIS TOPIC**

- Evidence describing the diagnostic value of symptoms for cancer can help assess which patients who present to primary care need urgent specialist assessment.
- Current evidence is limited as age is often handled categorically, smoking status is not taken into account and study periods are historical.
- Further, evidence is concentrated on assessing the risk of specific cancer sites, although the same symptom can be related to cancer of different organs.

**WHAT THIS STUDY ADDS**

- We present evidence on age-, sex- and smoking-status-specific estimates of the risk of cancer of different organs and overall, alongside estimates of non-cancer death.
- Estimates relate to patients who present with one of 15 possible cancer symptoms from a relatively recent period.
- Certain symptoms such as jaundice and dysphagia are associated with a high risk of non-cancer death in older patients.
- Other symptoms, such as unintended weight loss, fatigue and abdominal pain, are associated with excess risk of a range of different cancers.

**HOW THIS STUDY MIGHT AFFECT RESEARCH, PRACTICE OR POLICY**

- We provide detailed evidence and results that may help frame future research studies into the risk of cancer in symptomatic patients and update and refine policy on referral and diagnostic investigation of patients in primary care.

## Introduction

 Most patients with cancer are diagnosed after symptomatic presentation,^1^ and, given the paucity of effective tests to enable population-based cancer screening, this is likely to be the case for the coming decade. Appropriately suspecting the diagnosis of cancer in symptomatic patients is difficult, as symptoms may be caused by many other diseases. Even so-termed ‘alarm’ or ‘red-flag’ symptoms typically have positive predictive values for cancer that do not exceed 5% in women of any age or in men younger than 70.^2^ In the UK, many patients with cancer experience diagnostic delays in the form of multiple pre-referral consultations and prolonged intervals to diagnosis, despite practice guidelines issued by the National Institute for Health and Social Care Excellence (NICE) that aimed to enable prompt diagnosis of cancer in primary care.^3 4^ Such delays are associated with adverse patient experience and worse clinical outcomes.^5^,^8^

Currently, the majority of research publications supporting practice guidelines come from case-control studies, examining symptom-related risk of specific cancer sites. This study design ignores that presenting symptoms are often shared between different cancers and diseases other than cancer; there has been no comprehensive examination of the risk of the full spectrum of possible cancer types for the most relevant presenting symptoms. Further, guideline recommendations handle major cancer risk factors sub-optimally, as smoking status is typically ignored as a risk stratifier, and age is typically not considered as a continuous variable, leading to information loss. Competing risk of death is also ignored, meaning that management decisions centred on cancer risk ignore risks related to other diseases.

This study is motivated by the need for evidence to support the updating of clinical practice guidelines for the primary care management of patients who present with symptoms of possible underlying cancer. Such evidence is needed both in terms of quantifying the absolute risk of different cancer types and also the probability of patients dying without a cancer diagnosis. We also aim to aid the development of and complement the use of risk prediction tools by describing in detail the associations between symptoms and cancer risk. We therefore assess age-, sex- and smoking-specific risk of cancer diagnosis and non-cancer mortality following primary care consultation for one of 15 new-onset symptoms.

## Methods

### Study population

We used a cohort study design, based on medical records from English National Health Service general practices that contributed anonymised primary-care electronic health records to the Clinical Practice Research Datalink Gold (CPRD). CPRD covers approximately 6.9% of the UK population,^9^ and patients in CPRD are broadly representative of the UK general population with respect to age, sex and ethnicity.^9^ CPRD was linked to cancer diagnosis information from the English national cancer registry.^10^ We considered all cancers excluding non-melanoma skin cancer, as non-melanoma skin cancer is imperfectly registered and primarily managed in primary care.

**Figure 1:**
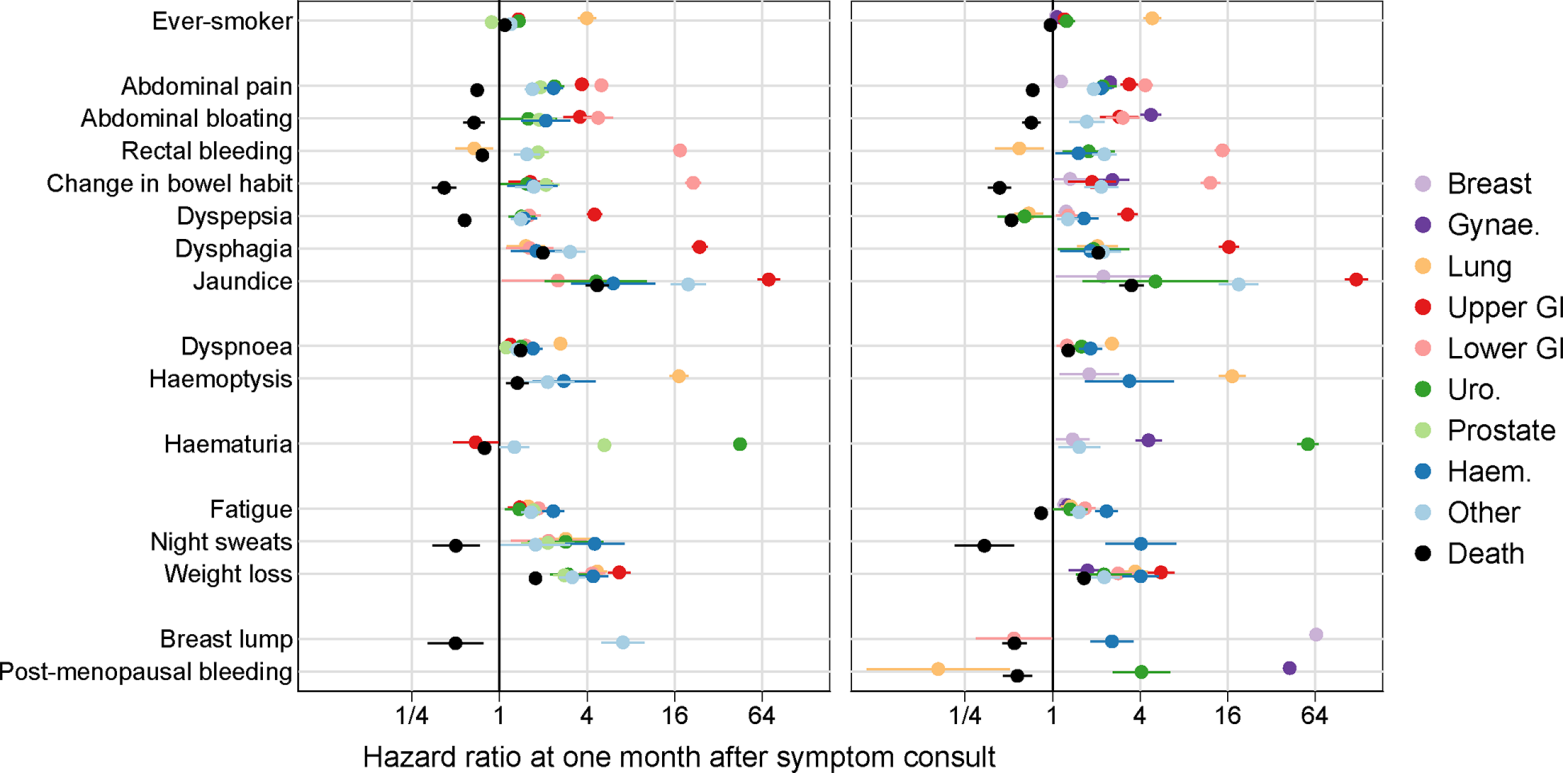
HRs for each cancer site and for non-cancer death at 1 month after index, for men (left) and women (right). Ever-smokers are compared with never-smokers; each symptom is compared with the control group. Models are stratified by sex and adjusted for age, smoking status and the presence of symptoms at the index date.

A study flowchart is given in online supplemental appendix 1 figure 1. We first extracted a random sample of patients from CPRD for use as a reference group, choosing index dates randomly from ‘valid’ follow-ups from 1 January 2007 to 31 December 2017. Patients in this reference group were not necessarily symptom-free (online supplemental appendix 1 table 1). Coded symptom data are known to be incomplete,^11 12^ so it was not possible to create a truly symptom-free control group with the data available for this study. Thus, we chose to use a reference group that would represent the average risk for patients registered in primary care. We then created a symptomatic cohort of all patients in CPRD Gold who had consulted for any of 15 presenting symptoms and who were not in the reference group, choosing the index date as the date of their first ‘valid’ consultation for a symptom during 1 January 2007 to 31 December 2017.

For an individual patient, follow-up was judged to be ‘valid’ if they had been registered at their practice for at least 1 year; their practice was judged by CPRD to be providing data of a suitable standard for use in research (ie, after the practice’s ‘up-to-standard’ date); it was before the last data transfer to CPRD (ie, the ‘last collection’ date); the patient was registered at a CPRD practice (ie, before the patient’s ‘transfer out’ date and before their death); the patient was aged 30–99; and the patient had not yet had a recorded cancer diagnosis in the cancer registry (excluding non-melanoma skin cancer).

### Outcomes

Both mortality and cancer diagnoses were considered. Mortality was identified from the primary care record; such information is highly concordant with the ‘gold standard’ official death registration records and is correct within 1 month 98% of the time.^13^ Cancers were split into seven groups for men and eight groups for women, summarised below and with a full ICD10 codelist in online supplemental appendix 1 table 2, guided by underlying body systems and corresponding major clinical specialties receiving urgent referrals for suspected cancer in England.^14^ Cancer diagnoses were sourced from linkages with the national cancer registry, and only the first cancer diagnosis was considered (excluding non-melanoma skin cancer); available cancer data covered diagnoses up until 31 December 2018.

The cancer groups considered were:

- Breast cancer (women only), including invasive breast and in-situ breast cancers.
- Gynaecological cancer (women only), including invasive cervical, in-situ cervical, ovarian, uterine and vulvar cancers.
- Lung, including lung cancer and mesothelioma.
- Upper gastrointestinal (GI), including liver, oesophageal, pancreatic and stomach cancers.
- Lower GI, including colon and rectal cancers.
- Urological, including bladder, in-situ bladder, kidney and other urinary tract cancers.
- Prostate cancer (men only).
- Haematological, including Hodgkin’s lymphoma, non-Hodgkin’s lymphoma, acute myeloid leukaemia, chronic lymphocytic leukaemia, other leukaemias, myeloma and other haematological cancers
- Other, including all other sites, specifically melanoma, unknown primary, thyroid and meningeal cancers, also including testicular cancer and male breast cancer.

The first outcome (of cancer diagnosis or non-cancer death) experienced by each patient was considered in the analysis. This means, for example, that in the analyses of cumulative incidence, a patient who died shortly following a cancer diagnosis would only be considered to have had a cancer diagnosis, and their death would not contribute to the estimation of mortality risk, irrespective of the cause of death. Patients with a cancer diagnosis on the same day as their death (including death certificate-only registrations of cancer) were treated as having had a cancer diagnosis rather than having died, noting that death certificate-only registrations remained at <0.4% through the study period.^15^

### Symptoms

Patients were selected due to a primary care presentation with one (or more) of 15 cancer-relevant symptoms or due to being in the reference group. The index date of symptomatic patients was the date of their first recorded symptom during ‘valid’ follow-up (defined in Methods—study population).

The symptoms we considered were a subset of those known to be associated with the risk of specific types of cancer and are already included in referral guidelines for symptomatic cancer.^3 16^ The included symptoms form part of the presentation in 40% of all patients with cancer in England.^1^ We identified symptoms from coded primary care data using existing Read v2 phenotyping algorithms.^16^ The symptoms we considered were:

- Abdominal symptoms:
  - Abdominal pain.
  - Abdominal bloating (including abdominal distension).
  - Rectal bleeding
  - Change in bowel habits.
  - Dyspepsia
  - Dysphagia
  - Jaundice
- Respiratory symptoms
  - Dyspnoea
  - Haemoptysis
- Urological symptoms
  - Haematuria.
- Non-specific symptoms:
  - Fatigue
  - Night sweats.
  - Weight loss.
- Breast and reproductive organ symptoms:
  - Breast lump (including in men).
  - Post-menopausal bleeding.

Only the first presenting symptom for each patient was included, and each patient was included at most once in the analysis. For example, if a patient had a consultation for a breast lump in 2007 that did not result in a cancer diagnosis and a consultation for abdominal pain in 2010 that did result in a cancer diagnosis, only the risk after the 2007 consultation for a breast lump would be included in analysis. Symptoms were included in the model using one-hot encoding, with patients in the reference group having all symptom variables set to 0. Where patients in a symptomatic cohort presented with two or more symptoms on their index date, all were included as index symptoms (such occurrences were rare, see end of the Results section). Symptoms that were not consulted for on the same day as the index were not considered.

### Smoking status, sex and age

Patients were categorised as ever-smokers or never-smokers. Ever-smokers included all patients with a record of being current or ex-smokers in their entire primary care record, including periods after a cancer diagnosis or before their record became eligible for use in this study; never-smokers included all other patients. Patients were grouped as male or female based on the recorded gender in their primary care record. Patients’ age was estimated as the number of years between the mid-point of their year of birth and their index date.

### Statistical methods

The initial analysis described the distribution of patients in the sample and counts of cancer diagnoses and deaths within 12 months of any index symptom.

Hazards for specific cancers and non-cancer mortality were estimated using flexible parametric (Royston–Parmar) time-to-event models,^17^ using three degrees of freedom to model the baseline hazard. Follow-up for these analyses was censored at 18 months after the index symptom, at the first event (ie, cancer diagnosis or death) or the end of the available cancer registry follow-up on 31 December 2018 if earlier. Models were stratified by sex and included the following covariates:

- Age (restricted cubic spline with six knots).
- Smoking status (binary, ever record of smoking in primary care data vs never).
- Index symptom (15 binary variables indicating the symptom(s) each patient had on their index date (all zero for patients in the reference group)).
- An interaction with (the log of) follow-up time in months for each index symptom, allowing the association between symptom and cause-specific risk to decay over time. This was motivated by the fact that following many possible symptoms of cancer, excess risk is highest in the first months following presentation (eg,^18^).

The cumulative incidence of cancer group and non-cancer mortality was estimated by combining each of the cause-specific models into a multistate model using the latent failure time approach.^18^ We report cumulative incidence for combinations of age-, sex- and smoking-specific symptoms up to 12 months follow-up, with results focusing on estimated cumulative incidence at 12 months and age considered in 5 year intervals. To sense-check these model-based estimates, we additionally examined the crude cumulative incidence for each cancer group and non-cancer mortality within 12 months of each symptom by sex and smoking status using Aalen–Johansen non-parametric cumulative incidence curves.^19 20^

Concordant with the methods and evidence that informed the development of NICE guidelines, we have considered the modelled cumulative incidence at 12 months to represent the positive predictive value for the outcome for the symptom.^3^ Further, we calculated the (sex-/smoking-/symptom-specific) age at which the cancer risk exceeded the 3% risk threshold for referrals used in the UK. We additionally present similar estimates for each individual cancer group.

Statistical modelling used Stata 17 MP. Simulation of failure times was performed on a high-performance cluster using Stata 16 MP. Survival models were fit using the *merlin* package,^21^ and multistate modelling was facilitated by the *multistate* package.^22^ In principle, the cancer risk for any combination of symptoms can be estimated from the cause-specific models, but these have not been produced due to computational limitations and the very large number of potential combinations. Data extraction and analysis code are available at https://github.com/MattEBarclay/cprd_symptom_cancer_1.

### Patient and public involvement

The study forms part of a programme of work examining the predictive value of symptoms for cancer diagnosis using electronic health records data. To support this programme, we ran three focus groups in August and September 2023 including a total of 15 patient and public involvement volunteers. Study reporting was informed by input from these volunteers, but no specific changes were made.

## Results

The analysis cohort included 1 622 419 patients, 835 995 with an eligible first symptom recorded between 2007 and 2017 (table 1; see online supplemental appendix 2 tables 1–16 for the demographics of the reference group and each symptomatic sub-cohort). More than half of the cohort (64%, 1 040 862) were aged under 60 at the index (69% of the reference group vs 60% of those with symptoms, online supplemental appendix 2 table 1, with 24 731 (1.5%) patients aged 90 or older. The distribution of symptoms was uneven, with 14.6% of the cohort having abdominal pain as the index symptom, followed by fatigue (8.9%), dyspnoea (8.8%), dyspepsia (6.8%), rectal bleeding (3.0%), breast lump (2.4%), haematuria (1.6%), abdominal bloating (1.4%), weight loss (1.2%), change in bowel habit (1.1%), dysphagia (0.9%), post-menopausal bleeding (0.5%), night sweats (0.5%), haemoptysis (0.4%) and jaundice (0.1%). The majority of patients (64%) had at least one smoking-related read code in their records and were identified as ever-smokers; recorded smoking was slightly less common in the reference group (60%, online supplemental appendix 2 table 1). Within 12 months of their first recorded symptom, 36 802 patients had a cancer diagnosis and 28 867 patients died without a cancer diagnosis (a further 9288 died following a cancer diagnosis); both cancer and mortality risk were higher in older patients. Ever-smokers had a slightly higher cancer risk than patients without any smoking-related codes (table 1).

**Table 1:** Cohort summary

	Cohort		Cancers within 12 months		Deaths within 12 months, no preceding cancer diagnosis		Deaths within 12 months	
	N	(col %)	N	(row %)	N	(row %)	N	(row %)
**Total**	1 622 419		36 802	(2.3)	28 867	(1.8)	38 155	(2.4)
**Age at index (grouped**)								
30 to 39	395 413	(24.4)	1571	(0.4)	426	(0.1)	488	(0.1)
40 to 49	350 133	(21.6)	3063	(0.9)	792	(0.2)	1027	(0.3)
50 to 59	295 316	(18.2)	5080	(1.7)	1343	(0.5)	2105	(0.7)
60 to 69	259 039	(16.0)	9014	(3.5)	2829	(1.1)	4799	(1.9)
70 to 79	185 854	(11.5)	10 142	(5.5)	6007	(3.2)	8967	(4.8)
80 to 89	111 933	(6.9)	6818	(6.1)	11 453	(10.2)	14 173	(12.7)
90 to 99	24 731	(1.5)	1114	(4.5)	6017	(24.3)	6596	(26.7)
**Sex**								
Female	880 888	(54.3)	19 808	(2.2)	15 671	(1.8)	19 930	(2.3)
Male	741 531	(45.7)	16 994	(2.3)	13 196	(1.8)	18 225	(2.5)
**IMD group**								
Least deprived	377 575	(23.3)	8934	(2.4)	5661	(1.5)	7662	(2.0)
2	356 859	(22.0)	8347	(2.3)	6177	(1.7)	8208	(2.3)
3	342 184	(21.1)	7755	(2.3)	6355	(1.9)	8244	(2.4)
4	294 638	(18.2)	6483	(2.2)	5559	(1.9)	7364	(2.5)
Most deprived	251 163	(15.5)	5283	(2.1)	5115	(2.0)	6677	(2.7)
**Any record of smoking**								
Never-smoker	586 639	(36.2)	10 390	(1.8)	10 043	(1.7)	12 302	(2.1)
Ever-smoker	1 035 780	(63.8)	26 412	(2.5)	18 824	(1.8)	25 853	(2.5)
**Index symptom**								
Reference group	786 424	(48.5%)	7536	(1.0)	12 520	(1.6)	14 554	(1.9)
Abdominal pain	236 226	(14.6%)	5675	(2.4)	2203	(0.9)	3871	(1.6)
Abdominal bloating	23 228	(1.4%)	660	(2.8)	272	(1.2)	457	(2.0)
Rectal bleeding	49 273	(3.0%)	1896	(3.8)	875	(1.8)	1098	(2.2)
Change in bowel habit	17 629	(1.1%)	1091	(6.2)	165	(0.9)	366	(2.1)
Dyspepsia	110 312	(6.8%)	2178	(2.0)	976	(0.9)	1605	(1.5)
Dysphagia	15 291	(0.9%)	1053	(6.9)	1181	(7.7)	1638	(10.7)
Jaundice	1898	(0.1%)	468	(24.7)	221	(11.6)	510	(26.9)
Dyspnoea	142 431	(8.8%)	4015	(2.8)	6308	(4.4)	7820	(5.5)
Haemoptysis	5984	(0.4%)	417	(7.0)	147	(2.5)	330	(5.5)
Haematuria	26 051	(1.6%)	2791	(10.7)	601	(2.3)	987	(3.8)
Fatigue	143 719	(8.9%)	2476	(1.7)	2247	(1.6)	3015	(2.1)
Night sweats	7857	(0.5%)	137	(1.7)	31	(0.4)	67	(0.9)
Weight loss	20 163	(1.2%)	1291	(6.4)	1196	(5.9)	1846	(9.2)
Breast lump	38 623	(2.4%)	4814	(12.5)	90	(0.2)	277	(0.7)
Post-menopausal bleed	8252	(0.5%)	799	(9.7)	50	(0.6)	107	(1.3)

### Age-adjusted cancer-specific HRs for smoking and each index symptom

Both male and female ever-smokers had far higher cancer-specific hazards of lung cancer than non-smokers (figure 1 and online supplemental appendix 3, HR 4.8, 95% CI 4.2 to 5.6, for women and HR 4.0, 95% CI 3.5 to 4.6, for men) and elevated hazards of urological (eg, for men: HR 1.4, 95%CI 1.2 to 1.5, online supplemental appendix 3 table 4) and upper GI cancers (eg, for men: HR 1.4, 95% CI 1.2 to 1.5, online supplemental appendix 3 table 1).

Patients consulting for symptoms of possible cancer had similar or greater cause-specific hazards for almost every cancer site than the reference population (figure 1 and online supplemental appendix 3). Yet for 10 of the 15 studied symptoms, the symptom was associated with lower cause-specific hazards for death than the reference group (the exceptions being dysphagia, jaundice, dyspnoea, haemoptysis and weight loss).

**Figure 2:**
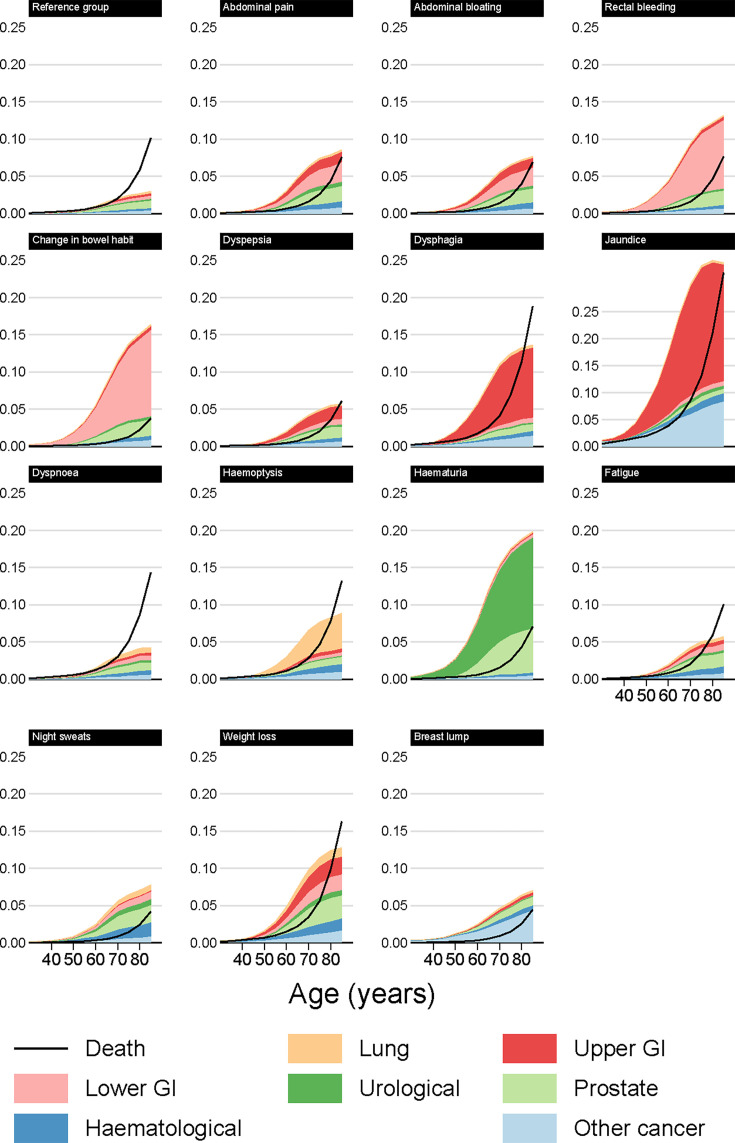
Modelled cancer and mortality risk at 12 months by index symptom, male non-smokers.

**Figure 3:**
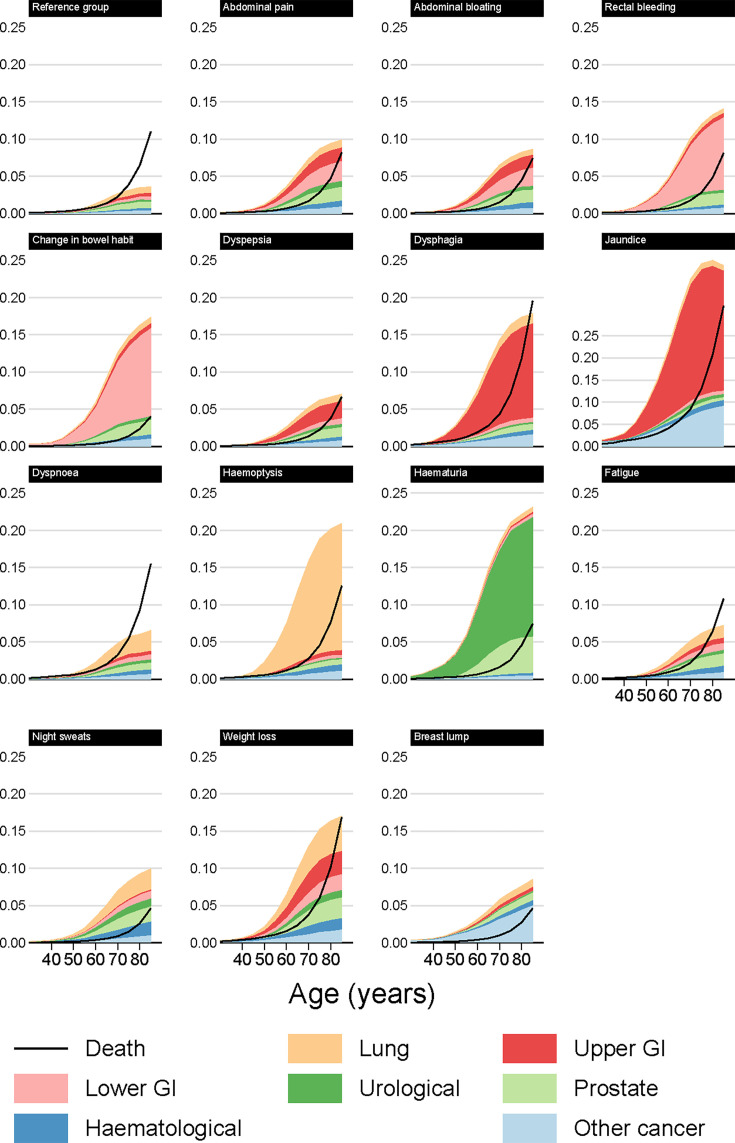
Modelled cancer and mortality risk at 12 months by index symptom, male smokers.

**Figure 4:**
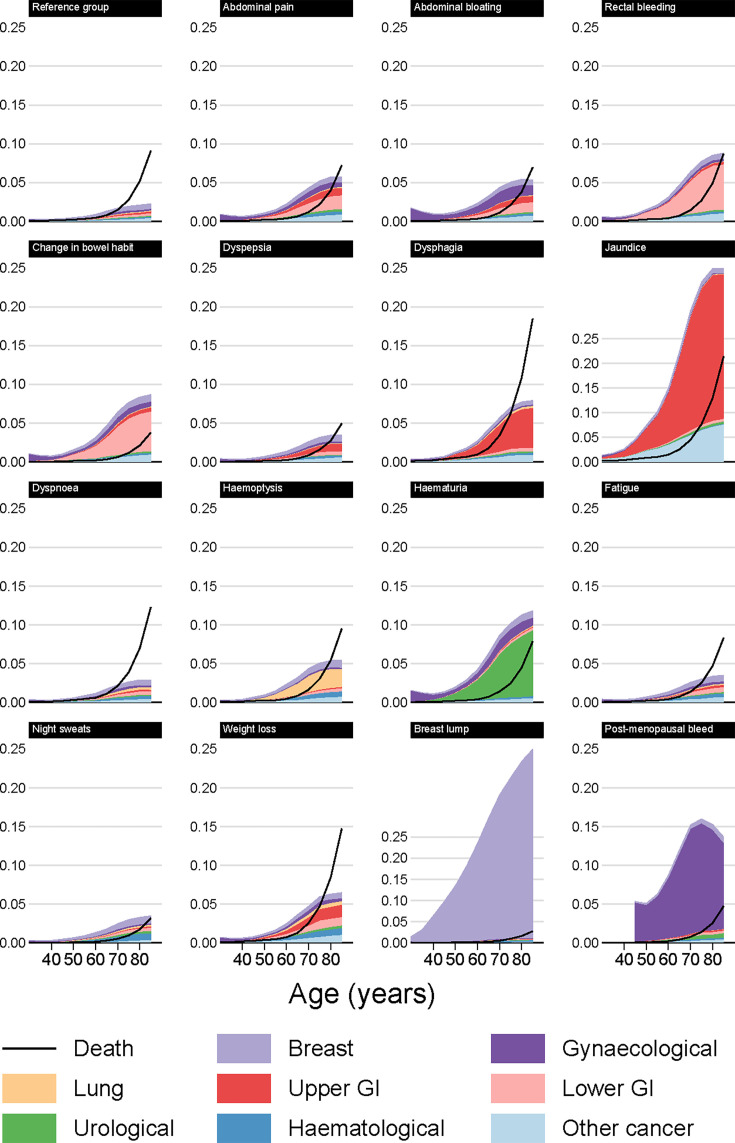
Modelled cancer and mortality risk at 12 months by index symptom, female non-smokers.

Further, for many symptoms associated with a very high initial hazard of a specific cancer, while the hazard typically remained elevated at least 12 months after the index consultation, it tended to reduce over time. For example, online supplemental appendix 3 table 1 shows HRs for lung cancer. The HR for haemoptysis is 17.1, but there is a statistically significant interaction with (the natural log of) follow-up time in months with an HR of 0.7; by 12 months the HR for lung cancer is estimated to have decreased to around 7.3. This fits with the non-parametric results shown in the Aalen–Johansen plots, where after haemoptysis presentation diagnoses of lung cancer rapidly accrue until about 3 months follow-up, after which they continue growing but less rapidly (online supplemental appendix 2 figures 1–4).

#### Abdominal symptoms (abdominal pain, abdominal bloating, rectal bleeding, change in bowel habit, dyspepsia, dysphagia, jaundice)

For both men and women presentations with abdominal symptoms were associated with increased hazard of multiple types of cancer, particularly lower GI cancer (online supplemental appendix 3 tables 3 and 13) and upper GI cancer (online supplemental appendix 3 tables 2 and 12). At the same time, abdominal symptoms were associated with decreased hazard of death without a cancer diagnosis when compared with the reference group, except for dysphagia and jaundice (figure 1 and online supplemental appendix 3 tables 8 and 17). Cause-specific HRs at 1 month after the presentation were highest regarding lower GI cancer for rectal bleeding (eg, for men: HR 17.4, 95% CI 15.7 to 19.4) and change in bowel habit (eg, for men: HR 21.5, 95% CI 19.0 to 24.3) and highest regarding upper GI cancer for jaundice (eg, for women: HR 122, 95% CI 102 to 147) and dysphagia (eg, for women: HR 16.4, 95% CI 14.0 to 19.2); HRs decreased substantially over follow-up for these symptoms. Abdominal pain and abdominal bloating were associated with HRs at the consultation of around four for both upper and lower GI cancers (eg, abdominal bloating in women with HR for lower GI cancer of 3.0, 95% CI 2.3 to 4.0), with abdominal bloating having a similar association for gynaecological cancers in women (HR 4.8, 95% CI 4.0 to 5.6, online supplemental appendix 3 table 10), while dyspepsia was associated with an HR of around four for upper GI cancer. Patients with abdominal symptoms also appeared at elevated risk for urological and haematological cancers and for prostate and gynaecological cancers.

#### Respiratory symptoms (dyspnoea, haemoptysis)

Respiratory symptoms were primarily associated with lung cancer, but the strength of the association varied (figure 1 and online supplemental appendix 3 tables 1 and 11). Patients with haemoptysis had a cause-specific HR of around 16 at consultation compared with the reference group (eg, for men, HR 17.1, 95% CI 14.8 to 19.8), while the association with dyspnoea was weaker but still notable (eg, for men, HR 2.6, 95% CI 2.4 to 2.9). Other types of cancer, notably haematological cancers, also had elevated cause-specific hazards after presentation with haemoptysis (eg, for men, the HR for haematological cancer being 2.8, 95% CI 1.7 to 4.6, online supplemental appendix 3 table 6) or dyspnoea (HR 1.7, 95% CI 1.5 to 1.8).

#### Urological symptoms (haematuria)

Haematuria in women was primarily associated with urological cancers (HR 57, 95% CI 48 to 67) and with gynaecological cancers (HR 4.6, 95% CI 3.7 to 5.6) (figure 1 and online supplemental appendix 3 tables 10 and 14). In men, it was associated with urological cancers (HR 45, 95% CI 40 to 50) and prostate cancer (HR 5.3, 95% CI 4.8 to 5.8) (online supplemental appendix 3 tables 4 and 5).

#### Non-specific symptoms (fatigue, night sweats, weight loss)

Non-specific symptoms were typically associated with elevated cause-specific HRs for all cancer groups considered (figure 1 and online supplemental appendix 3) and generally HRs appeared relatively similar in strength for each of the three non-specific symptoms. Weight loss had the strongest associations overall (cancer-specific HRs general between 2 and 5), followed by night sweats (HRs generally between 1 and 4, though imprecisely estimated), followed by fatigue (HRs between 1 and 2). It often appeared that the strongest cause-specific associations were for haematological cancers, though CIs tended to overlap with those of other cancer groups.

#### Breast and reproductive organ symptoms (breast lump, post-menopausal bleeding)

Post-menopausal bleeding was associated with large cause-specific HRs for gynaecological cancer (HR 43, 95% CI 39 to 47) and substantial cause-specific HRs for urological cancer (HR 4.1, 95% CI 2.6 to 6.4) (figure 1 and online supplemental appendix 3 tables 10 and 14). Breast lump in women was associated principally with breast cancer (HR 65, 95% CI 61 to 69) and to a lesser extent with haematological cancer (HR 2.6, 95% CI 1.80 to 3.6) (online supplemental appendix 3 tables 9 and 15). A small number of men present with breast lump, and these men had cause-specific HRs for the ‘other cancer’ group, which included male breast cancer, of 7.1 (95% CI 5.0 to 10.0) (online supplemental appendix 3 table 7).

### Risk of specific cancer sites by age, sex and smoking status

**Figure 5:**
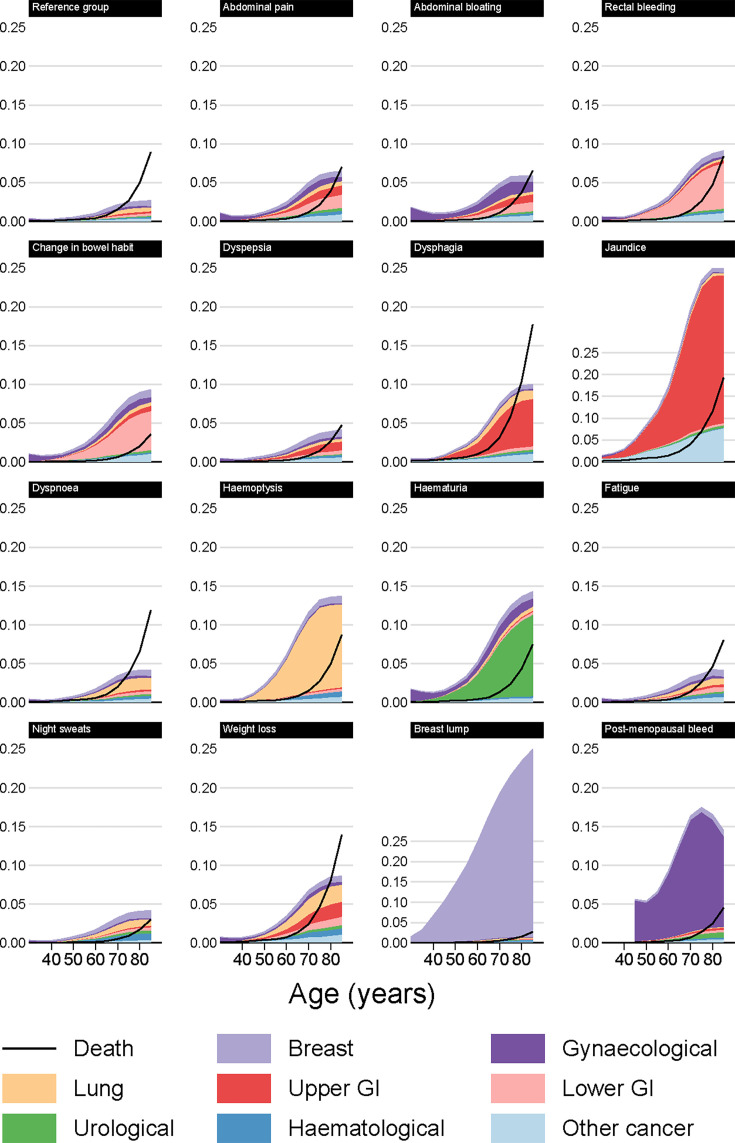
Modelled cancer and mortality risk at 12 months by index symptom, female smokers.

After symptom presentation for patients with single index symptoms and based on simulations combining the cause-specific models, we present simulated cumulative incidence of each cancer site and of death without cancer at 3 months (online supplemental appendix 4 figures 1–4), 6 months (, online supplemental appendix 4 figures 5–8) and 12 months (figures2^5^, online supplemental appendix 5). Hereafter in this section, we discuss cumulative incidence at 12 months after symptom consultation. Unlike the HRs presented above, estimates of cumulative incidence varied substantially by sex, as women have lower baseline cancer risk.

#### 3% any cancer risk thresholds at 12 months

Patients reaching a 3% risk of any cancer may not reach such a risk level for any specific cancer group, especially for symptoms associated with multiple types of cancer. For example, female smokers presenting with weight loss had a 3% risk of cancer from age 60, but did not reach the 3% risk threshold at any age when any of the individual cancer groups were considered on their own (table 2). For male non-smokers, the risk of any cancer reached the 3% threshold from the following ages and onwards: 45 for jaundice; 55 for dysphagia, weight loss, haematuria and change in bowel habit; 60 for haemoptysis and rectal bleeding; 65 for abdominal pain and bloating, night sweats and breast lump; and 70 for dyspepsia, dyspnoea and fatigue. For smokers, this threshold was often reached up to 5 years younger. Conversely, compared with male patients presenting with the same symptom, female patients reached the 3% threshold at an older age on average, with the main exception being breast lump for which the 3% threshold (in women) was reached from age 40.

**Table 2:** Modelled age at which patients presenting with each symptom had a 3% risk (ie, high enough to trigger urgent referral for suspected cancer in England) of all cancers combined and of specific cancer sites, by smoking status and sex.

Men	Never-smokers	Ever-smokers
Reference group	Any cancer (90)	Any cancer (75)
Abdominal pain	Any cancer (60)	Any cancer (60)
Abdominal bloating	Any cancer (65)	Any cancer (60)
Rectal bleeding	Any cancer (60); lower GI (65)	Any cancer (60); lower GI (60)
Change in bowel habit	Any cancer (55); lower GI (60)	Any cancer (55); lower GI (60)
Dyspepsia	Any cancer (65)	Any cancer (65)
Dysphagia	Any cancer (55); upper GI (60)	Any cancer (55); upper GI (55)
Jaundice	Any cancer (45); upper GI (50); other (55)	Any cancer (45); upper GI (50); other (55)
Dyspnoea	Any cancer (70)	Any cancer (65)
Haemoptysis	Any cancer (60); lung (70)	Any cancer (55); lung (55)
Haematuria	Any cancer (55); urological (55); prostate (65)	Any cancer (50); urological (55); prostate (70)
Fatigue	Any cancer (65)	Any cancer (65)
Night sweats	Any cancer (65)	Any cancer (60)
Weight loss	Any cancer (60); prostate (80)	Any cancer (55); lung (70); upper GI (75)
Breast lump	Any cancer (65); other (75)	Any cancer (60); other (70)
**Women**	**Never-smokers**	**Ever-smokers**
Reference group		
Abdominal pain	Any cancer (65)	Any cancer (65)
Abdominal bloating	Any cancer (65)	Any cancer (65)
Rectal bleeding	Any cancer (60); lower GI (70)	Any cancer (60); lower GI (70)
Change in bowel habit	Any cancer (60); lower GI (70)	Any cancer (60); lower GI (70)
Dyspepsia	Any cancer (75)	Any cancer (70)
Dysphagia	Any cancer (65); upper GI (70)	Any cancer (60); upper GI (70)
Jaundice	Any cancer (45); upper GI (50); other (60)	Any cancer (40); upper GI (45); other (55)
Dyspnoea		Any cancer (70)
Haemoptysis	Any cancer (65)	Any cancer (55); lung (60)
Haematuria	Any cancer (60); urological (65)	Any cancer (55); urological (60)
Fatigue	Any cancer (75)	Any cancer (70)
Night sweats	Any cancer (75)	Any cancer (70)
Weight loss	Any cancer (65)	Any cancer (60)
Breast lump	Any cancer (35); breast (40)	Any cancer (35); breast (35)
Post-menopausal bleeding	Any cancer (30); gynaecological (30)	Any cancer (30); gynaecological (30)

GI, gastrointestinal tract.

Notably, male smokers in the reference group had a 3% risk of any cancer from age 75, and male non-smokers from age 90; women in the reference group did not reach a 3% risk of cancer at any age.

A summary of risk of individual cancers is given in online supplemental appendix 6, plus additional graphical and tabular results in Appendices 4 and 5.

### Risk of non-cancer mortality

For most of the studied symptoms, symptomatic patients were less likely to die (without a cancer diagnosis) than similar patients in the reference group (figures2^5^). The three principal exceptions were jaundice, dysphagia and weight loss, for which post-presentation mortality exceeded that in the reference group, and also older patients with less-specific symptoms for whom the risk of non-cancer mortality was often higher than the risk of any cancer. For example, for male smokers presenting with dyspnoea, around 6% who presented at age 80 would develop cancer within 12 months, while 9% would die (figure 3, online supplemental appendix 5 table 1).

### Presentation with multiple symptoms

Among symptomatic patients, 1.2% (10 360 of 835 995) consulted for more than one of the 15 studied symptoms on their index date, and a further 2.5% (21 167) consulted for an additional studied symptom within 30 days of an index symptom but before a cancer diagnosis (table 3). The proportion of patients with multiple index symptoms subsequently diagnosed with cancer within 12 months of the index (4.6%, 95% CI 4.2% to 5.1%) was higher than for patients with a single index symptom (3.5%, 95% CI 3.5% to 3.5%). This higher risk of cancer in patients with multiple index symptoms appeared applicable to many of the symptoms considered, but sample size limitations meant proportions developing cancer could often not be estimated precisely.

**Table 3:** Summary of cancer outcomes for patients with multiple different recorded symptoms at index presentation and within 30 days of index symptom.

Index symptom	Any other symptoms at the index	Patients	Cancers within 12 months of the index		
			N	%	(95% CI)
Any	No	825 635	28 834	3.5%	(3.5%, 3.5%)
	Yes	10 360	480	4.6%	(4.2%, 5.1%)
	Within 30 days*	21 167	1429	6.8%	(6.4%, 7.1%)
Abdominal pain	No	231 598	5510	2.4%	(2.3%, 2.4%)
	Yes	2335	101	4.3%	(3.6%, 5.2%)
	Within 30 days*	6122	379	6.2%	(5.6%, 6.8%)
Abdominal bloating	No	825 635	28 834	3.5%	(3.5%, 3.5%)
	Yes	10 360	480	4.6%	(4.2%, 5.1%)
	Within 30 days*	21 167	1429	6.8%	(6.4%, 7.1%)
Rectal bleeding	No	47 774	1831	3.8%	(3.7%, 4.0%)
	Yes	741	38	5.1%	(3.8%, 7.0%)
	Within 30 days*	1116	61	5.5%	(4.3%, 7.0%)
Change in bowel habit	No	16 857	1042	6.2%	(5.8%, 6.6%)
	Yes	355	25	7.0%	(4.8%, 10.2%)
	Within 30 days*	520	77	14.8%	(12.0%, 18.1%)
Dyspepsia	No	106 843	2090	2.0%	(1.9%, 2.0%)
	Yes	1645	35	2.1%	(1.5%, 2.9%)
	Within 30 days*	3282	219	6.7%	(5.9%, 7.6%)
Dysphagia	No	14 760	1021	6.9%	(6.5%, 7.3%)
	Yes	232	17	7.3%	(4.6%, 11.4%)
	Within 30 days*	1054	56	5.3%	(4.1%, 6.8%)
Jaundice	No	1759	450	25.6%	(23.6%, 27.7%)
	Yes	58	9	15.5%	(8.4%, 26.9%)
	Within 30 days*	81	17	21.0%	(13.5%, 31.1%)
Dyspnoea	No	139 758	3899	2.8%	(2.7%, 2.9%)
	Yes	1336	61	4.6%	(3.6%, 5.8%)
	Within 30 days*	2655	173	6.5%	(5.6%, 7.5%)
Haemoptysis	No	5750	406	7.1%	(6.4%, 7.8%)
	Yes	109	6	5.5%	(2.5%, 11.5%)
	Within 30 days*	198	20	10.1%	(6.6%, 15.1%)
Haematuria	No	25 438	2749	10.8%	(10.4%, 11.2%)
	Yes	315	22	7.0%	(4.7%, 10.3%)
	Within 30 days*	636	76	12.0%	(9.7%, 14.7%)
Fatigue	No	140 212	2353	1.7%	(1.6%, 1.7%)
	Yes	1720	58	3.4%	(2.6%, 4.3%)
	Within 30 days*	3132	157	5.0%	(4.3%, 5.8%)
Night sweats	No	7527	128	1.7%	(1.4%, 2.0%)
	Yes	148	5	3.4%	(1.5%, 7.7%)
	Within 30 days*	162	6	3.7%	(1.7%, 7.8%)
Weight loss	No	19 168	1193	6.2%	(5.9%, 6.6%)
	Yes	449	52	11.6%	(8.9%, 14.9%)
	Within 30 days*	725	90	12.4%	(10.2%, 15.0%)
Breast lump	No	38 045	4765	12.5%	(12.2%, 12.9%)
	Yes	262	25	9.5%	(6.5%, 13.7%)
	Within 30 days*	345	18	5.2%	(3.3%, 8.1%)
Post-menopausal bleed	No	8092	784	9.7%	(9.1%, 10.4%)
	Yes	80	10	12.5%	(6.9%, 21.5%)
	Within 30 days*	145	14	9.7%	(5.8%, 15.6%)

* Subset of patients without other symptoms at the index

## Discussion

Using a cohort design, we comprehensively estimated the risk of different cancer diagnoses and non-cancer mortality following presentation in primary care with one of 15 index symptoms and in a reference group that was not selected based on symptom status and so should approximate the risk in the general population. There was considerable variation in the risk by age and by sex. Smoking status was highly informative for cancer risk of patients with respiratory or non-organ-specific symptoms. Smokers typically reached the 3% threshold warranting referral for cancer investigations up to 5 years younger than non-smokers. The findings highlight the importance of including smoking status in clinical guidelines and referral decisions in patients with a new-onset symptom. Even symptoms with strong, well-established associations with specific cancer often have notable associations with other types of cancer. One example is dyspnoea, which is typically considered a symptom of lung cancer, but we find is also associated with an HR for haematological cancers of around 1.7 in both men and women. We also provide estimates of cancer risk while considering the potential for non-cancer mortality. For the oldest patients—and those with symptoms such as dysphagia or jaundice—the risk of death without a cancer diagnosis reached or exceeded the risk of cancer.

### Strengths and weaknesses

Key strengths of the study are (a) the large representative dataset, allowing examination of a range of both common and rare symptoms and outcomes; (b) the joint estimation of the risks of different outcomes, including non-cancer mortality and risk of different types of cancer; and (c) the use of cancer registry data to ascertain the presence of cancer, as cancer may be under- or over-recorded in non-registry sources.^23^ While this study represents the most comprehensive and detailed description of the risk of cancer in symptomatic patients to date, there are various areas where future work could make further improvements.

Our study covers a period from 2007 to 2017, during which there have been many secular changes such as the introduction of public health education campaigns to raise awareness of symptoms of possible cancer among members of the public, alongside changes in clinical guidelines for referral for suspected cancer, and how NHS diagnostic services are configured, but we have not examined secular trends in estimated risks. Further, the study only considers deaths in patients without cancer, but it may be important to understand if patients die quickly after a cancer diagnosis. Our measure of the smoking status does not allow for a refined appreciation of smoking history and dose-response relationships. Additionally, our analytical approach only allowed each patient to be included once, not making full use of the longitudinal nature of EHR datasets.^24^ We did not consider interactions between symptoms and simulated outcomes for patients with a single symptom only, in part due to only a few patients having multiple symptoms. We did not have access to free-text data, despite evidence that coded data does not capture all symptoms.^11 12^ Finally, we only examined 15 symptoms, ignoring the many other symptoms and important health conditions that may be associated with the risk of cancer.^1 17 25^ A more detailed examination of potential limitations is given in online supplemental appendix 7.

### Comparison with literature

A large and growing literature describes the risk of cancer following symptom presentations in primary care; Moore and colleagues summarised the literature pre-2020,^17^ and there are several recent papers.^26^,^29^ Existing literature (a) rarely considers competing non-cancer mortality risk, (b) rarely considers smoking status and (c) frequently provides no or only limited information on the age-dependent and sex-specific nature of the risk of different cancers. Much of the previous evidence additionally considers either the risk of all cancers combined or focuses on specific cancer sites judged to be of relevance to the specific examined symptoms *a priori*. We improve on previous descriptive studies by presenting a broad range of possible cancer diagnoses following presentation with a wider spectrum of index symptoms. Further research is needed to extend analyses similar to those reported here to a wider collection of symptoms.

Some existing evidence on the so-called red-flag symptoms such as rectal bleeding and haemoptysis suggests the risk of cancer exceeds 3% for all ages but did not examine the risk in different age groups^17^; our findings indicate that the risk of cancer following these symptoms only exceeds 3% beyond certain age cut-offs. Furthermore, we show that for non-specific symptoms, the risk of any cancer exceeds 3% at a considerably earlier age than the risk of a specific cancer type, underscoring the need for studies that comprehensively examine all major cancer types. Weight loss provides a cardinal example, where the risk of any cancer exceeded 3% in male non-smokers from age 55, but the risk of any individual site only reached 3% at age 85.

Other studies have aimed to develop risk prediction tools for cancer intended for use in a primary care setting (see, eg,^30^,^32^), and in particular the QCancer risk prediction tool^33 34^ already considers a range of symptoms and a risk of a diagnosis of different types of cancer. For decisions about the management of an individual patient, a risk prediction tool including multiple potential predictors may be more suitable than the results presented in this paper. We view our results as complementary; by describing what is effectively the average risk in patients presenting with these symptoms (by age, sex and smoking status), we can inform high-level policy decisions around the symptomatic diagnosis of cancer such as clinical guideline recommendations and help developers of more detailed risk prediction models by highlighting symptoms they may wish to consider. Further, our consideration of mortality risk provides relevant information that is frequently missing from current risk prediction tools (including QCancer) and that is especially important in frail and elderly populations.

### Implications

Symptoms recorded in primary care data can be highly informative about both cancer risk and short-term mortality risk. In some cases, for example, lung cancer, smoking status is very strongly associated with the risk of cancer following a certain symptom. The risk of cancer and non-cancer mortality varies considerably by age; describing the ‘overall’ risk of cancer following a symptom may be misleading if non-cancer mortality is not considered. Some (non-cancer) deaths will relate to as-yet undiagnosed diseases which, like a cancer diagnosis, necessitates specialist assessment in secondary care, though this should be the subject of future inquiries.

For researchers, our results underline the methodological importance of accounting for the fact that symptoms may be associated with multiple different disease outcomes. Advanced statistical modelling strategies are helpful in assessing diagnostic outcomes using EHR data, and current statistical packages allow for relatively straightforward handling of competing risks either by directly modelling cumulative incidence (eg, the Fine-Grey model^35^) or, as here, by combining several cause-specific models.^36^ Diagnostic research should adopt strategies that allow consideration of the risk of several potentially related diseases (eg, multiple types of cancer, as in this study), which can be done even with simple analytical approaches such as the appropriate use of logistic regression.^29^

For clinicians and policymakers, our systematic assessment of the risk of cancer (and of non-cancer mortality) in symptomatic patients in primary care raises two key questions.

First, whether all age-sex-smoking status groups presenting with each of the studied symptoms and with an estimated any-cancer risk of above 3% should explicitly be added to NICE referral guidelines. This may indeed be justified, though given the high mortality rates in the oldest patients, there might also be a risk of over-testing in older men in particular. However, the degree to which the risk of over-testing is a concern relates to the exact causes of non-cancer mortality and the extent to which it relates to pre-diagnosed or new non-neoplastic diseases which could benefit from specialist diagnostic assessment and earlier diagnosis. As the components of non-cancer mortality due to pre-existing or new conditions are unclear, this should be addressed in future research. The current approach to cancer referral uses a normative threshold applicable to patients of any age and with any symptoms, and the results highlight the importance of considering whether patients are likely to benefit from prompt diagnosis.

Second, whether current referral pathways are necessarily ideal. For example, many abdominal symptoms were strongly associated with lower GI, upper GI and gynaecological cancers, and some form of referral pathway offering combined multi-specialty assessment may be justified for patients with these symptoms. Further, symptoms were often strongly associated with less common cancers such as haematological neoplasms; however, due to the low incidence of these conditions, the absolute risk rarely or never reached 3%; optimal diagnostic management of these patients is clearly challenging. Our findings may be helpful in clarifying referral criteria for new non-specific cancer pathways.

## Conclusions

The risk of cancer diagnosis and non-cancer mortality after symptomatic presentation can be comparable and both should be considered in referral and investigation decisions—alongside age, sex and smoking status. A holistic and stratified assessment of the risk in symptomatic patients, which considers the risk of a cancer diagnosis, the risk of a diagnosis of individual types of cancer and the risk of non-cancer mortality, is needed particularly for patients presenting with which are vague or non-specific symptoms associated with multiple cancer types and appreciable non-cancer mortality risk. Our results can support the updating of referral and management guidelines for symptomatic patients presenting in primary care.

## Supplementary material

10.1136/bmjonc-2024-000500 online supplemental appendix 1

## Data Availability

Data may be obtained from a third party and are not publicly available.
