# Supplementary material for: Cancer incidence and competing mortality risk following 15 presenting symptoms in primary care: a population-based cohort study using electronic healthcare records
Source: BMJ Oncol. 2024 Nov 21;3(1):e000500. doi: 10.1136/bmjonc-2024-000500 (PMC11660325; doi:10.1136/bmjonc-2024-000500)
Supplement: online supplemental appendix 1 [file bmjonc-3-1-s001.pdf]

Supplementary material

Contents

Appendix 1. Supplementary methods. 2
Appendix 2. Additional non-parametric and descriptive results. 8
Appendix 3. Details of the outcome-specific models. 35
Appendix 4. Figures showing simulated outcomes at 3 and 6 months after index symptom by age, sex and smoking status. 52
Appendix 5. Table showing simulated outcomes at 12 months after index symptom by age, sex and smoking status. 61
Appendix 6. Additional detail on the risk of specific cancer sites. 99
Appendix 7. Additional detail on the limitations of this study 101

Appendix 1. Supplementary methods.

Details of simulation approach

The simulations in this study used to estimate absolute risk of cancer (or of non-cancer death) over elapsed time since index symptom are based solely on the Royston-Parmar time-to-event models summarised in Appendix 3.

The simulations do this in a brute-force manner. We produce an imaginary patient with a given set of covariates (e.g., a 70 year old smoker with presenting with haemoptysis). We simulate follow-up time for this patient up to 12 months for each of our outcome models, so we will simulate a event date for each of the eight relevant outcomes (diagnosis with cancer of one of seven sites, or death without a cancer diagnosis). This imaginary patient may have multiple simulated event dates, or they may reach the end of the year without any simulated event dates at all.

We then look for the first simulated event this patient has, and we consider the patient to have had that event.

We repeat for a large number of imaginary patients with the same set of covariates, in our case 100,000 patients for each covariate set.

This combination of models and simulation of outcomes is facilitated by the multistate package.

This means that estimated cancer risks draw solely on the estimates reported by our study, and do not draw in any information on cancer risk from other sources.

Appendix 1 Figure 1A. Cohort flowchart for the reference group.

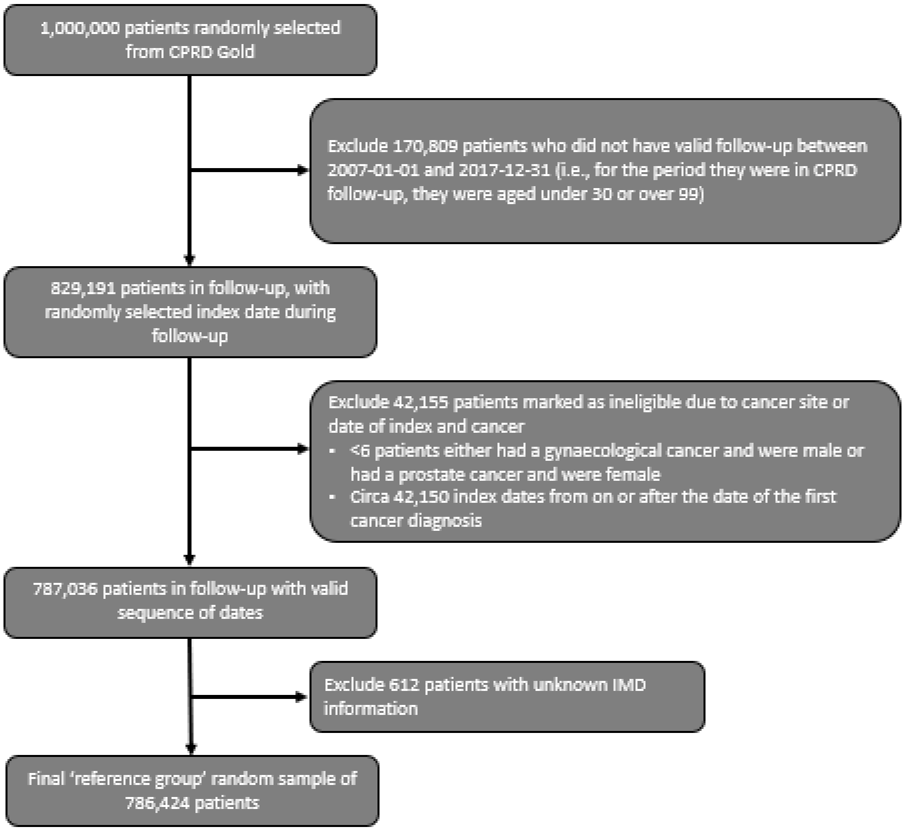

Appendix 1 Figure 1B. Cohort flowchart for the symptomatic cohort and the final analysis cohort.

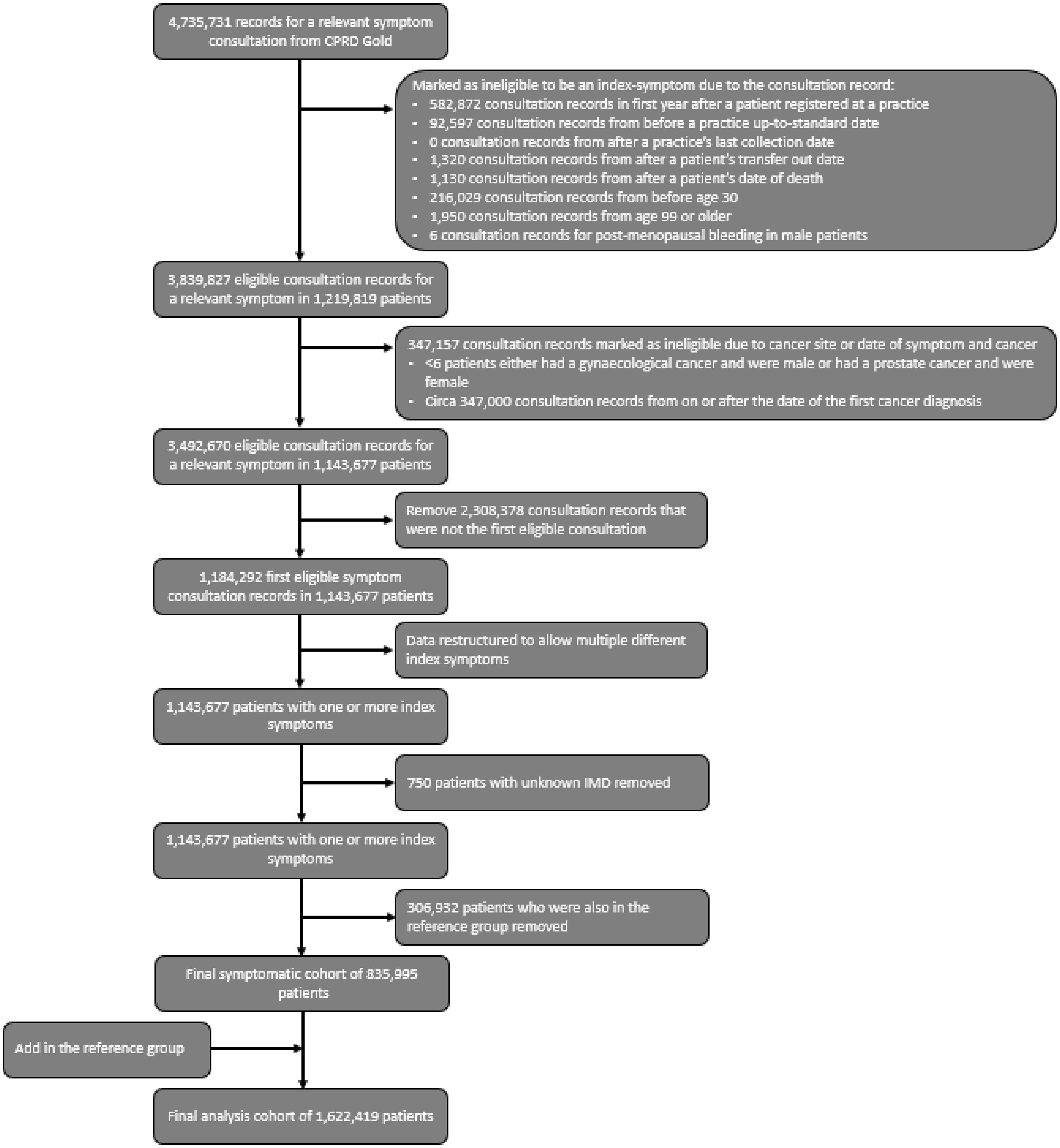

Appendix 1 Table 1. Counts of recorded symptoms for patients in the reference group in the year either side of their index date.

Reference group (N = 786,424)	Occurring On index date N	(% of total)	Year before index N	% of total	Year after index N	% of total
Any symptom	*455*	*(0.1%)*	*106,045*	*(13.5%)*	*95,096*	*(12.1%)*
Abdominal pain	136	(0.0%)	34,192	(4.3%)	29,101	(3.7%)
Abdominal bloating	12	(0.0%)	3,816	(0.5%)	3,475	(0.4%)
Rectal bleeding	21	(0.0%)	6,430	(0.8%)	5,684	(0.7%)
Change in bowel habit	<10		2,306	(0.3%)	2,145	(0.3%)
Dyspepsia	60	(0.0%)	16,138	(2.1%)	13,909	(1.8%)
Dysphagia	<10		2,154	(0.3%)	2,214	(0.3%)
Jaundice	<10		208	(0.0%)	307	(0.0%)
Dyspnoea	118	(0.0%)	25,469	(3.2%)	25,194	(3.2%)
Haemoptysis	<10		901	(0.1%)	852	(0.1%)
Haematuria	15	(0.0%)	3,185	(0.4%)	3,111	(0.4%)
Fatigue	69	(0.0%)	19,738	(2.5%)	16,907	(2.1%)
Night sweats	<10		1,179	(0.1%)	965	(0.1%)
Weight loss	14	(0.0%)	2,802	(0.4%)	2,689	(0.3%)
Breast lump	10	(0.0%)	4,505	(0.6%)	4,201	(0.5%)
Post-menopausal bleeding	<10		917	(0.1%)	908	(0.1%)

Appendix 1 Table 2 . Cancer site codelist.

Analysis grouping	Cancer site - specific	ICD10 code
Breast	Breast	C50
Breast	Breast (in-situ)	D05
Gynaecological	Cervix	C53
Gynaecological	Cervix (in-situ)	D06
Gynaecological	Ovary	C56
Gynaecological	Ovary	C57
Gynaecological	Uterus	C54
Gynaecological	Uterus	C55
Gynaecological	Vulva	C51
Lung	Lung	C33
Lung	Lung	C34
Lung	Mesothelioma	C45
Upper GI	Liver	C22
Upper GI	Oesophagus	C15
Upper GI	Pancreas	C25
Upper GI	Stomach	C16
Lower GI	Colon	C18
Lower GI	Colon	C19
Lower GI	Rectum	C20
Urological	Bladder	C67
Urological	Bladder (in-situ)	D09
Urological	Kidney	C64
Urological	Other and unspecified urinary	C65
Urological	Other and unspecified urinary	C66
Urological	Other and unspecified urinary	C68
Prostate	Prostate	C61
Haematological	Acute myeloid leukaemia	C920
Haematological	Acute myeloid leukaemia	C924
Haematological	Acute myeloid leukaemia	C925
Haematological	Acute myeloid leukaemia	C930
Haematological	Acute myeloid leukaemia	C940
Haematological	Acute myeloid leukaemia	C942
Haematological	Chronic lymphocytic leukaemia	C911
Haematological	Hodgkin lymphoma	C81
Haematological	Multiple myeloma	C90
Haematological	Non-Hodgkin lymphoma	C82
Haematological	Non-Hodgkin lymphoma	C83
Haematological	Non-Hodgkin lymphoma	C84
Haematological	Non-Hodgkin lymphoma	C85
Haematological	Other haematological	C88
Haematological	Other haematological	C912
Haematological	Other haematological	C913
Haematological	Other haematological	C914
Haematological	Other haematological	C915
Haematological	Other haematological	C917
Haematological	Other haematological	C919
Haematological	Other haematological	C922
Haematological	Other haematological	C923
Haematological	Other haematological	C927
Haematological	Other haematological	C929
Haematological	Other haematological	C931
Haematological	Other haematological	C932
Haematological	Other haematological	C937
Haematological	Other haematological	C939
Haematological	Other haematological	C943
Haematological	Other haematological	C944
Haematological	Other haematological	C945
Haematological	Other haematological	C947
Haematological	Other haematological	C95
Haematological	Other haematological	C96
Haematological	Other leukaemia	C910
Haematological	Other leukaemia	C921
Other cancer	Bone sarcoma	C40
Other cancer	Bone sarcoma	C41
Other cancer	Brain	C71
Other cancer	Brain	D330
Other cancer	Brain	D331
Other cancer	Brain	D332
Other cancer	Brain	D430
Other cancer	Brain	D431
Other cancer	Brain	D432
Other cancer	Connective and soft tissue sarcoma	C48
Other cancer	Connective and soft tissue sarcoma	C49
Other cancer	Larynx	C32
Other cancer	Melanoma	C43

Analysis grouping	Cancer site - specific	ICD10 code
Other cancer	Meninges	C70
Other cancer	Meninges	D32
Other cancer	Meninges	D42
Other cancer	Non-specific head and neck	C00
Other cancer	Non-specific head and neck	C14
Other cancer	Non-specific head and neck	C31
Other cancer	Oral cavity	C02
Other cancer	Oral cavity	C03
Other cancer	Oral cavity	C04
Other cancer	Oral cavity	C06
Other cancer	Oropharynx	C01
Other cancer	Oropharynx	C09
Other cancer	Oropharynx	C10
Other cancer	Other CNS and intracranial	C720
Other cancer	Other CNS and intracranial	C721
Other cancer	Other CNS and intracranial	C722
Other cancer	Other CNS and intracranial	C723
Other cancer	Other CNS and intracranial	C724
Other cancer	Other CNS and intracranial	C725
Other cancer	Other CNS and intracranial	C751
Other cancer	Other CNS and intracranial	C752
Other cancer	Other CNS and intracranial	C753
Other cancer	Other CNS and intracranial	D333
Other cancer	Other CNS and intracranial	D334
Other cancer	Other CNS and intracranial	D35
Other cancer	Other CNS and intracranial	D433
Other cancer	Other CNS and intracranial	D434
Other cancer	Other CNS and intracranial	D44
Other cancer	Other head and neck	C05
Other cancer	Other head and neck	C07
Other cancer	Other head and neck	C08
Other cancer	Other head and neck	C11
Other cancer	Other head and neck	C12
Other cancer	Other head and neck	C13
Other cancer	Other malignant neoplasms	C17
Other cancer	Other malignant neoplasms	C21
Other cancer	Other malignant neoplasms	C23
Other cancer	Other malignant neoplasms	C24
Other cancer	Other malignant neoplasms	C26
Other cancer	Other malignant neoplasms	C30
Other cancer	Other malignant neoplasms	C37
Other cancer	Other malignant neoplasms	C38
Other cancer	Other malignant neoplasms	C39
Other cancer	Other malignant neoplasms	C46
Other cancer	Other malignant neoplasms	C47
Other cancer	Other malignant neoplasms	C52
Other cancer	Other malignant neoplasms	C58
Other cancer	Other malignant neoplasms	C60
Other cancer	Other malignant neoplasms	C63
Other cancer	Other malignant neoplasms	C69
Other cancer	Other malignant neoplasms	C728
Other cancer	Other malignant neoplasms	C729
Other cancer	Other malignant neoplasms	C74
Other cancer	Other malignant neoplasms	C750
Other cancer	Other malignant neoplasms	C754
Other cancer	Other malignant neoplasms	C755
Other cancer	Other malignant neoplasms	C758
Other cancer	Other malignant neoplasms	C759
Other cancer	Other malignant neoplasms	C76
Other cancer	Other malignant neoplasms	C97
Other cancer	Testis	C62
Other cancer	Thyroid	C73
Other cancer	Unknown primary	C77
Other cancer	Unknown primary	C78
Other cancer	Unknown primary	C79
Other cancer	Unknown primary	C80

Appendix 2. Additional non-parametric and descriptive results.

Appendix 2 Table 1. Demographics of the reference group.

	Cohort		Cancers within 12 months		Deaths within 12 months, no preceding cancer diagnosis		Deaths within 12 months	
	N	(col %)	N	(row %)	N	(row %)	N	(row %)
Reference group	786,424		7,536	(1.0)	12,520	(1.6)	14,554	(1.9)
Age at index (grouped)								
30 to 39	227,649	(28.9)	448	(0.2)	253	(0.1)	268	(0.1)
40 to 49	176,160	(22.4)	567	(0.3)	427	(0.2)	471	(0.3)
50 to 59	139,629	(17.8)	997	(0.7)	591	(0.4)	752	(0.5)
60 to 69	111,947	(14.2)	1,751	(1.6)	1,210	(1.1)	1,579	(1.4)
70 to 79	72,807	(9.3)	1,963	(2.7)	2,443	(3.4)	3,060	(4.2)
80 to 89	46,311	(5.9)	1,524	(3.3)	4,875	(10.5)	5,544	(12.0)
90 to 99	11,921	(1.5)	286	(2.4)	2,721	(22.8)	2,880	(24.2)
Sex								
Female	392,101	(49.9)	3,871	(1.0)	6,886	(1.8)	7,847	(2.0)
Male	394,323	(50.1)	3,665	(0.9)	5,634	(1.4)	6,707	(1.7)
IMD group								
Least deprived	180,925	(23.0)	1,733	(1.0)	2,435	(1.3)	2,847	(1.6)
2	172,705	(22.0)	1,652	(1.0)	2,672	(1.5)	3,084	(1.8)
3	166,105	(21.1)	1,627	(1.0)	2,715	(1.6)	3,125	(1.9)
4	144,965	(18.4)	1,385	(1.0)	2,410	(1.7)	2,835	(2.0)
Most deprived	121,724	(15.5)	1,139	(0.9)	2,288	(1.9)	2,663	(2.2)
Any record of smoking								
Never smoker	312,309	(39.7)	2,139	(0.7)	4,688	(1.5)	5,189	(1.7)
Ever smoker	474,115	(60.3)	5,397	(1.1)	7,832	(1.7)	9,365	(2.0)

Appendix 2 Table 2. Demographics of the abdominal pain cohort.

	Cohort		Cancers within 12 months		Deaths within 12 months, no preceding cancer diagnosis		Deaths within 12 months	
	N	(col %)	N	(row %)	N	(row %)	N	(row %)
Abdominal pain	236,226		5,675	(2.4)	2,203	(0.9)	3,871	(1.6)
Age at index (grouped)								
30 to 39	64,571	(27.3)	342	(0.5)	52	(0.1)	77	(0.1)
40 to 49	56,250	(23.8)	469	(0.8)	93	(0.2)	164	(0.3)
50 to 59	44,055	(18.6)	925	(2.1)	149	(0.3)	354	(0.8)
60 to 69	36,512	(15.5)	1,613	(4.4)	242	(0.7)	714	(2.0)
70 to 79	22,760	(9.6)	1,470	(6.5)	490	(2.2)	973	(4.3)
80 to 89	10,243	(4.3)	740	(7.2)	771	(7.5)	1,110	(10.8)
90 to 99	1,835	(0.8)	116	(6.3)	406	(22.1)	479	(26.1)
Sex								
Female	144,132	(61.0)	3,000	(2.1)	1,240	(0.9)	2,093	(1.5)
Male	92,094	(39.0)	2,675	(2.9)	963	(1.0)	1,778	(1.9)
IMD group								
Least deprived	56,443	(23.9)	1,423	(2.5)	431	(0.8)	806	(1.4)
2	51,856	(22.0)	1,289	(2.5)	449	(0.9)	822	(1.6)
3	49,851	(21.1)	1,219	(2.4)	485	(1.0)	850	(1.7)
4	42,010	(17.8)	969	(2.3)	447	(1.1)	763	(1.8)
Most deprived	36,066	(15.3)	775	(2.1)	391	(1.1)	630	(1.7)
Any record of smoking								
Never smoker	83,292	(35.3)	1,695	(2.0)	701	(0.8)	1,164	(1.4)
Ever smoker	152,934	(64.7)	3,980	(2.6)	1,502	(1.0)	2,707	(1.8)

Appendix 2 Table 3. Demographics of the abdominal bloating cohort.

	Cohort		Cancers within 12 months		Deaths within 12 months, no preceding cancer diagnosis		Deaths within 12 months	
	N	(col %)	N	(row %)	N	(row %)	N	(row %)
Abdominal bloating	23,228		660	(2.8)	272	(1.2)	457	(2.0)
Age at index (grouped)								
30 to 39	4,671	(20.1)	27	(0.6)	<10		10	(0.2)
40 to 49	5,274	(22.7)	47	(0.9)	<10		14	(0.3)
50 to 59	4,517	(19.4)	101	(2.2)	26	(0.6)	39	(0.9)
60 to 69	3,973	(17.1)	165	(4.2)	32	(0.8)	81	(2.0)
70 to 79	3,024	(13.0)	214	(7.1)	52	(1.7)	117	(3.9)
80 to 89	1,516	(6.5)	90	(5.9)	100	(6.6)	135	(8.9)
90 to 99	253	(1.1)	16	(6.3)	51	(20.2)	61	(24.1)
Sex								
Female	15,297	(65.9)	395	(2.6)	163	(1.1)	263	(1.7)
Male	7,931	(34.1)	265	(3.3)	109	(1.4)	194	(2.4)
IMD group								
Least deprived	5,833	(25.1)	180	(3.1)	53	(0.9)	101	(1.7)
2	5,280	(22.7)	167	(3.2)	69	(1.3)	119	(2.3)
3	4,774	(20.6)	149	(3.1)	60	(1.3)	101	(2.1)
4	4,021	(17.3)	91	(2.3)	48	(1.2)	70	(1.7)
Most deprived	3,320	(14.3)	73	(2.2)	42	(1.3)	66	(2.0)
Any record of smoking								
Never smoker	8,169	(35.2)	229	(2.8)	106	(1.3)	170	(2.1)
Ever smoker	15,059	(64.8)	431	(2.9)	166	(1.1)	287	(1.9)

Appendix 2 Table 4. Demographics of the rectal bleeding cohort.

	Cohort		Cancers within 12 months		Deaths within 12 months, no preceding cancer diagnosis		Deaths within 12 months	
	N	(col %)	N	(row %)	N	(row %)	N	(row %)
Rectal bleeding	49,273		1,896	(3.8)	875	(1.8)	1,098	(2.2)
Age at index (grouped)								
30 to 39	10,567	(21.4)	39	(0.4)	15	(0.1)	15	(0.1)
40 to 49	10,772	(21.9)	133	(1.2)	31	(0.3)	37	(0.3)
50 to 59	9,738	(19.8)	315	(3.2)	54	(0.6)	70	(0.7)
60 to 69	8,320	(16.9)	504	(6.1)	71	(0.9)	107	(1.3)
70 to 79	5,605	(11.4)	524	(9.3)	147	(2.6)	209	(3.7)
80 to 89	3,452	(7.0)	330	(9.6)	355	(10.3)	435	(12.6)
90 to 99	819	(1.7)	51	(6.2)	202	(24.7)	225	(27.5)
Sex								
Female	21,575	(43.8)	788	(3.7)	505	(2.3)	598	(2.8)
Male	27,698	(56.2)	1,108	(4.0)	370	(1.3)	500	(1.8)
IMD group								
Least deprived	12,398	(25.2)	481	(3.9)	171	(1.4)	219	(1.8)
2	11,110	(22.5)	445	(4.0)	183	(1.6)	233	(2.1)
3	10,403	(21.1)	417	(4.0)	224	(2.2)	282	(2.7)
4	8,384	(17.0)	313	(3.7)	160	(1.9)	196	(2.3)
Most deprived	6,978	(14.2)	240	(3.4)	137	(2.0)	168	(2.4)
Any record of smoking								
Never smoker	17,338	(35.2)	558	(3.2)	337	(1.9)	399	(2.3)
Ever smoker	31,935	(64.8)	1,338	(4.2)	538	(1.7)	699	(2.2)

Appendix 2 Table 5. Demographics of the change in bowel habit cohort.

	Cohort		Cancers within 12 months		Deaths within 12 months, no preceding cancer diagnosis		Deaths within 12 months	
	N	(col %)	N	(row %)	N	(row %)	N	(row %)
Change in bowel habit	17,629		1,091	(6.2)	165	(0.9)	366	(2.1)
Age at index (grouped)								
30 to 39	1,420	(8.1)	12	(0.8)	<10		<10	
40 to 49	2,645	(15.0)	34	(1.3)	<10		<10	
50 to 59	3,960	(22.5)	159	(4.0)	<10		17	(0.4)
60 to 69	4,144	(23.5)	288	(6.9)	17	(0.4)	60	(1.4)
70 to 79	3,441	(19.5)	363	(10.5)	33	(1.0)	109	(3.2)
80 to 89	1,785	(10.1)	209	(11.7)	66	(3.7)	128	(7.2)
90 to 99	234	(1.3)	26	(11.1)	36	(15.4)	46	(19.7)
Sex								
Female	9,205	(52.2)	443	(4.8)	86	(0.9)	175	(1.9)
Male	8,424	(47.8)	648	(7.7)	79	(0.9)	191	(2.3)
IMD group								
Least deprived	5,048	(28.6)	306	(6.1)	41	(0.8)	91	(1.8)
2	4,176	(23.7)	249	(6.0)	38	(0.9)	85	(2.0)
3	3,872	(22.0)	235	(6.1)	36	(0.9)	84	(2.2)
4	2,739	(15.5)	193	(7.0)	29	(1.1)	62	(2.3)
Most deprived	1,794	(10.2)	108	(6.0)	21	(1.2)	44	(2.5)
Any record of smoking								
Never smoker	5,970	(33.9)	324	(5.4)	50	(0.8)	109	(1.8)
Ever smoker	11,659	(66.1)	767	(6.6)	115	(1.0)	257	(2.2)

Appendix 2 Table 6. Demographics of the dyspepsia cohort.

	Cohort		Cancers within 12 months		Deaths within 12 months, no preceding cancer diagnosis		Deaths within 12 months	
	N	(col %)	N	(row %)	N	(row %)	N	(row %)
Dyspepsia	110,312		2,178	(2.0)	976	(0.9)	1,605	(1.5)
Age at index (grouped)								
30 to 39	19,949	(18.1)	63	(0.3)	19	(0.1)	24	(0.1)
40 to 49	23,004	(20.9)	132	(0.6)	44	(0.2)	67	(0.3)
50 to 59	22,461	(20.4)	323	(1.4)	86	(0.4)	156	(0.7)
60 to 69	23,092	(20.9)	617	(2.7)	120	(0.5)	269	(1.2)
70 to 79	14,584	(13.2)	656	(4.5)	228	(1.6)	431	(3.0)
80 to 89	6,281	(5.7)	341	(5.4)	326	(5.2)	477	(7.6)
90 to 99	941	(0.9)	46	(4.9)	153	(16.3)	181	(19.2)
Sex								
Female	56,689	(51.4)	982	(1.7)	488	(0.9)	750	(1.3)
Male	53,623	(48.6)	1,196	(2.2)	488	(0.9)	855	(1.6)
IMD group								
Least deprived	25,136	(22.8)	503	(2.0)	158	(0.6)	289	(1.1)
2	23,702	(21.5)	484	(2.0)	216	(0.9)	364	(1.5)
3	23,169	(21.0)	472	(2.0)	212	(0.9)	336	(1.5)
4	20,214	(18.3)	389	(1.9)	208	(1.0)	333	(1.6)
Most deprived	18,091	(16.4)	330	(1.8)	182	(1.0)	283	(1.6)
Any record of smoking								
Never smoker	36,203	(32.8)	575	(1.6)	282	(0.8)	428	(1.2)
Ever smoker	74,109	(67.2)	1,603	(2.2)	694	(0.9)	1,177	(1.6)

Appendix 2 Table 7. Demographics of the dysphagia cohort.

	Cohort		Cancers within 12 months		Deaths within 12 months, no preceding cancer diagnosis		Deaths within 12 months	
	N	(col %)	N	(row %)	N	(row %)	N	(row %)
Dysphagia	15,291		1,053	(6.9)	1,181	(7.7)	1,638	(10.7)
Age at index (grouped)								
30 to 39	1,390	(9.1)	<10		<10		<10	
40 to 49	2,182	(14.3)	<50		<50		<50	
50 to 59	2,749	(18.0)	146	(5.3)	36	(1.3)	87	(3.2)
60 to 69	3,015	(19.7)	288	(9.6)	61	(2.0)	166	(5.5)
70 to 79	2,722	(17.8)	291	(10.7)	186	(6.8)	320	(11.8)
80 to 89	2,420	(15.8)	232	(9.6)	541	(22.4)	672	(27.8)
90 to 99	813	(5.3)	45	(5.5)	331	(40.7)	360	(44.3)
Sex								
Female	7,750	(50.7)	401	(5.2)	707	(9.1)	882	(11.4)
Male	7,541	(49.3)	652	(8.6)	474	(6.3)	756	(10.0)
IMD group								
Least deprived	3,606	(23.6)	229	(6.4)	250	(6.9)	346	(9.6)
2	3,390	(22.2)	241	(7.1)	261	(7.7)	365	(10.8)
3	3,293	(21.5)	212	(6.4)	264	(8.0)	353	(10.7)
4	2,745	(18.0)	196	(7.1)	229	(8.3)	317	(11.5)
Most deprived	2,257	(14.8)	175	(7.8)	177	(7.8)	257	(11.4)
Any record of smoking								
Never smoker	5,613	(36.7)	244	(4.3)	568	(10.1)	673	(12.0)
Ever smoker	9,678	(63.3)	809	(8.4)	613	(6.3)	965	(10.0)

Appendix 2 Table 8. Demographics of the jaundice cohort.

	Cohort		Cancers within 12 months		Deaths within 12 months, no preceding cancer diagnosis		Deaths within 12 months	
	N	(col %)	N	(row %)	N	(row %)	N	(row %)
Jaundice	1,898		468	(24.7)	221	(11.6)	510	(26.9)
Age at index (grouped)								
30 to 39	195	(10.3)	<10		10	(5.1)	10	(5.1)
40 to 49	271	(14.3)	<20		27	(10.0)	31	(11.4)
50 to 59	296	(15.6)	46	(15.5)	53	(17.9)	75	(25.3)
60 to 69	385	(20.3)	115	(29.9)	36	(9.4)	105	(27.3)
70 to 79	379	(20.0)	143	(37.7)	28	(7.4)	123	(32.5)
80 to 89	288	(15.2)	115	(39.9)	38	(13.2)	115	(39.9)
90 to 99	84	(4.4)	28	(33.3)	29	(34.5)	51	(60.7)
Sex								
Female	876	(46.2)	228	(26.0)	105	(12.0)	253	(28.9)
Male	1,022	(53.8)	240	(23.5)	116	(11.4)	257	(25.1)
IMD group								
Least deprived	407	(21.4)	106	(26.0)	36	(8.8)	97	(23.8)
2	410	(21.6)	122	(29.8)	43	(10.5)	111	(27.1)
3	391	(20.6)	93	(23.8)	56	(14.3)	111	(28.4)
4	353	(18.6)	76	(21.5)	35	(9.9)	89	(25.2)
Most deprived	337	(17.8)	71	(21.1)	51	(15.1)	102	(30.3)
Any record of smoking								
Never smoker	646	(34.0)	152	(23.5)	79	(12.2)	182	(28.2)
Ever smoker	1,252	(66.0)	316	(25.2)	142	(11.3)	328	(26.2)

Appendix 2 Table 9. Demographics of the dyspnoea cohort.

	Cohort		Cancers within 12 months		Deaths within 12 months, no preceding cancer diagnosis		Deaths within 12 months	
	N	(col %)	N	(row %)	N	(row %)	N	(row %)
Dyspnoea	142,431		4,015	(2.8)	6,308	(4.4)	7,820	(5.5)
Age at index (grouped)								
30 to 39	12,307	(8.6)	65	(0.5)	40	(0.3)	46	(0.4)
40 to 49	17,115	(12.0)	123	(0.7)	84	(0.5)	110	(0.6)
50 to 59	22,521	(15.8)	344	(1.5)	220	(1.0)	298	(1.3)
60 to 69	32,649	(22.9)	978	(3.0)	708	(2.2)	1,030	(3.2)
70 to 79	32,722	(23.0)	1,397	(4.3)	1,604	(4.9)	2,139	(6.5)
80 to 89	21,205	(14.9)	976	(4.6)	2,597	(12.2)	3,058	(14.4)
90 to 99	3,912	(2.7)	132	(3.4)	1,055	(27.0)	1,139	(29.1)
Sex								
Female	72,982	(51.2)	1,735	(2.4)	3,181	(4.4)	3,804	(5.2)
Male	69,449	(48.8)	2,280	(3.3)	3,127	(4.5)	4,016	(5.8)
IMD group								
Least deprived	28,212	(19.8)	812	(2.9)	1,173	(4.2)	1,478	(5.2)
2	30,026	(21.1)	863	(2.9)	1,335	(4.4)	1,675	(5.6)
3	29,691	(20.8)	809	(2.7)	1,348	(4.5)	1,646	(5.5)
4	27,696	(19.4)	787	(2.8)	1,266	(4.6)	1,561	(5.6)
Most deprived	26,806	(18.8)	744	(2.8)	1,186	(4.4)	1,460	(5.4)
Any record of smoking								
Never smoker	31,607	(22.2)	678	(2.1)	1,598	(5.1)	1,814	(5.7)
Ever smoker	110,824	(77.8)	3,337	(3.0)	4,710	(4.2)	6,006	(5.4)

Appendix 2 Table 10. Demographics of the haemoptysis cohort.

	Cohort		Cancers within 12 months		Deaths within 12 months, no preceding cancer diagnosis		Deaths within 12 months	
	N	(col %)	N	(row %)	N	(row %)	N	(row %)
Haemoptysis	5,984		417	(7.0)	147	(2.5)	330	(5.5)
Age at index (grouped)								
30 to 39	1,031	(17.2)	<10		<10		<10	
40 to 49	1,069	(17.9)	<20		<10		<10	
50 to 59	1,110	(18.5)	50	(4.5)	<10		21	(1.9)
60 to 69	1,252	(20.9)	122	(9.7)	16	(1.3)	57	(4.6)
70 to 79	929	(15.5)	133	(14.3)	39	(4.2)	112	(12.1)
80 to 89	506	(8.5)	78	(15.4)	50	(9.9)	98	(19.4)
90 to 99	87	(1.5)	12	(13.8)	25	(28.7)	33	(37.9)
Sex								
Female	2,159	(36.1)	132	(6.1)	48	(2.2)	107	(5.0)
Male	3,825	(63.9)	285	(7.5)	99	(2.6)	223	(5.8)
IMD group								
Least deprived	1,219	(20.4)	77	(6.3)	27	(2.2)	65	(5.3)
2	1,245	(20.8)	85	(6.8)	30	(2.4)	65	(5.2)
3	1,221	(20.4)	68	(5.6)	35	(2.9)	61	(5.0)
4	1,136	(19.0)	83	(7.3)	35	(3.1)	75	(6.6)
Most deprived	1,163	(19.4)	104	(8.9)	20	(1.7)	64	(5.5)
Any record of smoking								
Never smoker	1,462	(24.4)	35	(2.4)	39	(2.7)	52	(3.6)
Ever smoker	4,522	(75.6)	382	(8.4)	108	(2.4)	278	(6.1)

Appendix 2 Table 11. Demographics of the haematuria cohort.

	Cohort		Cancers within 12 months		Deaths within 12 months, no preceding cancer diagnosis		Deaths within 12 months	
	N	(col %)	N	(row %)	N	(row %)	N	(row %)
Haematuria	26,051		2,791	(10.7)	601	(2.3)	987	(3.8)
Age at index (grouped)								
30 to 39	2,435	(9.3)	26	(1.1)	<10		<10	
40 to 49	3,668	(14.1)	95	(2.6)	<10		<20	
50 to 59	4,547	(17.5)	274	(6.0)	16	(0.4)	34	(0.7)
60 to 69	5,895	(22.6)	768	(13.0)	34	(0.6)	89	(1.5)
70 to 79	5,585	(21.4)	935	(16.7)	131	(2.3)	248	(4.4)
80 to 89	3,292	(12.6)	614	(18.7)	259	(7.9)	414	(12.6)
90 to 99	629	(2.4)	79	(12.6)	149	(23.7)	184	(29.3)
Sex								
Female	9,156	(35.1)	610	(6.7)	188	(2.1)	282	(3.1)
Male	16,895	(64.9)	2,181	(12.9)	413	(2.4)	705	(4.2)
IMD group								
Least deprived	6,621	(25.4)	754	(11.4)	140	(2.1)	242	(3.7)
2	6,040	(23.2)	648	(10.7)	129	(2.1)	208	(3.4)
3	5,721	(22.0)	590	(10.3)	150	(2.6)	230	(4.0)
4	4,301	(16.5)	444	(10.3)	94	(2.2)	162	(3.8)
Most deprived	3,368	(12.9)	355	(10.5)	88	(2.6)	145	(4.3)
Any record of smoking								
Never smoker	7,932	(30.4)	650	(8.2)	223	(2.8)	313	(3.9)
Ever smoker	18,119	(69.6)	2,141	(11.8)	378	(2.1)	674	(3.7)

Appendix 2 Table 12. Demographics of the fatigue cohort.

	Cohort		Cancers within 12 months		Deaths within 12 months, no preceding cancer diagnosis		Deaths within 12 months	
	N	(col %)	N	(row %)	N	(row %)	N	(row %)
Fatigue	143,719		2,476	(1.7)	2,247	(1.6)	3,015	(2.1)
Age at index (grouped)								
30 to 39	34,525	(24.0)	147	(0.4)	14	(0.0)	17	(0.0)
40 to 49	34,539	(24.0)	188	(0.5)	36	(0.1)	56	(0.2)
50 to 59	27,180	(18.9)	334	(1.2)	62	(0.2)	126	(0.5)
60 to 69	20,015	(13.9)	566	(2.8)	192	(1.0)	326	(1.6)
70 to 79	15,148	(10.5)	649	(4.3)	435	(2.9)	675	(4.5)
80 to 89	10,148	(7.1)	504	(5.0)	936	(9.2)	1,192	(11.7)
90 to 99	2,164	(1.5)	88	(4.1)	572	(26.4)	623	(28.8)
Sex								
Female	95,070	(66.1)	1,359	(1.4)	1,352	(1.4)	1,734	(1.8)
Male	48,649	(33.9)	1,117	(2.3)	895	(1.8)	1,281	(2.6)
IMD group								
Least deprived	35,032	(24.4)	606	(1.7)	499	(1.4)	693	(2.0)
2	32,240	(22.4)	562	(1.7)	508	(1.6)	660	(2.0)
3	30,756	(21.4)	525	(1.7)	497	(1.6)	668	(2.2)
4	25,159	(17.5)	456	(1.8)	408	(1.6)	538	(2.1)
Most deprived	20,532	(14.3)	327	(1.6)	335	(1.6)	456	(2.2)
Any record of smoking								
Never smoker	53,143	(37.0)	724	(1.4)	877	(1.7)	1,087	(2.0)
Ever smoker	90,576	(63.0)	1,752	(1.9)	1,370	(1.5)	1,928	(2.1)

Appendix 2 Table 13. Demographics of the night sweats cohort.

	Cohort		Cancers within 12 months		Deaths within 12 months, no preceding cancer diagnosis		Deaths within 12 months	
	N	(col %)	N	(row %)	N	(row %)	N	(row %)
Night sweats	7,857		137	(1.7)	31	(0.4)	67	(0.9)
Age at index (grouped)								
30 to 39	2,052	(26.1)	11	(0.5)	<10		<10	
40 to 49	2,053	(26.1)	11	(0.5)	<10		<10	
50 to 59	1,520	(19.3)	22	(1.4)	<10		<10	
60 to 69	1,238	(15.8)	42	(3.4)	<10		19	(1.5)
70 to 79	708	(9.0)	32	(4.5)	11	(1.6)	18	(2.5)
80 to 89	255	(3.2)	<20		<10		17	(6.7)
90 to 99	31	(0.4)	<10		<10		<10	
Sex								
Female	4,711	(60.0)	51	(1.1)	13	(0.3)	25	(0.5)
Male	3,146	(40.0)	86	(2.7)	18	(0.6)	42	(1.3)
IMD group								
Least deprived	1,929	(24.6)	30	(1.6)	<10		15	(0.8)
2	1,843	(23.5)	32	(1.7)	<10		<15	
3	1,627	(20.7)	26	(1.6)	10	(0.6)	18	(1.1)
4	1,351	(17.2)	26	(1.9)	<10		15	(1.1)
Most deprived	1,107	(14.1)	23	(2.1)	<10		<10	
Any record of smoking								
Never smoker	2,368	(30.1)	29	(1.2)	13	(0.5)	18	(0.8)
Ever smoker	5,489	(69.9)	108	(2.0)	18	(0.3)	49	(0.9)

Appendix 2 Table 14. Demographics of the weight loss cohort.

	Cohort		Cancers within 12 months		Deaths within 12 months, no preceding cancer diagnosis		Deaths within 12 months	
	N	(col %)	N	(row %)	N	(row %)	N	(row %)
Weight loss	20,163		1,291	(6.4)	1,196	(5.9)	1,846	(9.2)
Age at index (grouped)								
30 to 39	2,531	(12.6)	10	(0.4)	<10		<10	
40 to 49	2,950	(14.6)	31	(1.1)	<15		<50	
50 to 59	3,075	(15.3)	110	(3.6)	41	(1.3)	78	(2.5)
60 to 69	3,367	(16.7)	288	(8.6)	90	(2.7)	220	(6.5)
70 to 79	3,921	(19.4)	444	(11.3)	215	(5.5)	446	(11.4)
80 to 89	3,469	(17.2)	358	(10.3)	548	(15.8)	759	(21.9)
90 to 99	850	(4.2)	50	(5.9)	292	(34.4)	320	(37.6)
Sex								
Female	10,750	(53.3)	501	(4.7)	702	(6.5)	956	(8.9)
Male	9,413	(46.7)	790	(8.4)	494	(5.2)	890	(9.5)
IMD group								
Least deprived	4,217	(20.9)	268	(6.4)	248	(5.9)	359	(8.5)
2	4,312	(21.4)	302	(7.0)	261	(6.1)	408	(9.5)
3	4,194	(20.8)	251	(6.0)	279	(6.7)	401	(9.6)
4	3,880	(19.2)	260	(6.7)	201	(5.2)	336	(8.7)
Most deprived	3,560	(17.7)	210	(5.9)	207	(5.8)	342	(9.6)
Any record of smoking								
Never smoker	5,954	(29.5)	317	(5.3)	482	(8.1)	638	(10.7)
Ever smoker	14,209	(70.5)	974	(6.9)	714	(5.0)	1,208	(8.5)

Appendix 2 Table 15. Demographics of the breast lump cohort.

	Cohort		Cancers within 12 months		Deaths within 12 months, no preceding cancer diagnosis		Deaths within 12 months	
	N	(col %)	N	(row %)	N	(row %)	N	(row %)
Breast lump	38,623		4,814	(12.5)	90	(0.2)	277	(0.7)
Age at index (grouped)								
30 to 39	12,189	(31.6)	376	(3.1)	<10		<10	
40 to 49	14,080	(36.5)	1,184	(8.4)	<10		<20	
50 to 59	5,929	(15.4)	789	(13.3)	<10		23	(0.4)
60 to 69	3,056	(7.9)	740	(24.2)	<10		30	(1.0)
70 to 79	2,012	(5.2)	878	(43.6)	12	(0.6)	56	(2.8)
80 to 89	1,120	(2.9)	704	(62.9)	36	(3.2)	101	(9.0)
90 to 99	237	(0.6)	143	(60.3)	20	(8.4)	44	(18.6)
Sex								
Female	36,392	(94.2)	4,764	(13.1)	78	(0.2)	259	(0.7)
Male	2,231	(5.8)	50	(2.2)	12	(0.5)	18	(0.8)
IMD group								
Least deprived	10,572	(27.4)	1,337	(12.6)	19	(0.2)	59	(0.6)
2	8,857	(22.9)	1,122	(12.7)	14	(0.2)	57	(0.6)
3	7,925	(20.5)	1,020	(12.9)	20	(0.3)	50	(0.6)
4	6,296	(16.3)	747	(11.9)	18	(0.3)	64	(1.0)
Most deprived	4,973	(12.9)	588	(11.8)	19	(0.4)	47	(0.9)
Any record of smoking								
Never smoker	14,911	(38.6)	1,879	(12.6)	48	(0.3)	137	(0.9)
Ever smoker	23,712	(61.4)	2,935	(12.4)	42	(0.2)	140	(0.6)

Appendix 2 Table 16. Demographics of the post-menopausal bleeding cohort.

	Cohort		Cancers within 12 months		Deaths within 12 months, no preceding cancer diagnosis		Deaths within 12 months	
	N	(col %)	N	(row %)	N	(row %)	N	(row %)
Post-menopausal bleed	8,252		799	(9.7)	50	(0.6)	107	(1.3)
Age at index (grouped)								
30 to 39	<10		<10		<10		<10	
40 to 49	533	(6.5)	<10		<10		<10	
50 to 59	4,155	(50.4)	196	(4.7)	<10		<10	
60 to 69	2,091	(25.3)	312	(14.9)	<10		20	(1.0)
70 to 79	984	(11.9)	196	(19.9)	10	(1.0)	29	(2.9)
80 to 89	427	(5.2)	82	(19.2)	27	(6.3)	46	(10.8)
90 to 99	<100		<10		<10		<10	
Sex								
Female	8,252	(100.0)	799	(9.7)	50	(0.6)	107	(1.3)
IMD group								
Least deprived	2,317	(28.1)	203	(8.8)	14	(0.6)	32	(1.4)
2	1,948	(23.6)	174	(8.9)	14	(0.7)	25	(1.3)
3	1,749	(21.2)	178	(10.2)	15	(0.9)	25	(1.4)
4	1,296	(15.7)	146	(11.3)	<10		<20	
Most deprived	942	(11.4)	98	(10.4)	<10		<10	
Any record of smoking								
Never smoker	3,267	(39.6)	309	(9.5)	23	(0.7)	45	(1.4)
Ever smoker	4,985	(60.4)	490	(9.8)	27	(0.5)	62	(1.2)

Appendix 2 Table 17. Most common specific cancer sites in the 12 months following index by index symptom.

Index symptom	Incident cancers (first only)	Female never-smokers	Female ever-smokers	Male never-smokers	Male ever-smokers
Reference cohort	No cancer	164528	223702	145642	245016
Reference cohort	Lung	50	423	28	550
Reference cohort	Breast	359	540		7
Reference cohort	Prostate			216	613
Reference cohort	Colon	112	172	71	237
Reference cohort	Cervix (in-situ)	100	211		
Reference cohort	Unknown primary	55	104	25	94
Reference cohort	Non-Hodgkin lymphoma	49	64	36	114
Reference cohort	Pancreas	51	89	18	91
Reference cohort	Melanoma	43	57	48	90
Reference cohort	Rectum	43	62	26	94
Reference cohort	Bladder	26	38	23	119
Reference cohort	Oesophagus	16	53	26	108
Reference cohort	Stomach	31	36	25	108
Reference cohort	Ovary	79	99		
Reference cohort	Kidney	29	37	25	72
Reference cohort	Other malignant neoplasms	28	55	18	56
Reference cohort	Brain	25	39	27	55
Reference cohort	Uterus	65	74		
Reference cohort	Multiple myeloma	14	27	19	55
Reference cohort	Liver	21	23		63
Reference cohort	Chronic lymphocytic leukaemia	12	19	10	41
Reference cohort	Bladder (in-situ)		15	13	52
Reference cohort	Breast (in-situ)	32	43		
Reference cohort	Mesothelioma	6	5	14	42
Reference cohort	Acute myeloid leukaemia	12	15	8	31
Reference cohort	Oral cavity	8	15	5	27
Reference cohort	Thyroid	15	21	9	8
Reference cohort	Cervix	16	33		
Reference cohort	Connective and soft tissue sarcoma	13	13		19
Reference cohort	Meninges	19	17		9
Reference cohort	Oropharynx		14		25
Reference cohort	Other haematological	5	6	8	19
Reference cohort	Larynx		6		32
Reference cohort	Other CNS and intracranial	13	7	5	9
Reference cohort	Testis			19	14
Reference cohort	Other head and neck		12	6	12
Reference cohort	Vulva	9	20		
Reference cohort	Hodgkin lymphoma	8	5		15
Reference cohort	Other leukaemia		8	7	7
Reference cohort	Other and unspecified urinary		6		14
Reference cohort	Non-specific head and neck				5
Reference cohort	Aggregated (sites with counts <5)	21	3	19	4
Abdominal pain	No cancer	53508	86276	27390	61154
Abdominal pain	Colon	225	351	140	457
Abdominal pain	Prostate			135	327
Abdominal pain	Pancreas	72	148	49	169
Abdominal pain	Ovary	148	223		
Abdominal pain	Lung	22	132	12	156
Abdominal pain	Breast	110	194		
Abdominal pain	Non-Hodgkin lymphoma	60	74	33	100
Abdominal pain	Unknown primary	57	97	15	73
Abdominal pain	Other malignant neoplasms	40	70	14	77

Index symptom	Incident cancers (first only)	Female never-smokers	Female ever-smokers	Male never-smokers	Male ever-smokers
Abdominal pain	Stomach	26	41	27	105
Abdominal pain	Kidney	26	56	26	90
Abdominal pain	Cervix (in-situ)	41	139		
Abdominal pain	Oesophagus	19	39	24	90
Abdominal pain	Rectum	20	32	21	67
Abdominal pain	Liver	23	35	17	53
Abdominal pain	Uterus	55	51		
Abdominal pain	Bladder	12	24	12	58
Abdominal pain	Melanoma	27	25	8	19
Abdominal pain	Connective and soft tissue sarcoma	15	27	6	14
Abdominal pain	Multiple myeloma	10	19	9	24
Abdominal pain	Mesothelioma	6	9	9	19
Abdominal pain	Cervix	15	24		
Abdominal pain	Breast (in-situ)	7	25		
Abdominal pain	Bladder (in-situ)				32
Abdominal pain	Chronic lymphocytic leukaemia		6	5	16
Abdominal pain	Brain		8	5	10
Abdominal pain	Thyroid	8	11		
Abdominal pain	Acute myeloid leukaemia			8	11
Abdominal pain	Testis			9	7
Abdominal pain	Other leukaemia			5	6
Abdominal pain	Larynx				9
Abdominal pain	Other and unspecified urinary				8
Abdominal pain	Other haematological				8
Abdominal pain	Oral cavity				7
Abdominal pain	Meninges		7		
Abdominal pain	Other CNS and intracranial				6
Abdominal pain	Other head and neck				5
Abdominal pain	Aggregated (sites with counts <5)	30	24	14	14
Abdominal bloating	No cancer	5495	9048	2245	5213
Abdominal bloating	Ovary	55	64		
Abdominal bloating	Colon	14	25	12	32
Abdominal bloating	Prostate			17	28
Abdominal bloating	Pancreas	8	19		16
Abdominal bloating	Rectum	8	8		22
Abdominal bloating	Breast	11	24		
Abdominal bloating	Lung		9		14
Abdominal bloating	Cervix (in-situ)		20		
Abdominal bloating	Connective and soft tissue sarcoma	5	8		
Abdominal bloating	Other malignant neoplasms	5			8
Abdominal bloating	Oesophagus				12
Abdominal bloating	Liver				11
Abdominal bloating	Non-Hodgkin lymphoma				11
Abdominal bloating	Stomach		5		5
Abdominal bloating	Breast (in-situ)	7			
Abdominal bloating	Melanoma	6			
Abdominal bloating	Kidney	6			
Abdominal bloating	Uterus		6		
Abdominal bloating	Unknown primary	5			
Abdominal bloating	Aggregated (sites with counts <5)	24	36	32	30
Rectal bleeding	No cancer	8108	12371	8402	17766
Rectal bleeding	Rectum	81	137	101	317
Rectal bleeding	Colon	99	161	92	233
Rectal bleeding	Prostate			38	92

Index symptom	Incident cancers (first only)	Female never-smokers	Female ever-smokers	Male never-smokers	Male ever-smokers
Rectal bleeding	Other malignant neoplasms	14	37	6	18
Rectal bleeding	Breast	16	44		
Rectal bleeding	Lung		13		27
Rectal bleeding	Stomach	8	7		19
Rectal bleeding	Non-Hodgkin lymphoma	6	6	8	13
Rectal bleeding	Melanoma		14		11
Rectal bleeding	Unknown primary	6	10		7
Rectal bleeding	Uterus	8	11		
Rectal bleeding	Cervix (in-situ)	5	12		
Rectal bleeding	Kidney				15
Rectal bleeding	Ovary	7	6		
Rectal bleeding	Bladder		6		7
Rectal bleeding	Oesophagus				11
Rectal bleeding	Bladder (in-situ)				8
Rectal bleeding	Multiple myeloma				8
Rectal bleeding	Liver				6
Rectal bleeding	Pancreas				5
Rectal bleeding	Other head and neck				5
Rectal bleeding	Brain			5	
Rectal bleeding	Aggregated (sites with counts <5)	29	36	21	16
Change in bowel habit	No cancer	3188	5357	2331	5269
Change in bowel habit	Colon	53	76	51	161
Change in bowel habit	Rectum	42	51	51	162
Change in bowel habit	Prostate			12	60
Change in bowel habit	Breast	13	29		
Change in bowel habit	Ovary	15	24		
Change in bowel habit	Lung		16		21
Change in bowel habit	Pancreas	6	12		8
Change in bowel habit	Other malignant neoplasms	7	7	6	5
Change in bowel habit	Unknown primary		7		13
Change in bowel habit	Non-Hodgkin lymphoma		5		9
Change in bowel habit	Uterus	6	6		
Change in bowel habit	Oesophagus				7
Change in bowel habit	Liver				7
Change in bowel habit	Bladder				6
Change in bowel habit	Kidney				5
Change in bowel habit	Breast (in-situ)		5		
Change in bowel habit	Stomach				5
Change in bowel habit	Aggregated (sites with counts <5)	29	25	25	19
Dyspepsia	No cancer	20569	34203	14478	37118
Dyspepsia	Oesophagus	30	55	38	163
Dyspepsia	Stomach	20	44	34	114
Dyspepsia	Prostate			40	162
Dyspepsia	Breast	70	120		
Dyspepsia	Colon	34	50	24	70
Dyspepsia	Lung	7	55	5	103
Dyspepsia	Pancreas	32	33	8	48
Dyspepsia	Non-Hodgkin lymphoma	13	25	12	35
Dyspepsia	Unknown primary	14	27	7	30
Dyspepsia	Other malignant neoplasms	11	20	8	20
Dyspepsia	Kidney	9	5	9	28
Dyspepsia	Ovary	19	27		
Dyspepsia	Uterus	16	22		
Dyspepsia	Melanoma	13	13		12

Index symptom	Incident cancers (first only)	Female never-smokers	Female ever-smokers	Male never-smokers	Male ever-smokers
Dyspepsia	Cervix (in-situ)	9	26		
Dyspepsia	Liver	5	5		23
Dyspepsia	Bladder			5	27
Dyspepsia	Multiple myeloma	7	7	6	10
Dyspepsia	Rectum		11		17
Dyspepsia	Brain	6	6		9
Dyspepsia	Breast (in-situ)	7	13		
Dyspepsia	Chronic lymphocytic leukaemia		8		9
Dyspepsia	Oropharynx			5	9
Dyspepsia	Other CNS and intracranial				8
Dyspepsia	Bladder (in-situ)				8
Dyspepsia	Acute myeloid leukaemia				7
Dyspepsia	Larynx				7
Dyspepsia	Other leukaemia		6		
Dyspepsia	Meninges		6		
Dyspepsia	Thyroid		6		
Dyspepsia	Connective and soft tissue sarcoma				5
Dyspepsia	Mesothelioma				5
Dyspepsia	Aggregated (sites with counts <5)	12	27	19	20
Dysphagia	No cancer	3112	4085	2142	4617
Dysphagia	Oesophagus	67	134	59	298
Dysphagia	Stomach	7	16	20	72
Dysphagia	Lung		35		34
Dysphagia	Prostate			6	25
Dysphagia	Colon	12		5	9
Dysphagia	Breast	8	14		
Dysphagia	Non-Hodgkin lymphoma		5		14
Dysphagia	Unknown primary		5		9
Dysphagia	Larynx				11
Dysphagia	Other head and neck				11
Dysphagia	Oropharynx				10
Dysphagia	Thyroid		5		
Dysphagia	Ovary		5		
Dysphagia	Non-specific head and neck				5
Dysphagia	Aggregated (sites with counts <5)	33	49	23	30
Jaundice	No cancer	261	352	203	545
Jaundice	Pancreas	43	81	41	72
Jaundice	Liver	12	25	10	26
Jaundice	Other malignant neoplasms	7	20	10	33
Jaundice	Unknown primary	7	8	5	7
Jaundice	Lung				5
Jaundice	Aggregated (sites with counts <5)	6	13	7	18
Dyspnoea	No cancer	19397	51179	11162	55411
Dyspnoea	Lung	36	483	20	670
Dyspnoea	Colon	42	104	40	164
Dyspnoea	Prostate			64	283
Dyspnoea	Breast	73	216		5
Dyspnoea	Mesothelioma	7	15	27	115
Dyspnoea	Non-Hodgkin lymphoma	21	39	27	75
Dyspnoea	Unknown primary	16	53	10	82
Dyspnoea	Bladder		27	5	68
Dyspnoea	Kidney	11	29	10	39
Dyspnoea	Oesophagus	11	23	5	48
Dyspnoea	Stomach	9	11	11	54

Index symptom	Incident cancers (first only)	Female never-smokers	Female ever-smokers	Male never-smokers	Male ever-smokers
Dyspnoea	Pancreas	10	26		35
Dyspnoea	Ovary	18	47		
Dyspnoea	Other malignant neoplasms	7	22	6	28
Dyspnoea	Acute myeloid leukaemia	6	22	6	26
Dyspnoea	Multiple myeloma	10	14	10	25
Dyspnoea	Melanoma	7	17	6	20
Dyspnoea	Liver	5	6	5	33
Dyspnoea	Rectum	5	12		31
Dyspnoea	Uterus	12	28		
Dyspnoea	Larynx		8		30
Dyspnoea	Bladder (in-situ)		11		25
Dyspnoea	Cervix (in-situ)	8	27		
Dyspnoea	Chronic lymphocytic leukaemia		12	5	11
Dyspnoea	Breast (in-situ)	8	14		
Dyspnoea	Oropharynx		7		13
Dyspnoea	Brain	5			14
Dyspnoea	Oral cavity		7		11
Dyspnoea	Connective and soft tissue sarcoma		8		8
Dyspnoea	Other haematological		8		7
Dyspnoea	Thyroid	5	6		
Dyspnoea	Cervix		9		
Dyspnoea	Other leukaemia				7
Dyspnoea	Meninges		6		
Dyspnoea	Non-specific head and neck				5
Dyspnoea	Hodgkin lymphoma				5
Dyspnoea	Other head and neck				5
Dyspnoea	Aggregated (sites with counts <5)	31	18	33	15
Haemoptysis	No cancer	616	1362	780	2689
Haemoptysis	Lung	8	88	10	204
Haemoptysis	Prostate				10
Haemoptysis	Breast		9		
Haemoptysis	Larynx				6
Haemoptysis	Colon				5
Haemoptysis	Stomach				5
Haemoptysis	Aggregated (sites with counts <5)	10	17	7	33
Haematuria	No cancer	3095	5324	4097	10467
Haematuria	Bladder	64	162	139	708
Haematuria	Prostate			119	351
Haematuria	Bladder (in-situ)	22	63	75	288
Haematuria	Kidney	18	30	56	161
Haematuria	Other and unspecified urinary	14	17	20	50
Haematuria	Uterus	30	37		
Haematuria	Lung		19		42
Haematuria	Colon	6	5	6	27
Haematuria	Breast	18	16		
Haematuria	Unknown primary		7		15
Haematuria	Non-Hodgkin lymphoma		5		15
Haematuria	Other malignant neoplasms				10
Haematuria	Ovary		9		
Haematuria	Stomach				6
Haematuria	Cervix		6		
Haematuria	Melanoma				6
Haematuria	Multiple myeloma				6
Haematuria	Brain				5

Index symptom	Incident cancers (first only)	Female never-smokers	Female ever-smokers	Male never-smokers	Male ever-smokers
Haematuria	Rectum				5
Haematuria	Aggregated (sites with counts <5)	28	27	29	28
Fatigue	No cancer	37144	55527	14691	32165
Fatigue	Lung	21	144	12	170
Fatigue	Prostate			63	191
Fatigue	Breast	101	149		
Fatigue	Colon	60	79	28	83
Fatigue	Non-Hodgkin lymphoma	24	40	17	45
Fatigue	Cervix (in-situ)	28	74		
Fatigue	Brain	20	25	12	25
Fatigue	Unknown primary	21	25	7	26
Fatigue	Pancreas	16	21	7	24
Fatigue	Melanoma	19	18	6	20
Fatigue	Kidney	6	21	9	26
Fatigue	Other malignant neoplasms	9	20	6	16
Fatigue	Ovary	28	22		
Fatigue	Rectum	7	15	7	19
Fatigue	Liver	8	12	8	19
Fatigue	Acute myeloid leukaemia	7	17	6	16
Fatigue	Uterus	19	26		
Fatigue	Multiple myeloma	10	10	5	17
Fatigue	Bladder	5	10		25
Fatigue	Oesophagus		8		27
Fatigue	Stomach	5	10		18
Fatigue	Chronic lymphocytic leukaemia	6	6		16
Fatigue	Breast (in-situ)	12	15		
Fatigue	Meninges	7	13		
Fatigue	Mesothelioma			5	15
Fatigue	Hodgkin lymphoma	7	6		6
Fatigue	Connective and soft tissue sarcoma		7	5	6
Fatigue	Thyroid		12		
Fatigue	Other haematological				11
Fatigue	Bladder (in-situ)				7
Fatigue	Cervix		6		
Fatigue	Oral cavity		6		
Fatigue	Other CNS and intracranial		6		
Fatigue	Oropharynx				5
Fatigue	Aggregated (sites with counts <5)	28	20	28	24
Night sweats	No cancer	1454	3101	839	2148
Night sweats	Lung		7		16
Night sweats	Prostate				14
Night sweats	Breast	6	5		
Night sweats	Hodgkin lymphoma				7
Night sweats	Colon				6
Night sweats	Kidney				5
Night sweats	Aggregated (sites with counts <5)	9	23	14	21
Weight loss	No cancer	3477	6506	2036	6360
Weight loss	Lung	6	90	11	151
Weight loss	Colon	27	25	15	58
Weight loss	Prostate			31	93
Weight loss	Pancreas	12	26	17	37
Weight loss	Unknown primary	13	14	9	41
Weight loss	Stomach	10	23	5	37
Weight loss	Oesophagus	6	14	6	30

Index symptom	Incident cancers (first only)	Female never-smokers	Female ever-smokers	Male never-smokers	Male ever-smokers
Weight loss	Non-Hodgkin lymphoma	6	9	14	24
Weight loss	Breast	17	25		
Weight loss	Kidney	5			28
Weight loss	Rectum	8		5	17
Weight loss	Other malignant neoplasms	5	12		11
Weight loss	Liver	5		5	13
Weight loss	Multiple myeloma		6	6	8
Weight loss	Ovary	9	11		
Weight loss	Bladder		7		8
Weight loss	Mesothelioma				8
Weight loss	Brain				8
Weight loss	Chronic lymphocytic leukaemia				7
Weight loss	Hodgkin lymphoma				6
Weight loss	Cervix (in-situ)		6		
Weight loss	Bladder (in-situ)				6
Weight loss	Oral cavity				5
Weight loss	Other haematological				5
Weight loss	Larynx				5
Weight loss	Aggregated (sites with counts <5)	35	48	16	12
Breast lump	No cancer	12312	19045	615	1546
Breast lump	Breast	1741	2666	8	20
Breast lump	Breast (in-situ)	68	108		
Breast lump	Cervix (in-situ)	12	26		
Breast lump	Non-Hodgkin lymphoma	8	20		
Breast lump	Melanoma	6	7		
Breast lump	Lung		11		
Breast lump	Ovary		5		
Breast lump	Aggregated (sites with counts <5)	23	38	7	15
Post-menopausal bleed	No cancer	2932	4446		
Post-menopausal bleed	Uterus	231	356		
Post-menopausal bleed	Ovary	27	29		
Post-menopausal bleed	Cervix	14	31		
Post-menopausal bleed	Breast	5	19		
Post-menopausal bleed	Bladder		11		
Post-menopausal bleed	Cervix (in-situ)		5		
Post-menopausal bleed	Colon	5			
Post-menopausal bleed	Aggregated (sites with counts <5)	26	35		

Appendix 2 Figure 1. Aalen-Johansen estimates of cumulative incidence up to 12 months, male non-smokers

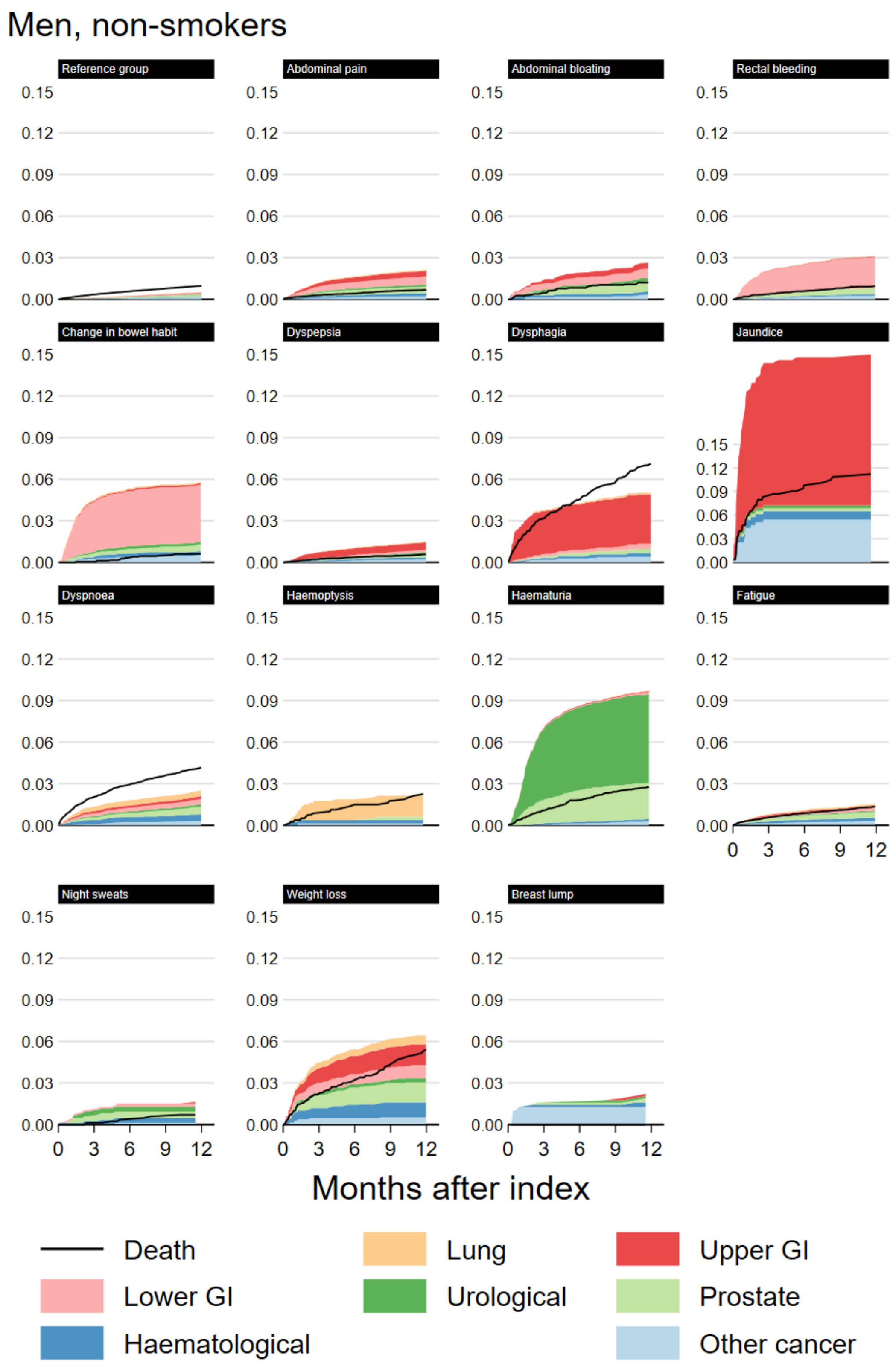

Appendix 2 Figure 2. Aalen-Johansen estimates of cumulative incidence up to 12 months, male smokers

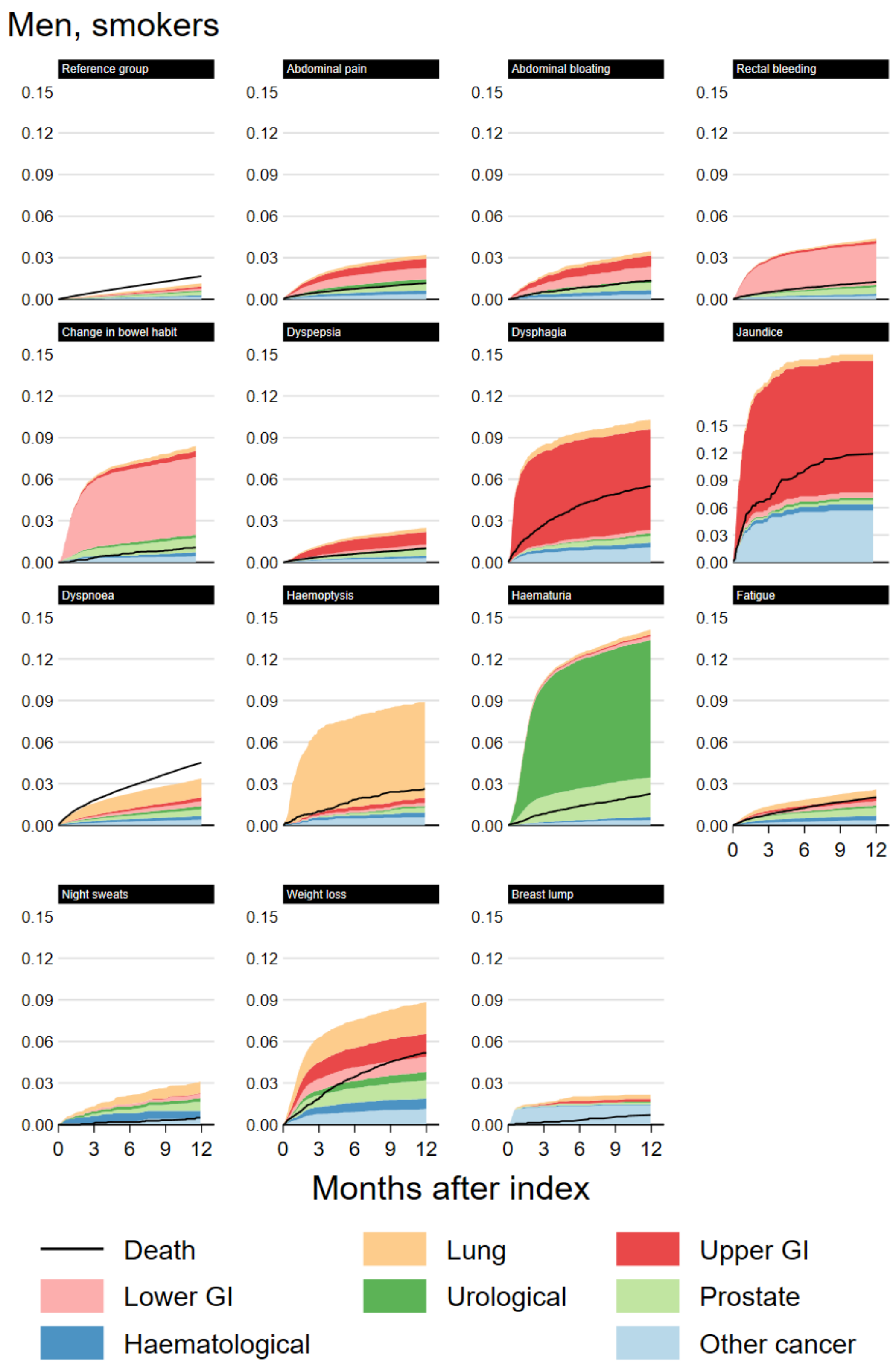

Appendix 2 Figure 3. Aalen-Johansen estimates of cumulative incidence up to 12 months, female non-smokers

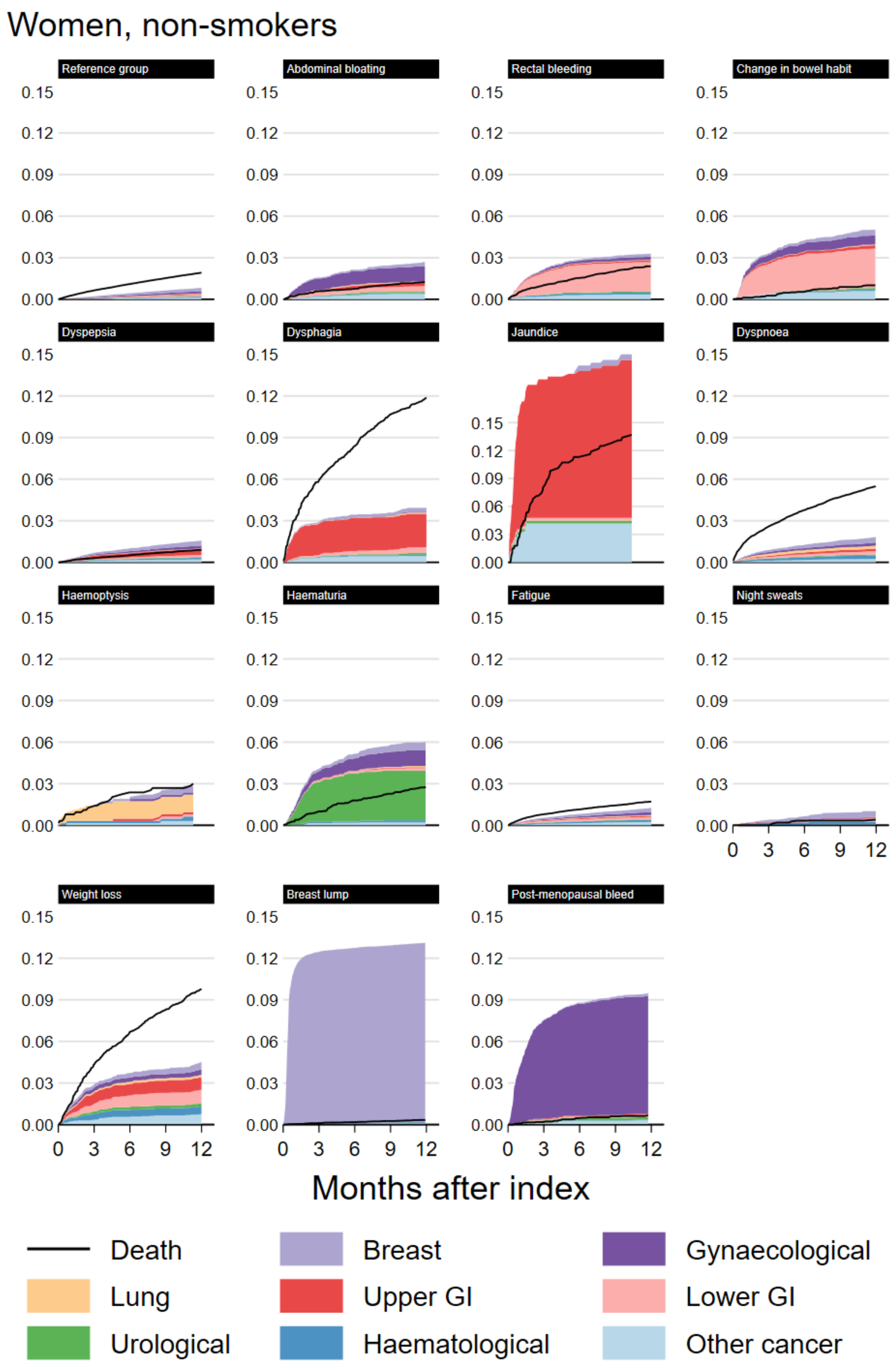

Appendix 2 Figure 4. Aalen-Johansen estimates of cumulative incidence up to 12 months, female smokers

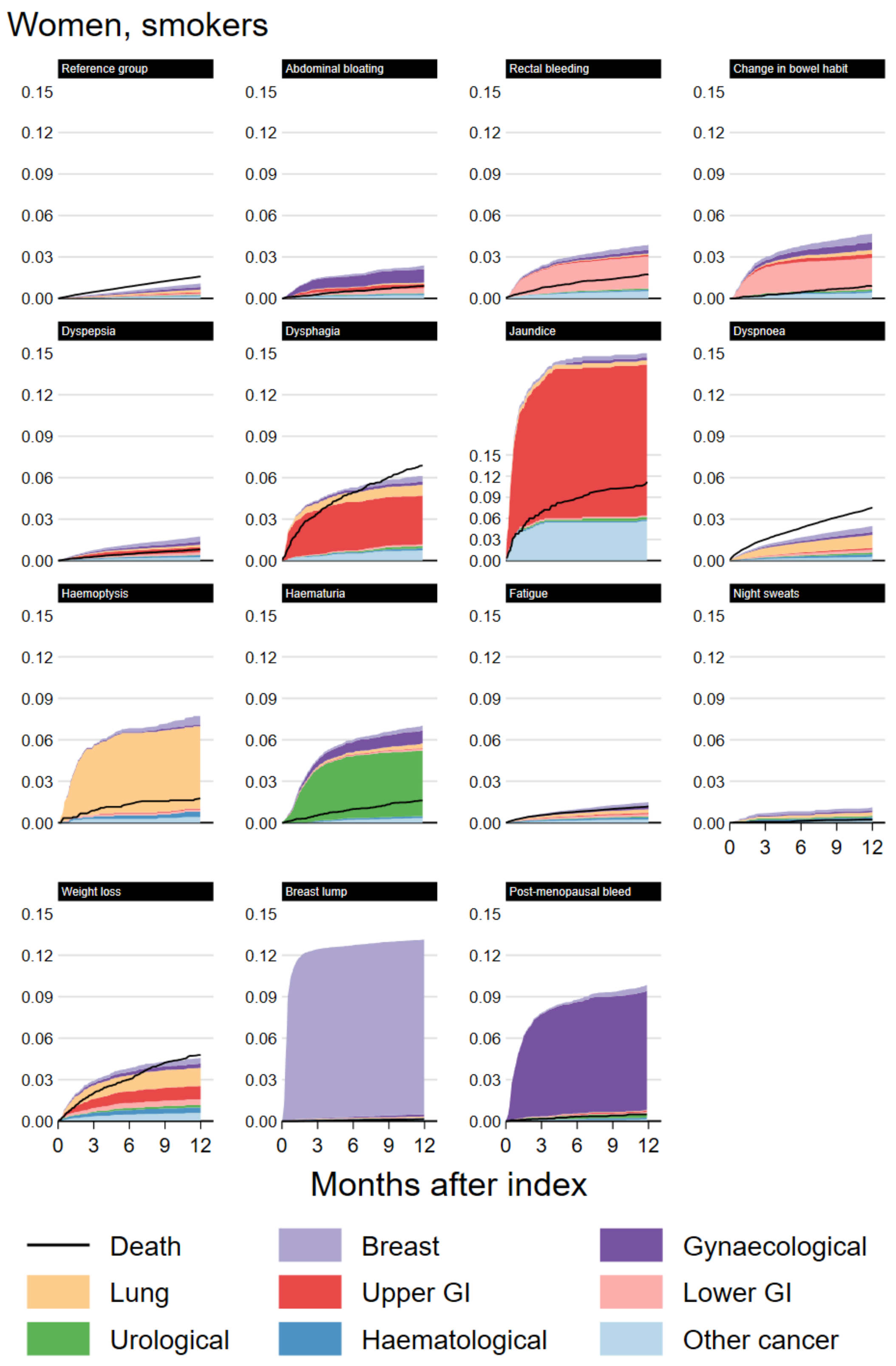

Appendix 3. Details of the outcome-specific models.

Appendix 3 Table 1. Hazard ratios for lung cancer, men.

	Hazard ratio		
**Outcome: Lung**	**Estimate**	**(95% CI)**	**p-value**
**Smoking status**			
Never smoker	(ref)		
Ever-smoker	3.99	(3.45, 4.60)	<0.001
**Symptoms**			
Reference cohort	(ref)		
Abdominal pain	0.99	(0.86, 1.14)	0.860
ln(time) * Abdominal pain	0.81	(0.78, 0.85)	<0.001
Abdominal bloating	0.73	(0.47, 1.13)	0.159
ln(time) * Abdominal bloating	0.84	(0.74, 0.95)	0.007
Rectal bleeding	0.67	(0.50, 0.90)	0.008
ln(time) * Rectal bleeding	0.95	(0.85, 1.07)	0.415
Change in bowel habit	0.88	(0.61, 1.27)	0.492
ln(time) * Change in bowel habit	0.91	(0.80, 1.03)	0.118
Dyspepsia	0.95	(0.80, 1.13)	0.585
ln(time) * Dyspepsia	0.88	(0.83, 0.93)	<0.001
Dysphagia	1.52	(1.12, 2.05)	0.007
ln(time) * Dysphagia	0.79	(0.73, 0.84)	<0.001
Jaundice	1.92	(0.85, 4.31)	0.116
ln(time) * Jaundice	0.77	(0.65, 0.90)	0.001
Dyspnoea	2.62	(2.40, 2.87)	<0.001
ln(time) * Dyspnoea	0.78	(0.75, 0.80)	<0.001
Haemoptysis	17.14	(14.81, 19.84)	<0.001
ln(time) * Haemoptysis	0.71	(0.69, 0.74)	<0.001
Haematuria	0.79	(0.61, 1.02)	0.072
ln(time) * Haematuria	0.94	(0.86, 1.04)	0.231
Fatigue	1.57	(1.36, 1.82)	<0.001
ln(time) * Fatigue	0.79	(0.76, 0.82)	<0.001
Night sweats	2.85	(1.89, 4.31)	<0.001
ln(time) * Night sweats	0.86	(0.76, 0.98)	0.018
Weight loss	4.70	(4.01, 5.50)	<0.001
ln(time) * Weight loss	0.74	(0.71, 0.77)	<0.001
Breast lump	0.93	(0.39, 2.25)	0.876
ln(time) * Breast lump	0.79	(0.66, 0.95)	0.013

Appendix 3 Table 2. Hazard ratios for upper GI cancer, men.

	Hazard ratio		
**Outcome: Upper GI**	**Estimate**	**(95% CI)**	**p-value**
**Smoking status**			
Never smoker	(ref)		
Ever-smoker	1.35	(1.23, 1.48)	<0.001
**Symptoms**			
Reference cohort	(ref)		
Abdominal pain	3.68	(3.29, 4.12)	<0.001
ln(time) * Abdominal pain	0.75	(0.72, 0.78)	<0.001
Abdominal bloating	3.57	(2.75, 4.64)	<0.001
ln(time) * Abdominal bloating	0.78	(0.73, 0.84)	<0.001
Rectal bleeding	1.26	(0.96, 1.65)	0.094
ln(time) * Rectal bleeding	0.77	(0.72, 0.81)	<0.001
Change in bowel habit	1.63	(1.15, 2.30)	0.006
ln(time) * Change in bowel habit	0.85	(0.76, 0.94)	0.002
Dyspepsia	4.50	(3.99, 5.08)	<0.001
ln(time) * Dyspepsia	0.75	(0.72, 0.78)	<0.001
Dysphagia	23.78	(21.06, 26.86)	<0.001
ln(time) * Dysphagia	0.67	(0.64, 0.69)	<0.001
Jaundice	70.89	(59.44, 84.56)	<0.001
ln(time) * Jaundice	0.67	(0.64, 0.69)	<0.001
Dyspnoea	1.19	(1.03, 1.38)	0.020
ln(time) * Dyspnoea	0.88	(0.84, 0.93)	<0.001
Haemoptysis	1.42	(0.78, 2.58)	0.247
ln(time) * Haemoptysis	0.81	(0.69, 0.94)	0.006
Haematuria	0.69	(0.48, 0.98)	0.038
ln(time) * Haematuria	0.92	(0.81, 1.05)	0.202
Fatigue	1.38	(1.14, 1.67)	<0.001
ln(time) * Fatigue	0.78	(0.74, 0.82)	<0.001
Night sweats	0.84	(0.34, 2.05)	0.700
ln(time) * Night sweats	1.97	(0.76, 5.10)	0.162
Weight loss	6.67	(5.60, 7.93)	<0.001
ln(time) * Weight loss	0.74	(0.71, 0.78)	<0.001
Breast lump	1.12	(0.42, 3.00)	0.821
ln(time) * Breast lump	0.88	(0.64, 1.22)	0.445

Appendix 3 Table 3. Hazard ratios for lower GI cancer, men.

Outcome: Lower GI	Hazard ratio Estimate	(95% CI)	p-value
**Smoking status**			
Never smoker	(ref)		
Ever-smoker	1.04	(0.96, 1.12)	0.359
**Symptoms**			
Reference cohort	(ref)		
Abdominal pain	5.02	(4.51, 5.59)	<0.001
ln(time) * Abdominal pain	0.76	(0.73, 0.78)	<0.001
Abdominal bloating	4.77	(3.78, 6.02)	<0.001
ln(time) * Abdominal bloating	0.79	(0.75, 0.84)	<0.001
Rectal bleeding	17.45	(15.69, 19.40)	<0.001
ln(time) * Rectal bleeding	0.73	(0.70, 0.75)	<0.001
Change in bowel habit	21.47	(18.98, 24.28)	<0.001
ln(time) * Change in bowel habit	0.71	(0.69, 0.74)	<0.001
Dyspepsia	1.60	(1.34, 1.91)	<0.001
ln(time) * Dyspepsia	0.91	(0.85, 0.97)	0.002
Dysphagia	1.62	(1.11, 2.35)	0.012
ln(time) * Dysphagia	0.89	(0.79, 1.00)	0.045
Jaundice	2.52	(1.04, 6.13)	0.041
ln(time) * Jaundice	0.74	(0.64, 0.85)	<0.001
Dyspnoea	1.52	(1.32, 1.74)	<0.001
ln(time) * Dyspnoea	0.95	(0.90, 1.00)	0.032
Haemoptysis	1.09	(0.54, 2.19)	0.812
ln(time) * Haemoptysis	0.90	(0.72, 1.13)	0.357
Haematuria	1.13	(0.85, 1.50)	0.408
ln(time) * Haematuria	0.93	(0.85, 1.03)	0.186
Fatigue	1.86	(1.57, 2.20)	<0.001
ln(time) * Fatigue	0.84	(0.80, 0.88)	<0.001
Night sweats	2.18	(1.20, 3.95)	0.011
ln(time) * Night sweats	0.90	(0.74, 1.10)	0.304
Weight loss	4.33	(3.52, 5.34)	<0.001
ln(time) * Weight loss	0.75	(0.72, 0.79)	<0.001
Breast lump	0.26	(0.03, 1.89)	0.182
ln(time) * Breast lump	2.09	(0.24, 18.57)	0.508

Appendix 3 Table 4. Hazard ratios for urological cancer, men.

	Hazard ratio		
**Outcome: Urological**	**Estimate**	**(95% CI)**	**p-value**
**Smoking status**			
Never smoker	(ref)		
Ever-smoker	1.36	(1.24, 1.49)	<0.001
**Symptoms**			
Reference cohort	(ref)		
Abdominal pain	2.39	(2.06, 2.78)	<0.001
ln(time) * Abdominal pain	0.81	(0.77, 0.85)	<0.001
Abdominal bloating	1.58	(1.01, 2.47)	0.046
ln(time) * Abdominal bloating	0.90	(0.76, 1.06)	0.195
Rectal bleeding	1.29	(0.95, 1.76)	0.108
ln(time) * Rectal bleeding	0.88	(0.79, 0.98)	0.019
Change in bowel habit	1.55	(1.02, 2.35)	0.041
ln(time) * Change in bowel habit	0.80	(0.71, 0.90)	<0.001
Dyspepsia	1.42	(1.15, 1.75)	0.001
ln(time) * Dyspepsia	0.96	(0.88, 1.05)	0.412
Dysphagia	1.22	(0.74, 2.01)	0.433
ln(time) * Dysphagia	1.14	(0.86, 1.50)	0.362
Jaundice	4.61	(2.06, 10.33)	<0.001
ln(time) * Jaundice	0.84	(0.67, 1.05)	0.121
Dyspnoea	1.41	(1.20, 1.66)	<0.001
ln(time) * Dyspnoea	0.89	(0.84, 0.95)	<0.001
Haemoptysis	0.89	(0.37, 2.15)	0.792
ln(time) * Haemoptysis	1.06	(0.69, 1.64)	0.787
Haematuria	45.08	(40.56, 50.10)	<0.001
ln(time) * Haematuria	0.69	(0.66, 0.72)	<0.001
Fatigue	1.37	(1.10, 1.71)	0.006
ln(time) * Fatigue	0.85	(0.79, 0.91)	<0.001
Night sweats	2.85	(1.57, 5.19)	<0.001
ln(time) * Night sweats	0.74	(0.66, 0.83)	<0.001
Weight loss	2.97	(2.23, 3.97)	<0.001
ln(time) * Weight loss	0.73	(0.68, 0.77)	<0.001
Breast lump	1.03	(0.33, 3.25)	0.959
ln(time) * Breast lump	1.63	(0.59, 4.52)	0.351

Appendix 3 Table 5. Hazard ratios for prostate cancer, men.

Outcome: Prostate	Estimate	Hazard ratio (95% CI)	p-value
**Smoking status**			
Never smoker	(ref)		
Ever-smoker	0.89	(0.82, 0.95)	<0.001
**Symptoms**			
Reference cohort	(ref)		
Abdominal pain	1.92	(1.74, 2.12)	<0.001
ln(time) * Abdominal pain	0.84	(0.82, 0.88)	<0.001
Abdominal bloating	1.88	(1.47, 2.40)	<0.001
ln(time) * Abdominal bloating	0.93	(0.85, 1.03)	0.156
Rectal bleeding	1.84	(1.56, 2.17)	<0.001
ln(time) * Rectal bleeding	0.86	(0.82, 0.91)	<0.001
Change in bowel habit	2.08	(1.68, 2.58)	<0.001
ln(time) * Change in bowel habit	0.83	(0.78, 0.89)	<0.001
Dyspepsia	1.45	(1.27, 1.65)	<0.001
ln(time) * Dyspepsia	0.94	(0.89, 0.99)	0.020
Dysphagia	1.04	(0.76, 1.43)	0.808
ln(time) * Dysphagia	0.85	(0.77, 0.94)	0.001
Jaundice	1.25	(0.52, 3.03)	0.622
ln(time) * Jaundice	0.79	(0.65, 0.96)	0.018
Dyspnoea	1.11	(1.00, 1.24)	0.043
ln(time) * Dyspnoea	0.97	(0.93, 1.02)	0.232
Haemoptysis	1.17	(0.74, 1.87)	0.499
ln(time) * Haemoptysis	1.16	(0.89, 1.51)	0.274
Haematuria	5.27	(4.76, 5.84)	<0.001
ln(time) * Haematuria	0.77	(0.75, 0.80)	<0.001
Fatigue	1.72	(1.52, 1.94)	<0.001
ln(time) * Fatigue	0.83	(0.80, 0.87)	<0.001
Night sweats	2.15	(1.41, 3.28)	<0.001
ln(time) * Night sweats	0.91	(0.78, 1.06)	0.229
Weight loss	2.79	(2.34, 3.32)	<0.001
ln(time) * Weight loss	0.76	(0.73, 0.80)	<0.001
Breast lump	1.17	(0.58, 2.34)	0.665
ln(time) * Breast lump	0.99	(0.72, 1.38)	0.965

Append 3 Table 6. Hazard ratios for haematological cancer, men.

	Hazard ratio		
**Outcome: Haematological**	**Estimate**	**(95% CI)**	**p-value**
**Smoking status**			
Never smoker	(ref)		
Ever-smoker	0.98	(0.88, 1.10)	0.742
**Symptoms**			
Reference cohort	(ref)		
Abdominal pain	2.35	(2.03, 2.72)	<0.001
ln(time) * Abdominal pain	0.80	(0.77, 0.85)	<0.001
Abdominal bloating	2.08	(1.42, 3.07)	<0.001
ln(time) * Abdominal bloating	0.75	(0.69, 0.82)	<0.001
Rectal bleeding	1.26	(0.93, 1.71)	0.142
ln(time) * Rectal bleeding	0.81	(0.75, 0.88)	<0.001
Change in bowel habit	1.69	(1.14, 2.50)	0.009
ln(time) * Change in bowel habit	0.83	(0.75, 0.93)	0.001
Dyspepsia	1.47	(1.20, 1.81)	<0.001
ln(time) * Dyspepsia	0.89	(0.83, 0.95)	0.001
Dysphagia	1.79	(1.20, 2.68)	0.005
ln(time) * Dysphagia	0.76	(0.70, 0.83)	<0.001
Jaundice	6.06	(3.11, 11.81)	<0.001
ln(time) * Jaundice	0.76	(0.66, 0.87)	<0.001
Dyspnoea	1.70	(1.46, 1.98)	<0.001
ln(time) * Dyspnoea	0.81	(0.77, 0.85)	<0.001
Haemoptysis	2.77	(1.69, 4.57)	<0.001
ln(time) * Haemoptysis	0.79	(0.70, 0.88)	<0.001
Haematuria	1.33	(0.98, 1.80)	0.066
ln(time) * Haematuria	0.95	(0.84, 1.07)	0.372
Fatigue	2.34	(1.97, 2.79)	<0.001
ln(time) * Fatigue	0.77	(0.73, 0.81)	<0.001
Night sweats	4.52	(2.82, 7.23)	<0.001
ln(time) * Night sweats	0.71	(0.66, 0.78)	<0.001
Weight loss	4.41	(3.47, 5.59)	<0.001
ln(time) * Weight loss	0.75	(0.71, 0.79)	<0.001
Breast lump	2.14	(0.95, 4.78)	0.065
ln(time) * Breast lump	0.97	(0.69, 1.36)	0.848

Appendix 3 Table 7. Hazard ratios for other cancers, men.

	Hazard ratio		
**Outcome: Other cancer**	**Estimate**	**(95% CI)**	**p-value**
**Smoking status**			
Never smoker	(ref)		
Ever-smoker	1.20	(1.09, 1.32)	<0.001
**Symptoms**			
Reference cohort	(ref)		
Abdominal pain	1.69	(1.49, 1.90)	<0.001
ln(time) * Abdominal pain	0.82	(0.79, 0.85)	<0.001
Abdominal bloating	1.40	(0.98, 1.99)	0.063
ln(time) * Abdominal bloating	0.85	(0.76, 0.95)	0.004
Rectal bleeding	1.54	(1.26, 1.90)	<0.001
ln(time) * Rectal bleeding	0.87	(0.81, 0.93)	<0.001
Change in bowel habit	1.72	(1.28, 2.32)	<0.001
ln(time) * Change in bowel habit	0.78	(0.72, 0.83)	<0.001
Dyspepsia	1.40	(1.20, 1.64)	<0.001
ln(time) * Dyspepsia	0.85	(0.81, 0.89)	<0.001
Dysphagia	3.06	(2.41, 3.89)	<0.001
ln(time) * Dysphagia	0.77	(0.73, 0.82)	<0.001
Jaundice	19.84	(15.05, 26.16)	<0.001
ln(time) * Jaundice	0.69	(0.66, 0.72)	<0.001
Dyspnoea	1.32	(1.17, 1.50)	<0.001
ln(time) * Dyspnoea	0.86	(0.82, 0.90)	<0.001
Haemoptysis	2.15	(1.41, 3.28)	<0.001
ln(time) * Haemoptysis	0.80	(0.71, 0.89)	<0.001
Haematuria	1.27	(1.00, 1.61)	0.049
ln(time) * Haematuria	0.91	(0.84, 0.99)	0.032
Fatigue	1.65	(1.42, 1.92)	<0.001
ln(time) * Fatigue	0.81	(0.77, 0.85)	<0.001
Night sweats	1.77	(1.03, 3.07)	0.040
ln(time) * Night sweats	0.88	(0.73, 1.04)	0.141
Weight loss	3.17	(2.57, 3.90)	<0.001
ln(time) * Weight loss	0.76	(0.72, 0.79)	<0.001
Breast lump	7.07	(5.04, 9.91)	<0.001
ln(time) * Breast lump	0.67	(0.64, 0.70)	<0.001

Appendix 3 Table 8. Hazard ratios for death (no cancer), men.

Outcome: Death	Hazard ratio Estimate	(95% CI)	p-value
**Smoking status**			
Never smoker	(ref)		
Ever-smoker	1.09	(1.05, 1.13)	<0.001
**Symptoms**			
Reference cohort	(ref)		
Abdominal pain	0.70	(0.66, 0.75)	<0.001
ln(time) * Abdominal pain	0.90	(0.88, 0.92)	<0.001
Abdominal bloating	0.67	(0.57, 0.79)	<0.001
ln(time) * Abdominal bloating	0.97	(0.91, 1.02)	0.243
Rectal bleeding	0.76	(0.69, 0.84)	<0.001
ln(time) * Rectal bleeding	0.92	(0.90, 0.95)	<0.001
Change in bowel habit	0.42	(0.34, 0.51)	<0.001
ln(time) * Change in bowel habit	1.06	(0.97, 1.15)	0.200
Dyspepsia	0.58	(0.53, 0.63)	<0.001
ln(time) * Dyspepsia	0.96	(0.93, 0.98)	0.002
Dysphagia	1.99	(1.82, 2.17)	<0.001
ln(time) * Dysphagia	0.90	(0.88, 0.92)	<0.001
Jaundice	4.70	(3.92, 5.63)	<0.001
ln(time) * Jaundice	0.84	(0.81, 0.87)	<0.001
Dyspnoea	1.40	(1.34, 1.45)	<0.001
ln(time) * Dyspnoea	0.93	(0.92, 0.95)	<0.001
Haemoptysis	1.33	(1.11, 1.59)	0.002
ln(time) * Haemoptysis	0.95	(0.90, 1.01)	0.087
Haematuria	0.79	(0.72, 0.86)	<0.001
ln(time) * Haematuria	0.98	(0.95, 1.01)	0.169
Fatigue	0.95	(0.90, 1.02)	0.147
ln(time) * Fatigue	0.93	(0.92, 0.95)	<0.001
Night sweats	0.50	(0.35, 0.73)	<0.001
ln(time) * Night sweats	1.27	(1.01, 1.59)	0.039
Weight loss	1.77	(1.63, 1.92)	<0.001
ln(time) * Weight loss	0.97	(0.94, 1.00)	0.044
Breast lump	0.50	(0.32, 0.77)	0.002
ln(time) * Breast lump	1.19	(0.93, 1.51)	0.162

Appendix 3 Table 9. Hazard ratios for breast cancer, women.

Outcome: Breast	Hazard ratio Estimate	(95% CI)	p-value
**Smoking status**			
Never smoker	(ref)		
Ever-smoker	1.05	(1.01, 1.10)	0.020
**Symptoms**			
Reference cohort	(ref)		
Abdominal pain	1.14	(1.03, 1.26)	0.009
ln(time) * Abdominal pain	1.05	(1.00, 1.11)	0.052
Abdominal bloating	1.22	(0.96, 1.56)	0.106
ln(time) * Abdominal bloating	1.07	(0.93, 1.22)	0.345
Rectal bleeding	1.12	(0.91, 1.37)	0.275
ln(time) * Rectal bleeding	0.96	(0.88, 1.05)	0.352
Change in bowel habit	1.32	(1.02, 1.71)	0.033
ln(time) * Change in bowel habit	0.88	(0.80, 0.97)	0.012
Dyspepsia	1.24	(1.10, 1.41)	<0.001
ln(time) * Dyspepsia	0.97	(0.91, 1.03)	0.270
Dysphagia	0.86	(0.61, 1.20)	0.372
ln(time) * Dysphagia	0.91	(0.80, 1.03)	0.149
Jaundice	2.24	(1.06, 4.72)	0.035
ln(time) * Jaundice	0.86	(0.68, 1.08)	0.196
Dyspnoea	1.06	(0.95, 1.18)	0.266
ln(time) * Dyspnoea	0.92	(0.88, 0.97)	<0.001
Haemoptysis	1.79	(1.12, 2.86)	0.015
ln(time) * Haemoptysis	1.25	(0.91, 1.72)	0.162
Haematuria	1.37	(1.06, 1.78)	0.017
ln(time) * Haematuria	0.97	(0.86, 1.09)	0.585
Fatigue	1.21	(1.08, 1.35)	<0.001
ln(time) * Fatigue	0.95	(0.90, 1.00)	0.034
Night sweats	1.35	(0.85, 2.12)	0.200
ln(time) * Night sweats	0.89	(0.75, 1.05)	0.165
Weight loss	1.07	(0.82, 1.39)	0.608
ln(time) * Weight loss	0.84	(0.77, 0.92)	<0.001
Breast lump	64.98	(61.10, 69.10)	<0.001
ln(time) * Breast lump	0.64	(0.62, 0.65)	<0.001
Post-menopausal bleed	1.22	(0.90, 1.65)	0.191
ln(time) * Post-menopausal bleed	1.09	(0.92, 1.29)	0.300

Appendix 3 Table 10. Hazard ratios for gynaecological cancer, women.

	Hazard ratio		
**Outcome: Gynaecological**	**Estimate**	**(95% CI)**	**p-value**
**Smoking status**			
Never smoker	(ref)		
Ever-smoker	1.07	(1.01, 1.15)	0.032
**Symptoms**			
Reference cohort	(ref)		
Abdominal pain	2.49	(2.27, 2.73)	<0.001
ln(time) * Abdominal pain	0.80	(0.77, 0.82)	<0.001
Abdominal bloating	4.75	(4.03, 5.60)	<0.001
ln(time) * Abdominal bloating	0.72	(0.70, 0.75)	<0.001
Rectal bleeding	1.25	(0.97, 1.60)	0.086
ln(time) * Rectal bleeding	0.86	(0.79, 0.94)	<0.001
Change in bowel habit	2.58	(1.98, 3.36)	<0.001
ln(time) * Change in bowel habit	0.78	(0.73, 0.84)	<0.001
Dyspepsia	1.15	(0.97, 1.35)	0.109
ln(time) * Dyspepsia	0.93	(0.87, 1.00)	0.042
Dysphagia	0.76	(0.45, 1.30)	0.318
ln(time) * Dysphagia	0.85	(0.71, 1.00)	0.056
Jaundice	1.34	(0.34, 5.38)	0.676
ln(time) * Jaundice	1.00	(0.57, 1.78)	0.989
Dyspnoea	1.06	(0.92, 1.24)	0.414
ln(time) * Dyspnoea	0.87	(0.82, 0.92)	<0.001
Haemoptysis	0.74	(0.28, 1.98)	0.552
ln(time) * Haemoptysis	0.89	(0.63, 1.27)	0.528
Haematuria	4.57	(3.72, 5.61)	<0.001
ln(time) * Haematuria	0.81	(0.76, 0.86)	<0.001
Fatigue	1.27	(1.12, 1.45)	<0.001
ln(time) * Fatigue	0.95	(0.90, 1.01)	0.104
Night sweats	0.74	(0.37, 1.49)	0.402
ln(time) * Night sweats	0.91	(0.70, 1.19)	0.500
Weight loss	1.74	(1.30, 2.33)	<0.001
ln(time) * Weight loss	0.82	(0.75, 0.89)	<0.001
Breast lump	1.19	(0.95, 1.49)	0.131
ln(time) * Breast lump	1.08	(0.96, 1.21)	0.216
Post-menopausal bleed	42.73	(38.56, 47.34)	<0.001
ln(time) * Post-menopausal bleed	0.68	(0.66, 0.70)	<0.001

Appendix 3 Table 11. Hazard ratios for lung cancer, women.

Outcome: Lung	Hazard ratio Estimate	(95% CI)	p-value
**Smoking status**			
Never smoker	(ref)		
Ever-smoker	4.85	(4.22, 5.56)	<0.001
**Symptoms**			
Reference cohort	(ref)		
Abdominal pain	1.00	(0.86, 1.17)	0.995
ln(time) * Abdominal pain	0.90	(0.85, 0.95)	<0.001
Abdominal bloating	0.66	(0.41, 1.05)	0.081
ln(time) * Abdominal bloating	0.87	(0.75, 1.01)	0.062
Rectal bleeding	0.59	(0.40, 0.87)	0.008
ln(time) * Rectal bleeding	1.04	(0.87, 1.26)	0.644
Change in bowel habit	0.94	(0.62, 1.41)	0.762
ln(time) * Change in bowel habit	0.95	(0.81, 1.11)	0.516
Dyspepsia	0.69	(0.55, 0.86)	<0.001
ln(time) * Dyspepsia	0.92	(0.84, 0.99)	0.035
Dysphagia	2.04	(1.49, 2.79)	<0.001
ln(time) * Dysphagia	0.79	(0.73, 0.85)	<0.001
Jaundice	0.81	(0.21, 3.10)	0.757
ln(time) * Jaundice	0.55	(0.46, 0.66)	<0.001
Dyspnoea	2.57	(2.30, 2.86)	<0.001
ln(time) * Dyspnoea	0.81	(0.78, 0.84)	<0.001
Haemoptysis	17.20	(13.94, 21.22)	<0.001
ln(time) * Haemoptysis	0.73	(0.70, 0.77)	<0.001
Haematuria	1.17	(0.80, 1.70)	0.424
ln(time) * Haematuria	1.05	(0.88, 1.24)	0.615
Fatigue	1.33	(1.14, 1.56)	<0.001
ln(time) * Fatigue	0.86	(0.81, 0.90)	<0.001
Night sweats	1.57	(0.86, 2.85)	0.140
ln(time) * Night sweats	0.91	(0.73, 1.12)	0.358
Weight loss	3.70	(3.03, 4.52)	<0.001
ln(time) * Weight loss	0.78	(0.74, 0.82)	<0.001
Breast lump	0.82	(0.52, 1.28)	0.384
ln(time) * Breast lump	0.88	(0.77, 1.02)	0.086
Post-menopausal bleed	0.16	(0.05, 0.51)	0.002
ln(time) * Post-menopausal bleed	0.88	(0.62, 1.27)	0.506

Appendix 3 Table 12. Hazard ratios for upper GI cancer, women.

Outcome: Upper GI	Hazard ratio Estimate	(95% CI)	p-value
**Smoking status**			
Never smoker	(ref)		
Ever-smoker	1.20	(1.10, 1.32)	<0.001
**Symptoms**			
Reference cohort	(ref)		
Abdominal pain	3.37	(2.95, 3.85)	<0.001
ln(time) * Abdominal pain	0.75	(0.72, 0.79)	<0.001
Abdominal bloating	2.88	(2.14, 3.88)	<0.001
ln(time) * Abdominal bloating	0.78	(0.72, 0.84)	<0.001
Rectal bleeding	1.10	(0.76, 1.58)	0.624
ln(time) * Rectal bleeding	0.76	(0.69, 0.82)	<0.001
Change in bowel habit	1.87	(1.29, 2.73)	0.001
ln(time) * Change in bowel habit	0.79	(0.71, 0.88)	<0.001
Dyspepsia	3.28	(2.81, 3.84)	<0.001
ln(time) * Dyspepsia	0.77	(0.73, 0.81)	<0.001
Dysphagia	16.36	(13.95, 19.18)	<0.001
ln(time) * Dysphagia	0.66	(0.63, 0.69)	<0.001
Jaundice	122.63	(102.30, 147.01)	<0.001
ln(time) * Jaundice	0.66	(0.63, 0.69)	<0.001
Dyspnoea	0.88	(0.73, 1.08)	0.218
ln(time) * Dyspnoea	0.90	(0.84, 0.97)	0.009
Haemoptysis	0.61	(0.15, 2.45)	0.487
ln(time) * Haemoptysis	0.88	(0.56, 1.40)	0.598
Haematuria	0.49	(0.23, 1.03)	0.058
ln(time) * Haematuria	0.78	(0.65, 0.93)	0.007
Fatigue	1.04	(0.84, 1.29)	0.689
ln(time) * Fatigue	0.81	(0.76, 0.87)	<0.001
Night sweats	0.50	(0.13, 2.01)	0.331
ln(time) * Night sweats	0.77	(0.56, 1.06)	0.104
Weight loss	5.57	(4.52, 6.87)	<0.001
ln(time) * Weight loss	0.74	(0.70, 0.78)	<0.001
Breast lump	0.78	(0.44, 1.38)	0.389
ln(time) * Breast lump	0.98	(0.76, 1.25)	0.853
Post-menopausal bleed	1.06	(0.58, 1.92)	0.856
ln(time) * Post-menopausal bleed	1.01	(0.77, 1.33)	0.938

Appendix 3 Table 13. Hazard ratios for lower GI cancer, women.

	Hazard ratio		
**Outcome: Lower GI**	**Estimate**	**(95% CI)**	**p-value**
**Smoking status**			
Never smoker	(ref)		
Ever-smoker	0.96	(0.89, 1.04)	0.339
**Symptoms**			
Reference cohort	(ref)		
Abdominal pain	4.35	(3.89, 4.86)	<0.001
ln(time) * Abdominal pain	0.71	(0.68, 0.74)	<0.001
Abdominal bloating	3.04	(2.34, 3.95)	<0.001
ln(time) * Abdominal bloating	0.72	(0.67, 0.77)	<0.001
Rectal bleeding	14.74	(13.08, 16.62)	<0.001
ln(time) * Rectal bleeding	0.68	(0.65, 0.72)	<0.001
Change in bowel habit	12.18	(10.45, 14.21)	<0.001
ln(time) * Change in bowel habit	0.66	(0.63, 0.69)	<0.001
Dyspepsia	1.28	(1.05, 1.56)	0.013
ln(time) * Dyspepsia	0.84	(0.78, 0.90)	<0.001
Dysphagia	1.35	(0.89, 2.05)	0.157
ln(time) * Dysphagia	0.85	(0.73, 0.98)	0.029
Jaundice	2.05	(0.65, 6.45)	0.222
ln(time) * Jaundice	0.70	(0.57, 0.86)	<0.001
Dyspnoea	1.26	(1.07, 1.47)	0.004
ln(time) * Dyspnoea	0.88	(0.82, 0.94)	<0.001
Haemoptysis	1.01	(0.38, 2.71)	0.977
ln(time) * Haemoptysis	0.74	(0.58, 0.94)	0.015
Haematuria	0.97	(0.60, 1.57)	0.892
ln(time) * Haematuria	0.84	(0.71, 0.99)	0.041
Fatigue	1.67	(1.43, 1.96)	<0.001
ln(time) * Fatigue	0.78	(0.74, 0.83)	<0.001
Night sweats	1.48	(0.73, 2.99)	0.272
ln(time) * Night sweats	1.25	(0.76, 2.08)	0.380
Weight loss	2.82	(2.19, 3.64)	<0.001
ln(time) * Weight loss	0.71	(0.67, 0.76)	<0.001
Breast lump	0.54	(0.30, 0.99)	0.046
ln(time) * Breast lump	0.95	(0.73, 1.25)	0.729
Post-menopausal bleed	1.07	(0.63, 1.81)	0.814
ln(time) * Post-menopausal bleed	0.87	(0.71, 1.08)	0.204

Appendix 3 Table 14. Hazard ratios for urological cancer, women.

Outcome: Urological	Hazard ratio Estimate	(95% CI)	p-value
**Smoking status**			
Never smoker	(ref)		
Ever-smoker	1.25	(1.10, 1.42)	<0.001
**Symptoms**			
Reference cohort	(ref)		
Abdominal pain	2.23	(1.80, 2.75)	<0.001
ln(time) * Abdominal pain	0.82	(0.77, 0.88)	<0.001
Abdominal bloating	1.56	(0.89, 2.73)	0.119
ln(time) * Abdominal bloating	0.95	(0.76, 1.19)	0.674
Rectal bleeding	1.77	(1.17, 2.68)	0.007
ln(time) * Rectal bleeding	0.97	(0.82, 1.14)	0.710
Change in bowel habit	1.71	(0.98, 3.00)	0.060
ln(time) * Change in bowel habit	0.89	(0.74, 1.08)	0.240
Dyspepsia	0.64	(0.42, 0.98)	0.040
ln(time) * Dyspepsia	0.86	(0.76, 0.98)	0.029
Dysphagia	1.92	(1.09, 3.36)	0.023
ln(time) * Dysphagia	0.96	(0.77, 1.19)	0.700
Jaundice	5.11	(1.62, 16.14)	0.005
ln(time) * Jaundice	0.73	(0.60, 0.89)	0.002
Dyspnoea	1.58	(1.26, 1.99)	<0.001
ln(time) * Dyspnoea	1.03	(0.93, 1.14)	0.527
Haemoptysis	0.00	(0.00, 0.00)	0.999
ln(time) * Haemoptysis	2.54	(0.00, 0.00)	1.000
Haematuria	57.06	(48.37, 67.29)	<0.001
ln(time) * Haematuria	0.72	(0.68, 0.77)	<0.001
Fatigue	1.32	(1.01, 1.74)	0.046
ln(time) * Fatigue	0.95	(0.86, 1.06)	0.391
Night sweats	2.04	(0.76, 5.49)	0.158
ln(time) * Night sweats	0.76	(0.64, 0.90)	0.002
Weight loss	2.26	(1.45, 3.50)	<0.001
ln(time) * Weight loss	0.77	(0.69, 0.85)	<0.001
Breast lump	1.01	(0.50, 2.06)	0.970
ln(time) * Breast lump	1.15	(0.79, 1.69)	0.467
Post-menopausal bleed	4.09	(2.61, 6.41)	<0.001
ln(time) * Post-menopausal bleed	0.76	(0.68, 0.84)	<0.001

Appendix 3 Table 15. Hazard ratios for haematological cancer, women.

	Hazard ratio		
**Outcome: Haematological**	**Estimate**	**(95% CI)**	**p-value**
**Smoking status**			
Never smoker	(ref)		
Ever-smoker	0.98	(0.88, 1.11)	0.794
**Symptoms**			
Reference cohort	(ref)		
Abdominal pain	2.16	(1.82, 2.56)	<0.001
ln(time) * Abdominal pain	0.82	(0.78, 0.88)	<0.001
Abdominal bloating	1.39	(0.87, 2.24)	0.169
ln(time) * Abdominal bloating	0.84	(0.73, 0.98)	0.026
Rectal bleeding	1.51	(1.05, 2.17)	0.026
ln(time) * Rectal bleeding	0.83	(0.74, 0.93)	0.001
Change in bowel habit	1.14	(0.65, 1.98)	0.648
ln(time) * Change in bowel habit	0.92	(0.75, 1.14)	0.460
Dyspepsia	1.64	(1.31, 2.06)	<0.001
ln(time) * Dyspepsia	0.88	(0.81, 0.95)	0.002
Dysphagia	1.82	(1.13, 2.93)	0.013
ln(time) * Dysphagia	1.05	(0.83, 1.32)	0.708
Jaundice	0.45	(0.05, 4.23)	0.487
ln(time) * Jaundice	0.51	(0.40, 0.65)	<0.001
Dyspnoea	1.83	(1.52, 2.19)	<0.001
ln(time) * Dyspnoea	0.82	(0.77, 0.87)	<0.001
Haemoptysis	3.38	(1.68, 6.81)	<0.001
ln(time) * Haemoptysis	0.78	(0.66, 0.93)	0.005
Haematuria	1.34	(0.78, 2.28)	0.287
ln(time) * Haematuria	0.83	(0.71, 0.96)	0.013
Fatigue	2.35	(1.96, 2.82)	<0.001
ln(time) * Fatigue	0.79	(0.74, 0.84)	<0.001
Night sweats	4.05	(2.33, 7.05)	<0.001
ln(time) * Night sweats	0.77	(0.67, 0.89)	<0.001
Weight loss	4.04	(3.04, 5.37)	<0.001
ln(time) * Weight loss	0.76	(0.71, 0.82)	<0.001
Breast lump	2.56	(1.83, 3.60)	<0.001
ln(time) * Breast lump	0.73	(0.68, 0.79)	<0.001
Post-menopausal bleed	1.33	(0.73, 2.43)	0.351
ln(time) * Post-menopausal bleed	0.94	(0.74, 1.19)	0.593

Appendix 3 Table 16. Hazard ratios for other cancers, women.

Outcome: Other cancer	Hazard ratio Estimate	(95% CI)	p-value
**Smoking status**			
Never smoker	(ref)		
Ever-smoker	1.06	(0.98, 1.15)	0.149
**Symptoms**			
Reference cohort	(ref)		
Abdominal pain	1.92	(1.72, 2.15)	<0.001
ln(time) * Abdominal pain	0.79	(0.76, 0.83)	<0.001
Abdominal bloating	1.72	(1.31, 2.27)	<0.001
ln(time) * Abdominal bloating	0.82	(0.75, 0.90)	<0.001
Rectal bleeding	2.27	(1.87, 2.76)	<0.001
ln(time) * Rectal bleeding	0.77	(0.73, 0.82)	<0.001
Change in bowel habit	2.16	(1.65, 2.83)	<0.001
ln(time) * Change in bowel habit	0.81	(0.75, 0.88)	<0.001
Dyspepsia	1.27	(1.08, 1.50)	0.004
ln(time) * Dyspepsia	0.84	(0.80, 0.90)	<0.001
Dysphagia	2.23	(1.69, 2.94)	<0.001
ln(time) * Dysphagia	0.78	(0.72, 0.84)	<0.001
Jaundice	19.08	(13.98, 26.04)	<0.001
ln(time) * Jaundice	0.66	(0.62, 0.70)	<0.001
Dyspnoea	1.10	(0.96, 1.27)	0.179
ln(time) * Dyspnoea	0.86	(0.82, 0.91)	<0.001
Haemoptysis	1.62	(0.84, 3.13)	0.150
ln(time) * Haemoptysis	0.78	(0.66, 0.94)	0.008
Haematuria	1.53	(1.10, 2.12)	0.011
ln(time) * Haematuria	0.97	(0.84, 1.12)	0.650
Fatigue	1.52	(1.33, 1.74)	<0.001
ln(time) * Fatigue	0.83	(0.79, 0.87)	<0.001
Night sweats	0.96	(0.48, 1.92)	0.898
ln(time) * Night sweats	1.01	(0.72, 1.42)	0.943
Weight loss	2.27	(1.79, 2.88)	<0.001
ln(time) * Weight loss	0.78	(0.73, 0.83)	<0.001
Breast lump	1.24	(0.93, 1.66)	0.149
ln(time) * Breast lump	0.92	(0.82, 1.03)	0.139
Post-menopausal bleed	1.08	(0.70, 1.66)	0.733
ln(time) * Post-menopausal bleed	0.80	(0.71, 0.91)	<0.001

Appendix 3 Table 17. Hazard ratios for death (no cancer), women.

	Hazard ratio		
**Outcome: Death**	**Estimate**	**(95% CI)**	**p-value**
**Smoking status**			
Never smoker	(ref)		
Ever-smoker	0.97	(0.94, 1.00)	0.025
**Symptoms**			
Reference cohort	(ref)		
Abdominal pain	0.73	(0.69, 0.77)	<0.001
ln(time) * Abdominal pain	0.89	(0.88, 0.91)	<0.001
Abdominal bloating	0.72	(0.62, 0.82)	<0.001
ln(time) * Abdominal bloating	0.94	(0.89, 0.98)	0.010
Rectal bleeding	0.93	(0.85, 1.01)	0.091
ln(time) * Rectal bleeding	0.92	(0.89, 0.95)	<0.001
Change in bowel habit	0.43	(0.36, 0.52)	<0.001
ln(time) * Change in bowel habit	1.04	(0.96, 1.13)	0.361
Dyspepsia	0.52	(0.48, 0.57)	<0.001
ln(time) * Dyspepsia	0.97	(0.94, 1.00)	0.047
Dysphagia	2.07	(1.92, 2.22)	<0.001
ln(time) * Dysphagia	0.88	(0.86, 0.90)	<0.001
Jaundice	3.49	(2.88, 4.23)	<0.001
ln(time) * Jaundice	0.86	(0.82, 0.90)	<0.001
Dyspnoea	1.28	(1.24, 1.33)	<0.001
ln(time) * Dyspnoea	0.91	(0.90, 0.92)	<0.001
Haemoptysis	0.97	(0.75, 1.27)	0.852
ln(time) * Haemoptysis	0.87	(0.81, 0.94)	<0.001
Haematuria	0.93	(0.82, 1.05)	0.263
ln(time) * Haematuria	1.01	(0.96, 1.06)	0.770
Fatigue	0.84	(0.79, 0.88)	<0.001
ln(time) * Fatigue	0.89	(0.87, 0.90)	<0.001
Night sweats	0.34	(0.21, 0.54)	<0.001
ln(time) * Night sweats	1.03	(0.83, 1.26)	0.806
Weight loss	1.65	(1.53, 1.77)	<0.001
ln(time) * Weight loss	0.94	(0.91, 0.96)	<0.001
Breast lump	0.55	(0.45, 0.66)	<0.001
ln(time) * Breast lump	1.07	(0.99, 1.17)	0.099
Post-menopausal bleed	0.57	(0.46, 0.72)	<0.001
ln(time) * Post-menopausal bleed	1.04	(0.94, 1.16)	0.414

Appendix 4. Figures showing simulated outcomes at 3 and 6 months after index symptom by age, sex and smoking status.

Appendix 4 Figure 1. Cumulative incidence at 3 months, male non-smokers

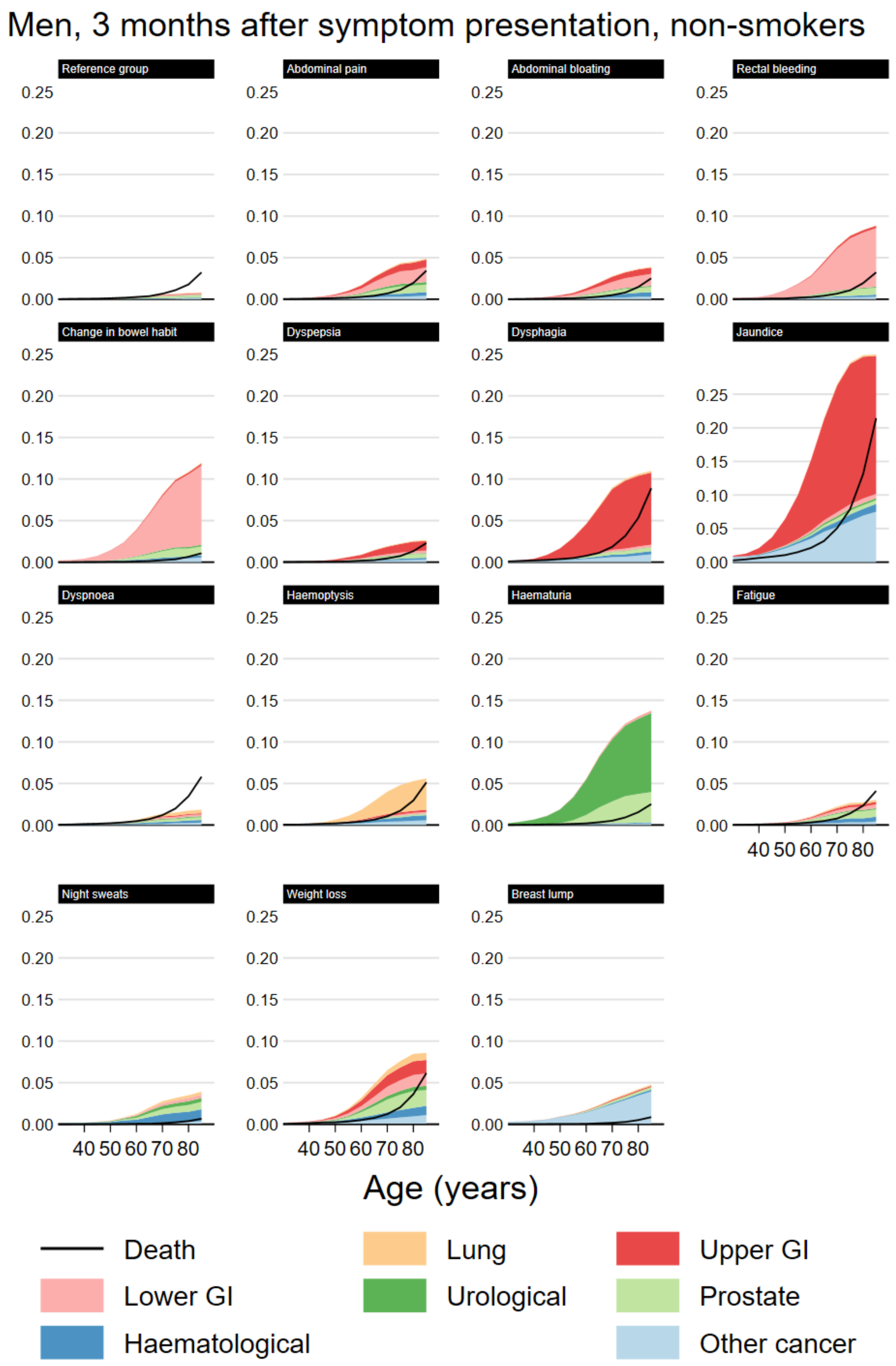

Appendix 4 Figure 2. Cumulative incidence at 3 months, male smokers

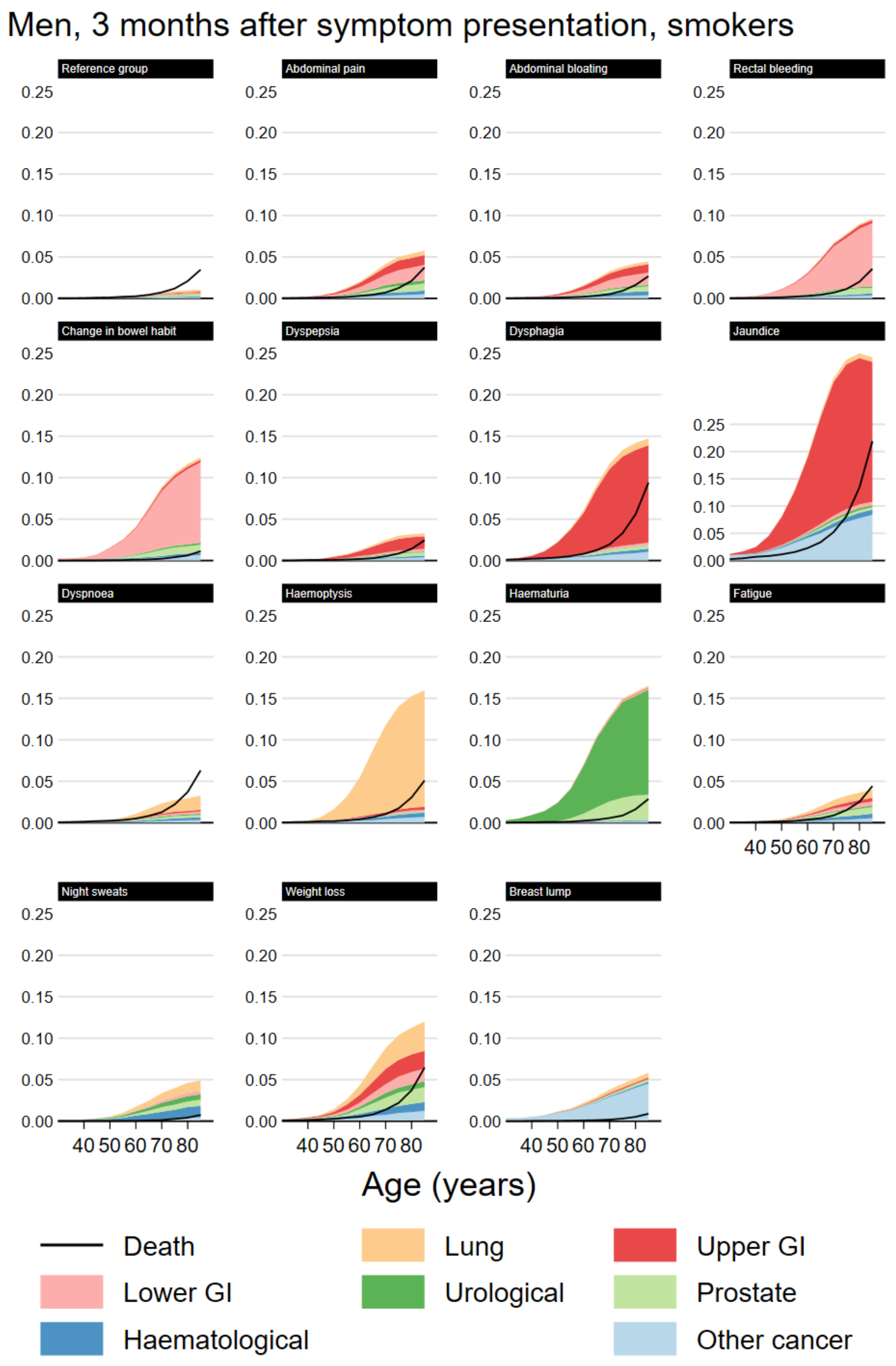

Appendix 4 Figure 3. Cumulative incidence at 3 months, female non-smokers

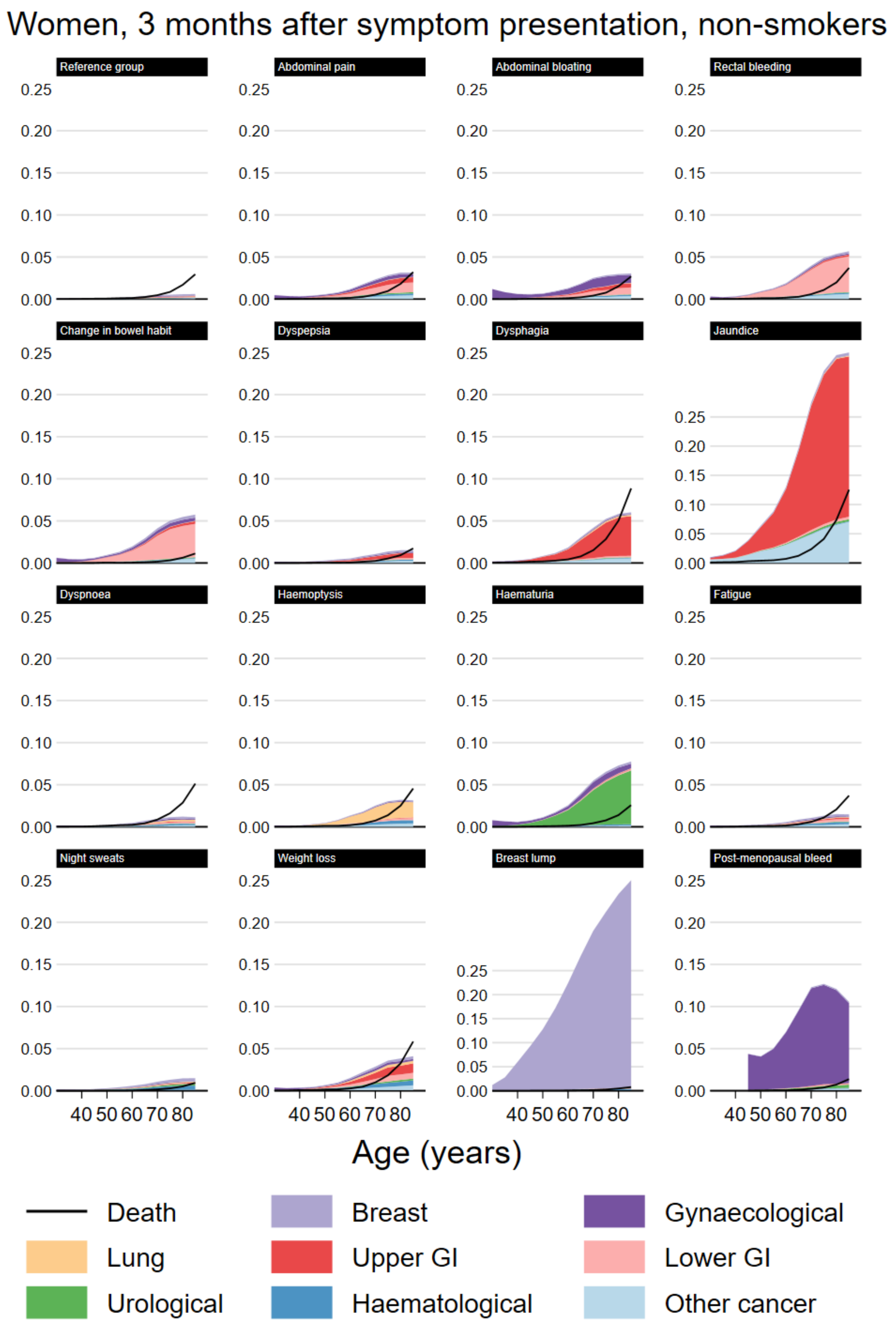

Appendix 4 Figure 4. Cumulative incidence at 3 months, female smokers

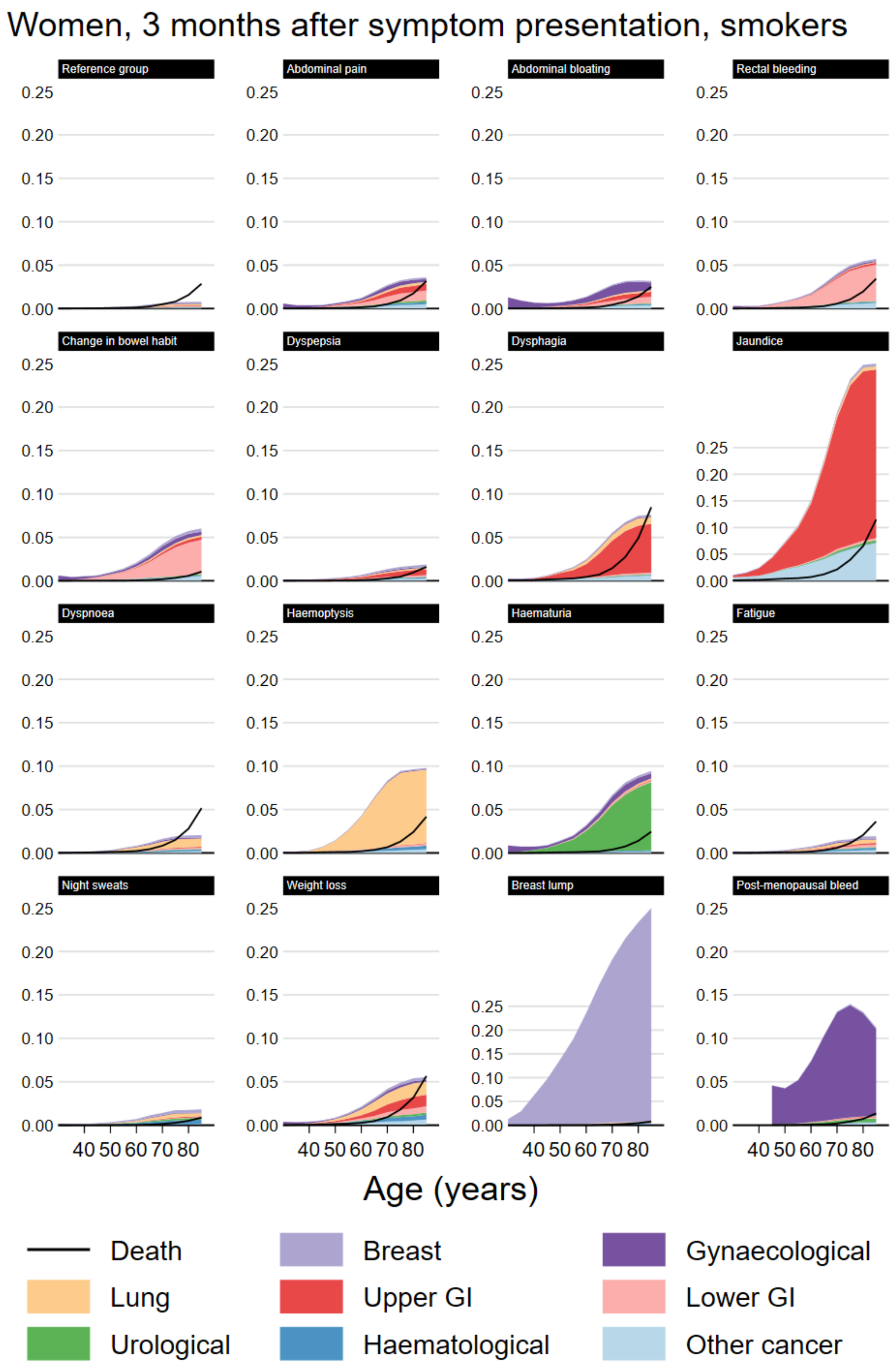

Appendix 4 Figure 5. Cumulative incidence at 6 months, male non-smokers

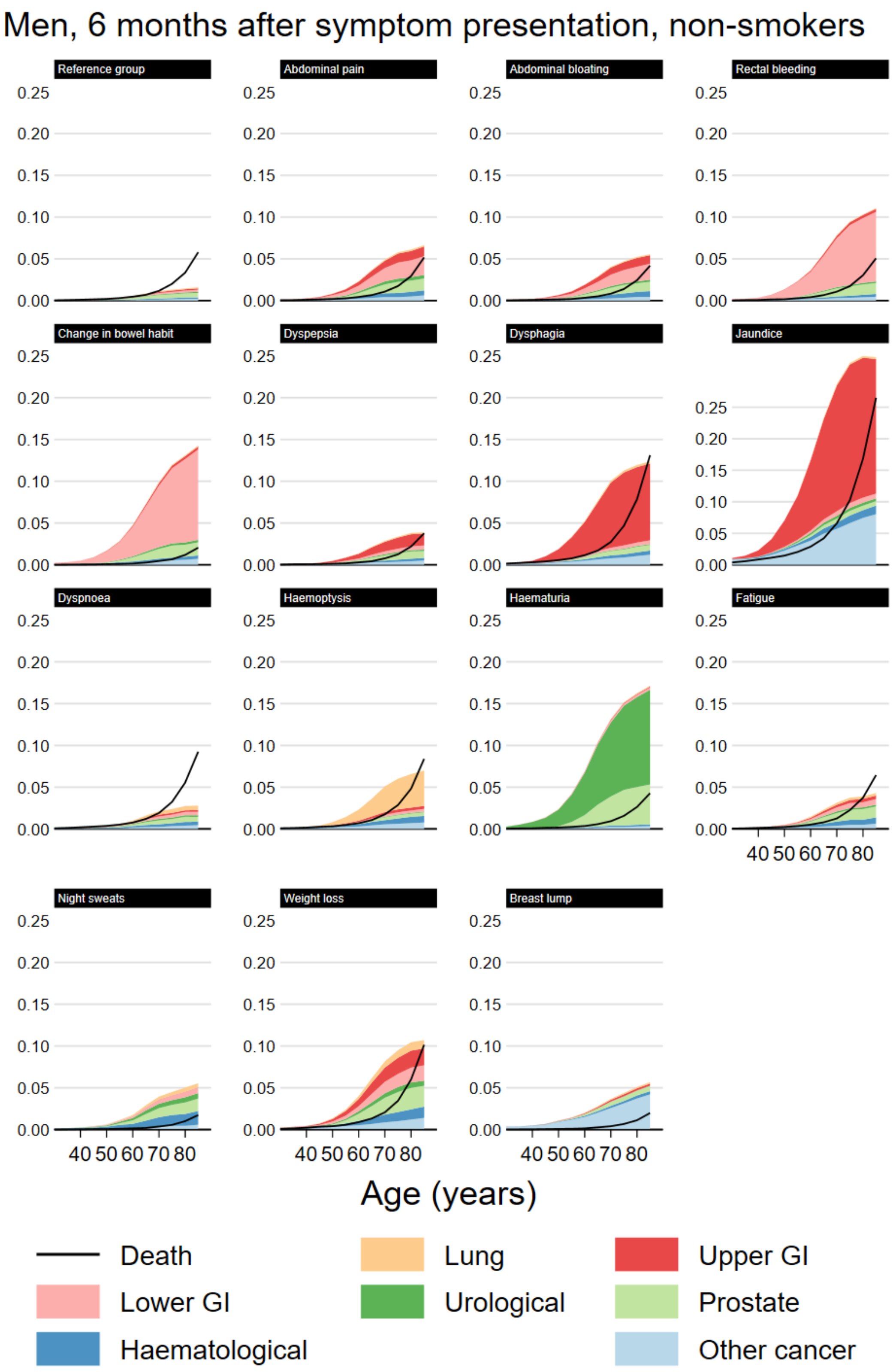

Appendix 4 Figure 6. Cumulative incidence at 6 months, male smokers

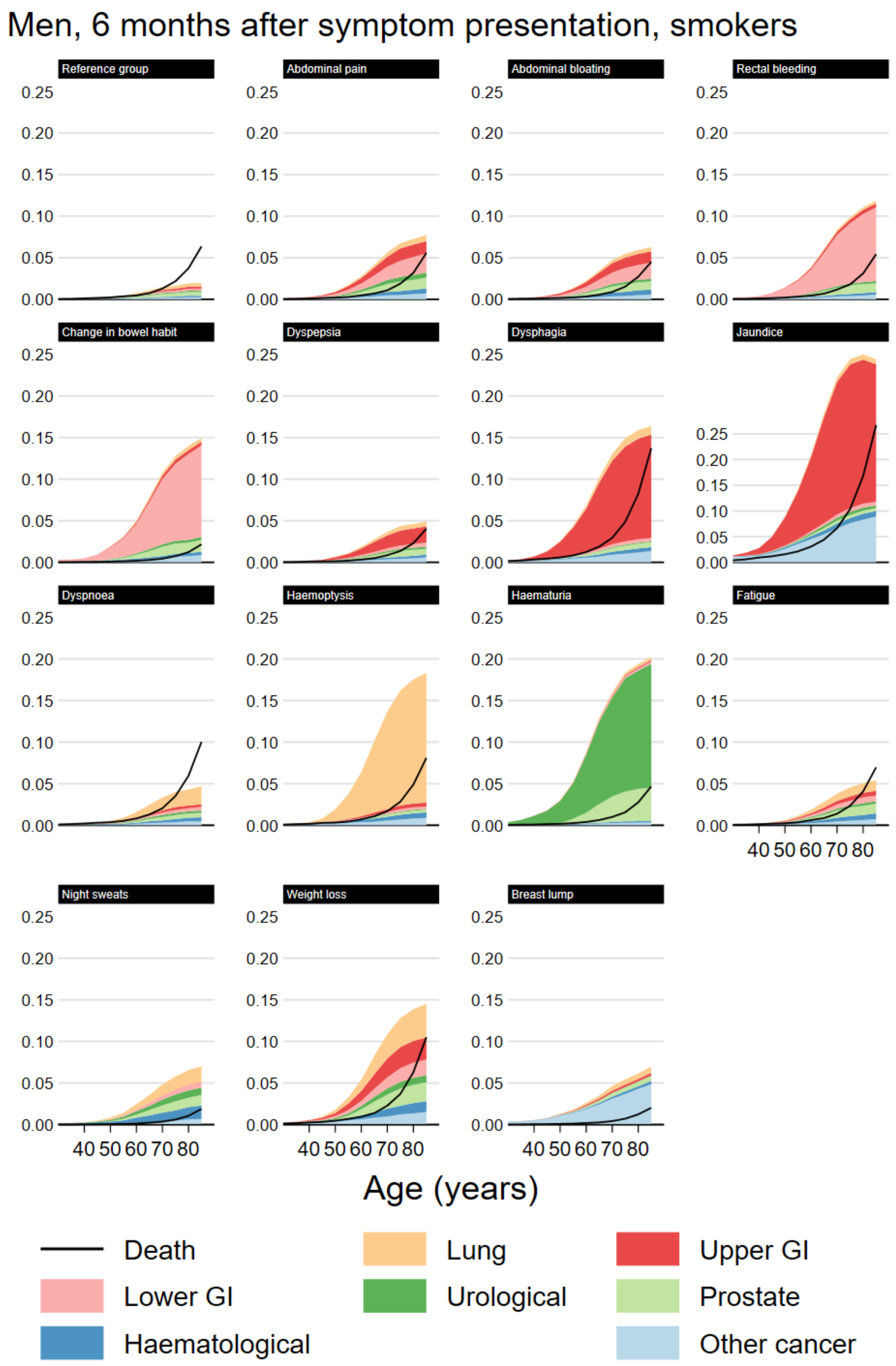

Appendix 4 Figure 7. Cumulative incidence at 6 months, female non-smokers

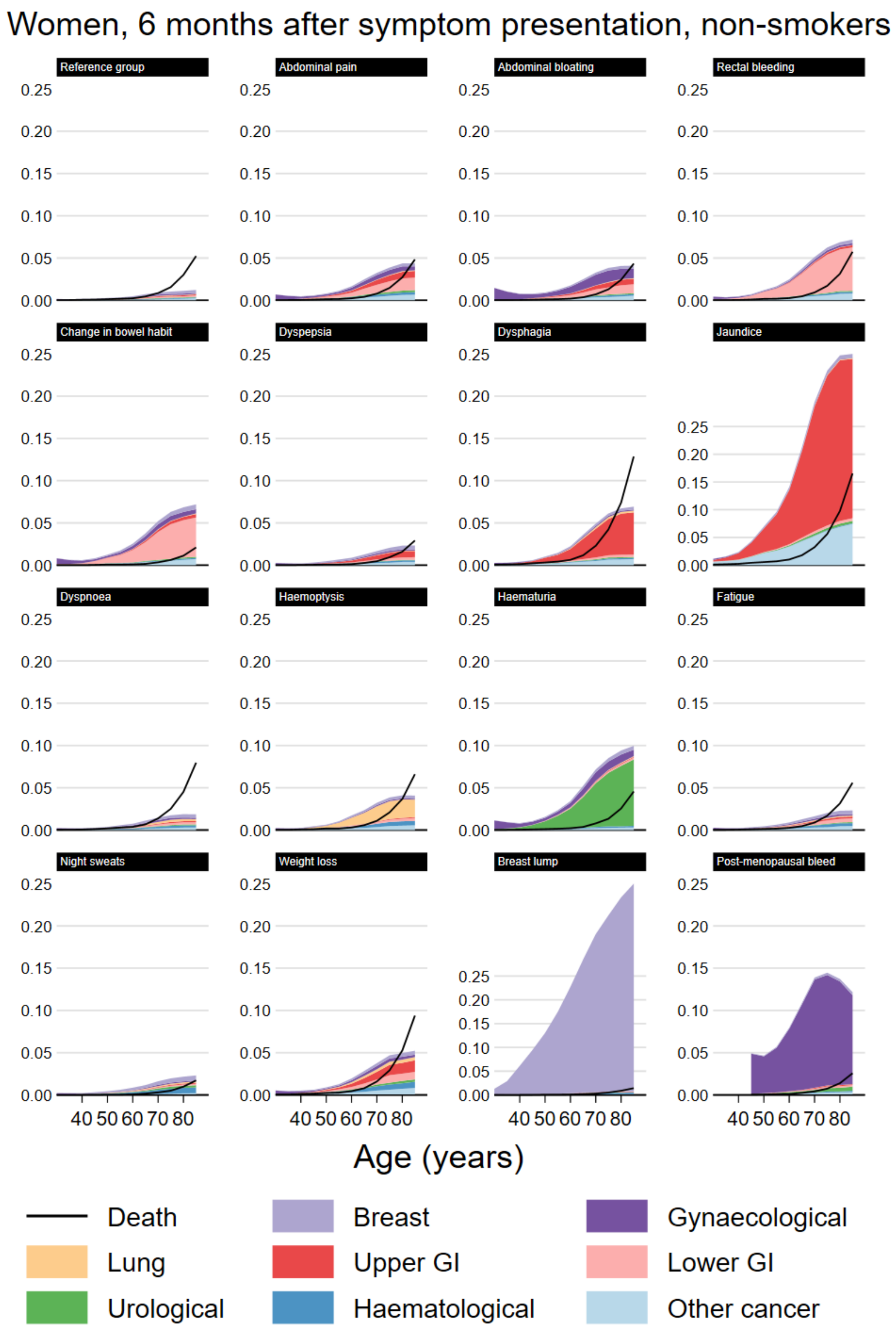

Appendix 4 Figure 8. Cumulative incidence at 6 months, female smokers

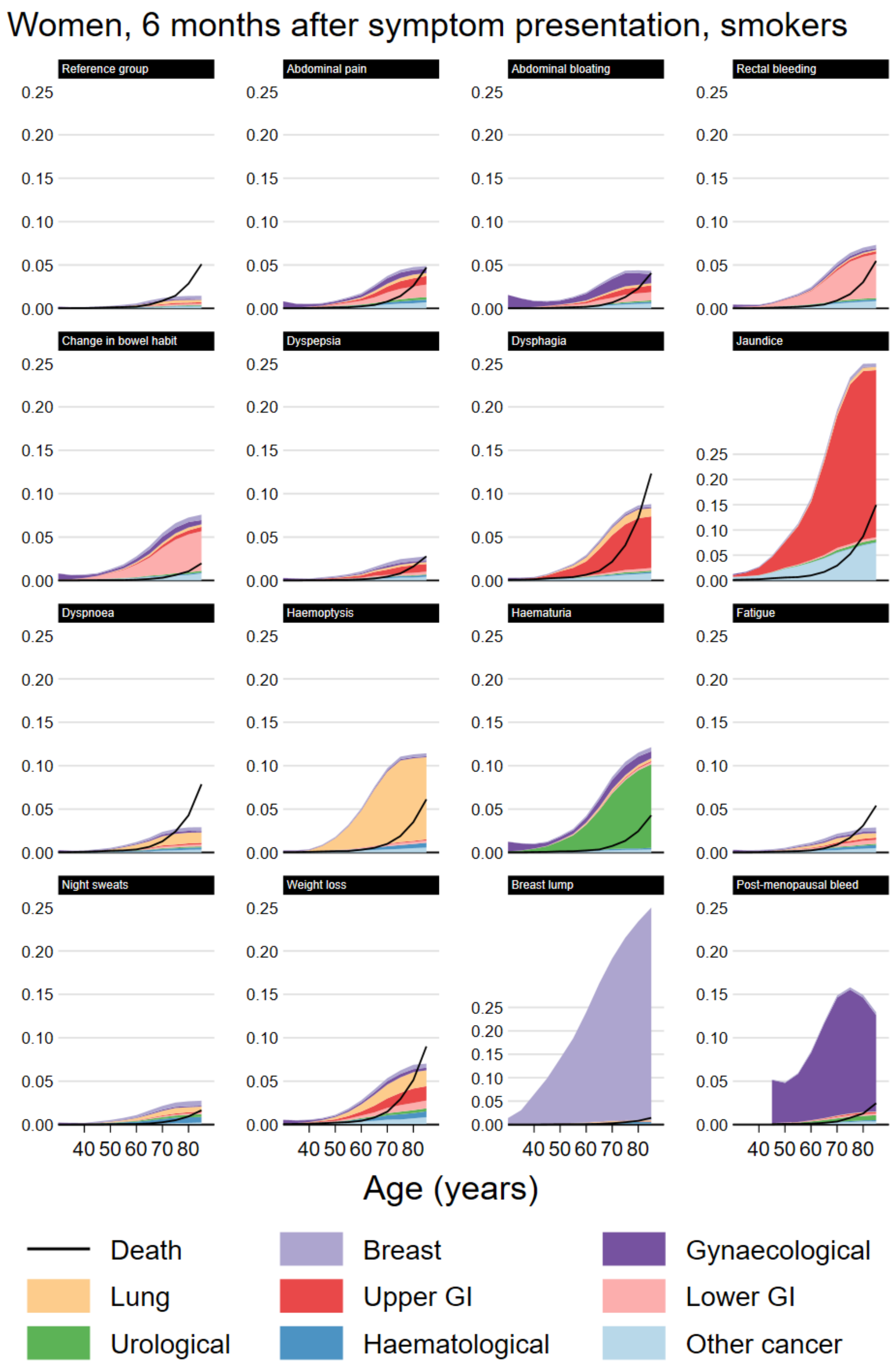

Appendix 5. Table showing simulated outcomes at 12 months after index symptom by age, sex and smoking status.

Appendix 5 Table 1. Estimated probability of cancer and mortality within 12 months for patients presenting aged 30, 40, 50, 60, 70, 80, 90 with each index symptom. Only probabilities greater than 0.001 are shown.

Sex	Index symptom	Smoking status	Age (years)	Outcome event	Probability of outcome	(95% CI)
Women	Abdominal pain	Never smoker	30	Gyanecological	0.008	(0.003, 0.021)
Women	Abdominal pain	Never smoker	40	Gyanecological	0.003	(0.002, 0.007)
Women	Abdominal pain	Never smoker	50	Breast	0.002	(<0.001, 0.003)
Women	Abdominal pain	Never smoker	50	Gyanecological	0.003	(0.001, 0.005)
Women	Abdominal pain	Never smoker	50	Lower GI	0.003	(0.001, 0.006)
Women	Abdominal pain	Never smoker	50	Other cancers	0.002	(<0.001, 0.005)
Women	Abdominal pain	Never smoker	50	Death w/o cancer	0.002	(<0.001, 0.004)
Women	Abdominal pain	Never smoker	60	Breast	0.003	(0.001, 0.009)
Women	Abdominal pain	Never smoker	60	Gyanecological	0.004	(0.002, 0.007)
Women	Abdominal pain	Never smoker	60	Upper GI	0.003	(0.002, 0.006)
Women	Abdominal pain	Never smoker	60	Lower GI	0.005	(0.003, 0.011)
Women	Abdominal pain	Never smoker	60	Haematological	0.002	(<0.001, 0.004)
Women	Abdominal pain	Never smoker	60	Other cancers	0.004	(0.001, 0.010)
Women	Abdominal pain	Never smoker	60	Death w/o cancer	0.004	(0.001, 0.011)
Women	Abdominal pain	Never smoker	70	Breast	0.005	(0.002, 0.011)
Women	Abdominal pain	Never smoker	70	Gyanecological	0.007	(0.003, 0.017)
Women	Abdominal pain	Never smoker	70	Upper GI	0.007	(0.003, 0.015)
Women	Abdominal pain	Never smoker	70	Lower GI	0.012	(0.007, 0.022)
Women	Abdominal pain	Never smoker	70	Urological	0.002	(0.001, 0.004)
Women	Abdominal pain	Never smoker	70	Haematological	0.003	(0.002, 0.008)
Women	Abdominal pain	Never smoker	70	Other cancers	0.006	(0.002, 0.014)
Women	Abdominal pain	Never smoker	70	Death w/o cancer	0.012	(0.007, 0.019)
Women	Abdominal pain	Never smoker	80	Breast	0.007	(0.003, 0.015)
Women	Abdominal pain	Never smoker	80	Gyanecological	0.007	(0.004, 0.013)
Women	Abdominal pain	Never smoker	80	Lung	0.001	(<0.001, 0.003)
Women	Abdominal pain	Never smoker	80	Upper GI	0.010	(0.006, 0.017)
Women	Abdominal pain	Never smoker	80	Lower GI	0.017	(0.011, 0.026)
Women	Abdominal pain	Never smoker	80	Urological	0.003	(0.001, 0.007)
Women	Abdominal pain	Never smoker	80	Haematological	0.004	(0.002, 0.008)
Women	Abdominal pain	Never smoker	80	Other cancers	0.008	(0.004, 0.019)
Women	Abdominal pain	Never smoker	80	Death w/o cancer	0.040	(0.027, 0.059)
Women	Abdominal pain	Never smoker	90	Breast	0.008	(0.004, 0.016)
Women	Abdominal pain	Never smoker	90	Gyanecological	0.005	(0.002, 0.012)
Women	Abdominal pain	Never smoker	90	Upper GI	0.010	(0.004, 0.027)
Women	Abdominal pain	Never smoker	90	Lower GI	0.018	(0.009, 0.033)
Women	Abdominal pain	Never smoker	90	Urological	0.003	(<0.001, 0.000)
Women	Abdominal pain	Never smoker	90	Haematological	0.004	(0.002, 0.009)
Women	Abdominal pain	Never smoker	90	Other cancers	0.010	(0.005, 0.018)
Women	Abdominal pain	Never smoker	90	Death w/o cancer	0.124	(0.107, 0.143)
Women	Abdominal pain	Ever smoker	30	Gyanecological	0.010	(0.003, 0.034)
Women	Abdominal pain	Ever smoker	40	Gyanecological	0.004	(0.001, 0.011)
Women	Abdominal pain	Ever smoker	40	Other cancers	0.001	(<0.001, 0.002)
Women	Abdominal pain	Ever smoker	50	Breast	0.002	(<0.001, 0.004)
Women	Abdominal pain	Ever smoker	50	Gyanecological	0.003	(0.001, 0.005)
Women	Abdominal pain	Ever smoker	50	Upper GI	0.001	(<0.001, 0.003)
Women	Abdominal pain	Ever smoker	50	Lower GI	0.002	(0.001, 0.005)
Women	Abdominal pain	Ever smoker	50	Other cancers	0.002	(0.001, 0.005)
Women	Abdominal pain	Ever smoker	50	Death w/o cancer	0.002	(<0.001, 0.004)
Women	Abdominal pain	Ever smoker	60	Breast	0.004	(0.002, 0.008)
Women	Abdominal pain	Ever smoker	60	Gyanecological	0.004	(0.002, 0.010)
Women	Abdominal pain	Ever smoker	60	Lung	0.002	(<0.001, 0.006)

Sex	Index symptom	Smoking status	Age (years)	Outcome event	Probability of outcome	(95% CI)
Women	Abdominal pain	Ever smoker	60	Upper GI	0.003	(0.001, 0.007)
Women	Abdominal pain	Ever smoker	60	Lower GI	0.005	(0.002, 0.018)
Women	Abdominal pain	Ever smoker	60	Urological	0.001	(<0.001, 0.002)
Women	Abdominal pain	Ever smoker	60	Haematological	0.002	(<0.001, 0.004)
Women	Abdominal pain	Ever smoker	60	Other cancers	0.003	(0.001, 0.010)
Women	Abdominal pain	Ever smoker	60	Death w/o cancer	0.003	(0.002, 0.007)
Women	Abdominal pain	Ever smoker	70	Breast	0.005	(0.002, 0.013)
Women	Abdominal pain	Ever smoker	70	Gyanecological	0.008	(0.004, 0.017)
Women	Abdominal pain	Ever smoker	70	Lung	0.005	(0.002, 0.011)
Women	Abdominal pain	Ever smoker	70	Upper GI	0.008	(0.003, 0.022)
Women	Abdominal pain	Ever smoker	70	Lower GI	0.012	(0.007, 0.019)
Women	Abdominal pain	Ever smoker	70	Urological	0.002	(<0.001, 0.007)
Women	Abdominal pain	Ever smoker	70	Haematological	0.003	(0.002, 0.005)
Women	Abdominal pain	Ever smoker	70	Other cancers	0.006	(0.002, 0.017)
Women	Abdominal pain	Ever smoker	70	Death w/o cancer	0.012	(0.006, 0.024)
Women	Abdominal pain	Ever smoker	80	Breast	0.006	(<0.001, 0.000)
Women	Abdominal pain	Ever smoker	80	Gyanecological	0.007	(0.004, 0.014)
Women	Abdominal pain	Ever smoker	80	Lung	0.006	(0.003, 0.015)
Women	Abdominal pain	Ever smoker	80	Upper GI	0.012	(0.006, 0.023)
Women	Abdominal pain	Ever smoker	80	Lower GI	0.016	(0.010, 0.027)
Women	Abdominal pain	Ever smoker	80	Urological	0.004	(0.001, 0.009)
Women	Abdominal pain	Ever smoker	80	Haematological	0.004	(0.002, 0.009)
Women	Abdominal pain	Ever smoker	80	Other cancers	0.008	(0.004, 0.016)
Women	Abdominal pain	Ever smoker	80	Death w/o cancer	0.039	(0.028, 0.053)
Women	Abdominal pain	Ever smoker	90	Breast	0.008	(0.003, 0.021)
Women	Abdominal pain	Ever smoker	90	Gyanecological	0.005	(0.002, 0.016)
Women	Abdominal pain	Ever smoker	90	Lung	0.005	(0.003, 0.008)
Women	Abdominal pain	Ever smoker	90	Upper GI	0.012	(0.006, 0.021)
Women	Abdominal pain	Ever smoker	90	Lower GI	0.017	(0.011, 0.027)
Women	Abdominal pain	Ever smoker	90	Urological	0.004	(0.001, 0.011)
Women	Abdominal pain	Ever smoker	90	Haematological	0.004	(0.002, 0.009)
Women	Abdominal pain	Ever smoker	90	Other cancers	0.011	(0.005, 0.021)
Women	Abdominal pain	Ever smoker	90	Death w/o cancer	0.120	(0.102, 0.140)
Women	Abdominal bloating	Never smoker	30	Gyanecological	0.016	(0.010, 0.027)
Women	Abdominal bloating	Never smoker	40	Gyanecological	0.007	(0.003, 0.018)
Women	Abdominal bloating	Never smoker	50	Breast	0.002	(0.001, 0.004)
Women	Abdominal bloating	Never smoker	50	Gyanecological	0.005	(0.002, 0.015)
Women	Abdominal bloating	Never smoker	50	Lower GI	0.002	(<0.001, 0.004)
Women	Abdominal bloating	Never smoker	50	Other cancers	0.002	(<0.001, 0.003)
Women	Abdominal bloating	Never smoker	50	Death w/o cancer	0.002	(<0.001, 0.004)
Women	Abdominal bloating	Never smoker	60	Breast	0.003	(0.001, 0.008)
Women	Abdominal bloating	Never smoker	60	Gyanecological	0.009	(0.004, 0.022)
Women	Abdominal bloating	Never smoker	60	Upper GI	0.002	(<0.001, 0.005)
Women	Abdominal bloating	Never smoker	60	Lower GI	0.004	(0.002, 0.007)
Women	Abdominal bloating	Never smoker	60	Other cancers	0.003	(0.001, 0.009)
Women	Abdominal bloating	Never smoker	60	Death w/o cancer	0.003	(0.001, 0.008)
Women	Abdominal bloating	Never smoker	70	Breast	0.005	(0.002, 0.014)
Women	Abdominal bloating	Never smoker	70	Gyanecological	0.015	(0.009, 0.024)
Women	Abdominal bloating	Never smoker	70	Upper GI	0.006	(0.003, 0.015)
Women	Abdominal bloating	Never smoker	70	Lower GI	0.009	(0.004, 0.018)
Women	Abdominal bloating	Never smoker	70	Urological	0.002	(<0.001, 0.003)
Women	Abdominal bloating	Never smoker	70	Haematological	0.002	(<0.001, 0.004)
Women	Abdominal bloating	Never smoker	70	Other cancers	0.005	(0.002, 0.014)
Women	Abdominal bloating	Never smoker	70	Death w/o cancer	0.011	(0.006, 0.020)
Women	Abdominal bloating	Never smoker	80	Breast	0.007	(0.003, 0.015)
Women	Abdominal bloating	Never smoker	80	Gyanecological	0.014	(0.010, 0.021)
Women	Abdominal bloating	Never smoker	80	Upper GI	0.008	(0.005, 0.014)
Women	Abdominal bloating	Never smoker	80	Lower GI	0.012	(0.007, 0.021)
Women	Abdominal bloating	Never smoker	80	Urological	0.002	(<0.001, 0.004)
Women	Abdominal bloating	Never smoker	80	Haematological	0.003	(0.001, 0.006)
Women	Abdominal bloating	Never smoker	80	Other cancers	0.007	(0.003, 0.016)
Women	Abdominal bloating	Never smoker	80	Death w/o cancer	0.038	(0.025, 0.057)

Sex	Index symptom	Smoking status	Age (years)	Outcome event	Probability of outcome	(95% CI)
Women	Abdominal bloating	Never smoker	90	Breast	0.008	(0.004, 0.015)
Women	Abdominal bloating	Never smoker	90	Gyanecological	0.010	(0.006, 0.016)
Women	Abdominal bloating	Never smoker	90	Upper GI	0.008	(0.003, 0.025)
Women	Abdominal bloating	Never smoker	90	Lower GI	0.013	(0.006, 0.025)
Women	Abdominal bloating	Never smoker	90	Urological	0.002	(0.001, 0.005)
Women	Abdominal bloating	Never smoker	90	Haematological	0.003	(0.001, 0.007)
Women	Abdominal bloating	Never smoker	90	Other cancers	0.009	(0.004, 0.019)
Women	Abdominal bloating	Never smoker	90	Death w/o cancer	0.119	(0.098, 0.143)
Women	Abdominal bloating	Ever smoker	30	Gyanecological	0.017	(0.010, 0.029)
Women	Abdominal bloating	Ever smoker	40	Breast	0.001	(<0.001, 0.001)
Women	Abdominal bloating	Ever smoker	40	Gyanecological	0.008	(0.004, 0.014)
Women	Abdominal bloating	Ever smoker	50	Breast	0.002	(0.001, 0.004)
Women	Abdominal bloating	Ever smoker	50	Gyanecological	0.005	(0.003, 0.009)
Women	Abdominal bloating	Ever smoker	50	Upper GI	0.001	(<0.001, 0.002)
Women	Abdominal bloating	Ever smoker	50	Lower GI	0.002	(<0.001, 0.003)
Women	Abdominal bloating	Ever smoker	50	Other cancers	0.002	(<0.001, 0.004)
Women	Abdominal bloating	Ever smoker	50	Death w/o cancer	0.002	(<0.001, 0.004)
Women	Abdominal bloating	Ever smoker	60	Breast	0.003	(0.001, 0.007)
Women	Abdominal bloating	Ever smoker	60	Gyanecological	0.009	(0.005, 0.016)
Women	Abdominal bloating	Ever smoker	60	Lung	0.002	(0.001, 0.003)
Women	Abdominal bloating	Ever smoker	60	Upper GI	0.003	(0.002, 0.005)
Women	Abdominal bloating	Ever smoker	60	Lower GI	0.003	(0.001, 0.009)
Women	Abdominal bloating	Ever smoker	60	Haematological	0.001	(<0.001, 0.002)
Women	Abdominal bloating	Ever smoker	60	Other cancers	0.003	(0.001, 0.008)
Women	Abdominal bloating	Ever smoker	60	Death w/o cancer	0.003	(0.001, 0.006)
Women	Abdominal bloating	Ever smoker	70	Breast	0.006	(0.003, 0.012)
Women	Abdominal bloating	Ever smoker	70	Gyanecological	0.016	(0.010, 0.027)
Women	Abdominal bloating	Ever smoker	70	Lung	0.003	(0.001, 0.008)
Women	Abdominal bloating	Ever smoker	70	Upper GI	0.008	(0.003, 0.020)
Women	Abdominal bloating	Ever smoker	70	Lower GI	0.008	(0.004, 0.014)
Women	Abdominal bloating	Ever smoker	70	Urological	0.002	(<0.001, 0.004)
Women	Abdominal bloating	Ever smoker	70	Haematological	0.002	(0.001, 0.003)
Women	Abdominal bloating	Ever smoker	70	Other cancers	0.006	(0.002, 0.016)
Women	Abdominal bloating	Ever smoker	70	Death w/o cancer	0.011	(0.005, 0.023)
Women	Abdominal bloating	Ever smoker	80	Breast	0.007	(0.003, 0.017)
Women	Abdominal bloating	Ever smoker	80	Gyanecological	0.015	(0.010, 0.023)
Women	Abdominal bloating	Ever smoker	80	Lung	0.004	(0.001, 0.011)
Women	Abdominal bloating	Ever smoker	80	Upper GI	0.009	(0.004, 0.020)
Women	Abdominal bloating	Ever smoker	80	Lower GI	0.011	(0.006, 0.021)
Women	Abdominal bloating	Ever smoker	80	Urological	0.002	(<0.001, 0.006)
Women	Abdominal bloating	Ever smoker	80	Haematological	0.003	(0.001, 0.004)
Women	Abdominal bloating	Ever smoker	80	Other cancers	0.007	(0.004, 0.014)
Women	Abdominal bloating	Ever smoker	80	Death w/o cancer	0.037	(0.027, 0.052)
Women	Abdominal bloating	Ever smoker	90	Breast	0.008	(0.003, 0.021)
Women	Abdominal bloating	Ever smoker	90	Gyanecological	0.011	(0.006, 0.021)
Women	Abdominal bloating	Ever smoker	90	Lung	0.003	(0.001, 0.010)
Women	Abdominal bloating	Ever smoker	90	Upper GI	0.010	(0.005, 0.019)
Women	Abdominal bloating	Ever smoker	90	Lower GI	0.012	(0.007, 0.020)
Women	Abdominal bloating	Ever smoker	90	Urological	0.003	(0.001, 0.008)
Women	Abdominal bloating	Ever smoker	90	Haematological	0.003	(0.001, 0.006)
Women	Abdominal bloating	Ever smoker	90	Other cancers	0.009	(0.005, 0.018)
Women	Abdominal bloating	Ever smoker	90	Death w/o cancer	0.115	(0.091, 0.145)
Women	Breast lump	Never smoker	30	Breast	0.011	(0.006, 0.021)
Women	Breast lump	Never smoker	30	Gyanecological	0.003	(0.001, 0.007)
Women	Breast lump	Never smoker	40	Breast	0.062	(0.046, 0.083)
Women	Breast lump	Never smoker	40	Gyanecological	0.001	(<0.001, 0.003)
Women	Breast lump	Never smoker	50	Breast	0.132	(0.110, 0.156)
Women	Breast lump	Never smoker	50	Haematological	0.001	(<0.001, 0.002)
Women	Breast lump	Never smoker	50	Other cancers	0.001	(<0.001, 0.002)
Women	Breast lump	Never smoker	50	Death w/o cancer	0.001	(<0.001, 0.003)
Women	Breast lump	Never smoker	60	Breast	0.230	(0.191, 0.274)
Women	Breast lump	Never smoker	60	Gyanecological	0.001	(<0.001, 0.003)

Sex	Index symptom	Smoking status	Age (years)	Outcome event	Probability of outcome	(95% CI)
Women	Breast lump	Never smoker	60	Haematological	0.002	(<0.001, 0.004)
Women	Breast lump	Never smoker	60	Other cancers	0.001	(<0.001, 0.003)
Women	Breast lump	Never smoker	60	Death w/o cancer	0.002	(<0.001, 0.005)
Women	Breast lump	Never smoker	70	Breast	0.340	(0.302, 0.380)
Women	Breast lump	Never smoker	70	Gyanecological	0.002	(<0.001, 0.004)
Women	Breast lump	Never smoker	70	Haematological	0.003	(0.001, 0.007)
Women	Breast lump	Never smoker	70	Other cancers	0.002	(<0.001, 0.006)
Women	Breast lump	Never smoker	70	Death w/o cancer	0.005	(0.002, 0.013)
Women	Breast lump	Never smoker	80	Breast	0.417	(0.380, 0.455)
Women	Breast lump	Never smoker	80	Gyanecological	0.002	(<0.001, 0.003)
Women	Breast lump	Never smoker	80	Upper GI	0.001	(<0.001, 0.002)
Women	Breast lump	Never smoker	80	Lower GI	0.001	(<0.001, 0.002)
Women	Breast lump	Never smoker	80	Haematological	0.004	(0.001, 0.011)
Women	Breast lump	Never smoker	80	Other cancers	0.003	(0.001, 0.007)
Women	Breast lump	Never smoker	80	Death w/o cancer	0.016	(0.009, 0.028)
Women	Breast lump	Never smoker	90	Breast	0.474	(0.427, 0.521)
Women	Breast lump	Never smoker	90	Gyanecological	0.001	(<0.001, 0.002)
Women	Breast lump	Never smoker	90	Upper GI	0.001	(<0.001, 0.002)
Women	Breast lump	Never smoker	90	Lower GI	0.001	(<0.001, 0.002)
Women	Breast lump	Never smoker	90	Haematological	0.004	(0.002, 0.009)
Women	Breast lump	Never smoker	90	Other cancers	0.003	(0.001, 0.009)
Women	Breast lump	Never smoker	90	Death w/o cancer	0.047	(0.033, 0.068)
Women	Breast lump	Ever smoker	30	Breast	0.012	(0.006, 0.026)
Women	Breast lump	Ever smoker	30	Gyanecological	0.003	(0.001, 0.008)
Women	Breast lump	Ever smoker	40	Breast	0.065	(0.051, 0.082)
Women	Breast lump	Ever smoker	40	Gyanecological	0.002	(<0.001, 0.003)
Women	Breast lump	Ever smoker	50	Breast	0.142	(0.114, 0.175)
Women	Breast lump	Ever smoker	50	Other cancers	0.001	(<0.001, 0.002)
Women	Breast lump	Ever smoker	50	Death w/o cancer	0.001	(<0.001, 0.002)
Women	Breast lump	Ever smoker	60	Breast	0.241	(0.208, 0.277)
Women	Breast lump	Ever smoker	60	Gyanecological	0.001	(<0.001, 0.003)
Women	Breast lump	Ever smoker	60	Lung	0.002	(<0.001, 0.003)
Women	Breast lump	Ever smoker	60	Haematological	0.002	(<0.001, 0.004)
Women	Breast lump	Ever smoker	60	Other cancers	0.002	(<0.001, 0.003)
Women	Breast lump	Ever smoker	60	Death w/o cancer	0.002	(<0.001, 0.003)
Women	Breast lump	Ever smoker	70	Breast	0.355	(0.323, 0.387)
Women	Breast lump	Ever smoker	70	Gyanecological	0.002	(<0.001, 0.004)
Women	Breast lump	Ever smoker	70	Lung	0.003	(0.001, 0.008)
Women	Breast lump	Ever smoker	70	Upper GI	0.001	(<0.001, 0.002)
Women	Breast lump	Ever smoker	70	Haematological	0.003	(0.001, 0.007)
Women	Breast lump	Ever smoker	70	Other cancers	0.003	(0.001, 0.005)
Women	Breast lump	Ever smoker	70	Death w/o cancer	0.005	(0.002, 0.012)
Women	Breast lump	Ever smoker	80	Breast	0.433	(0.385, 0.482)
Women	Breast lump	Ever smoker	80	Gyanecological	0.002	(<0.001, 0.004)
Women	Breast lump	Ever smoker	80	Lung	0.003	(0.001, 0.008)
Women	Breast lump	Ever smoker	80	Upper GI	0.001	(<0.001, 0.002)
Women	Breast lump	Ever smoker	80	Lower GI	0.001	(<0.001, 0.002)
Women	Breast lump	Ever smoker	80	Haematological	0.004	(0.001, 0.011)
Women	Breast lump	Ever smoker	80	Other cancers	0.003	(0.001, 0.008)
Women	Breast lump	Ever smoker	80	Death w/o cancer	0.015	(0.010, 0.024)
Women	Breast lump	Ever smoker	90	Breast	0.496	(0.460, 0.533)
Women	Breast lump	Ever smoker	90	Gyanecological	0.001	(<0.001, 0.002)
Women	Breast lump	Ever smoker	90	Lung	0.002	(<0.001, 0.007)
Women	Breast lump	Ever smoker	90	Upper GI	0.001	(<0.001, 0.002)
Women	Breast lump	Ever smoker	90	Haematological	0.004	(0.002, 0.006)
Women	Breast lump	Ever smoker	90	Other cancers	0.003	(0.001, 0.008)
Women	Breast lump	Ever smoker	90	Death w/o cancer	0.044	(0.032, 0.061)
Women	Change in bowel habit	Never smoker	30	Gyanecological	0.009	(0.003, 0.022)
Women	Change in bowel habit	Never smoker	40	Gyanecological	0.004	(0.001, 0.012)
Women	Change in bowel habit	Never smoker	40	Lower GI	0.002	(<0.001, 0.003)
Women	Change in bowel habit	Never smoker	50	Breast	0.002	(0.001, 0.005)
Women	Change in bowel habit	Never smoker	50	Gyanecological	0.003	(0.001, 0.005)

Sex	Index symptom	Smoking status	Age (years)	Outcome event	Probability of outcome	(95% CI)
Women	Change in bowel habit	Never smoker	50	Lower GI	0.007	(0.003, 0.015)
Women	Change in bowel habit	Never smoker	50	Other cancers	0.002	(0.001, 0.006)
Women	Change in bowel habit	Never smoker	50	Death w/o cancer	0.001	(<0.001, 0.002)
Women	Change in bowel habit	Never smoker	60	Breast	0.004	(0.002, 0.010)
Women	Change in bowel habit	Never smoker	60	Gyanecological	0.005	(0.002, 0.013)
Women	Change in bowel habit	Never smoker	60	Upper GI	0.001	(<0.001, 0.003)
Women	Change in bowel habit	Never smoker	60	Lower GI	0.016	(0.010, 0.025)
Women	Change in bowel habit	Never smoker	60	Other cancers	0.003	(0.002, 0.006)
Women	Change in bowel habit	Never smoker	60	Death w/o cancer	0.002	(<0.001, 0.004)
Women	Change in bowel habit	Never smoker	70	Breast	0.007	(0.002, 0.018)
Women	Change in bowel habit	Never smoker	70	Gyanecological	0.008	(0.004, 0.014)
Women	Change in bowel habit	Never smoker	70	Lung	0.001	(<0.001, 0.002)
Women	Change in bowel habit	Never smoker	70	Upper GI	0.003	(0.001, 0.009)
Women	Change in bowel habit	Never smoker	70	Lower GI	0.037	(0.027, 0.051)
Women	Change in bowel habit	Never smoker	70	Urological	0.002	(<0.001, 0.003)
Women	Change in bowel habit	Never smoker	70	Haematological	0.001	(<0.001, 0.003)
Women	Change in bowel habit	Never smoker	70	Other cancers	0.006	(0.003, 0.014)
Women	Change in bowel habit	Never smoker	70	Death w/o cancer	0.006	(0.002, 0.016)
Women	Change in bowel habit	Never smoker	80	Breast	0.009	(0.004, 0.017)
Women	Change in bowel habit	Never smoker	80	Gyanecological	0.007	(0.004, 0.013)
Women	Change in bowel habit	Never smoker	80	Upper GI	0.005	(0.002, 0.010)
Women	Change in bowel habit	Never smoker	80	Lower GI	0.049	(0.036, 0.066)
Women	Change in bowel habit	Never smoker	80	Urological	0.002	(<0.001, 0.004)
Women	Change in bowel habit	Never smoker	80	Haematological	0.002	(<0.001, 0.006)
Women	Change in bowel habit	Never smoker	80	Other cancers	0.009	(0.004, 0.020)
Women	Change in bowel habit	Never smoker	80	Death w/o cancer	0.021	(0.015, 0.031)
Women	Change in bowel habit	Never smoker	90	Breast	0.010	(0.006, 0.016)
Women	Change in bowel habit	Never smoker	90	Gyanecological	0.005	(0.003, 0.009)
Women	Change in bowel habit	Never smoker	90	Lung	0.001	(<0.001, 0.002)
Women	Change in bowel habit	Never smoker	90	Upper GI	0.005	(0.002, 0.012)
Women	Change in bowel habit	Never smoker	90	Lower GI	0.055	(0.036, 0.082)
Women	Change in bowel habit	Never smoker	90	Urological	0.002	(0.001, 0.004)
Women	Change in bowel habit	Never smoker	90	Haematological	0.002	(<0.001, 0.004)
Women	Change in bowel habit	Never smoker	90	Other cancers	0.011	(0.006, 0.022)
Women	Change in bowel habit	Never smoker	90	Death w/o cancer	0.065	(0.047, 0.090)
Women	Change in bowel habit	Ever smoker	30	Gyanecological	0.009	(0.004, 0.021)
Women	Change in bowel habit	Ever smoker	40	Breast	0.001	(<0.001, 0.002)
Women	Change in bowel habit	Ever smoker	40	Gyanecological	0.004	(0.002, 0.011)
Women	Change in bowel habit	Ever smoker	40	Lower GI	0.002	(<0.001, 0.004)
Women	Change in bowel habit	Ever smoker	40	Other cancers	0.001	(<0.001, 0.002)
Women	Change in bowel habit	Ever smoker	50	Breast	0.002	(0.001, 0.004)
Women	Change in bowel habit	Ever smoker	50	Gyanecological	0.003	(0.001, 0.006)
Women	Change in bowel habit	Ever smoker	50	Lower GI	0.007	(0.003, 0.015)
Women	Change in bowel habit	Ever smoker	50	Other cancers	0.002	(0.001, 0.006)
Women	Change in bowel habit	Ever smoker	60	Breast	0.005	(0.003, 0.008)
Women	Change in bowel habit	Ever smoker	60	Gyanecological	0.005	(0.002, 0.013)
Women	Change in bowel habit	Ever smoker	60	Lung	0.002	(0.001, 0.006)
Women	Change in bowel habit	Ever smoker	60	Upper GI	0.002	(<0.001, 0.004)
Women	Change in bowel habit	Ever smoker	60	Lower GI	0.016	(0.010, 0.028)
Women	Change in bowel habit	Ever smoker	60	Other cancers	0.004	(0.002, 0.008)
Women	Change in bowel habit	Ever smoker	60	Death w/o cancer	0.002	(<0.001, 0.004)
Women	Change in bowel habit	Ever smoker	70	Breast	0.007	(<0.001, 0.000)
Women	Change in bowel habit	Ever smoker	70	Gyanecological	0.009	(0.005, 0.014)
Women	Change in bowel habit	Ever smoker	70	Lung	0.005	(0.002, 0.013)
Women	Change in bowel habit	Ever smoker	70	Upper GI	0.004	(0.002, 0.011)
Women	Change in bowel habit	Ever smoker	70	Lower GI	0.034	(0.024, 0.047)
Women	Change in bowel habit	Ever smoker	70	Urological	0.002	(<0.001, 0.004)
Women	Change in bowel habit	Ever smoker	70	Haematological	0.002	(<0.001, 0.004)
Women	Change in bowel habit	Ever smoker	70	Other cancers	0.006	(0.002, 0.021)
Women	Change in bowel habit	Ever smoker	70	Death w/o cancer	0.006	(0.003, 0.014)
Women	Change in bowel habit	Ever smoker	80	Breast	0.008	(0.004, 0.016)
Women	Change in bowel habit	Ever smoker	80	Gyanecological	0.008	(0.004, 0.016)

Sex	Index symptom	Smoking status	Age (years)	Outcome event	Probability of outcome	(95% CI)
Women	Change in bowel habit	Ever smoker	80	Lung	0.005	(0.002, 0.011)
Women	Change in bowel habit	Ever smoker	80	Upper GI	0.006	(0.002, 0.015)
Women	Change in bowel habit	Ever smoker	80	Lower GI	0.049	(0.036, 0.065)
Women	Change in bowel habit	Ever smoker	80	Urological	0.003	(0.001, 0.006)
Women	Change in bowel habit	Ever smoker	80	Haematological	0.002	(<0.001, 0.004)
Women	Change in bowel habit	Ever smoker	80	Other cancers	0.009	(0.003, 0.025)
Women	Change in bowel habit	Ever smoker	80	Death w/o cancer	0.020	(0.013, 0.032)
Women	Change in bowel habit	Ever smoker	90	Breast	0.011	(0.005, 0.021)
Women	Change in bowel habit	Ever smoker	90	Gyanecological	0.005	(0.003, 0.011)
Women	Change in bowel habit	Ever smoker	90	Lung	0.005	(0.002, 0.014)
Women	Change in bowel habit	Ever smoker	90	Upper GI	0.006	(0.003, 0.012)
Women	Change in bowel habit	Ever smoker	90	Lower GI	0.051	(0.036, 0.070)
Women	Change in bowel habit	Ever smoker	90	Urological	0.003	(0.001, 0.008)
Women	Change in bowel habit	Ever smoker	90	Haematological	0.002	(<0.001, 0.005)
Women	Change in bowel habit	Ever smoker	90	Other cancers	0.011	(0.005, 0.024)
Women	Change in bowel habit	Ever smoker	90	Death w/o cancer	0.064	(0.045, 0.091)
Women	Dyspepsia	Never smoker	30	Gyanecological	0.003	(0.002, 0.005)
Women	Dyspepsia	Never smoker	40	Gyanecological	0.001	(<0.001, 0.002)
Women	Dyspepsia	Never smoker	50	Breast	0.002	(<0.001, 0.005)
Women	Dyspepsia	Never smoker	50	Upper GI	0.001	(<0.001, 0.002)
Women	Dyspepsia	Never smoker	50	Other cancers	0.002	(<0.001, 0.003)
Women	Dyspepsia	Never smoker	50	Death w/o cancer	0.001	(<0.001, 0.003)
Women	Dyspepsia	Never smoker	60	Breast	0.004	(0.002, 0.009)
Women	Dyspepsia	Never smoker	60	Gyanecological	0.002	(<0.001, 0.004)
Women	Dyspepsia	Never smoker	60	Upper GI	0.003	(0.001, 0.006)
Women	Dyspepsia	Never smoker	60	Lower GI	0.001	(<0.001, 0.003)
Women	Dyspepsia	Never smoker	60	Haematological	0.001	(<0.001, 0.002)
Women	Dyspepsia	Never smoker	60	Other cancers	0.002	(<0.001, 0.006)
Women	Dyspepsia	Never smoker	60	Death w/o cancer	0.002	(0.001, 0.004)
Women	Dyspepsia	Never smoker	70	Breast	0.006	(0.002, 0.015)
Women	Dyspepsia	Never smoker	70	Gyanecological	0.003	(0.001, 0.006)
Women	Dyspepsia	Never smoker	70	Upper GI	0.006	(0.003, 0.014)
Women	Dyspepsia	Never smoker	70	Lower GI	0.004	(0.002, 0.008)
Women	Dyspepsia	Never smoker	70	Haematological	0.003	(0.001, 0.005)
Women	Dyspepsia	Never smoker	70	Other cancers	0.004	(0.001, 0.010)
Women	Dyspepsia	Never smoker	70	Death w/o cancer	0.008	(0.004, 0.017)
Women	Dyspepsia	Never smoker	80	Breast	0.008	(0.004, 0.015)
Women	Dyspepsia	Never smoker	80	Gyanecological	0.003	(0.001, 0.007)
Women	Dyspepsia	Never smoker	80	Upper GI	0.010	(0.003, 0.027)
Women	Dyspepsia	Never smoker	80	Lower GI	0.004	(0.002, 0.009)
Women	Dyspepsia	Never smoker	80	Haematological	0.003	(0.001, 0.007)
Women	Dyspepsia	Never smoker	80	Other cancers	0.006	(0.003, 0.012)
Women	Dyspepsia	Never smoker	80	Death w/o cancer	0.027	(0.020, 0.037)
Women	Dyspepsia	Never smoker	90	Breast	0.009	(0.004, 0.019)
Women	Dyspepsia	Never smoker	90	Gyanecological	0.002	(<0.001, 0.006)
Women	Dyspepsia	Never smoker	90	Upper GI	0.009	(0.005, 0.019)
Women	Dyspepsia	Never smoker	90	Lower GI	0.004	(0.002, 0.009)
Women	Dyspepsia	Never smoker	90	Haematological	0.003	(0.001, 0.009)
Women	Dyspepsia	Never smoker	90	Other cancers	0.006	(0.003, 0.011)
Women	Dyspepsia	Never smoker	90	Death w/o cancer	0.088	(0.068, 0.114)
Women	Dyspepsia	Ever smoker	30	Gyanecological	0.004	(0.001, 0.009)
Women	Dyspepsia	Ever smoker	40	Breast	0.001	(<0.001, 0.002)
Women	Dyspepsia	Ever smoker	40	Gyanecological	0.001	(<0.001, 0.003)
Women	Dyspepsia	Ever smoker	50	Breast	0.002	(<0.001, 0.005)
Women	Dyspepsia	Ever smoker	50	Gyanecological	0.001	(<0.001, 0.002)
Women	Dyspepsia	Ever smoker	50	Upper GI	0.001	(<0.001, 0.003)
Women	Dyspepsia	Ever smoker	50	Other cancers	0.002	(<0.001, 0.003)
Women	Dyspepsia	Ever smoker	50	Death w/o cancer	0.001	(<0.001, 0.002)
Women	Dyspepsia	Ever smoker	60	Breast	0.004	(0.002, 0.008)
Women	Dyspepsia	Ever smoker	60	Gyanecological	0.002	(0.001, 0.003)
Women	Dyspepsia	Ever smoker	60	Lung	0.002	(<0.001, 0.004)
Women	Dyspepsia	Ever smoker	60	Upper GI	0.003	(0.001, 0.009)

Sex	Index symptom	Smoking status	Age (years)	Outcome event	Probability of outcome	(95% CI)
Women	Dyspepsia	Ever smoker	60	Lower GI	0.001	(<0.001, 0.003)
Women	Dyspepsia	Ever smoker	60	Haematological	0.001	(<0.001, 0.002)
Women	Dyspepsia	Ever smoker	60	Other cancers	0.002	(<0.001, 0.005)
Women	Dyspepsia	Ever smoker	60	Death w/o cancer	0.002	(<0.001, 0.005)
Women	Dyspepsia	Ever smoker	70	Breast	0.006	(0.003, 0.015)
Women	Dyspepsia	Ever smoker	70	Gyanecological	0.003	(0.001, 0.008)
Women	Dyspepsia	Ever smoker	70	Lung	0.004	(0.001, 0.011)
Women	Dyspepsia	Ever smoker	70	Upper GI	0.008	(0.004, 0.016)
Women	Dyspepsia	Ever smoker	70	Lower GI	0.003	(0.001, 0.007)
Women	Dyspepsia	Ever smoker	70	Haematological	0.002	(0.001, 0.005)
Women	Dyspepsia	Ever smoker	70	Other cancers	0.004	(0.001, 0.011)
Women	Dyspepsia	Ever smoker	70	Death w/o cancer	0.008	(0.004, 0.016)
Women	Dyspepsia	Ever smoker	80	Breast	0.008	(0.003, 0.018)
Women	Dyspepsia	Ever smoker	80	Gyanecological	0.003	(0.001, 0.008)
Women	Dyspepsia	Ever smoker	80	Lung	0.004	(0.001, 0.012)
Women	Dyspepsia	Ever smoker	80	Upper GI	0.011	(0.006, 0.020)
Women	Dyspepsia	Ever smoker	80	Lower GI	0.004	(0.002, 0.009)
Women	Dyspepsia	Ever smoker	80	Urological	0.001	(<0.001, 0.002)
Women	Dyspepsia	Ever smoker	80	Haematological	0.003	(0.001, 0.006)
Women	Dyspepsia	Ever smoker	80	Other cancers	0.005	(0.002, 0.015)
Women	Dyspepsia	Ever smoker	80	Death w/o cancer	0.027	(0.018, 0.038)
Women	Dyspepsia	Ever smoker	90	Breast	0.010	(0.005, 0.018)
Women	Dyspepsia	Ever smoker	90	Gyanecological	0.003	(0.001, 0.005)
Women	Dyspepsia	Ever smoker	90	Lung	0.004	(0.001, 0.009)
Women	Dyspepsia	Ever smoker	90	Upper GI	0.012	(0.007, 0.020)
Women	Dyspepsia	Ever smoker	90	Lower GI	0.004	(0.002, 0.009)
Women	Dyspepsia	Ever smoker	90	Urological	0.001	(<0.001, 0.002)
Women	Dyspepsia	Ever smoker	90	Haematological	0.003	(0.001, 0.008)
Women	Dyspepsia	Ever smoker	90	Other cancers	0.007	(0.002, 0.020)
Women	Dyspepsia	Ever smoker	90	Death w/o cancer	0.086	(0.069, 0.106)
Women	Dysphagia	Never smoker	30	Gyanecological	0.002	(0.001, 0.005)
Women	Dysphagia	Never smoker	30	Death w/o cancer	0.001	(<0.001, 0.002)
Women	Dysphagia	Never smoker	40	Gyanecological	0.001	(<0.001, 0.002)
Women	Dysphagia	Never smoker	40	Upper GI	0.002	(0.001, 0.003)
Women	Dysphagia	Never smoker	40	Other cancers	0.001	(<0.001, 0.002)
Women	Dysphagia	Never smoker	40	Death w/o cancer	0.002	(<0.001, 0.005)
Women	Dysphagia	Never smoker	50	Breast	0.001	(<0.001, 0.002)
Women	Dysphagia	Never smoker	50	Upper GI	0.006	(0.003, 0.013)
Women	Dysphagia	Never smoker	50	Other cancers	0.003	(<0.001, 0.007)
Women	Dysphagia	Never smoker	50	Death w/o cancer	0.005	(0.002, 0.012)
Women	Dysphagia	Never smoker	60	Breast	0.003	(0.001, 0.005)
Women	Dysphagia	Never smoker	60	Gyanecological	0.001	(<0.001, 0.003)
Women	Dysphagia	Never smoker	60	Lung	0.001	(<0.001, 0.002)
Women	Dysphagia	Never smoker	60	Upper GI	0.014	(0.008, 0.025)
Women	Dysphagia	Never smoker	60	Lower GI	0.002	(<0.001, 0.003)
Women	Dysphagia	Never smoker	60	Haematological	0.001	(<0.001, 0.003)
Women	Dysphagia	Never smoker	60	Other cancers	0.004	(0.002, 0.008)
Women	Dysphagia	Never smoker	60	Death w/o cancer	0.009	(0.005, 0.018)
Women	Dysphagia	Never smoker	70	Breast	0.004	(0.002, 0.009)
Women	Dysphagia	Never smoker	70	Gyanecological	0.002	(0.001, 0.005)
Women	Dysphagia	Never smoker	70	Lung	0.002	(<0.001, 0.006)
Women	Dysphagia	Never smoker	70	Upper GI	0.035	(0.024, 0.051)
Women	Dysphagia	Never smoker	70	Lower GI	0.003	(0.001, 0.007)
Women	Dysphagia	Never smoker	70	Urological	0.002	(<0.001, 0.003)
Women	Dysphagia	Never smoker	70	Haematological	0.002	(<0.001, 0.005)
Women	Dysphagia	Never smoker	70	Other cancers	0.007	(0.003, 0.017)
Women	Dysphagia	Never smoker	70	Death w/o cancer	0.034	(0.025, 0.046)
Women	Dysphagia	Never smoker	80	Breast	0.005	(0.003, 0.008)
Women	Dysphagia	Never smoker	80	Gyanecological	0.002	(<0.001, 0.005)
Women	Dysphagia	Never smoker	80	Lung	0.003	(<0.001, 0.006)
Women	Dysphagia	Never smoker	80	Upper GI	0.050	(0.034, 0.073)
Women	Dysphagia	Never smoker	80	Lower GI	0.005	(0.002, 0.009)

Sex	Index symptom	Smoking status	Age (years)	Outcome event	Probability of outcome	(95% CI)
Women	Dysphagia	Never smoker	80	Urological	0.002	(<0.001, 0.004)
Women	Dysphagia	Never smoker	80	Haematological	0.003	(0.001, 0.006)
Women	Dysphagia	Never smoker	80	Other cancers	0.009	(0.004, 0.019)
Women	Dysphagia	Never smoker	80	Death w/o cancer	0.108	(0.088, 0.132)
Women	Dysphagia	Never smoker	90	Breast	0.005	(0.002, 0.011)
Women	Dysphagia	Never smoker	90	Gyanecological	0.001	(<0.001, 0.003)
Women	Dysphagia	Never smoker	90	Lung	0.002	(<0.001, 0.006)
Women	Dysphagia	Never smoker	90	Upper GI	0.049	(0.037, 0.065)
Women	Dysphagia	Never smoker	90	Lower GI	0.004	(0.001, 0.011)
Women	Dysphagia	Never smoker	90	Urological	0.002	(0.001, 0.004)
Women	Dysphagia	Never smoker	90	Haematological	0.003	(0.001, 0.006)
Women	Dysphagia	Never smoker	90	Other cancers	0.010	(0.005, 0.021)
Women	Dysphagia	Never smoker	90	Death w/o cancer	0.310	(0.282, 0.339)
Women	Dysphagia	Ever smoker	30	Gyanecological	0.003	(<0.001, 0.007)
Women	Dysphagia	Ever smoker	30	Death w/o cancer	0.001	(<0.001, 0.002)
Women	Dysphagia	Ever smoker	40	Upper GI	0.002	(<0.001, 0.004)
Women	Dysphagia	Ever smoker	40	Other cancers	0.001	(<0.001, 0.002)
Women	Dysphagia	Ever smoker	40	Death w/o cancer	0.002	(<0.001, 0.005)
Women	Dysphagia	Ever smoker	50	Breast	0.002	(<0.001, 0.003)
Women	Dysphagia	Ever smoker	50	Lung	0.002	(<0.001, 0.004)
Women	Dysphagia	Ever smoker	50	Upper GI	0.008	(0.004, 0.017)
Women	Dysphagia	Ever smoker	50	Other cancers	0.003	(0.001, 0.007)
Women	Dysphagia	Ever smoker	50	Death w/o cancer	0.005	(0.003, 0.008)
Women	Dysphagia	Ever smoker	60	Breast	0.003	(<0.001, 0.007)
Women	Dysphagia	Ever smoker	60	Gyanecological	0.001	(<0.001, 0.002)
Women	Dysphagia	Ever smoker	60	Lung	0.006	(0.002, 0.016)
Women	Dysphagia	Ever smoker	60	Upper GI	0.017	(0.010, 0.028)
Women	Dysphagia	Ever smoker	60	Lower GI	0.001	(<0.001, 0.003)
Women	Dysphagia	Ever smoker	60	Haematological	0.001	(<0.001, 0.003)
Women	Dysphagia	Ever smoker	60	Other cancers	0.004	(0.002, 0.011)
Women	Dysphagia	Ever smoker	60	Death w/o cancer	0.010	(0.006, 0.016)
Women	Dysphagia	Ever smoker	70	Breast	0.004	(0.002, 0.010)
Women	Dysphagia	Ever smoker	70	Gyanecological	0.002	(<0.001, 0.005)
Women	Dysphagia	Ever smoker	70	Lung	0.011	(0.006, 0.019)
Women	Dysphagia	Ever smoker	70	Upper GI	0.044	(0.030, 0.063)
Women	Dysphagia	Ever smoker	70	Lower GI	0.003	(0.001, 0.008)
Women	Dysphagia	Ever smoker	70	Urological	0.002	(0.001, 0.004)
Women	Dysphagia	Ever smoker	70	Haematological	0.002	(0.001, 0.004)
Women	Dysphagia	Ever smoker	70	Other cancers	0.007	(0.003, 0.015)
Women	Dysphagia	Ever smoker	70	Death w/o cancer	0.031	(0.023, 0.042)
Women	Dysphagia	Ever smoker	80	Breast	0.005	(0.002, 0.012)
Women	Dysphagia	Ever smoker	80	Gyanecological	0.002	(0.001, 0.005)
Women	Dysphagia	Ever smoker	80	Lung	0.013	(0.007, 0.024)
Women	Dysphagia	Ever smoker	80	Upper GI	0.060	(0.045, 0.080)
Women	Dysphagia	Ever smoker	80	Lower GI	0.004	(0.002, 0.008)
Women	Dysphagia	Ever smoker	80	Urological	0.003	(0.001, 0.005)
Women	Dysphagia	Ever smoker	80	Haematological	0.003	(0.001, 0.006)
Women	Dysphagia	Ever smoker	80	Other cancers	0.010	(0.005, 0.019)
Women	Dysphagia	Ever smoker	80	Death w/o cancer	0.104	(0.086, 0.125)
Women	Dysphagia	Ever smoker	90	Breast	0.005	(0.002, 0.015)
Women	Dysphagia	Ever smoker	90	Gyanecological	0.001	(<0.001, 0.002)
Women	Dysphagia	Ever smoker	90	Lung	0.010	(0.005, 0.019)
Women	Dysphagia	Ever smoker	90	Upper GI	0.060	(0.045, 0.079)
Women	Dysphagia	Ever smoker	90	Lower GI	0.004	(0.002, 0.009)
Women	Dysphagia	Ever smoker	90	Urological	0.003	(0.001, 0.006)
Women	Dysphagia	Ever smoker	90	Haematological	0.002	(0.001, 0.005)
Women	Dysphagia	Ever smoker	90	Other cancers	0.010	(0.005, 0.020)
Women	Dysphagia	Ever smoker	90	Death w/o cancer	0.297	(0.262, 0.335)
Women	Dyspnoea	Never smoker	30	Gyanecological	0.003	(0.001, 0.008)
Women	Dyspnoea	Never smoker	40	Gyanecological	0.001	(<0.001, 0.002)
Women	Dyspnoea	Never smoker	40	Death w/o cancer	0.001	(<0.001, 0.002)
Women	Dyspnoea	Never smoker	50	Breast	0.002	(<0.001, 0.003)

Sex	Index symptom	Smoking status	Age (years)	Outcome event	Probability of outcome	(95% CI)
Women	Dyspnoea	Never smoker	50	Other cancers	0.001	(<0.001, 0.002)
Women	Dyspnoea	Never smoker	50	Death w/o cancer	0.003	(0.002, 0.006)
Women	Dyspnoea	Never smoker	60	Breast	0.003	(0.001, 0.007)
Women	Dyspnoea	Never smoker	60	Gyanecological	0.002	(<0.001, 0.004)
Women	Dyspnoea	Never smoker	60	Lung	0.001	(<0.001, 0.004)
Women	Dyspnoea	Never smoker	60	Lower GI	0.002	(<0.001, 0.003)
Women	Dyspnoea	Never smoker	60	Haematological	0.001	(<0.001, 0.003)
Women	Dyspnoea	Never smoker	60	Other cancers	0.002	(<0.001, 0.004)
Women	Dyspnoea	Never smoker	60	Death w/o cancer	0.006	(0.003, 0.012)
Women	Dyspnoea	Never smoker	70	Breast	0.005	(0.002, 0.013)
Women	Dyspnoea	Never smoker	70	Gyanecological	0.003	(0.001, 0.009)
Women	Dyspnoea	Never smoker	70	Lung	0.003	(0.001, 0.005)
Women	Dyspnoea	Never smoker	70	Upper GI	0.002	(<0.001, 0.003)
Women	Dyspnoea	Never smoker	70	Lower GI	0.003	(0.001, 0.007)
Women	Dyspnoea	Never smoker	70	Urological	0.001	(<0.001, 0.003)
Women	Dyspnoea	Never smoker	70	Haematological	0.003	(0.001, 0.006)
Women	Dyspnoea	Never smoker	70	Other cancers	0.003	(0.002, 0.006)
Women	Dyspnoea	Never smoker	70	Death w/o cancer	0.021	(0.013, 0.033)
Women	Dyspnoea	Never smoker	80	Breast	0.007	(0.003, 0.017)
Women	Dyspnoea	Never smoker	80	Gyanecological	0.003	(<0.001, 0.006)
Women	Dyspnoea	Never smoker	80	Lung	0.003	(0.001, 0.009)
Women	Dyspnoea	Never smoker	80	Upper GI	0.002	(0.001, 0.004)
Women	Dyspnoea	Never smoker	80	Lower GI	0.004	(0.002, 0.012)
Women	Dyspnoea	Never smoker	80	Urological	0.002	(<0.001, 0.004)
Women	Dyspnoea	Never smoker	80	Haematological	0.004	(0.002, 0.007)
Women	Dyspnoea	Never smoker	80	Other cancers	0.004	(0.002, 0.008)
Women	Dyspnoea	Never smoker	80	Death w/o cancer	0.070	(0.054, 0.090)
Women	Dyspnoea	Never smoker	90	Breast	0.007	(0.004, 0.013)
Women	Dyspnoea	Never smoker	90	Gyanecological	0.002	(0.001, 0.004)
Women	Dyspnoea	Never smoker	90	Lung	0.003	(<0.001, 0.009)
Women	Dyspnoea	Never smoker	90	Upper GI	0.002	(<0.001, 0.005)
Women	Dyspnoea	Never smoker	90	Lower GI	0.005	(0.002, 0.011)
Women	Dyspnoea	Never smoker	90	Urological	0.002	(<0.001, 0.004)
Women	Dyspnoea	Never smoker	90	Haematological	0.004	(0.001, 0.009)
Women	Dyspnoea	Never smoker	90	Other cancers	0.005	(0.002, 0.017)
Women	Dyspnoea	Never smoker	90	Death w/o cancer	0.213	(0.195, 0.232)
Women	Dyspnoea	Ever smoker	30	Gyanecological	0.004	(0.002, 0.008)
Women	Dyspnoea	Ever smoker	40	Gyanecological	0.001	(<0.001, 0.003)
Women	Dyspnoea	Ever smoker	40	Death w/o cancer	0.001	(<0.001, 0.003)
Women	Dyspnoea	Ever smoker	50	Breast	0.002	(<0.001, 0.004)
Women	Dyspnoea	Ever smoker	50	Lung	0.002	(<0.001, 0.005)
Women	Dyspnoea	Ever smoker	50	Other cancers	0.001	(<0.001, 0.003)
Women	Dyspnoea	Ever smoker	50	Death w/o cancer	0.003	(0.001, 0.008)
Women	Dyspnoea	Ever smoker	60	Breast	0.003	(0.002, 0.008)
Women	Dyspnoea	Ever smoker	60	Gyanecological	0.001	(<0.001, 0.003)
Women	Dyspnoea	Ever smoker	60	Lung	0.007	(0.003, 0.017)
Women	Dyspnoea	Ever smoker	60	Lower GI	0.001	(<0.001, 0.003)
Women	Dyspnoea	Ever smoker	60	Haematological	0.001	(<0.001, 0.003)
Women	Dyspnoea	Ever smoker	60	Other cancers	0.002	(<0.001, 0.004)
Women	Dyspnoea	Ever smoker	60	Death w/o cancer	0.006	(0.003, 0.011)
Women	Dyspnoea	Ever smoker	70	Breast	0.005	(0.002, 0.013)
Women	Dyspnoea	Ever smoker	70	Gyanecological	0.003	(0.001, 0.008)
Women	Dyspnoea	Ever smoker	70	Lung	0.014	(0.008, 0.024)
Women	Dyspnoea	Ever smoker	70	Upper GI	0.002	(<0.001, 0.004)
Women	Dyspnoea	Ever smoker	70	Lower GI	0.003	(0.001, 0.008)
Women	Dyspnoea	Ever smoker	70	Urological	0.002	(<0.001, 0.003)
Women	Dyspnoea	Ever smoker	70	Haematological	0.003	(0.001, 0.006)
Women	Dyspnoea	Ever smoker	70	Other cancers	0.003	(0.001, 0.008)
Women	Dyspnoea	Ever smoker	70	Death w/o cancer	0.020	(0.013, 0.030)
Women	Dyspnoea	Ever smoker	80	Breast	0.007	(0.003, 0.015)
Women	Dyspnoea	Ever smoker	80	Gyanecological	0.003	(0.001, 0.007)
Women	Dyspnoea	Ever smoker	80	Lung	0.015	(0.009, 0.027)

Sex	Index symptom	Smoking status	Age (years)	Outcome event	Probability of outcome	(95% CI)
Women	Dyspnoea	Ever smoker	80	Upper GI	0.003	(0.001, 0.005)
Women	Dyspnoea	Ever smoker	80	Lower GI	0.004	(0.002, 0.007)
Women	Dyspnoea	Ever smoker	80	Urological	0.002	(0.001, 0.005)
Women	Dyspnoea	Ever smoker	80	Haematological	0.003	(0.001, 0.007)
Women	Dyspnoea	Ever smoker	80	Other cancers	0.005	(0.002, 0.012)
Women	Dyspnoea	Ever smoker	80	Death w/o cancer	0.066	(0.051, 0.084)
Women	Dyspnoea	Ever smoker	90	Breast	0.008	(0.003, 0.022)
Women	Dyspnoea	Ever smoker	90	Gyanecological	0.002	(<0.001, 0.005)
Women	Dyspnoea	Ever smoker	90	Lung	0.014	(0.008, 0.024)
Women	Dyspnoea	Ever smoker	90	Upper GI	0.003	(0.001, 0.006)
Women	Dyspnoea	Ever smoker	90	Lower GI	0.004	(0.002, 0.009)
Women	Dyspnoea	Ever smoker	90	Urological	0.002	(0.001, 0.006)
Women	Dyspnoea	Ever smoker	90	Haematological	0.004	(0.002, 0.008)
Women	Dyspnoea	Ever smoker	90	Other cancers	0.006	(0.004, 0.008)
Women	Dyspnoea	Ever smoker	90	Death w/o cancer	0.202	(0.178, 0.228)
Women	Fatigue	Never smoker	30	Gyanecological	0.003	(0.002, 0.007)
Women	Fatigue	Never smoker	40	Gyanecological	0.001	(<0.001, 0.003)
Women	Fatigue	Never smoker	50	Breast	0.002	(<0.001, 0.004)
Women	Fatigue	Never smoker	50	Gyanecological	0.001	(<0.001, 0.002)
Women	Fatigue	Never smoker	50	Other cancers	0.002	(<0.001, 0.003)
Women	Fatigue	Never smoker	50	Death w/o cancer	0.002	(<0.001, 0.005)
Women	Fatigue	Never smoker	60	Breast	0.004	(0.002, 0.007)
Women	Fatigue	Never smoker	60	Gyanecological	0.002	(<0.001, 0.004)
Women	Fatigue	Never smoker	60	Lower GI	0.002	(<0.001, 0.004)
Women	Fatigue	Never smoker	60	Haematological	0.002	(<0.001, 0.004)
Women	Fatigue	Never smoker	60	Other cancers	0.003	(<0.001, 0.007)
Women	Fatigue	Never smoker	60	Death w/o cancer	0.004	(0.002, 0.009)
Women	Fatigue	Never smoker	70	Breast	0.006	(0.003, 0.011)
Women	Fatigue	Never smoker	70	Gyanecological	0.003	(0.001, 0.009)
Women	Fatigue	Never smoker	70	Lung	0.002	(<0.001, 0.003)
Women	Fatigue	Never smoker	70	Upper GI	0.002	(0.001, 0.006)
Women	Fatigue	Never smoker	70	Lower GI	0.004	(0.002, 0.011)
Women	Fatigue	Never smoker	70	Urological	0.001	(<0.001, 0.002)
Women	Fatigue	Never smoker	70	Haematological	0.003	(0.002, 0.006)
Women	Fatigue	Never smoker	70	Other cancers	0.005	(0.002, 0.010)
Women	Fatigue	Never smoker	70	Death w/o cancer	0.014	(0.008, 0.023)
Women	Fatigue	Never smoker	80	Breast	0.007	(0.003, 0.016)
Women	Fatigue	Never smoker	80	Gyanecological	0.003	(0.002, 0.006)
Women	Fatigue	Never smoker	80	Lung	0.002	(<0.001, 0.003)
Women	Fatigue	Never smoker	80	Upper GI	0.003	(0.002, 0.005)
Women	Fatigue	Never smoker	80	Lower GI	0.006	(0.002, 0.015)
Women	Fatigue	Never smoker	80	Urological	0.002	(<0.001, 0.003)
Women	Fatigue	Never smoker	80	Haematological	0.004	(0.001, 0.014)
Women	Fatigue	Never smoker	80	Other cancers	0.007	(0.003, 0.016)
Women	Fatigue	Never smoker	80	Death w/o cancer	0.047	(0.034, 0.064)
Women	Fatigue	Never smoker	90	Breast	0.008	(0.003, 0.021)
Women	Fatigue	Never smoker	90	Gyanecological	0.002	(<0.001, 0.006)
Women	Fatigue	Never smoker	90	Lung	0.002	(<0.001, 0.003)
Women	Fatigue	Never smoker	90	Upper GI	0.003	(0.001, 0.007)
Women	Fatigue	Never smoker	90	Lower GI	0.006	(0.003, 0.012)
Women	Fatigue	Never smoker	90	Urological	0.002	(<0.001, 0.003)
Women	Fatigue	Never smoker	90	Haematological	0.005	(0.002, 0.010)
Women	Fatigue	Never smoker	90	Other cancers	0.007	(0.004, 0.014)
Women	Fatigue	Never smoker	90	Death w/o cancer	0.145	(0.125, 0.167)
Women	Fatigue	Ever smoker	30	Gyanecological	0.004	(0.002, 0.011)
Women	Fatigue	Ever smoker	40	Gyanecological	0.002	(<0.001, 0.003)
Women	Fatigue	Ever smoker	50	Breast	0.002	(0.001, 0.005)
Women	Fatigue	Ever smoker	50	Gyanecological	0.001	(<0.001, 0.002)
Women	Fatigue	Ever smoker	50	Lung	0.001	(<0.001, 0.002)
Women	Fatigue	Ever smoker	50	Other cancers	0.002	(<0.001, 0.004)
Women	Fatigue	Ever smoker	50	Death w/o cancer	0.002	(<0.001, 0.005)
Women	Fatigue	Ever smoker	60	Breast	0.004	(0.002, 0.009)

Sex	Index symptom	Smoking status	Age (years)	Outcome event	Probability of outcome	(95% CI)
Women	Fatigue	Ever smoker	60	Gyanecological	0.002	(0.001, 0.004)
Women	Fatigue	Ever smoker	60	Lung	0.004	(0.001, 0.010)
Women	Fatigue	Ever smoker	60	Upper GI	0.001	(<0.001, 0.002)
Women	Fatigue	Ever smoker	60	Lower GI	0.002	(<0.001, 0.005)
Women	Fatigue	Ever smoker	60	Haematological	0.002	(<0.001, 0.005)
Women	Fatigue	Ever smoker	60	Other cancers	0.003	(0.001, 0.006)
Women	Fatigue	Ever smoker	60	Death w/o cancer	0.004	(0.002, 0.008)
Women	Fatigue	Ever smoker	70	Breast	0.006	(0.002, 0.016)
Women	Fatigue	Ever smoker	70	Gyanecological	0.003	(0.001, 0.008)
Women	Fatigue	Ever smoker	70	Lung	0.007	(0.003, 0.015)
Women	Fatigue	Ever smoker	70	Upper GI	0.003	(0.002, 0.004)
Women	Fatigue	Ever smoker	70	Lower GI	0.004	(0.002, 0.010)
Women	Fatigue	Ever smoker	70	Urological	0.001	(<0.001, 0.003)
Women	Fatigue	Ever smoker	70	Haematological	0.004	(0.002, 0.008)
Women	Fatigue	Ever smoker	70	Other cancers	0.004	(0.002, 0.008)
Women	Fatigue	Ever smoker	70	Death w/o cancer	0.014	(0.008, 0.024)
Women	Fatigue	Ever smoker	80	Breast	0.007	(0.004, 0.015)
Women	Fatigue	Ever smoker	80	Gyanecological	0.003	(0.002, 0.007)
Women	Fatigue	Ever smoker	80	Lung	0.009	(0.004, 0.018)
Women	Fatigue	Ever smoker	80	Upper GI	0.003	(0.001, 0.009)
Women	Fatigue	Ever smoker	80	Lower GI	0.006	(0.004, 0.011)
Women	Fatigue	Ever smoker	80	Urological	0.002	(<0.001, 0.004)
Women	Fatigue	Ever smoker	80	Haematological	0.005	(0.002, 0.013)
Women	Fatigue	Ever smoker	80	Other cancers	0.007	(0.003, 0.015)
Women	Fatigue	Ever smoker	80	Death w/o cancer	0.046	(0.035, 0.060)
Women	Fatigue	Ever smoker	90	Breast	0.009	(0.004, 0.017)
Women	Fatigue	Ever smoker	90	Gyanecological	0.002	(0.001, 0.005)
Women	Fatigue	Ever smoker	90	Lung	0.007	(0.004, 0.014)
Women	Fatigue	Ever smoker	90	Upper GI	0.003	(0.002, 0.007)
Women	Fatigue	Ever smoker	90	Lower GI	0.006	(0.003, 0.012)
Women	Fatigue	Ever smoker	90	Urological	0.002	(<0.001, 0.005)
Women	Fatigue	Ever smoker	90	Haematological	0.005	(0.002, 0.012)
Women	Fatigue	Ever smoker	90	Other cancers	0.008	(0.004, 0.016)
Women	Fatigue	Ever smoker	90	Death w/o cancer	0.141	(0.122, 0.162)
Women	Haematuria	Never smoker	30	Gyanecological	0.014	(0.009, 0.023)
Women	Haematuria	Never smoker	40	Breast	0.001	(<0.001, 0.002)
Women	Haematuria	Never smoker	40	Gyanecological	0.006	(0.002, 0.016)
Women	Haematuria	Never smoker	40	Urological	0.003	(0.001, 0.008)
Women	Haematuria	Never smoker	50	Breast	0.002	(<0.001, 0.005)
Women	Haematuria	Never smoker	50	Gyanecological	0.005	(0.002, 0.009)
Women	Haematuria	Never smoker	50	Urological	0.010	(0.005, 0.020)
Women	Haematuria	Never smoker	50	Other cancers	0.002	(<0.001, 0.003)
Women	Haematuria	Never smoker	50	Death w/o cancer	0.002	(<0.001, 0.004)
Women	Haematuria	Never smoker	60	Breast	0.004	(0.002, 0.008)
Women	Haematuria	Never smoker	60	Gyanecological	0.007	(0.003, 0.016)
Women	Haematuria	Never smoker	60	Lower GI	0.001	(<0.001, 0.003)
Women	Haematuria	Never smoker	60	Urological	0.026	(0.018, 0.038)
Women	Haematuria	Never smoker	60	Other cancers	0.002	(0.001, 0.005)
Women	Haematuria	Never smoker	60	Death w/o cancer	0.004	(0.002, 0.007)
Women	Haematuria	Never smoker	70	Breast	0.006	(0.004, 0.009)
Women	Haematuria	Never smoker	70	Gyanecological	0.014	(0.008, 0.024)
Women	Haematuria	Never smoker	70	Lower GI	0.003	(0.001, 0.005)
Women	Haematuria	Never smoker	70	Urological	0.057	(0.043, 0.075)
Women	Haematuria	Never smoker	70	Haematological	0.002	(<0.001, 0.005)
Women	Haematuria	Never smoker	70	Other cancers	0.004	(0.002, 0.008)
Women	Haematuria	Never smoker	70	Death w/o cancer	0.014	(0.007, 0.027)
Women	Haematuria	Never smoker	80	Breast	0.008	(0.002, 0.028)
Women	Haematuria	Never smoker	80	Gyanecological	0.013	(0.008, 0.022)
Women	Haematuria	Never smoker	80	Lung	0.001	(<0.001, 0.003)
Women	Haematuria	Never smoker	80	Upper GI	0.001	(<0.001, 0.002)
Women	Haematuria	Never smoker	80	Lower GI	0.003	(0.001, 0.008)
Women	Haematuria	Never smoker	80	Urological	0.078	(0.058, 0.106)

Sex	Index symptom	Smoking status	Age (years)	Outcome event	Probability of outcome	(95% CI)
Women	Haematuria	Never smoker	80	Haematological	0.002	(0.001, 0.005)
Women	Haematuria	Never smoker	80	Other cancers	0.005	(0.003, 0.008)
Women	Haematuria	Never smoker	80	Death w/o cancer	0.045	(0.032, 0.063)
Women	Haematuria	Never smoker	90	Breast	0.009	(0.004, 0.018)
Women	Haematuria	Never smoker	90	Gyanecological	0.009	(0.003, 0.023)
Women	Haematuria	Never smoker	90	Upper GI	0.001	(<0.001, 0.003)
Women	Haematuria	Never smoker	90	Lower GI	0.003	(0.002, 0.007)
Women	Haematuria	Never smoker	90	Urological	0.091	(0.069, 0.120)
Women	Haematuria	Never smoker	90	Haematological	0.003	(0.001, 0.005)
Women	Haematuria	Never smoker	90	Other cancers	0.006	(0.002, 0.017)
Women	Haematuria	Never smoker	90	Death w/o cancer	0.137	(0.111, 0.167)
Women	Haematuria	Ever smoker	30	Gyanecological	0.016	(0.009, 0.026)
Women	Haematuria	Ever smoker	40	Gyanecological	0.007	(0.002, 0.019)
Women	Haematuria	Ever smoker	40	Urological	0.004	(0.002, 0.011)
Women	Haematuria	Ever smoker	50	Breast	0.002	(0.001, 0.005)
Women	Haematuria	Ever smoker	50	Gyanecological	0.005	(0.002, 0.012)
Women	Haematuria	Ever smoker	50	Urological	0.013	(0.007, 0.025)
Women	Haematuria	Ever smoker	50	Other cancers	0.001	(<0.001, 0.003)
Women	Haematuria	Ever smoker	50	Death w/o cancer	0.003	(0.001, 0.006)
Women	Haematuria	Ever smoker	60	Breast	0.004	(0.002, 0.009)
Women	Haematuria	Ever smoker	60	Gyanecological	0.008	(0.003, 0.019)
Women	Haematuria	Ever smoker	60	Lung	0.003	(0.001, 0.007)
Women	Haematuria	Ever smoker	60	Lower GI	0.001	(<0.001, 0.003)
Women	Haematuria	Ever smoker	60	Urological	0.033	(0.025, 0.044)
Women	Haematuria	Ever smoker	60	Haematological	0.001	(<0.001, 0.002)
Women	Haematuria	Ever smoker	60	Other cancers	0.002	(<0.001, 0.006)
Women	Haematuria	Ever smoker	60	Death w/o cancer	0.004	(0.002, 0.010)
Women	Haematuria	Ever smoker	70	Breast	0.006	(0.002, 0.017)
Women	Haematuria	Ever smoker	70	Gyanecological	0.014	(0.008, 0.023)
Women	Haematuria	Ever smoker	70	Lung	0.005	(0.002, 0.013)
Women	Haematuria	Ever smoker	70	Upper GI	0.001	(<0.001, 0.002)
Women	Haematuria	Ever smoker	70	Lower GI	0.002	(0.001, 0.005)
Women	Haematuria	Ever smoker	70	Urological	0.070	(0.053, 0.092)
Women	Haematuria	Ever smoker	70	Haematological	0.002	(<0.001, 0.006)
Women	Haematuria	Ever smoker	70	Other cancers	0.004	(0.002, 0.010)
Women	Haematuria	Ever smoker	70	Death w/o cancer	0.013	(0.008, 0.023)
Women	Haematuria	Ever smoker	80	Breast	0.008	(0.003, 0.022)
Women	Haematuria	Ever smoker	80	Gyanecological	0.013	(0.007, 0.023)
Women	Haematuria	Ever smoker	80	Lung	0.005	(0.002, 0.013)
Women	Haematuria	Ever smoker	80	Upper GI	0.002	(<0.001, 0.003)
Women	Haematuria	Ever smoker	80	Lower GI	0.003	(0.001, 0.007)
Women	Haematuria	Ever smoker	80	Urological	0.098	(0.082, 0.117)
Women	Haematuria	Ever smoker	80	Haematological	0.002	(0.001, 0.004)
Women	Haematuria	Ever smoker	80	Other cancers	0.005	(0.002, 0.015)
Women	Haematuria	Ever smoker	80	Death w/o cancer	0.043	(0.032, 0.059)
Women	Haematuria	Ever smoker	90	Breast	0.009	(0.005, 0.018)
Women	Haematuria	Ever smoker	90	Gyanecological	0.009	(0.005, 0.015)
Women	Haematuria	Ever smoker	90	Lung	0.005	(0.002, 0.014)
Women	Haematuria	Ever smoker	90	Upper GI	0.002	(<0.001, 0.004)
Women	Haematuria	Ever smoker	90	Lower GI	0.003	(0.001, 0.007)
Women	Haematuria	Ever smoker	90	Urological	0.112	(0.087, 0.143)
Women	Haematuria	Ever smoker	90	Haematological	0.003	(<0.001, 0.008)
Women	Haematuria	Ever smoker	90	Other cancers	0.007	(0.004, 0.013)
Women	Haematuria	Ever smoker	90	Death w/o cancer	0.130	(0.105, 0.159)
Women	Haemoptysis	Never smoker	30	Gyanecological	0.002	(<0.001, 0.006)
Women	Haemoptysis	Never smoker	40	Breast	0.001	(<0.001, 0.002)
Women	Haemoptysis	Never smoker	40	Death w/o cancer	0.001	(<0.001, 0.002)
Women	Haemoptysis	Never smoker	50	Breast	0.002	(0.001, 0.005)
Women	Haemoptysis	Never smoker	50	Lung	0.003	(<0.001, 0.010)
Women	Haemoptysis	Never smoker	50	Haematological	0.001	(<0.001, 0.003)
Women	Haemoptysis	Never smoker	50	Other cancers	0.002	(<0.001, 0.004)
Women	Haemoptysis	Never smoker	50	Death w/o cancer	0.003	(0.001, 0.007)

Sex	Index symptom	Smoking status	Age (years)	Outcome event	Probability of outcome	(95% CI)
Women	Haemoptysis	Never smoker	60	Breast	0.005	(0.002, 0.010)
Women	Haemoptysis	Never smoker	60	Gyanecological	0.001	(<0.001, 0.002)
Women	Haemoptysis	Never smoker	60	Lung	0.011	(0.005, 0.023)
Women	Haemoptysis	Never smoker	60	Lower GI	0.001	(<0.001, 0.003)
Women	Haemoptysis	Never smoker	60	Haematological	0.003	(0.001, 0.008)
Women	Haemoptysis	Never smoker	60	Other cancers	0.003	(0.001, 0.007)
Women	Haemoptysis	Never smoker	60	Death w/o cancer	0.005	(0.002, 0.012)
Women	Haemoptysis	Never smoker	70	Breast	0.007	(0.002, 0.019)
Women	Haemoptysis	Never smoker	70	Gyanecological	0.002	(<0.001, 0.006)
Women	Haemoptysis	Never smoker	70	Lung	0.021	(0.015, 0.030)
Women	Haemoptysis	Never smoker	70	Lower GI	0.003	(<0.001, 0.006)
Women	Haemoptysis	Never smoker	70	Haematological	0.005	(0.002, 0.015)
Women	Haemoptysis	Never smoker	70	Other cancers	0.005	(0.002, 0.012)
Women	Haemoptysis	Never smoker	70	Death w/o cancer	0.016	(0.010, 0.026)
Women	Haemoptysis	Never smoker	80	Breast	0.009	(0.004, 0.020)
Women	Haemoptysis	Never smoker	80	Gyanecological	0.002	(0.001, 0.004)
Women	Haemoptysis	Never smoker	80	Lung	0.024	(0.015, 0.041)
Women	Haemoptysis	Never smoker	80	Upper GI	0.002	(<0.001, 0.004)
Women	Haemoptysis	Never smoker	80	Lower GI	0.004	(0.001, 0.011)
Women	Haemoptysis	Never smoker	80	Haematological	0.007	(0.002, 0.022)
Women	Haemoptysis	Never smoker	80	Other cancers	0.007	(0.002, 0.024)
Women	Haemoptysis	Never smoker	80	Death w/o cancer	0.054	(0.036, 0.081)
Women	Haemoptysis	Never smoker	90	Breast	0.009	(0.005, 0.017)
Women	Haemoptysis	Never smoker	90	Gyanecological	0.001	(<0.001, 0.003)
Women	Haemoptysis	Never smoker	90	Lung	0.021	(0.013, 0.034)
Women	Haemoptysis	Never smoker	90	Upper GI	0.001	(<0.001, 0.003)
Women	Haemoptysis	Never smoker	90	Lower GI	0.004	(0.001, 0.012)
Women	Haemoptysis	Never smoker	90	Haematological	0.006	(0.003, 0.013)
Women	Haemoptysis	Never smoker	90	Other cancers	0.008	(0.004, 0.019)
Women	Haemoptysis	Never smoker	90	Death w/o cancer	0.165	(0.128, 0.211)
Women	Haemoptysis	Ever smoker	30	Gyanecological	0.002	(0.001, 0.004)
Women	Haemoptysis	Ever smoker	40	Breast	0.001	(<0.001, 0.002)
Women	Haemoptysis	Ever smoker	40	Gyanecological	0.001	(<0.001, 0.002)
Women	Haemoptysis	Ever smoker	40	Lung	0.002	(<0.001, 0.005)
Women	Haemoptysis	Ever smoker	40	Death w/o cancer	0.001	(<0.001, 0.002)
Women	Haemoptysis	Ever smoker	50	Breast	0.003	(0.001, 0.006)
Women	Haemoptysis	Ever smoker	50	Lung	0.016	(0.008, 0.029)
Women	Haemoptysis	Ever smoker	50	Haematological	0.002	(<0.001, 0.003)
Women	Haemoptysis	Ever smoker	50	Other cancers	0.002	(<0.001, 0.004)
Women	Haemoptysis	Ever smoker	50	Death w/o cancer	0.003	(<0.001, 0.007)
Women	Haemoptysis	Ever smoker	60	Breast	0.004	(0.001, 0.014)
Women	Haemoptysis	Ever smoker	60	Gyanecological	0.001	(<0.001, 0.002)
Women	Haemoptysis	Ever smoker	60	Lung	0.049	(0.037, 0.064)
Women	Haemoptysis	Ever smoker	60	Lower GI	0.001	(<0.001, 0.002)
Women	Haemoptysis	Ever smoker	60	Haematological	0.003	(0.001, 0.006)
Women	Haemoptysis	Ever smoker	60	Other cancers	0.003	(0.001, 0.009)
Women	Haemoptysis	Ever smoker	60	Death w/o cancer	0.005	(0.001, 0.014)
Women	Haemoptysis	Ever smoker	70	Breast	0.007	(0.002, 0.022)
Women	Haemoptysis	Ever smoker	70	Gyanecological	0.002	(0.001, 0.004)
Women	Haemoptysis	Ever smoker	70	Lung	0.094	(0.076, 0.116)
Women	Haemoptysis	Ever smoker	70	Upper GI	0.001	(<0.001, 0.003)
Women	Haemoptysis	Ever smoker	70	Lower GI	0.003	(0.001, 0.006)
Women	Haemoptysis	Ever smoker	70	Haematological	0.005	(0.001, 0.015)
Women	Haemoptysis	Ever smoker	70	Other cancers	0.005	(0.002, 0.013)
Women	Haemoptysis	Ever smoker	70	Death w/o cancer	0.015	(0.008, 0.028)
Women	Haemoptysis	Ever smoker	80	Breast	0.008	(0.003, 0.019)
Women	Haemoptysis	Ever smoker	80	Gyanecological	0.002	(<0.001, 0.004)
Women	Haemoptysis	Ever smoker	80	Lung	0.108	(0.080, 0.143)
Women	Haemoptysis	Ever smoker	80	Upper GI	0.002	(<0.001, 0.005)
Women	Haemoptysis	Ever smoker	80	Lower GI	0.003	(<0.001, 0.011)
Women	Haemoptysis	Ever smoker	80	Haematological	0.006	(0.002, 0.022)
Women	Haemoptysis	Ever smoker	80	Other cancers	0.007	(0.002, 0.019)

Sex	Index symptom	Smoking status	Age (years)	Outcome event	Probability of outcome	(95% CI)
Women	Haemoptysis	Ever smoker	80	Death w/o cancer	0.049	(0.036, 0.068)
Women	Haemoptysis	Ever smoker	90	Breast	0.009	(0.004, 0.020)
Women	Haemoptysis	Ever smoker	90	Gyanecological	0.001	(<0.001, 0.003)
Women	Haemoptysis	Ever smoker	90	Lung	0.098	(0.074, 0.129)
Women	Haemoptysis	Ever smoker	90	Upper GI	0.002	(<0.001, 0.004)
Women	Haemoptysis	Ever smoker	90	Lower GI	0.003	(0.001, 0.009)
Women	Haemoptysis	Ever smoker	90	Haematological	0.006	(0.003, 0.011)
Women	Haemoptysis	Ever smoker	90	Other cancers	0.008	(0.003, 0.020)
Women	Haemoptysis	Ever smoker	90	Death w/o cancer	0.151	(0.110, 0.205)
Women	Jaundice	Never smoker	30	Gyanecological	0.004	(0.001, 0.009)
Women	Jaundice	Never smoker	30	Upper GI	0.003	(0.002, 0.005)
Women	Jaundice	Never smoker	30	Other cancers	0.007	(0.003, 0.014)
Women	Jaundice	Never smoker	30	Death w/o cancer	0.002	(<0.001, 0.005)
Women	Jaundice	Never smoker	40	Breast	0.002	(0.001, 0.003)
Women	Jaundice	Never smoker	40	Gyanecological	0.001	(<0.001, 0.004)
Women	Jaundice	Never smoker	40	Upper GI	0.012	(0.006, 0.027)
Women	Jaundice	Never smoker	40	Other cancers	0.010	(0.005, 0.019)
Women	Jaundice	Never smoker	40	Death w/o cancer	0.004	(0.002, 0.009)
Women	Jaundice	Never smoker	50	Breast	0.004	(0.002, 0.010)
Women	Jaundice	Never smoker	50	Upper GI	0.044	(0.028, 0.068)
Women	Jaundice	Never smoker	50	Other cancers	0.023	(0.016, 0.034)
Women	Jaundice	Never smoker	50	Death w/o cancer	0.008	(0.004, 0.015)
Women	Jaundice	Never smoker	60	Breast	0.006	(0.002, 0.018)
Women	Jaundice	Never smoker	60	Gyanecological	0.002	(<0.001, 0.006)
Women	Jaundice	Never smoker	60	Upper GI	0.101	(0.083, 0.123)
Women	Jaundice	Never smoker	60	Lower GI	0.003	(<0.001, 0.008)
Women	Jaundice	Never smoker	60	Urological	0.002	(<0.001, 0.004)
Women	Jaundice	Never smoker	60	Other cancers	0.035	(0.020, 0.063)
Women	Jaundice	Never smoker	60	Death w/o cancer	0.015	(0.009, 0.024)
Women	Jaundice	Never smoker	70	Breast	0.008	(0.004, 0.019)
Women	Jaundice	Never smoker	70	Gyanecological	0.002	(<0.001, 0.006)
Women	Jaundice	Never smoker	70	Lung	0.001	(<0.001, 0.003)
Women	Jaundice	Never smoker	70	Upper GI	0.231	(0.198, 0.268)
Women	Jaundice	Never smoker	70	Lower GI	0.005	(0.001, 0.014)
Women	Jaundice	Never smoker	70	Urological	0.004	(0.002, 0.009)
Women	Jaundice	Never smoker	70	Other cancers	0.055	(0.038, 0.080)
Women	Jaundice	Never smoker	70	Death w/o cancer	0.045	(0.029, 0.068)
Women	Jaundice	Never smoker	80	Breast	0.009	(0.004, 0.021)
Women	Jaundice	Never smoker	80	Gyanecological	0.002	(<0.001, 0.005)
Women	Jaundice	Never smoker	80	Lung	0.001	(<0.001, 0.003)
Women	Jaundice	Never smoker	80	Upper GI	0.296	(0.248, 0.350)
Women	Jaundice	Never smoker	80	Lower GI	0.006	(0.002, 0.018)
Women	Jaundice	Never smoker	80	Urological	0.005	(<0.001, 0.000)
Women	Jaundice	Never smoker	80	Haematological	0.001	(<0.001, 0.003)
Women	Jaundice	Never smoker	80	Other cancers	0.072	(0.050, 0.102)
Women	Jaundice	Never smoker	80	Death w/o cancer	0.129	(0.101, 0.164)
Women	Jaundice	Never smoker	90	Breast	0.008	(0.003, 0.022)
Women	Jaundice	Never smoker	90	Lung	0.001	(<0.001, 0.003)
Women	Jaundice	Never smoker	90	Upper GI	0.280	(0.237, 0.327)
Women	Jaundice	Never smoker	90	Lower GI	0.005	(0.001, 0.018)
Women	Jaundice	Never smoker	90	Urological	0.005	(0.002, 0.012)
Women	Jaundice	Never smoker	90	Haematological	0.001	(<0.001, 0.004)
Women	Jaundice	Never smoker	90	Other cancers	0.080	(0.057, 0.112)
Women	Jaundice	Never smoker	90	Death w/o cancer	0.336	(0.283, 0.392)
Women	Jaundice	Ever smoker	30	Gyanecological	0.004	(<0.001, 0.015)
Women	Jaundice	Ever smoker	30	Upper GI	0.003	(0.001, 0.009)
Women	Jaundice	Ever smoker	30	Other cancers	0.007	(0.003, 0.018)
Women	Jaundice	Ever smoker	30	Death w/o cancer	0.002	(<0.001, 0.005)
Women	Jaundice	Ever smoker	40	Breast	0.002	(<0.001, 0.004)
Women	Jaundice	Ever smoker	40	Gyanecological	0.002	(<0.001, 0.003)
Women	Jaundice	Ever smoker	40	Upper GI	0.015	(0.009, 0.026)
Women	Jaundice	Ever smoker	40	Other cancers	0.011	(0.005, 0.022)

Sex	Index symptom	Smoking status	Age (years)	Outcome event	Probability of outcome	(95% CI)
Women	Jaundice	Ever smoker	40	Death w/o cancer	0.004	(0.001, 0.011)
Women	Jaundice	Ever smoker	50	Breast	0.004	(0.002, 0.008)
Women	Jaundice	Ever smoker	50	Gyanecological	0.001	(<0.001, 0.002)
Women	Jaundice	Ever smoker	50	Upper GI	0.053	(0.041, 0.068)
Women	Jaundice	Ever smoker	50	Lower GI	0.001	(<0.001, 0.003)
Women	Jaundice	Ever smoker	50	Urological	0.001	(<0.001, 0.002)
Women	Jaundice	Ever smoker	50	Other cancers	0.024	(0.015, 0.039)
Women	Jaundice	Ever smoker	50	Death w/o cancer	0.009	(0.004, 0.017)
Women	Jaundice	Ever smoker	60	Breast	0.007	(0.002, 0.024)
Women	Jaundice	Ever smoker	60	Gyanecological	0.002	(<0.001, 0.004)
Women	Jaundice	Ever smoker	60	Lung	0.003	(<0.001, 0.009)
Women	Jaundice	Ever smoker	60	Upper GI	0.119	(0.091, 0.156)
Women	Jaundice	Ever smoker	60	Lower GI	0.002	(<0.001, 0.006)
Women	Jaundice	Ever smoker	60	Urological	0.003	(0.001, 0.007)
Women	Jaundice	Ever smoker	60	Other cancers	0.037	(0.023, 0.059)
Women	Jaundice	Ever smoker	60	Death w/o cancer	0.014	(0.008, 0.024)
Women	Jaundice	Ever smoker	70	Breast	0.008	(0.002, 0.029)
Women	Jaundice	Ever smoker	70	Gyanecological	0.002	(<0.001, 0.007)
Women	Jaundice	Ever smoker	70	Lung	0.006	(0.001, 0.032)
Women	Jaundice	Ever smoker	70	Upper GI	0.267	(0.220, 0.322)
Women	Jaundice	Ever smoker	70	Lower GI	0.005	(0.002, 0.012)
Women	Jaundice	Ever smoker	70	Urological	0.005	(0.002, 0.017)
Women	Jaundice	Ever smoker	70	Haematological	0.001	(<0.001, 0.003)
Women	Jaundice	Ever smoker	70	Other cancers	0.057	(0.039, 0.084)
Women	Jaundice	Ever smoker	70	Death w/o cancer	0.040	(0.029, 0.056)
Women	Jaundice	Ever smoker	80	Breast	0.009	(0.003, 0.026)
Women	Jaundice	Ever smoker	80	Gyanecological	0.002	(<0.001, 0.007)
Women	Jaundice	Ever smoker	80	Lung	0.006	(0.001, 0.030)
Women	Jaundice	Ever smoker	80	Upper GI	0.342	(0.307, 0.380)
Women	Jaundice	Ever smoker	80	Lower GI	0.005	(0.001, 0.020)
Women	Jaundice	Ever smoker	80	Urological	0.006	(0.002, 0.019)
Women	Jaundice	Ever smoker	80	Haematological	0.001	(<0.001, 0.002)
Women	Jaundice	Ever smoker	80	Other cancers	0.072	(0.045, 0.114)
Women	Jaundice	Ever smoker	80	Death w/o cancer	0.115	(0.093, 0.143)
Women	Jaundice	Ever smoker	90	Breast	0.008	(0.004, 0.017)
Women	Jaundice	Ever smoker	90	Lung	0.006	(0.002, 0.021)
Women	Jaundice	Ever smoker	90	Upper GI	0.325	(0.278, 0.376)
Women	Jaundice	Ever smoker	90	Lower GI	0.005	(0.001, 0.016)
Women	Jaundice	Ever smoker	90	Urological	0.006	(0.002, 0.021)
Women	Jaundice	Ever smoker	90	Haematological	0.001	(<0.001, 0.004)
Women	Jaundice	Ever smoker	90	Other cancers	0.083	(0.060, 0.113)
Women	Jaundice	Ever smoker	90	Death w/o cancer	0.304	(0.249, 0.365)
Women	Night sweats	Never smoker	30	Gyanecological	0.002	(0.001, 0.005)
Women	Night sweats	Never smoker	40	Breast	0.001	(<0.001, 0.002)
Women	Night sweats	Never smoker	50	Breast	0.002	(0.001, 0.004)
Women	Night sweats	Never smoker	50	Haematological	0.001	(<0.001, 0.003)
Women	Night sweats	Never smoker	60	Breast	0.005	(<0.001, 0.000)
Women	Night sweats	Never smoker	60	Gyanecological	0.001	(<0.001, 0.002)
Women	Night sweats	Never smoker	60	Lower GI	0.001	(<0.001, 0.003)
Women	Night sweats	Never smoker	60	Haematological	0.003	(0.001, 0.008)
Women	Night sweats	Never smoker	60	Other cancers	0.001	(<0.001, 0.002)
Women	Night sweats	Never smoker	60	Death w/o cancer	0.001	(<0.001, 0.003)
Women	Night sweats	Never smoker	70	Breast	0.007	(0.003, 0.014)
Women	Night sweats	Never smoker	70	Gyanecological	0.002	(0.001, 0.005)
Women	Night sweats	Never smoker	70	Lung	0.002	(<0.001, 0.003)
Women	Night sweats	Never smoker	70	Lower GI	0.003	(0.001, 0.006)
Women	Night sweats	Never smoker	70	Urological	0.002	(<0.001, 0.004)
Women	Night sweats	Never smoker	70	Haematological	0.006	(0.002, 0.017)
Women	Night sweats	Never smoker	70	Other cancers	0.002	(<0.001, 0.006)
Women	Night sweats	Never smoker	70	Death w/o cancer	0.005	(0.003, 0.010)
Women	Night sweats	Never smoker	80	Breast	0.009	(0.005, 0.017)
Women	Night sweats	Never smoker	80	Gyanecological	0.002	(<0.001, 0.004)

Sex	Index symptom	Smoking status	Age (years)	Outcome event	Probability of outcome	(95% CI)
Women	Night sweats	Never smoker	80	Lung	0.002	(<0.001, 0.004)
Women	Night sweats	Never smoker	80	Upper GI	0.001	(<0.001, 0.003)
Women	Night sweats	Never smoker	80	Lower GI	0.004	(0.002, 0.009)
Women	Night sweats	Never smoker	80	Urological	0.003	(0.001, 0.007)
Women	Night sweats	Never smoker	80	Haematological	0.008	(0.004, 0.018)
Women	Night sweats	Never smoker	80	Other cancers	0.004	(0.001, 0.010)
Women	Night sweats	Never smoker	80	Death w/o cancer	0.017	(0.008, 0.035)
Women	Night sweats	Never smoker	90	Breast	0.010	(0.004, 0.024)
Women	Night sweats	Never smoker	90	Gyanecological	0.001	(<0.001, 0.003)
Women	Night sweats	Never smoker	90	Lung	0.002	(<0.001, 0.004)
Women	Night sweats	Never smoker	90	Upper GI	0.001	(<0.001, 0.004)
Women	Night sweats	Never smoker	90	Lower GI	0.004	(0.002, 0.010)
Women	Night sweats	Never smoker	90	Urological	0.003	(0.001, 0.010)
Women	Night sweats	Never smoker	90	Haematological	0.008	(0.002, 0.033)
Women	Night sweats	Never smoker	90	Other cancers	0.004	(0.002, 0.009)
Women	Night sweats	Never smoker	90	Death w/o cancer	0.056	(0.030, 0.102)
Women	Night sweats	Ever smoker	30	Gyanecological	0.003	(0.001, 0.006)
Women	Night sweats	Ever smoker	50	Breast	0.002	(0.001, 0.005)
Women	Night sweats	Ever smoker	50	Lung	0.001	(<0.001, 0.003)
Women	Night sweats	Ever smoker	50	Haematological	0.001	(<0.001, 0.003)
Women	Night sweats	Ever smoker	50	Other cancers	0.001	(<0.001, 0.002)
Women	Night sweats	Ever smoker	60	Breast	0.004	(0.002, 0.012)
Women	Night sweats	Ever smoker	60	Gyanecological	0.001	(<0.001, 0.002)
Women	Night sweats	Ever smoker	60	Lung	0.004	(0.001, 0.013)
Women	Night sweats	Ever smoker	60	Lower GI	0.001	(<0.001, 0.002)
Women	Night sweats	Ever smoker	60	Urological	0.001	(<0.001, 0.002)
Women	Night sweats	Ever smoker	60	Haematological	0.003	(0.001, 0.008)
Women	Night sweats	Ever smoker	60	Other cancers	0.002	(<0.001, 0.003)
Women	Night sweats	Ever smoker	60	Death w/o cancer	0.001	(<0.001, 0.003)
Women	Night sweats	Ever smoker	70	Breast	0.007	(0.003, 0.019)
Women	Night sweats	Ever smoker	70	Gyanecological	0.002	(<0.001, 0.005)
Women	Night sweats	Ever smoker	70	Lung	0.008	(0.003, 0.024)
Women	Night sweats	Ever smoker	70	Upper GI	0.001	(<0.001, 0.002)
Women	Night sweats	Ever smoker	70	Lower GI	0.003	(0.001, 0.007)
Women	Night sweats	Ever smoker	70	Urological	0.002	(<0.001, 0.007)
Women	Night sweats	Ever smoker	70	Haematological	0.007	(0.003, 0.016)
Women	Night sweats	Ever smoker	70	Other cancers	0.003	(0.001, 0.006)
Women	Night sweats	Ever smoker	70	Death w/o cancer	0.005	(0.002, 0.015)
Women	Night sweats	Ever smoker	80	Breast	0.009	(0.004, 0.024)
Women	Night sweats	Ever smoker	80	Gyanecological	0.002	(<0.001, 0.004)
Women	Night sweats	Ever smoker	80	Lung	0.009	(0.003, 0.024)
Women	Night sweats	Ever smoker	80	Upper GI	0.002	(<0.001, 0.004)
Women	Night sweats	Ever smoker	80	Lower GI	0.004	(0.001, 0.012)
Women	Night sweats	Ever smoker	80	Urological	0.004	(0.002, 0.009)
Women	Night sweats	Ever smoker	80	Haematological	0.008	(0.004, 0.019)
Women	Night sweats	Ever smoker	80	Other cancers	0.004	(0.002, 0.007)
Women	Night sweats	Ever smoker	80	Death w/o cancer	0.017	(0.008, 0.039)
Women	Night sweats	Ever smoker	90	Breast	0.011	(0.006, 0.021)
Women	Night sweats	Ever smoker	90	Gyanecological	0.001	(<0.001, 0.003)
Women	Night sweats	Ever smoker	90	Lung	0.008	(0.002, 0.032)
Women	Night sweats	Ever smoker	90	Upper GI	0.002	(<0.001, 0.004)
Women	Night sweats	Ever smoker	90	Lower GI	0.004	(0.002, 0.008)
Women	Night sweats	Ever smoker	90	Urological	0.004	(0.001, 0.013)
Women	Night sweats	Ever smoker	90	Haematological	0.009	(0.004, 0.017)
Women	Night sweats	Ever smoker	90	Other cancers	0.005	(0.001, 0.016)
Women	Night sweats	Ever smoker	90	Death w/o cancer	0.054	(0.033, 0.087)
Women	Post-menopausal bleed	Never smoker	30	Gyanecological	0.145	(0.108, 0.190)
Women	Post-menopausal bleed	Never smoker	40	Gyanecological	0.068	(0.045, 0.101)
Women	Post-menopausal bleed	Never smoker	50	Breast	0.002	(<0.001, 0.004)
Women	Post-menopausal bleed	Never smoker	50	Gyanecological	0.046	(0.028, 0.077)
Women	Post-menopausal bleed	Never smoker	50	Other cancers	0.001	(<0.001, 0.002)
Women	Post-menopausal bleed	Never smoker	50	Death w/o cancer	0.001	(<0.001, 0.002)

Sex	Index symptom	Smoking status	Age (years)	Outcome event	Probability of outcome	(95% CI)
Women	Post-menopausal bleed	Never smoker	60	Breast	0.003	(0.001, 0.007)
Women	Post-menopausal bleed	Never smoker	60	Gyanecological	0.078	(0.060, 0.103)
Women	Post-menopausal bleed	Never smoker	60	Lower GI	0.001	(<0.001, 0.002)
Women	Post-menopausal bleed	Never smoker	60	Urological	0.002	(<0.001, 0.003)
Women	Post-menopausal bleed	Never smoker	60	Haematological	0.001	(<0.001, 0.002)
Women	Post-menopausal bleed	Never smoker	60	Other cancers	0.002	(<0.001, 0.005)
Women	Post-menopausal bleed	Never smoker	60	Death w/o cancer	0.002	(<0.001, 0.005)
Women	Post-menopausal bleed	Never smoker	70	Breast	0.005	(0.002, 0.012)
Women	Post-menopausal bleed	Never smoker	70	Gyanecological	0.136	(0.111, 0.164)
Women	Post-menopausal bleed	Never smoker	70	Upper GI	0.002	(<0.001, 0.004)
Women	Post-menopausal bleed	Never smoker	70	Lower GI	0.002	(0.001, 0.004)
Women	Post-menopausal bleed	Never smoker	70	Urological	0.003	(0.001, 0.009)
Women	Post-menopausal bleed	Never smoker	70	Haematological	0.002	(<0.001, 0.004)
Women	Post-menopausal bleed	Never smoker	70	Other cancers	0.003	(<0.001, 0.008)
Women	Post-menopausal bleed	Never smoker	70	Death w/o cancer	0.008	(0.004, 0.016)
Women	Post-menopausal bleed	Never smoker	80	Breast	0.006	(0.004, 0.011)
Women	Post-menopausal bleed	Never smoker	80	Gyanecological	0.130	(0.104, 0.161)
Women	Post-menopausal bleed	Never smoker	80	Upper GI	0.002	(0.001, 0.004)
Women	Post-menopausal bleed	Never smoker	80	Lower GI	0.003	(0.001, 0.008)
Women	Post-menopausal bleed	Never smoker	80	Urological	0.005	(0.002, 0.013)
Women	Post-menopausal bleed	Never smoker	80	Haematological	0.002	(<0.001, 0.004)
Women	Post-menopausal bleed	Never smoker	80	Other cancers	0.004	(0.001, 0.014)
Women	Post-menopausal bleed	Never smoker	80	Death w/o cancer	0.026	(0.018, 0.037)
Women	Post-menopausal bleed	Never smoker	90	Breast	0.007	(0.004, 0.014)
Women	Post-menopausal bleed	Never smoker	90	Gyanecological	0.096	(0.074, 0.124)
Women	Post-menopausal bleed	Never smoker	90	Upper GI	0.002	(0.001, 0.004)
Women	Post-menopausal bleed	Never smoker	90	Lower GI	0.004	(0.002, 0.009)
Women	Post-menopausal bleed	Never smoker	90	Urological	0.006	(0.002, 0.018)
Women	Post-menopausal bleed	Never smoker	90	Haematological	0.002	(<0.001, 0.005)
Women	Post-menopausal bleed	Never smoker	90	Other cancers	0.005	(0.002, 0.016)
Women	Post-menopausal bleed	Never smoker	90	Death w/o cancer	0.085	(0.066, 0.109)
Women	Post-menopausal bleed	Ever smoker	30	Gyanecological	0.153	(0.122, 0.191)
Women	Post-menopausal bleed	Ever smoker	40	Gyanecological	0.072	(0.048, 0.105)
Women	Post-menopausal bleed	Ever smoker	50	Breast	0.002	(0.001, 0.003)
Women	Post-menopausal bleed	Ever smoker	50	Gyanecological	0.049	(0.035, 0.068)
Women	Post-menopausal bleed	Ever smoker	50	Other cancers	0.001	(<0.001, 0.003)
Women	Post-menopausal bleed	Ever smoker	50	Death w/o cancer	0.001	(<0.001, 0.003)
Women	Post-menopausal bleed	Ever smoker	60	Breast	0.003	(0.002, 0.006)
Women	Post-menopausal bleed	Ever smoker	60	Gyanecological	0.083	(0.061, 0.113)
Women	Post-menopausal bleed	Ever smoker	60	Urological	0.002	(<0.001, 0.005)
Women	Post-menopausal bleed	Ever smoker	60	Other cancers	0.002	(<0.001, 0.004)
Women	Post-menopausal bleed	Ever smoker	60	Death w/o cancer	0.002	(0.001, 0.006)
Women	Post-menopausal bleed	Ever smoker	70	Breast	0.005	(0.003, 0.010)
Women	Post-menopausal bleed	Ever smoker	70	Gyanecological	0.144	(0.119, 0.175)
Women	Post-menopausal bleed	Ever smoker	70	Upper GI	0.002	(<0.001, 0.004)
Women	Post-menopausal bleed	Ever smoker	70	Lower GI	0.002	(<0.001, 0.006)
Women	Post-menopausal bleed	Ever smoker	70	Urological	0.004	(0.002, 0.011)
Women	Post-menopausal bleed	Ever smoker	70	Haematological	0.002	(<0.001, 0.004)
Women	Post-menopausal bleed	Ever smoker	70	Other cancers	0.003	(0.002, 0.007)
Women	Post-menopausal bleed	Ever smoker	70	Death w/o cancer	0.007	(0.003, 0.017)
Women	Post-menopausal bleed	Ever smoker	80	Breast	0.007	(0.003, 0.016)
Women	Post-menopausal bleed	Ever smoker	80	Gyanecological	0.139	(0.115, 0.167)
Women	Post-menopausal bleed	Ever smoker	80	Lung	0.001	(<0.001, 0.002)
Women	Post-menopausal bleed	Ever smoker	80	Upper GI	0.003	(0.001, 0.006)
Women	Post-menopausal bleed	Ever smoker	80	Lower GI	0.003	(0.001, 0.008)
Women	Post-menopausal bleed	Ever smoker	80	Urological	0.006	(0.003, 0.012)
Women	Post-menopausal bleed	Ever smoker	80	Haematological	0.002	(0.001, 0.004)
Women	Post-menopausal bleed	Ever smoker	80	Other cancers	0.005	(0.002, 0.011)
Women	Post-menopausal bleed	Ever smoker	80	Death w/o cancer	0.025	(0.017, 0.036)
Women	Post-menopausal bleed	Ever smoker	90	Breast	0.007	(0.003, 0.020)
Women	Post-menopausal bleed	Ever smoker	90	Gyanecological	0.102	(0.081, 0.128)
Women	Post-menopausal bleed	Ever smoker	90	Upper GI	0.003	(0.001, 0.005)

Sex	Index symptom	Smoking status	Age (years)	Outcome event	Probability of outcome	(95% CI)
Women	Post-menopausal bleed	Ever smoker	90	Lower GI	0.004	(0.001, 0.010)
Women	Post-menopausal bleed	Ever smoker	90	Urological	0.008	(0.003, 0.020)
Women	Post-menopausal bleed	Ever smoker	90	Haematological	0.002	(0.001, 0.004)
Women	Post-menopausal bleed	Ever smoker	90	Other cancers	0.006	(0.002, 0.015)
Women	Post-menopausal bleed	Ever smoker	90	Death w/o cancer	0.081	(0.059, 0.109)
Women	Rectal bleeding	Never smoker	30	Gyanecological	0.004	(0.002, 0.010)
Women	Rectal bleeding	Never smoker	40	Gyanecological	0.002	(0.001, 0.004)
Women	Rectal bleeding	Never smoker	40	Lower GI	0.002	(<0.001, 0.006)
Women	Rectal bleeding	Never smoker	40	Other cancers	0.001	(<0.001, 0.002)
Women	Rectal bleeding	Never smoker	50	Breast	0.002	(<0.001, 0.003)
Women	Rectal bleeding	Never smoker	50	Gyanecological	0.001	(<0.001, 0.002)
Women	Rectal bleeding	Never smoker	50	Lower GI	0.009	(0.004, 0.017)
Women	Rectal bleeding	Never smoker	50	Other cancers	0.003	(0.001, 0.006)
Women	Rectal bleeding	Never smoker	50	Death w/o cancer	0.003	(<0.001, 0.007)
Women	Rectal bleeding	Never smoker	60	Breast	0.003	(0.001, 0.010)
Women	Rectal bleeding	Never smoker	60	Gyanecological	0.002	(0.001, 0.004)
Women	Rectal bleeding	Never smoker	60	Lower GI	0.019	(0.013, 0.028)
Women	Rectal bleeding	Never smoker	60	Haematological	0.001	(<0.001, 0.003)
Women	Rectal bleeding	Never smoker	60	Other cancers	0.004	(0.001, 0.011)
Women	Rectal bleeding	Never smoker	60	Death w/o cancer	0.004	(0.001, 0.012)
Women	Rectal bleeding	Never smoker	70	Breast	0.005	(0.003, 0.011)
Women	Rectal bleeding	Never smoker	70	Gyanecological	0.004	(0.001, 0.009)
Women	Rectal bleeding	Never smoker	70	Upper GI	0.002	(<0.001, 0.003)
Women	Rectal bleeding	Never smoker	70	Lower GI	0.042	(0.031, 0.057)
Women	Rectal bleeding	Never smoker	70	Urological	0.001	(<0.001, 0.002)
Women	Rectal bleeding	Never smoker	70	Haematological	0.002	(<0.001, 0.006)
Women	Rectal bleeding	Never smoker	70	Other cancers	0.007	(0.004, 0.014)
Women	Rectal bleeding	Never smoker	70	Death w/o cancer	0.014	(0.009, 0.024)
Women	Rectal bleeding	Never smoker	80	Breast	0.006	(0.003, 0.015)
Women	Rectal bleeding	Never smoker	80	Gyanecological	0.003	(0.001, 0.009)
Women	Rectal bleeding	Never smoker	80	Upper GI	0.003	(0.002, 0.005)
Women	Rectal bleeding	Never smoker	80	Lower GI	0.057	(0.044, 0.074)
Women	Rectal bleeding	Never smoker	80	Urological	0.002	(<0.001, 0.005)
Women	Rectal bleeding	Never smoker	80	Haematological	0.003	(0.001, 0.008)
Women	Rectal bleeding	Never smoker	80	Other cancers	0.010	(0.005, 0.018)
Women	Rectal bleeding	Never smoker	80	Death w/o cancer	0.049	(0.036, 0.067)
Women	Rectal bleeding	Never smoker	90	Breast	0.007	(0.004, 0.012)
Women	Rectal bleeding	Never smoker	90	Gyanecological	0.002	(<0.001, 0.006)
Women	Rectal bleeding	Never smoker	90	Upper GI	0.003	(0.001, 0.007)
Women	Rectal bleeding	Never smoker	90	Lower GI	0.059	(0.044, 0.079)
Women	Rectal bleeding	Never smoker	90	Urological	0.002	(<0.001, 0.004)
Women	Rectal bleeding	Never smoker	90	Haematological	0.003	(0.001, 0.007)
Women	Rectal bleeding	Never smoker	90	Other cancers	0.012	(0.006, 0.023)
Women	Rectal bleeding	Never smoker	90	Death w/o cancer	0.147	(0.128, 0.168)
Women	Rectal bleeding	Ever smoker	30	Gyanecological	0.004	(0.002, 0.012)
Women	Rectal bleeding	Ever smoker	40	Gyanecological	0.002	(0.001, 0.003)
Women	Rectal bleeding	Ever smoker	40	Lower GI	0.002	(0.001, 0.004)
Women	Rectal bleeding	Ever smoker	40	Other cancers	0.001	(<0.001, 0.002)
Women	Rectal bleeding	Ever smoker	50	Breast	0.002	(<0.001, 0.004)
Women	Rectal bleeding	Ever smoker	50	Gyanecological	0.001	(<0.001, 0.002)
Women	Rectal bleeding	Ever smoker	50	Lower GI	0.008	(0.004, 0.013)
Women	Rectal bleeding	Ever smoker	50	Other cancers	0.003	(0.001, 0.006)
Women	Rectal bleeding	Ever smoker	50	Death w/o cancer	0.002	(<0.001, 0.005)
Women	Rectal bleeding	Ever smoker	60	Breast	0.004	(0.002, 0.008)
Women	Rectal bleeding	Ever smoker	60	Gyanecological	0.002	(0.001, 0.004)
Women	Rectal bleeding	Ever smoker	60	Lung	0.001	(<0.001, 0.003)
Women	Rectal bleeding	Ever smoker	60	Upper GI	0.001	(<0.001, 0.002)
Women	Rectal bleeding	Ever smoker	60	Lower GI	0.018	(0.011, 0.028)
Women	Rectal bleeding	Ever smoker	60	Haematological	0.001	(<0.001, 0.003)
Women	Rectal bleeding	Ever smoker	60	Other cancers	0.004	(0.002, 0.011)
Women	Rectal bleeding	Ever smoker	60	Death w/o cancer	0.004	(0.002, 0.011)
Women	Rectal bleeding	Ever smoker	70	Breast	0.006	(0.002, 0.015)

Sex	Index symptom	Smoking status	Age (years)	Outcome event	Probability of outcome	(95% CI)
Women	Rectal bleeding	Ever smoker	70	Gyanecological	0.004	(0.001, 0.010)
Women	Rectal bleeding	Ever smoker	70	Lung	0.002	(<0.001, 0.007)
Women	Rectal bleeding	Ever smoker	70	Upper GI	0.003	(0.001, 0.006)
Women	Rectal bleeding	Ever smoker	70	Lower GI	0.040	(0.028, 0.058)
Women	Rectal bleeding	Ever smoker	70	Urological	0.002	(<0.001, 0.004)
Women	Rectal bleeding	Ever smoker	70	Haematological	0.003	(0.001, 0.005)
Women	Rectal bleeding	Ever smoker	70	Other cancers	0.007	(0.004, 0.015)
Women	Rectal bleeding	Ever smoker	70	Death w/o cancer	0.014	(0.009, 0.021)
Women	Rectal bleeding	Ever smoker	80	Breast	0.006	(0.003, 0.014)
Women	Rectal bleeding	Ever smoker	80	Gyanecological	0.003	(0.002, 0.006)
Women	Rectal bleeding	Ever smoker	80	Lung	0.003	(0.001, 0.007)
Women	Rectal bleeding	Ever smoker	80	Upper GI	0.004	(0.001, 0.009)
Women	Rectal bleeding	Ever smoker	80	Lower GI	0.056	(0.041, 0.076)
Women	Rectal bleeding	Ever smoker	80	Urological	0.002	(<0.001, 0.006)
Women	Rectal bleeding	Ever smoker	80	Haematological	0.003	(0.001, 0.007)
Women	Rectal bleeding	Ever smoker	80	Other cancers	0.010	(0.005, 0.021)
Women	Rectal bleeding	Ever smoker	80	Death w/o cancer	0.047	(0.031, 0.070)
Women	Rectal bleeding	Ever smoker	90	Breast	0.008	(0.005, 0.014)
Women	Rectal bleeding	Ever smoker	90	Gyanecological	0.002	(0.001, 0.005)
Women	Rectal bleeding	Ever smoker	90	Lung	0.003	(0.001, 0.006)
Women	Rectal bleeding	Ever smoker	90	Upper GI	0.003	(0.002, 0.007)
Women	Rectal bleeding	Ever smoker	90	Lower GI	0.059	(0.043, 0.080)
Women	Rectal bleeding	Ever smoker	90	Urological	0.003	(0.001, 0.005)
Women	Rectal bleeding	Ever smoker	90	Haematological	0.003	(0.001, 0.007)
Women	Rectal bleeding	Ever smoker	90	Other cancers	0.012	(0.007, 0.022)
Women	Rectal bleeding	Ever smoker	90	Death w/o cancer	0.146	(0.124, 0.170)
Women	Weight loss	Never smoker	30	Gyanecological	0.006	(0.003, 0.011)
Women	Weight loss	Never smoker	40	Gyanecological	0.003	(0.001, 0.006)
Women	Weight loss	Never smoker	40	Other cancers	0.001	(<0.001, 0.003)
Women	Weight loss	Never smoker	40	Death w/o cancer	0.002	(<0.001, 0.004)
Women	Weight loss	Never smoker	50	Breast	0.002	(<0.001, 0.004)
Women	Weight loss	Never smoker	50	Gyanecological	0.002	(<0.001, 0.003)
Women	Weight loss	Never smoker	50	Upper GI	0.002	(<0.001, 0.004)
Women	Weight loss	Never smoker	50	Lower GI	0.002	(<0.001, 0.004)
Women	Weight loss	Never smoker	50	Haematological	0.002	(<0.001, 0.003)
Women	Weight loss	Never smoker	50	Other cancers	0.002	(<0.001, 0.006)
Women	Weight loss	Never smoker	50	Death w/o cancer	0.004	(0.002, 0.008)
Women	Weight loss	Never smoker	60	Breast	0.003	(0.001, 0.009)
Women	Weight loss	Never smoker	60	Gyanecological	0.003	(0.001, 0.006)
Women	Weight loss	Never smoker	60	Lung	0.002	(<0.001, 0.006)
Women	Weight loss	Never smoker	60	Upper GI	0.005	(0.002, 0.013)
Women	Weight loss	Never smoker	60	Lower GI	0.004	(0.001, 0.010)
Women	Weight loss	Never smoker	60	Urological	0.001	(<0.001, 0.002)
Women	Weight loss	Never smoker	60	Haematological	0.003	(0.001, 0.008)
Women	Weight loss	Never smoker	60	Other cancers	0.004	(0.001, 0.011)
Women	Weight loss	Never smoker	60	Death w/o cancer	0.007	(0.004, 0.012)
Women	Weight loss	Never smoker	70	Breast	0.005	(0.002, 0.010)
Women	Weight loss	Never smoker	70	Gyanecological	0.005	(0.002, 0.012)
Women	Weight loss	Never smoker	70	Lung	0.004	(0.001, 0.011)
Women	Weight loss	Never smoker	70	Upper GI	0.011	(0.005, 0.026)
Women	Weight loss	Never smoker	70	Lower GI	0.007	(0.004, 0.014)
Women	Weight loss	Never smoker	70	Urological	0.002	(0.001, 0.005)
Women	Weight loss	Never smoker	70	Haematological	0.006	(0.003, 0.014)
Women	Weight loss	Never smoker	70	Other cancers	0.007	(0.003, 0.014)
Women	Weight loss	Never smoker	70	Death w/o cancer	0.026	(0.019, 0.035)
Women	Weight loss	Never smoker	80	Breast	0.007	(0.003, 0.018)
Women	Weight loss	Never smoker	80	Gyanecological	0.005	(0.001, 0.015)
Women	Weight loss	Never smoker	80	Lung	0.005	(0.002, 0.014)
Women	Weight loss	Never smoker	80	Upper GI	0.015	(0.009, 0.026)
Women	Weight loss	Never smoker	80	Lower GI	0.011	(0.006, 0.020)
Women	Weight loss	Never smoker	80	Urological	0.003	(0.001, 0.009)
Women	Weight loss	Never smoker	80	Haematological	0.008	(0.004, 0.014)

Sex	Index symptom	Smoking status	Age (years)	Outcome event	Probability of outcome	(95% CI)
Women	Weight loss	Never smoker	80	Other cancers	0.010	(0.005, 0.017)
Women	Weight loss	Never smoker	80	Death w/o cancer	0.084	(0.064, 0.110)
Women	Weight loss	Never smoker	90	Breast	0.008	(0.004, 0.014)
Women	Weight loss	Never smoker	90	Gyanecological	0.003	(0.001, 0.009)
Women	Weight loss	Never smoker	90	Lung	0.004	(0.002, 0.010)
Women	Weight loss	Never smoker	90	Upper GI	0.015	(0.008, 0.028)
Women	Weight loss	Never smoker	90	Lower GI	0.011	(0.007, 0.018)
Women	Weight loss	Never smoker	90	Urological	0.003	(0.001, 0.008)
Women	Weight loss	Never smoker	90	Haematological	0.008	(0.004, 0.015)
Women	Weight loss	Never smoker	90	Other cancers	0.011	(0.006, 0.022)
Women	Weight loss	Never smoker	90	Death w/o cancer	0.248	(0.224, 0.274)
Women	Weight loss	Ever smoker	30	Gyanecological	0.006	(0.003, 0.011)
Women	Weight loss	Ever smoker	30	Death w/o cancer	0.001	(<0.001, 0.002)
Women	Weight loss	Ever smoker	40	Gyanecological	0.003	(0.001, 0.007)
Women	Weight loss	Ever smoker	40	Other cancers	0.001	(<0.001, 0.002)
Women	Weight loss	Ever smoker	40	Death w/o cancer	0.002	(<0.001, 0.003)
Women	Weight loss	Ever smoker	50	Breast	0.002	(<0.001, 0.004)
Women	Weight loss	Ever smoker	50	Gyanecological	0.002	(<0.001, 0.003)
Women	Weight loss	Ever smoker	50	Lung	0.003	(0.002, 0.007)
Women	Weight loss	Ever smoker	50	Upper GI	0.002	(<0.001, 0.005)
Women	Weight loss	Ever smoker	50	Lower GI	0.001	(<0.001, 0.003)
Women	Weight loss	Ever smoker	50	Haematological	0.001	(<0.001, 0.002)
Women	Weight loss	Ever smoker	50	Other cancers	0.003	(0.001, 0.005)
Women	Weight loss	Ever smoker	50	Death w/o cancer	0.004	(0.002, 0.009)
Women	Weight loss	Ever smoker	60	Breast	0.004	(0.002, 0.007)
Women	Weight loss	Ever smoker	60	Gyanecological	0.003	(0.001, 0.008)
Women	Weight loss	Ever smoker	60	Lung	0.010	(0.005, 0.022)
Women	Weight loss	Ever smoker	60	Upper GI	0.006	(0.002, 0.012)
Women	Weight loss	Ever smoker	60	Lower GI	0.003	(0.001, 0.008)
Women	Weight loss	Ever smoker	60	Urological	0.001	(<0.001, 0.003)
Women	Weight loss	Ever smoker	60	Haematological	0.003	(0.001, 0.008)
Women	Weight loss	Ever smoker	60	Other cancers	0.005	(0.002, 0.011)
Women	Weight loss	Ever smoker	60	Death w/o cancer	0.007	(0.004, 0.012)
Women	Weight loss	Ever smoker	70	Breast	0.005	(0.002, 0.013)
Women	Weight loss	Ever smoker	70	Gyanecological	0.006	(0.003, 0.010)
Women	Weight loss	Ever smoker	70	Lung	0.020	(0.014, 0.028)
Women	Weight loss	Ever smoker	70	Upper GI	0.013	(0.008, 0.022)
Women	Weight loss	Ever smoker	70	Lower GI	0.007	(0.004, 0.012)
Women	Weight loss	Ever smoker	70	Urological	0.003	(0.001, 0.008)
Women	Weight loss	Ever smoker	70	Haematological	0.006	(0.002, 0.018)
Women	Weight loss	Ever smoker	70	Other cancers	0.008	(0.003, 0.016)
Women	Weight loss	Ever smoker	70	Death w/o cancer	0.024	(0.016, 0.036)
Women	Weight loss	Ever smoker	80	Breast	0.007	(0.003, 0.017)
Women	Weight loss	Ever smoker	80	Gyanecological	0.005	(0.003, 0.008)
Women	Weight loss	Ever smoker	80	Lung	0.023	(0.013, 0.040)
Women	Weight loss	Ever smoker	80	Upper GI	0.019	(0.012, 0.032)
Women	Weight loss	Ever smoker	80	Lower GI	0.010	(0.005, 0.020)
Women	Weight loss	Ever smoker	80	Urological	0.004	(0.002, 0.008)
Women	Weight loss	Ever smoker	80	Haematological	0.007	(0.004, 0.016)
Women	Weight loss	Ever smoker	80	Other cancers	0.009	(0.005, 0.017)
Women	Weight loss	Ever smoker	80	Death w/o cancer	0.080	(0.064, 0.101)
Women	Weight loss	Ever smoker	90	Breast	0.008	(0.004, 0.014)
Women	Weight loss	Ever smoker	90	Gyanecological	0.003	(0.001, 0.008)
Women	Weight loss	Ever smoker	90	Lung	0.021	(0.012, 0.038)
Women	Weight loss	Ever smoker	90	Upper GI	0.019	(0.009, 0.039)
Women	Weight loss	Ever smoker	90	Lower GI	0.011	(0.005, 0.022)
Women	Weight loss	Ever smoker	90	Urological	0.004	(<0.001, 0.000)
Women	Weight loss	Ever smoker	90	Haematological	0.008	(0.004, 0.018)
Women	Weight loss	Ever smoker	90	Other cancers	0.011	(0.007, 0.018)
Women	Weight loss	Ever smoker	90	Death w/o cancer	0.239	(0.209, 0.272)
Men	Abdominal pain	Never smoker	40	Death w/o cancer	0.002	(0.001, 0.003)
Men	Abdominal pain	Never smoker	50	Upper GI	0.002	(<0.001, 0.004)

Sex	Index symptom	Smoking status	Age (years)	Outcome event	Probability of outcome	(95% CI)
Men	Abdominal pain	Never smoker	50	Lower GI	0.003	(0.002, 0.007)
Men	Abdominal pain	Never smoker	50	Urological	0.001	(<0.001, 0.002)
Men	Abdominal pain	Never smoker	50	Haematological	0.001	(<0.001, 0.003)
Men	Abdominal pain	Never smoker	50	Other cancers	0.002	(0.001, 0.004)
Men	Abdominal pain	Never smoker	50	Death w/o cancer	0.003	(0.002, 0.007)
Men	Abdominal pain	Never smoker	60	Upper GI	0.006	(0.002, 0.015)
Men	Abdominal pain	Never smoker	60	Lower GI	0.008	(0.004, 0.015)
Men	Abdominal pain	Never smoker	60	Urological	0.003	(0.001, 0.008)
Men	Abdominal pain	Never smoker	60	Prostate	0.007	(0.003, 0.015)
Men	Abdominal pain	Never smoker	60	Haematological	0.003	(0.001, 0.006)
Men	Abdominal pain	Never smoker	60	Other cancers	0.003	(0.001, 0.008)
Men	Abdominal pain	Never smoker	60	Death w/o cancer	0.007	(0.002, 0.019)
Men	Abdominal pain	Never smoker	70	Lung	0.002	(<0.001, 0.004)
Men	Abdominal pain	Never smoker	70	Upper GI	0.011	(0.006, 0.019)
Men	Abdominal pain	Never smoker	70	Lower GI	0.019	(0.011, 0.032)
Men	Abdominal pain	Never smoker	70	Urological	0.005	(0.002, 0.013)
Men	Abdominal pain	Never smoker	70	Prostate	0.016	(0.008, 0.030)
Men	Abdominal pain	Never smoker	70	Haematological	0.006	(0.003, 0.013)
Men	Abdominal pain	Never smoker	70	Other cancers	0.006	(0.003, 0.013)
Men	Abdominal pain	Never smoker	70	Death w/o cancer	0.016	(0.009, 0.030)
Men	Abdominal pain	Never smoker	80	Lung	0.002	(<0.001, 0.007)
Men	Abdominal pain	Never smoker	80	Upper GI	0.014	(0.008, 0.022)
Men	Abdominal pain	Never smoker	80	Lower GI	0.024	(0.016, 0.034)
Men	Abdominal pain	Never smoker	80	Urological	0.006	(0.002, 0.018)
Men	Abdominal pain	Never smoker	80	Prostate	0.019	(0.013, 0.030)
Men	Abdominal pain	Never smoker	80	Haematological	0.007	(0.004, 0.014)
Men	Abdominal pain	Never smoker	80	Other cancers	0.007	(0.004, 0.014)
Men	Abdominal pain	Never smoker	80	Death w/o cancer	0.044	(0.034, 0.057)
Men	Abdominal pain	Never smoker	90	Lung	0.002	(0.001, 0.006)
Men	Abdominal pain	Never smoker	90	Upper GI	0.014	(0.009, 0.021)
Men	Abdominal pain	Never smoker	90	Lower GI	0.029	(0.019, 0.042)
Men	Abdominal pain	Never smoker	90	Urological	0.006	(0.002, 0.015)
Men	Abdominal pain	Never smoker	90	Prostate	0.022	(0.015, 0.032)
Men	Abdominal pain	Never smoker	90	Haematological	0.009	(0.005, 0.019)
Men	Abdominal pain	Never smoker	90	Other cancers	0.010	(0.006, 0.017)
Men	Abdominal pain	Never smoker	90	Death w/o cancer	0.129	(0.111, 0.149)
Men	Abdominal pain	Ever smoker	40	Death w/o cancer	0.002	(<0.001, 0.004)
Men	Abdominal pain	Ever smoker	50	Upper GI	0.003	(0.001, 0.007)
Men	Abdominal pain	Ever smoker	50	Lower GI	0.003	(0.001, 0.007)
Men	Abdominal pain	Ever smoker	50	Urological	0.001	(<0.001, 0.003)
Men	Abdominal pain	Ever smoker	50	Haematological	0.001	(<0.001, 0.002)
Men	Abdominal pain	Ever smoker	50	Other cancers	0.002	(0.001, 0.005)
Men	Abdominal pain	Ever smoker	50	Death w/o cancer	0.003	(0.001, 0.009)
Men	Abdominal pain	Ever smoker	60	Lung	0.003	(0.001, 0.007)
Men	Abdominal pain	Ever smoker	60	Upper GI	0.008	(0.004, 0.017)
Men	Abdominal pain	Ever smoker	60	Lower GI	0.009	(0.004, 0.018)
Men	Abdominal pain	Ever smoker	60	Urological	0.004	(0.002, 0.007)
Men	Abdominal pain	Ever smoker	60	Prostate	0.006	(0.003, 0.012)
Men	Abdominal pain	Ever smoker	60	Haematological	0.003	(0.001, 0.007)
Men	Abdominal pain	Ever smoker	60	Other cancers	0.004	(0.001, 0.010)
Men	Abdominal pain	Ever smoker	60	Death w/o cancer	0.007	(0.003, 0.017)
Men	Abdominal pain	Ever smoker	70	Lung	0.007	(0.003, 0.016)
Men	Abdominal pain	Ever smoker	70	Upper GI	0.014	(0.010, 0.021)
Men	Abdominal pain	Ever smoker	70	Lower GI	0.019	(0.013, 0.028)
Men	Abdominal pain	Ever smoker	70	Urological	0.007	(0.002, 0.022)
Men	Abdominal pain	Ever smoker	70	Prostate	0.014	(0.009, 0.024)
Men	Abdominal pain	Ever smoker	70	Haematological	0.006	(0.002, 0.012)
Men	Abdominal pain	Ever smoker	70	Other cancers	0.007	(0.003, 0.014)
Men	Abdominal pain	Ever smoker	70	Death w/o cancer	0.017	(0.010, 0.027)
Men	Abdominal pain	Ever smoker	80	Lung	0.009	(0.004, 0.018)
Men	Abdominal pain	Ever smoker	80	Upper GI	0.018	(0.010, 0.033)
Men	Abdominal pain	Ever smoker	80	Lower GI	0.026	(0.016, 0.040)

Sex	Index symptom	Smoking status	Age (years)	Outcome event	Probability of outcome	(95% CI)
Men	Abdominal pain	Ever smoker	80	Urological	0.008	(0.003, 0.017)
Men	Abdominal pain	Ever smoker	80	Prostate	0.018	(0.010, 0.031)
Men	Abdominal pain	Ever smoker	80	Haematological	0.007	(0.003, 0.016)
Men	Abdominal pain	Ever smoker	80	Other cancers	0.009	(0.004, 0.019)
Men	Abdominal pain	Ever smoker	80	Death w/o cancer	0.047	(0.037, 0.060)
Men	Abdominal pain	Ever smoker	90	Lung	0.009	(0.004, 0.019)
Men	Abdominal pain	Ever smoker	90	Upper GI	0.019	(0.011, 0.033)
Men	Abdominal pain	Ever smoker	90	Lower GI	0.028	(0.018, 0.046)
Men	Abdominal pain	Ever smoker	90	Urological	0.008	(0.004, 0.017)
Men	Abdominal pain	Ever smoker	90	Prostate	0.019	(0.012, 0.029)
Men	Abdominal pain	Ever smoker	90	Haematological	0.009	(0.006, 0.014)
Men	Abdominal pain	Ever smoker	90	Other cancers	0.012	(0.007, 0.020)
Men	Abdominal pain	Ever smoker	90	Death w/o cancer	0.135	(0.115, 0.157)
Men	Abdominal bloating	Never smoker	40	Death w/o cancer	0.002	(<0.001, 0.004)
Men	Abdominal bloating	Never smoker	50	Upper GI	0.002	(<0.001, 0.004)
Men	Abdominal bloating	Never smoker	50	Lower GI	0.003	(0.001, 0.008)
Men	Abdominal bloating	Never smoker	50	Haematological	0.001	(<0.001, 0.003)
Men	Abdominal bloating	Never smoker	50	Other cancers	0.001	(<0.001, 0.003)
Men	Abdominal bloating	Never smoker	50	Death w/o cancer	0.002	(0.001, 0.006)
Men	Abdominal bloating	Never smoker	60	Upper GI	0.006	(0.003, 0.012)
Men	Abdominal bloating	Never smoker	60	Lower GI	0.008	(0.004, 0.017)
Men	Abdominal bloating	Never smoker	60	Urological	0.002	(0.001, 0.003)
Men	Abdominal bloating	Never smoker	60	Prostate	0.006	(0.002, 0.020)
Men	Abdominal bloating	Never smoker	60	Haematological	0.003	(<0.001, 0.008)
Men	Abdominal bloating	Never smoker	60	Other cancers	0.003	(0.001, 0.006)
Men	Abdominal bloating	Never smoker	60	Death w/o cancer	0.006	(0.003, 0.013)
Men	Abdominal bloating	Never smoker	70	Lung	0.002	(<0.001, 0.004)
Men	Abdominal bloating	Never smoker	70	Upper GI	0.011	(0.006, 0.020)
Men	Abdominal bloating	Never smoker	70	Lower GI	0.017	(0.010, 0.029)
Men	Abdominal bloating	Never smoker	70	Urological	0.003	(0.001, 0.009)
Men	Abdominal bloating	Never smoker	70	Prostate	0.014	(0.007, 0.028)
Men	Abdominal bloating	Never smoker	70	Haematological	0.005	(0.002, 0.011)
Men	Abdominal bloating	Never smoker	70	Other cancers	0.004	(0.002, 0.011)
Men	Abdominal bloating	Never smoker	70	Death w/o cancer	0.014	(0.008, 0.026)
Men	Abdominal bloating	Never smoker	80	Lung	0.002	(<0.001, 0.005)
Men	Abdominal bloating	Never smoker	80	Upper GI	0.013	(0.008, 0.022)
Men	Abdominal bloating	Never smoker	80	Lower GI	0.022	(0.014, 0.034)
Men	Abdominal bloating	Never smoker	80	Urological	0.004	(0.001, 0.010)
Men	Abdominal bloating	Never smoker	80	Prostate	0.018	(0.012, 0.027)
Men	Abdominal bloating	Never smoker	80	Haematological	0.007	(0.004, 0.014)
Men	Abdominal bloating	Never smoker	80	Other cancers	0.006	(0.003, 0.013)
Men	Abdominal bloating	Never smoker	80	Death w/o cancer	0.040	(0.028, 0.057)
Men	Abdominal bloating	Never smoker	90	Lung	0.002	(<0.001, 0.005)
Men	Abdominal bloating	Never smoker	90	Upper GI	0.013	(0.008, 0.022)
Men	Abdominal bloating	Never smoker	90	Lower GI	0.026	(0.017, 0.040)
Men	Abdominal bloating	Never smoker	90	Urological	0.004	(0.002, 0.008)
Men	Abdominal bloating	Never smoker	90	Prostate	0.019	(0.012, 0.032)
Men	Abdominal bloating	Never smoker	90	Haematological	0.009	(0.004, 0.020)
Men	Abdominal bloating	Never smoker	90	Other cancers	0.008	(0.004, 0.014)
Men	Abdominal bloating	Never smoker	90	Death w/o cancer	0.117	(0.094, 0.144)
Men	Abdominal bloating	Ever smoker	40	Death w/o cancer	0.002	(<0.001, 0.004)
Men	Abdominal bloating	Ever smoker	50	Upper GI	0.003	(0.001, 0.008)
Men	Abdominal bloating	Ever smoker	50	Lower GI	0.003	(0.001, 0.008)
Men	Abdominal bloating	Ever smoker	50	Other cancers	0.002	(<0.001, 0.003)
Men	Abdominal bloating	Ever smoker	50	Death w/o cancer	0.003	(0.001, 0.007)
Men	Abdominal bloating	Ever smoker	60	Lung	0.002	(<0.001, 0.005)
Men	Abdominal bloating	Ever smoker	60	Upper GI	0.008	(0.004, 0.015)
Men	Abdominal bloating	Ever smoker	60	Lower GI	0.008	(0.004, 0.015)
Men	Abdominal bloating	Ever smoker	60	Urological	0.002	(0.001, 0.005)
Men	Abdominal bloating	Ever smoker	60	Prostate	0.005	(0.002, 0.012)
Men	Abdominal bloating	Ever smoker	60	Haematological	0.003	(<0.001, 0.009)
Men	Abdominal bloating	Ever smoker	60	Other cancers	0.003	(0.001, 0.008)

Sex	Index symptom	Smoking status	Age (years)	Outcome event	Probability of outcome	(95% CI)
Men	Abdominal bloating	Ever smoker	60	Death w/o cancer	0.006	(0.003, 0.013)
Men	Abdominal bloating	Ever smoker	70	Lung	0.005	(0.003, 0.010)
Men	Abdominal bloating	Ever smoker	70	Upper GI	0.015	(0.008, 0.027)
Men	Abdominal bloating	Ever smoker	70	Lower GI	0.018	(0.012, 0.027)
Men	Abdominal bloating	Ever smoker	70	Urological	0.004	(0.002, 0.011)
Men	Abdominal bloating	Ever smoker	70	Prostate	0.013	(0.007, 0.023)
Men	Abdominal bloating	Ever smoker	70	Haematological	0.005	(0.002, 0.014)
Men	Abdominal bloating	Ever smoker	70	Other cancers	0.005	(0.003, 0.010)
Men	Abdominal bloating	Ever smoker	70	Death w/o cancer	0.015	(0.009, 0.025)
Men	Abdominal bloating	Ever smoker	80	Lung	0.006	(0.003, 0.012)
Men	Abdominal bloating	Ever smoker	80	Upper GI	0.017	(0.010, 0.030)
Men	Abdominal bloating	Ever smoker	80	Lower GI	0.022	(0.014, 0.036)
Men	Abdominal bloating	Ever smoker	80	Urological	0.005	(0.002, 0.011)
Men	Abdominal bloating	Ever smoker	80	Prostate	0.017	(0.009, 0.033)
Men	Abdominal bloating	Ever smoker	80	Haematological	0.007	(0.003, 0.017)
Men	Abdominal bloating	Ever smoker	80	Other cancers	0.008	(0.002, 0.026)
Men	Abdominal bloating	Ever smoker	80	Death w/o cancer	0.045	(0.034, 0.060)
Men	Abdominal bloating	Ever smoker	90	Lung	0.007	(0.003, 0.019)
Men	Abdominal bloating	Ever smoker	90	Upper GI	0.018	(0.009, 0.034)
Men	Abdominal bloating	Ever smoker	90	Lower GI	0.026	(0.016, 0.042)
Men	Abdominal bloating	Ever smoker	90	Urological	0.005	(0.002, 0.012)
Men	Abdominal bloating	Ever smoker	90	Prostate	0.018	(0.011, 0.029)
Men	Abdominal bloating	Ever smoker	90	Haematological	0.009	(0.005, 0.015)
Men	Abdominal bloating	Ever smoker	90	Other cancers	0.009	(0.005, 0.017)
Men	Abdominal bloating	Ever smoker	90	Death w/o cancer	0.128	(0.107, 0.152)
Men	Breast lump	Never smoker	30	Other cancers	0.003	(0.001, 0.009)
Men	Breast lump	Never smoker	40	Other cancers	0.005	(0.002, 0.009)
Men	Breast lump	Never smoker	50	Haematological	0.001	(<0.001, 0.003)
Men	Breast lump	Never smoker	50	Other cancers	0.010	(0.005, 0.018)
Men	Breast lump	Never smoker	50	Death w/o cancer	0.002	(<0.001, 0.003)
Men	Breast lump	Never smoker	60	Upper GI	0.001	(<0.001, 0.003)
Men	Breast lump	Never smoker	60	Prostate	0.004	(0.001, 0.010)
Men	Breast lump	Never smoker	60	Haematological	0.002	(0.001, 0.006)
Men	Breast lump	Never smoker	60	Other cancers	0.016	(0.009, 0.027)
Men	Breast lump	Never smoker	60	Death w/o cancer	0.003	(0.001, 0.008)
Men	Breast lump	Never smoker	70	Lung	0.002	(<0.001, 0.004)
Men	Breast lump	Never smoker	70	Upper GI	0.003	(0.001, 0.006)
Men	Breast lump	Never smoker	70	Urological	0.001	(<0.001, 0.003)
Men	Breast lump	Never smoker	70	Prostate	0.009	(0.003, 0.022)
Men	Breast lump	Never smoker	70	Haematological	0.005	(0.002, 0.010)
Men	Breast lump	Never smoker	70	Other cancers	0.027	(0.019, 0.038)
Men	Breast lump	Never smoker	70	Death w/o cancer	0.009	(0.005, 0.017)
Men	Breast lump	Never smoker	80	Lung	0.002	(<0.001, 0.006)
Men	Breast lump	Never smoker	80	Upper GI	0.004	(0.002, 0.010)
Men	Breast lump	Never smoker	80	Urological	0.002	(<0.001, 0.003)
Men	Breast lump	Never smoker	80	Prostate	0.011	(0.005, 0.026)
Men	Breast lump	Never smoker	80	Haematological	0.007	(0.003, 0.016)
Men	Breast lump	Never smoker	80	Other cancers	0.039	(0.027, 0.056)
Men	Breast lump	Never smoker	80	Death w/o cancer	0.026	(0.016, 0.041)
Men	Breast lump	Never smoker	90	Lung	0.003	(<0.001, 0.007)
Men	Breast lump	Never smoker	90	Upper GI	0.004	(0.002, 0.008)
Men	Breast lump	Never smoker	90	Urological	0.002	(<0.001, 0.004)
Men	Breast lump	Never smoker	90	Prostate	0.012	(0.005, 0.027)
Men	Breast lump	Never smoker	90	Haematological	0.008	(0.004, 0.015)
Men	Breast lump	Never smoker	90	Other cancers	0.051	(0.035, 0.072)
Men	Breast lump	Never smoker	90	Death w/o cancer	0.075	(0.049, 0.114)
Men	Breast lump	Ever smoker	30	Other cancers	0.004	(0.002, 0.007)
Men	Breast lump	Ever smoker	40	Other cancers	0.005	(0.003, 0.009)
Men	Breast lump	Ever smoker	40	Death w/o cancer	0.001	(<0.001, 0.002)
Men	Breast lump	Ever smoker	50	Haematological	0.001	(<0.001, 0.003)
Men	Breast lump	Ever smoker	50	Other cancers	0.011	(0.006, 0.021)
Men	Breast lump	Ever smoker	50	Death w/o cancer	0.002	(<0.001, 0.004)

Sex	Index symptom	Smoking status	Age (years)	Outcome event	Probability of outcome	(95% CI)
Men	Breast lump	Ever smoker	60	Lung	0.003	(0.001, 0.009)
Men	Breast lump	Ever smoker	60	Upper GI	0.002	(<0.001, 0.006)
Men	Breast lump	Ever smoker	60	Prostate	0.003	(0.001, 0.009)
Men	Breast lump	Ever smoker	60	Haematological	0.002	(<0.001, 0.007)
Men	Breast lump	Ever smoker	60	Other cancers	0.020	(0.013, 0.030)
Men	Breast lump	Ever smoker	60	Death w/o cancer	0.004	(0.002, 0.008)
Men	Breast lump	Ever smoker	70	Lung	0.007	(0.003, 0.017)
Men	Breast lump	Ever smoker	70	Upper GI	0.004	(0.001, 0.014)
Men	Breast lump	Ever smoker	70	Urological	0.002	(<0.001, 0.004)
Men	Breast lump	Ever smoker	70	Prostate	0.008	(0.003, 0.020)
Men	Breast lump	Ever smoker	70	Haematological	0.004	(0.001, 0.012)
Men	Breast lump	Ever smoker	70	Other cancers	0.033	(0.021, 0.051)
Men	Breast lump	Ever smoker	70	Death w/o cancer	0.010	(0.005, 0.020)
Men	Breast lump	Ever smoker	80	Lung	0.008	(0.003, 0.025)
Men	Breast lump	Ever smoker	80	Upper GI	0.005	(0.002, 0.012)
Men	Breast lump	Ever smoker	80	Urological	0.002	(<0.001, 0.006)
Men	Breast lump	Ever smoker	80	Prostate	0.010	(0.004, 0.023)
Men	Breast lump	Ever smoker	80	Haematological	0.006	(0.002, 0.016)
Men	Breast lump	Ever smoker	80	Other cancers	0.045	(0.031, 0.064)
Men	Breast lump	Ever smoker	80	Death w/o cancer	0.028	(0.015, 0.049)
Men	Breast lump	Ever smoker	90	Lung	0.010	(0.004, 0.026)
Men	Breast lump	Ever smoker	90	Upper GI	0.005	(0.001, 0.017)
Men	Breast lump	Ever smoker	90	Urological	0.002	(<0.001, 0.005)
Men	Breast lump	Ever smoker	90	Prostate	0.010	(0.003, 0.032)
Men	Breast lump	Ever smoker	90	Haematological	0.007	(0.002, 0.021)
Men	Breast lump	Ever smoker	90	Other cancers	0.061	(0.039, 0.093)
Men	Breast lump	Ever smoker	90	Death w/o cancer	0.081	(0.051, 0.126)
Men	Change in bowel habit	Never smoker	30	Lower GI	0.002	(<0.001, 0.005)
Men	Change in bowel habit	Never smoker	40	Lower GI	0.004	(0.002, 0.008)
Men	Change in bowel habit	Never smoker	40	Other cancers	0.001	(<0.001, 0.002)
Men	Change in bowel habit	Never smoker	50	Lower GI	0.014	(0.009, 0.023)
Men	Change in bowel habit	Never smoker	50	Other cancers	0.002	(<0.001, 0.005)
Men	Change in bowel habit	Never smoker	50	Death w/o cancer	0.002	(<0.001, 0.003)
Men	Change in bowel habit	Never smoker	60	Upper GI	0.002	(0.001, 0.004)
Men	Change in bowel habit	Never smoker	60	Lower GI	0.038	(0.025, 0.057)
Men	Change in bowel habit	Never smoker	60	Urological	0.002	(<0.001, 0.004)
Men	Change in bowel habit	Never smoker	60	Prostate	0.007	(0.004, 0.014)
Men	Change in bowel habit	Never smoker	60	Haematological	0.002	(0.001, 0.004)
Men	Change in bowel habit	Never smoker	60	Other cancers	0.003	(0.001, 0.008)
Men	Change in bowel habit	Never smoker	60	Death w/o cancer	0.003	(0.001, 0.009)
Men	Change in bowel habit	Never smoker	70	Lung	0.001	(<0.001, 0.003)
Men	Change in bowel habit	Never smoker	70	Upper GI	0.005	(0.002, 0.011)
Men	Change in bowel habit	Never smoker	70	Lower GI	0.080	(0.061, 0.104)
Men	Change in bowel habit	Never smoker	70	Urological	0.003	(0.001, 0.008)
Men	Change in bowel habit	Never smoker	70	Prostate	0.017	(0.011, 0.026)
Men	Change in bowel habit	Never smoker	70	Haematological	0.004	(0.002, 0.009)
Men	Change in bowel habit	Never smoker	70	Other cancers	0.006	(0.003, 0.012)
Men	Change in bowel habit	Never smoker	70	Death w/o cancer	0.009	(0.004, 0.021)
Men	Change in bowel habit	Never smoker	80	Lung	0.002	(<0.001, 0.003)
Men	Change in bowel habit	Never smoker	80	Upper GI	0.005	(0.002, 0.012)
Men	Change in bowel habit	Never smoker	80	Lower GI	0.107	(0.083, 0.138)
Men	Change in bowel habit	Never smoker	80	Urological	0.004	(0.002, 0.008)
Men	Change in bowel habit	Never smoker	80	Prostate	0.021	(0.012, 0.036)
Men	Change in bowel habit	Never smoker	80	Haematological	0.005	(0.002, 0.015)
Men	Change in bowel habit	Never smoker	80	Other cancers	0.008	(0.004, 0.015)
Men	Change in bowel habit	Never smoker	80	Death w/o cancer	0.022	(0.013, 0.037)
Men	Change in bowel habit	Never smoker	90	Lung	0.002	(<0.001, 0.004)
Men	Change in bowel habit	Never smoker	90	Upper GI	0.005	(0.002, 0.013)
Men	Change in bowel habit	Never smoker	90	Lower GI	0.125	(0.099, 0.156)
Men	Change in bowel habit	Never smoker	90	Urological	0.003	(0.001, 0.009)
Men	Change in bowel habit	Never smoker	90	Prostate	0.023	(0.015, 0.036)
Men	Change in bowel habit	Never smoker	90	Haematological	0.006	(0.002, 0.016)

Sex	Index symptom	Smoking status	Age (years)	Outcome event	Probability of outcome	(95% CI)
Men	Change in bowel habit	Never smoker	90	Other cancers	0.010	(0.004, 0.024)
Men	Change in bowel habit	Never smoker	90	Death w/o cancer	0.062	(0.047, 0.083)
Men	Change in bowel habit	Ever smoker	30	Lower GI	0.002	(0.001, 0.004)
Men	Change in bowel habit	Ever smoker	40	Lower GI	0.003	(0.001, 0.008)
Men	Change in bowel habit	Ever smoker	40	Other cancers	0.001	(<0.001, 0.002)
Men	Change in bowel habit	Ever smoker	50	Upper GI	0.001	(<0.001, 0.002)
Men	Change in bowel habit	Ever smoker	50	Lower GI	0.016	(0.010, 0.024)
Men	Change in bowel habit	Ever smoker	50	Other cancers	0.003	(0.001, 0.005)
Men	Change in bowel habit	Ever smoker	50	Death w/o cancer	0.002	(<0.001, 0.004)
Men	Change in bowel habit	Ever smoker	60	Lung	0.003	(0.001, 0.004)
Men	Change in bowel habit	Ever smoker	60	Upper GI	0.003	(0.001, 0.008)
Men	Change in bowel habit	Ever smoker	60	Lower GI	0.039	(0.029, 0.052)
Men	Change in bowel habit	Ever smoker	60	Urological	0.002	(<0.001, 0.005)
Men	Change in bowel habit	Ever smoker	60	Prostate	0.007	(0.003, 0.013)
Men	Change in bowel habit	Ever smoker	60	Haematological	0.002	(<0.001, 0.004)
Men	Change in bowel habit	Ever smoker	60	Other cancers	0.004	(0.002, 0.010)
Men	Change in bowel habit	Ever smoker	60	Death w/o cancer	0.003	(0.001, 0.010)
Men	Change in bowel habit	Ever smoker	70	Lung	0.005	(0.003, 0.010)
Men	Change in bowel habit	Ever smoker	70	Upper GI	0.006	(0.003, 0.014)
Men	Change in bowel habit	Ever smoker	70	Lower GI	0.085	(0.071, 0.101)
Men	Change in bowel habit	Ever smoker	70	Urological	0.004	(0.002, 0.009)
Men	Change in bowel habit	Ever smoker	70	Prostate	0.016	(0.008, 0.034)
Men	Change in bowel habit	Ever smoker	70	Haematological	0.003	(0.001, 0.009)
Men	Change in bowel habit	Ever smoker	70	Other cancers	0.007	(0.002, 0.020)
Men	Change in bowel habit	Ever smoker	70	Death w/o cancer	0.008	(0.004, 0.017)
Men	Change in bowel habit	Ever smoker	80	Lung	0.007	(0.003, 0.015)
Men	Change in bowel habit	Ever smoker	80	Upper GI	0.007	(0.004, 0.012)
Men	Change in bowel habit	Ever smoker	80	Lower GI	0.111	(0.092, 0.135)
Men	Change in bowel habit	Ever smoker	80	Urological	0.005	(0.002, 0.010)
Men	Change in bowel habit	Ever smoker	80	Prostate	0.019	(0.011, 0.030)
Men	Change in bowel habit	Ever smoker	80	Haematological	0.005	(0.002, 0.015)
Men	Change in bowel habit	Ever smoker	80	Other cancers	0.010	(0.004, 0.024)
Men	Change in bowel habit	Ever smoker	80	Death w/o cancer	0.024	(0.017, 0.032)
Men	Change in bowel habit	Ever smoker	90	Lung	0.007	(0.003, 0.020)
Men	Change in bowel habit	Ever smoker	90	Upper GI	0.007	(0.004, 0.014)
Men	Change in bowel habit	Ever smoker	90	Lower GI	0.128	(0.101, 0.161)
Men	Change in bowel habit	Ever smoker	90	Urological	0.005	(0.002, 0.010)
Men	Change in bowel habit	Ever smoker	90	Prostate	0.020	(0.014, 0.028)
Men	Change in bowel habit	Ever smoker	90	Haematological	0.006	(0.002, 0.019)
Men	Change in bowel habit	Ever smoker	90	Other cancers	0.012	(0.006, 0.025)
Men	Change in bowel habit	Ever smoker	90	Death w/o cancer	0.068	(0.054, 0.086)
Men	Dyspepsia	Never smoker	40	Death w/o cancer	0.001	(<0.001, 0.002)
Men	Dyspepsia	Never smoker	50	Upper GI	0.003	(0.001, 0.005)
Men	Dyspepsia	Never smoker	50	Other cancers	0.001	(<0.001, 0.003)
Men	Dyspepsia	Never smoker	50	Death w/o cancer	0.002	(0.001, 0.003)
Men	Dyspepsia	Never smoker	60	Upper GI	0.007	(0.003, 0.016)
Men	Dyspepsia	Never smoker	60	Lower GI	0.002	(0.001, 0.005)
Men	Dyspepsia	Never smoker	60	Urological	0.001	(<0.001, 0.003)
Men	Dyspepsia	Never smoker	60	Prostate	0.005	(0.002, 0.011)
Men	Dyspepsia	Never smoker	60	Haematological	0.002	(<0.001, 0.003)
Men	Dyspepsia	Never smoker	60	Other cancers	0.003	(0.001, 0.006)
Men	Dyspepsia	Never smoker	60	Death w/o cancer	0.005	(0.003, 0.009)
Men	Dyspepsia	Never smoker	70	Lung	0.002	(<0.001, 0.004)
Men	Dyspepsia	Never smoker	70	Upper GI	0.014	(0.009, 0.023)
Men	Dyspepsia	Never smoker	70	Lower GI	0.005	(0.003, 0.010)
Men	Dyspepsia	Never smoker	70	Urological	0.002	(<0.001, 0.006)
Men	Dyspepsia	Never smoker	70	Prostate	0.011	(0.006, 0.020)
Men	Dyspepsia	Never smoker	70	Haematological	0.003	(0.002, 0.007)
Men	Dyspepsia	Never smoker	70	Other cancers	0.004	(0.002, 0.009)
Men	Dyspepsia	Never smoker	70	Death w/o cancer	0.012	(0.006, 0.024)
Men	Dyspepsia	Never smoker	80	Lung	0.002	(<0.001, 0.005)
Men	Dyspepsia	Never smoker	80	Upper GI	0.017	(0.010, 0.030)

Sex	Index symptom	Smoking status	Age (years)	Outcome event	Probability of outcome	(95% CI)
Men	Dyspepsia	Never smoker	80	Lower GI	0.007	(0.003, 0.015)
Men	Dyspepsia	Never smoker	80	Urological	0.003	(0.001, 0.006)
Men	Dyspepsia	Never smoker	80	Prostate	0.015	(0.009, 0.027)
Men	Dyspepsia	Never smoker	80	Haematological	0.005	(0.002, 0.013)
Men	Dyspepsia	Never smoker	80	Other cancers	0.006	(0.002, 0.017)
Men	Dyspepsia	Never smoker	80	Death w/o cancer	0.035	(0.025, 0.050)
Men	Dyspepsia	Never smoker	90	Lung	0.002	(0.001, 0.005)
Men	Dyspepsia	Never smoker	90	Upper GI	0.019	(0.012, 0.029)
Men	Dyspepsia	Never smoker	90	Lower GI	0.007	(0.004, 0.015)
Men	Dyspepsia	Never smoker	90	Urological	0.003	(0.001, 0.009)
Men	Dyspepsia	Never smoker	90	Prostate	0.016	(0.010, 0.024)
Men	Dyspepsia	Never smoker	90	Haematological	0.006	(0.002, 0.013)
Men	Dyspepsia	Never smoker	90	Other cancers	0.008	(0.004, 0.016)
Men	Dyspepsia	Never smoker	90	Death w/o cancer	0.104	(0.085, 0.128)
Men	Dyspepsia	Ever smoker	40	Death w/o cancer	0.002	(<0.001, 0.003)
Men	Dyspepsia	Ever smoker	50	Upper GI	0.004	(0.001, 0.009)
Men	Dyspepsia	Ever smoker	50	Other cancers	0.002	(0.001, 0.004)
Men	Dyspepsia	Ever smoker	50	Death w/o cancer	0.002	(<0.001, 0.006)
Men	Dyspepsia	Ever smoker	60	Lung	0.003	(0.001, 0.007)
Men	Dyspepsia	Ever smoker	60	Upper GI	0.010	(0.005, 0.018)
Men	Dyspepsia	Ever smoker	60	Lower GI	0.003	(0.001, 0.006)
Men	Dyspepsia	Ever smoker	60	Urological	0.002	(<0.001, 0.005)
Men	Dyspepsia	Ever smoker	60	Prostate	0.004	(0.002, 0.008)
Men	Dyspepsia	Ever smoker	60	Haematological	0.002	(<0.001, 0.004)
Men	Dyspepsia	Ever smoker	60	Other cancers	0.003	(0.001, 0.007)
Men	Dyspepsia	Ever smoker	60	Death w/o cancer	0.006	(0.003, 0.011)
Men	Dyspepsia	Ever smoker	70	Lung	0.007	(0.003, 0.013)
Men	Dyspepsia	Ever smoker	70	Upper GI	0.019	(0.012, 0.030)
Men	Dyspepsia	Ever smoker	70	Lower GI	0.005	(0.002, 0.015)
Men	Dyspepsia	Ever smoker	70	Urological	0.003	(0.002, 0.007)
Men	Dyspepsia	Ever smoker	70	Prostate	0.010	(0.005, 0.022)
Men	Dyspepsia	Ever smoker	70	Haematological	0.003	(0.001, 0.008)
Men	Dyspepsia	Ever smoker	70	Other cancers	0.005	(0.002, 0.014)
Men	Dyspepsia	Ever smoker	70	Death w/o cancer	0.014	(0.008, 0.024)
Men	Dyspepsia	Ever smoker	80	Lung	0.008	(0.004, 0.017)
Men	Dyspepsia	Ever smoker	80	Upper GI	0.023	(0.013, 0.039)
Men	Dyspepsia	Ever smoker	80	Lower GI	0.007	(0.004, 0.013)
Men	Dyspepsia	Ever smoker	80	Urological	0.004	(0.002, 0.012)
Men	Dyspepsia	Ever smoker	80	Prostate	0.012	(0.007, 0.020)
Men	Dyspepsia	Ever smoker	80	Haematological	0.005	(0.002, 0.014)
Men	Dyspepsia	Ever smoker	80	Other cancers	0.007	(0.003, 0.019)
Men	Dyspepsia	Ever smoker	80	Death w/o cancer	0.038	(0.025, 0.056)
Men	Dyspepsia	Ever smoker	90	Lung	0.009	(0.004, 0.024)
Men	Dyspepsia	Ever smoker	90	Upper GI	0.024	(0.014, 0.039)
Men	Dyspepsia	Ever smoker	90	Lower GI	0.008	(0.004, 0.018)
Men	Dyspepsia	Ever smoker	90	Urological	0.004	(0.002, 0.012)
Men	Dyspepsia	Ever smoker	90	Prostate	0.014	(0.007, 0.027)
Men	Dyspepsia	Ever smoker	90	Haematological	0.005	(0.002, 0.012)
Men	Dyspepsia	Ever smoker	90	Other cancers	0.010	(0.005, 0.019)
Men	Dyspepsia	Ever smoker	90	Death w/o cancer	0.111	(0.091, 0.136)
Men	Dysphagia	Never smoker	30	Other cancers	0.001	(<0.001, 0.003)
Men	Dysphagia	Never smoker	30	Death w/o cancer	0.002	(0.001, 0.004)
Men	Dysphagia	Never smoker	40	Upper GI	0.003	(0.001, 0.008)
Men	Dysphagia	Never smoker	40	Other cancers	0.002	(<0.001, 0.004)
Men	Dysphagia	Never smoker	40	Death w/o cancer	0.005	(0.002, 0.009)
Men	Dysphagia	Never smoker	50	Upper GI	0.014	(0.008, 0.025)
Men	Dysphagia	Never smoker	50	Haematological	0.001	(<0.001, 0.002)
Men	Dysphagia	Never smoker	50	Other cancers	0.003	(0.001, 0.010)
Men	Dysphagia	Never smoker	50	Death w/o cancer	0.008	(0.005, 0.014)
Men	Dysphagia	Never smoker	60	Lung	0.001	(<0.001, 0.003)
Men	Dysphagia	Never smoker	60	Upper GI	0.042	(0.032, 0.054)
Men	Dysphagia	Never smoker	60	Lower GI	0.002	(<0.001, 0.006)

Sex	Index symptom	Smoking status	Age (years)	Outcome event	Probability of outcome	(95% CI)
Men	Dysphagia	Never smoker	60	Urological	0.001	(<0.001, 0.002)
Men	Dysphagia	Never smoker	60	Prostate	0.003	(0.001, 0.010)
Men	Dysphagia	Never smoker	60	Haematological	0.002	(0.001, 0.004)
Men	Dysphagia	Never smoker	60	Other cancers	0.006	(0.003, 0.012)
Men	Dysphagia	Never smoker	60	Death w/o cancer	0.017	(0.011, 0.028)
Men	Dysphagia	Never smoker	70	Lung	0.003	(<0.001, 0.007)
Men	Dysphagia	Never smoker	70	Upper GI	0.080	(0.059, 0.108)
Men	Dysphagia	Never smoker	70	Lower GI	0.005	(0.002, 0.010)
Men	Dysphagia	Never smoker	70	Urological	0.002	(<0.001, 0.004)
Men	Dysphagia	Never smoker	70	Prostate	0.008	(0.003, 0.020)
Men	Dysphagia	Never smoker	70	Haematological	0.005	(0.002, 0.013)
Men	Dysphagia	Never smoker	70	Other cancers	0.010	(0.006, 0.017)
Men	Dysphagia	Never smoker	70	Death w/o cancer	0.041	(0.030, 0.055)
Men	Dysphagia	Never smoker	80	Lung	0.003	(0.001, 0.010)
Men	Dysphagia	Never smoker	80	Upper GI	0.093	(0.075, 0.114)
Men	Dysphagia	Never smoker	80	Lower GI	0.006	(0.003, 0.014)
Men	Dysphagia	Never smoker	80	Urological	0.002	(<0.001, 0.005)
Men	Dysphagia	Never smoker	80	Prostate	0.010	(0.005, 0.019)
Men	Dysphagia	Never smoker	80	Haematological	0.006	(0.002, 0.015)
Men	Dysphagia	Never smoker	80	Other cancers	0.013	(0.007, 0.025)
Men	Dysphagia	Never smoker	80	Death w/o cancer	0.114	(0.091, 0.142)
Men	Dysphagia	Never smoker	90	Lung	0.003	(0.001, 0.010)
Men	Dysphagia	Never smoker	90	Upper GI	0.095	(0.073, 0.124)
Men	Dysphagia	Never smoker	90	Lower GI	0.006	(0.002, 0.017)
Men	Dysphagia	Never smoker	90	Urological	0.002	(<0.001, 0.004)
Men	Dysphagia	Never smoker	90	Prostate	0.010	(0.005, 0.020)
Men	Dysphagia	Never smoker	90	Haematological	0.006	(0.002, 0.019)
Men	Dysphagia	Never smoker	90	Other cancers	0.015	(0.010, 0.022)
Men	Dysphagia	Never smoker	90	Death w/o cancer	0.299	(0.264, 0.337)
Men	Dysphagia	Ever smoker	30	Other cancers	0.002	(<0.001, 0.003)
Men	Dysphagia	Ever smoker	30	Death w/o cancer	0.002	(0.001, 0.005)
Men	Dysphagia	Ever smoker	40	Upper GI	0.005	(0.002, 0.009)
Men	Dysphagia	Ever smoker	40	Other cancers	0.002	(<0.001, 0.005)
Men	Dysphagia	Ever smoker	40	Death w/o cancer	0.005	(0.003, 0.009)
Men	Dysphagia	Ever smoker	50	Lung	0.001	(<0.001, 0.003)
Men	Dysphagia	Ever smoker	50	Upper GI	0.020	(0.011, 0.036)
Men	Dysphagia	Ever smoker	50	Haematological	0.001	(<0.001, 0.002)
Men	Dysphagia	Ever smoker	50	Other cancers	0.004	(0.002, 0.012)
Men	Dysphagia	Ever smoker	50	Death w/o cancer	0.009	(0.005, 0.016)
Men	Dysphagia	Ever smoker	60	Lung	0.005	(<0.001, 0.000)
Men	Dysphagia	Ever smoker	60	Upper GI	0.054	(0.038, 0.076)
Men	Dysphagia	Ever smoker	60	Lower GI	0.002	(0.001, 0.005)
Men	Dysphagia	Ever smoker	60	Urological	0.001	(<0.001, 0.003)
Men	Dysphagia	Ever smoker	60	Prostate	0.003	(0.001, 0.008)
Men	Dysphagia	Ever smoker	60	Haematological	0.002	(0.001, 0.005)
Men	Dysphagia	Ever smoker	60	Other cancers	0.007	(0.004, 0.014)
Men	Dysphagia	Ever smoker	60	Death w/o cancer	0.019	(0.013, 0.029)
Men	Dysphagia	Ever smoker	70	Lung	0.010	(0.005, 0.020)
Men	Dysphagia	Ever smoker	70	Upper GI	0.103	(0.088, 0.120)
Men	Dysphagia	Ever smoker	70	Lower GI	0.005	(0.002, 0.015)
Men	Dysphagia	Ever smoker	70	Urological	0.002	(<0.001, 0.006)
Men	Dysphagia	Ever smoker	70	Prostate	0.007	(0.003, 0.016)
Men	Dysphagia	Ever smoker	70	Haematological	0.004	(0.002, 0.012)
Men	Dysphagia	Ever smoker	70	Other cancers	0.012	(0.007, 0.019)
Men	Dysphagia	Ever smoker	70	Death w/o cancer	0.043	(0.032, 0.059)
Men	Dysphagia	Ever smoker	80	Lung	0.013	(0.007, 0.025)
Men	Dysphagia	Ever smoker	80	Upper GI	0.124	(0.095, 0.160)
Men	Dysphagia	Ever smoker	80	Lower GI	0.006	(0.003, 0.012)
Men	Dysphagia	Ever smoker	80	Urological	0.002	(<0.001, 0.006)
Men	Dysphagia	Ever smoker	80	Prostate	0.008	(0.003, 0.023)
Men	Dysphagia	Ever smoker	80	Haematological	0.006	(0.002, 0.015)
Men	Dysphagia	Ever smoker	80	Other cancers	0.015	(0.009, 0.025)

Sex	Index symptom	Smoking status	Age (years)	Outcome event	Probability of outcome	(95% CI)
Men	Dysphagia	Ever smoker	80	Death w/o cancer	0.118	(0.095, 0.145)
Men	Dysphagia	Ever smoker	90	Lung	0.012	(0.006, 0.026)
Men	Dysphagia	Ever smoker	90	Upper GI	0.123	(0.100, 0.151)
Men	Dysphagia	Ever smoker	90	Lower GI	0.006	(0.003, 0.012)
Men	Dysphagia	Ever smoker	90	Urological	0.002	(<0.001, 0.006)
Men	Dysphagia	Ever smoker	90	Prostate	0.008	(0.004, 0.015)
Men	Dysphagia	Ever smoker	90	Haematological	0.007	(0.002, 0.020)
Men	Dysphagia	Ever smoker	90	Other cancers	0.018	(0.011, 0.031)
Men	Dysphagia	Ever smoker	90	Death w/o cancer	0.311	(0.281, 0.343)
Men	Dyspnoea	Never smoker	30	Death w/o cancer	0.001	(<0.001, 0.003)
Men	Dyspnoea	Never smoker	40	Death w/o cancer	0.003	(0.001, 0.007)
Men	Dyspnoea	Never smoker	50	Other cancers	0.001	(<0.001, 0.003)
Men	Dyspnoea	Never smoker	50	Death w/o cancer	0.006	(0.003, 0.012)
Men	Dyspnoea	Never smoker	60	Lung	0.002	(<0.001, 0.005)
Men	Dyspnoea	Never smoker	60	Upper GI	0.002	(<0.001, 0.003)
Men	Dyspnoea	Never smoker	60	Lower GI	0.002	(0.001, 0.004)
Men	Dyspnoea	Never smoker	60	Urological	0.001	(<0.001, 0.002)
Men	Dyspnoea	Never smoker	60	Prostate	0.003	(0.001, 0.008)
Men	Dyspnoea	Never smoker	60	Haematological	0.002	(0.001, 0.005)
Men	Dyspnoea	Never smoker	60	Other cancers	0.003	(0.001, 0.005)
Men	Dyspnoea	Never smoker	60	Death w/o cancer	0.013	(0.007, 0.022)
Men	Dyspnoea	Never smoker	70	Lung	0.005	(0.002, 0.013)
Men	Dyspnoea	Never smoker	70	Upper GI	0.003	(0.001, 0.008)
Men	Dyspnoea	Never smoker	70	Lower GI	0.005	(0.002, 0.010)
Men	Dyspnoea	Never smoker	70	Urological	0.003	(0.001, 0.006)
Men	Dyspnoea	Never smoker	70	Prostate	0.008	(0.004, 0.018)
Men	Dyspnoea	Never smoker	70	Haematological	0.004	(0.002, 0.010)
Men	Dyspnoea	Never smoker	70	Other cancers	0.004	(0.002, 0.009)
Men	Dyspnoea	Never smoker	70	Death w/o cancer	0.030	(0.021, 0.042)
Men	Dyspnoea	Never smoker	80	Lung	0.006	(0.003, 0.013)
Men	Dyspnoea	Never smoker	80	Upper GI	0.003	(0.001, 0.009)
Men	Dyspnoea	Never smoker	80	Lower GI	0.006	(0.003, 0.013)
Men	Dyspnoea	Never smoker	80	Urological	0.003	(0.001, 0.006)
Men	Dyspnoea	Never smoker	80	Prostate	0.011	(0.006, 0.018)
Men	Dyspnoea	Never smoker	80	Haematological	0.006	(0.003, 0.013)
Men	Dyspnoea	Never smoker	80	Other cancers	0.006	(0.003, 0.012)
Men	Dyspnoea	Never smoker	80	Death w/o cancer	0.087	(0.070, 0.107)
Men	Dyspnoea	Never smoker	90	Lung	0.007	(0.003, 0.015)
Men	Dyspnoea	Never smoker	90	Upper GI	0.004	(0.001, 0.009)
Men	Dyspnoea	Never smoker	90	Lower GI	0.007	(0.003, 0.013)
Men	Dyspnoea	Never smoker	90	Urological	0.003	(0.002, 0.007)
Men	Dyspnoea	Never smoker	90	Prostate	0.011	(0.006, 0.021)
Men	Dyspnoea	Never smoker	90	Haematological	0.006	(0.003, 0.012)
Men	Dyspnoea	Never smoker	90	Other cancers	0.007	(0.003, 0.017)
Men	Dyspnoea	Never smoker	90	Death w/o cancer	0.239	(0.212, 0.267)
Men	Dyspnoea	Ever smoker	30	Death w/o cancer	0.002	(<0.001, 0.004)
Men	Dyspnoea	Ever smoker	40	Death w/o cancer	0.004	(0.001, 0.011)
Men	Dyspnoea	Ever smoker	50	Lung	0.003	(<0.001, 0.000)
Men	Dyspnoea	Ever smoker	50	Other cancers	0.002	(0.001, 0.003)
Men	Dyspnoea	Ever smoker	50	Death w/o cancer	0.006	(0.003, 0.014)
Men	Dyspnoea	Ever smoker	60	Lung	0.009	(0.004, 0.018)
Men	Dyspnoea	Ever smoker	60	Upper GI	0.002	(0.001, 0.005)
Men	Dyspnoea	Ever smoker	60	Lower GI	0.002	(<0.001, 0.005)
Men	Dyspnoea	Ever smoker	60	Urological	0.002	(<0.001, 0.004)
Men	Dyspnoea	Ever smoker	60	Prostate	0.003	(0.001, 0.008)
Men	Dyspnoea	Ever smoker	60	Haematological	0.002	(<0.001, 0.005)
Men	Dyspnoea	Ever smoker	60	Other cancers	0.003	(0.001, 0.007)
Men	Dyspnoea	Ever smoker	60	Death w/o cancer	0.013	(0.007, 0.025)
Men	Dyspnoea	Ever smoker	70	Lung	0.019	(0.013, 0.030)
Men	Dyspnoea	Ever smoker	70	Upper GI	0.004	(0.001, 0.011)
Men	Dyspnoea	Ever smoker	70	Lower GI	0.005	(0.002, 0.011)
Men	Dyspnoea	Ever smoker	70	Urological	0.004	(0.002, 0.009)

Sex	Index symptom	Smoking status	Age (years)	Outcome event	Probability of outcome	(95% CI)
Men	Dyspnoea	Ever smoker	70	Prostate	0.007	(0.004, 0.016)
Men	Dyspnoea	Ever smoker	70	Haematological	0.004	(0.002, 0.009)
Men	Dyspnoea	Ever smoker	70	Other cancers	0.005	(0.002, 0.014)
Men	Dyspnoea	Ever smoker	70	Death w/o cancer	0.033	(0.021, 0.050)
Men	Dyspnoea	Ever smoker	80	Lung	0.024	(0.015, 0.039)
Men	Dyspnoea	Ever smoker	80	Upper GI	0.005	(0.001, 0.016)
Men	Dyspnoea	Ever smoker	80	Lower GI	0.006	(0.003, 0.012)
Men	Dyspnoea	Ever smoker	80	Urological	0.004	(0.001, 0.011)
Men	Dyspnoea	Ever smoker	80	Prostate	0.009	(0.004, 0.022)
Men	Dyspnoea	Ever smoker	80	Haematological	0.006	(0.002, 0.013)
Men	Dyspnoea	Ever smoker	80	Other cancers	0.007	(0.003, 0.016)
Men	Dyspnoea	Ever smoker	80	Death w/o cancer	0.093	(0.077, 0.112)
Men	Dyspnoea	Ever smoker	90	Lung	0.027	(0.016, 0.043)
Men	Dyspnoea	Ever smoker	90	Upper GI	0.005	(0.002, 0.013)
Men	Dyspnoea	Ever smoker	90	Lower GI	0.007	(0.004, 0.013)
Men	Dyspnoea	Ever smoker	90	Urological	0.004	(0.002, 0.010)
Men	Dyspnoea	Ever smoker	90	Prostate	0.010	(0.005, 0.019)
Men	Dyspnoea	Ever smoker	90	Haematological	0.007	(0.003, 0.013)
Men	Dyspnoea	Ever smoker	90	Other cancers	0.009	(0.005, 0.015)
Men	Dyspnoea	Ever smoker	90	Death w/o cancer	0.251	(0.227, 0.275)
Men	Fatigue	Never smoker	40	Other cancers	0.001	(<0.001, 0.002)
Men	Fatigue	Never smoker	40	Death w/o cancer	0.002	(<0.001, 0.005)
Men	Fatigue	Never smoker	50	Lower GI	0.001	(<0.001, 0.003)
Men	Fatigue	Never smoker	50	Haematological	0.001	(<0.001, 0.003)
Men	Fatigue	Never smoker	50	Other cancers	0.002	(<0.001, 0.004)
Men	Fatigue	Never smoker	50	Death w/o cancer	0.004	(0.002, 0.010)
Men	Fatigue	Never smoker	60	Lung	0.002	(<0.001, 0.003)
Men	Fatigue	Never smoker	60	Upper GI	0.002	(<0.001, 0.005)
Men	Fatigue	Never smoker	60	Lower GI	0.003	(0.001, 0.008)
Men	Fatigue	Never smoker	60	Urological	0.001	(<0.001, 0.003)
Men	Fatigue	Never smoker	60	Prostate	0.006	(0.003, 0.012)
Men	Fatigue	Never smoker	60	Haematological	0.003	(0.001, 0.008)
Men	Fatigue	Never smoker	60	Other cancers	0.003	(0.001, 0.009)
Men	Fatigue	Never smoker	60	Death w/o cancer	0.008	(0.005, 0.014)
Men	Fatigue	Never smoker	70	Lung	0.003	(0.001, 0.008)
Men	Fatigue	Never smoker	70	Upper GI	0.004	(0.002, 0.010)
Men	Fatigue	Never smoker	70	Lower GI	0.007	(0.004, 0.012)
Men	Fatigue	Never smoker	70	Urological	0.003	(0.001, 0.007)
Men	Fatigue	Never smoker	70	Prostate	0.015	(0.010, 0.023)
Men	Fatigue	Never smoker	70	Haematological	0.006	(0.003, 0.012)
Men	Fatigue	Never smoker	70	Other cancers	0.005	(0.002, 0.012)
Men	Fatigue	Never smoker	70	Death w/o cancer	0.021	(0.014, 0.030)
Men	Fatigue	Never smoker	80	Lung	0.004	(0.002, 0.007)
Men	Fatigue	Never smoker	80	Upper GI	0.005	(0.002, 0.015)
Men	Fatigue	Never smoker	80	Lower GI	0.008	(0.004, 0.015)
Men	Fatigue	Never smoker	80	Urological	0.003	(0.001, 0.009)
Men	Fatigue	Never smoker	80	Prostate	0.018	(0.011, 0.029)
Men	Fatigue	Never smoker	80	Haematological	0.008	(0.005, 0.013)
Men	Fatigue	Never smoker	80	Other cancers	0.007	(0.003, 0.016)
Men	Fatigue	Never smoker	80	Death w/o cancer	0.059	(0.044, 0.079)
Men	Fatigue	Never smoker	90	Lung	0.004	(0.001, 0.010)
Men	Fatigue	Never smoker	90	Upper GI	0.005	(0.003, 0.009)
Men	Fatigue	Never smoker	90	Lower GI	0.010	(0.005, 0.018)
Men	Fatigue	Never smoker	90	Urological	0.003	(0.002, 0.007)
Men	Fatigue	Never smoker	90	Prostate	0.019	(0.013, 0.029)
Men	Fatigue	Never smoker	90	Haematological	0.009	(0.005, 0.016)
Men	Fatigue	Never smoker	90	Other cancers	0.010	(0.005, 0.017)
Men	Fatigue	Never smoker	90	Death w/o cancer	0.167	(0.140, 0.198)
Men	Fatigue	Ever smoker	30	Death w/o cancer	0.001	(<0.001, 0.003)
Men	Fatigue	Ever smoker	40	Other cancers	0.001	(<0.001, 0.002)
Men	Fatigue	Ever smoker	40	Death w/o cancer	0.002	(<0.001, 0.006)
Men	Fatigue	Ever smoker	50	Lung	0.002	(<0.001, 0.003)

Sex	Index symptom	Smoking status	Age (years)	Outcome event	Probability of outcome	(95% CI)
Men	Fatigue	Ever smoker	50	Upper GI	0.001	(<0.001, 0.002)
Men	Fatigue	Ever smoker	50	Lower GI	0.001	(<0.001, 0.002)
Men	Fatigue	Ever smoker	50	Haematological	0.001	(<0.001, 0.003)
Men	Fatigue	Ever smoker	50	Other cancers	0.002	(0.001, 0.004)
Men	Fatigue	Ever smoker	50	Death w/o cancer	0.004	(0.002, 0.010)
Men	Fatigue	Ever smoker	60	Lung	0.005	(0.002, 0.012)
Men	Fatigue	Ever smoker	60	Upper GI	0.003	(0.001, 0.005)
Men	Fatigue	Ever smoker	60	Lower GI	0.003	(0.001, 0.007)
Men	Fatigue	Ever smoker	60	Urological	0.002	(0.001, 0.003)
Men	Fatigue	Ever smoker	60	Prostate	0.006	(0.003, 0.013)
Men	Fatigue	Ever smoker	60	Haematological	0.003	(0.001, 0.008)
Men	Fatigue	Ever smoker	60	Other cancers	0.004	(0.002, 0.006)
Men	Fatigue	Ever smoker	60	Death w/o cancer	0.010	(0.005, 0.018)
Men	Fatigue	Ever smoker	70	Lung	0.012	(0.006, 0.020)
Men	Fatigue	Ever smoker	70	Upper GI	0.005	(0.002, 0.011)
Men	Fatigue	Ever smoker	70	Lower GI	0.007	(0.003, 0.014)
Men	Fatigue	Ever smoker	70	Urological	0.003	(0.001, 0.009)
Men	Fatigue	Ever smoker	70	Prostate	0.013	(0.007, 0.022)
Men	Fatigue	Ever smoker	70	Haematological	0.006	(0.003, 0.012)
Men	Fatigue	Ever smoker	70	Other cancers	0.007	(0.003, 0.015)
Men	Fatigue	Ever smoker	70	Death w/o cancer	0.022	(0.015, 0.032)
Men	Fatigue	Ever smoker	80	Lung	0.015	(0.007, 0.029)
Men	Fatigue	Ever smoker	80	Upper GI	0.007	(0.004, 0.012)
Men	Fatigue	Ever smoker	80	Lower GI	0.010	(0.004, 0.023)
Men	Fatigue	Ever smoker	80	Urological	0.004	(0.002, 0.010)
Men	Fatigue	Ever smoker	80	Prostate	0.016	(0.009, 0.029)
Men	Fatigue	Ever smoker	80	Haematological	0.008	(0.004, 0.016)
Men	Fatigue	Ever smoker	80	Other cancers	0.009	(0.005, 0.014)
Men	Fatigue	Ever smoker	80	Death w/o cancer	0.064	(0.047, 0.088)
Men	Fatigue	Ever smoker	90	Lung	0.016	(0.008, 0.030)
Men	Fatigue	Ever smoker	90	Upper GI	0.007	(0.003, 0.015)
Men	Fatigue	Ever smoker	90	Lower GI	0.009	(0.005, 0.019)
Men	Fatigue	Ever smoker	90	Urological	0.005	(0.002, 0.013)
Men	Fatigue	Ever smoker	90	Prostate	0.017	(0.010, 0.028)
Men	Fatigue	Ever smoker	90	Haematological	0.010	(0.005, 0.020)
Men	Fatigue	Ever smoker	90	Other cancers	0.011	(0.005, 0.023)
Men	Fatigue	Ever smoker	90	Death w/o cancer	0.178	(0.157, 0.201)
Men	Haematuria	Never smoker	30	Urological	0.003	(<0.001, 0.007)
Men	Haematuria	Never smoker	40	Urological	0.009	(0.005, 0.015)
Men	Haematuria	Never smoker	40	Death w/o cancer	0.002	(<0.001, 0.003)
Men	Haematuria	Never smoker	50	Urological	0.022	(0.014, 0.035)
Men	Haematuria	Never smoker	50	Prostate	0.003	(<0.001, 0.007)
Men	Haematuria	Never smoker	50	Other cancers	0.001	(<0.001, 0.003)
Men	Haematuria	Never smoker	50	Death w/o cancer	0.003	(0.001, 0.007)
Men	Haematuria	Never smoker	60	Lower GI	0.001	(<0.001, 0.003)
Men	Haematuria	Never smoker	60	Urological	0.055	(0.040, 0.076)
Men	Haematuria	Never smoker	60	Prostate	0.018	(0.012, 0.029)
Men	Haematuria	Never smoker	60	Haematological	0.002	(<0.001, 0.003)
Men	Haematuria	Never smoker	60	Other cancers	0.002	(<0.001, 0.005)
Men	Haematuria	Never smoker	60	Death w/o cancer	0.006	(0.003, 0.010)
Men	Haematuria	Never smoker	70	Lung	0.001	(<0.001, 0.003)
Men	Haematuria	Never smoker	70	Upper GI	0.002	(<0.001, 0.003)
Men	Haematuria	Never smoker	70	Lower GI	0.003	(0.001, 0.009)
Men	Haematuria	Never smoker	70	Urological	0.097	(0.078, 0.119)
Men	Haematuria	Never smoker	70	Prostate	0.044	(0.034, 0.057)
Men	Haematuria	Never smoker	70	Haematological	0.003	(0.001, 0.007)
Men	Haematuria	Never smoker	70	Other cancers	0.004	(0.002, 0.007)
Men	Haematuria	Never smoker	70	Death w/o cancer	0.016	(0.010, 0.025)
Men	Haematuria	Never smoker	80	Lung	0.001	(<0.001, 0.003)
Men	Haematuria	Never smoker	80	Upper GI	0.002	(<0.001, 0.003)
Men	Haematuria	Never smoker	80	Lower GI	0.004	(0.002, 0.008)
Men	Haematuria	Never smoker	80	Urological	0.117	(0.099, 0.137)

Sex	Index symptom	Smoking status	Age (years)	Outcome event	Probability of outcome	(95% CI)
Men	Haematuria	Never smoker	80	Prostate	0.057	(0.043, 0.075)
Men	Haematuria	Never smoker	80	Haematological	0.003	(0.002, 0.006)
Men	Haematuria	Never smoker	80	Other cancers	0.005	(0.002, 0.009)
Men	Haematuria	Never smoker	80	Death w/o cancer	0.043	(0.032, 0.057)
Men	Haematuria	Never smoker	90	Lung	0.002	(<0.001, 0.004)
Men	Haematuria	Never smoker	90	Upper GI	0.002	(<0.001, 0.004)
Men	Haematuria	Never smoker	90	Lower GI	0.005	(0.002, 0.013)
Men	Haematuria	Never smoker	90	Urological	0.127	(0.102, 0.157)
Men	Haematuria	Never smoker	90	Prostate	0.059	(0.045, 0.078)
Men	Haematuria	Never smoker	90	Haematological	0.004	(0.001, 0.011)
Men	Haematuria	Never smoker	90	Other cancers	0.006	(0.003, 0.013)
Men	Haematuria	Never smoker	90	Death w/o cancer	0.121	(0.101, 0.144)
Men	Haematuria	Ever smoker	30	Urological	0.004	(0.002, 0.008)
Men	Haematuria	Ever smoker	40	Urological	0.012	(0.007, 0.021)
Men	Haematuria	Ever smoker	40	Death w/o cancer	0.002	(<0.001, 0.004)
Men	Haematuria	Ever smoker	50	Urological	0.029	(0.021, 0.039)
Men	Haematuria	Ever smoker	50	Prostate	0.002	(0.001, 0.004)
Men	Haematuria	Ever smoker	50	Other cancers	0.001	(<0.001, 0.003)
Men	Haematuria	Ever smoker	50	Death w/o cancer	0.003	(0.002, 0.007)
Men	Haematuria	Ever smoker	60	Lung	0.002	(<0.001, 0.006)
Men	Haematuria	Ever smoker	60	Upper GI	0.001	(<0.001, 0.002)
Men	Haematuria	Ever smoker	60	Lower GI	0.002	(<0.001, 0.003)
Men	Haematuria	Ever smoker	60	Urological	0.076	(0.059, 0.097)
Men	Haematuria	Ever smoker	60	Prostate	0.017	(0.010, 0.027)
Men	Haematuria	Ever smoker	60	Haematological	0.001	(<0.001, 0.003)
Men	Haematuria	Ever smoker	60	Other cancers	0.003	(0.001, 0.006)
Men	Haematuria	Ever smoker	60	Death w/o cancer	0.007	(0.004, 0.012)
Men	Haematuria	Ever smoker	70	Lung	0.004	(0.002, 0.011)
Men	Haematuria	Ever smoker	70	Upper GI	0.002	(<0.001, 0.005)
Men	Haematuria	Ever smoker	70	Lower GI	0.003	(0.001, 0.008)
Men	Haematuria	Ever smoker	70	Urological	0.129	(0.108, 0.154)
Men	Haematuria	Ever smoker	70	Prostate	0.039	(0.026, 0.057)
Men	Haematuria	Ever smoker	70	Haematological	0.002	(<0.001, 0.006)
Men	Haematuria	Ever smoker	70	Other cancers	0.004	(0.002, 0.009)
Men	Haematuria	Ever smoker	70	Death w/o cancer	0.016	(0.010, 0.025)
Men	Haematuria	Ever smoker	80	Lung	0.006	(0.003, 0.012)
Men	Haematuria	Ever smoker	80	Upper GI	0.003	(<0.001, 0.007)
Men	Haematuria	Ever smoker	80	Lower GI	0.004	(0.001, 0.011)
Men	Haematuria	Ever smoker	80	Urological	0.153	(0.131, 0.178)
Men	Haematuria	Ever smoker	80	Prostate	0.048	(0.037, 0.063)
Men	Haematuria	Ever smoker	80	Haematological	0.003	(0.001, 0.006)
Men	Haematuria	Ever smoker	80	Other cancers	0.005	(0.002, 0.017)
Men	Haematuria	Ever smoker	80	Death w/o cancer	0.046	(0.032, 0.065)
Men	Haematuria	Ever smoker	90	Lung	0.006	(0.003, 0.012)
Men	Haematuria	Ever smoker	90	Upper GI	0.003	(0.001, 0.005)
Men	Haematuria	Ever smoker	90	Lower GI	0.005	(0.002, 0.013)
Men	Haematuria	Ever smoker	90	Urological	0.164	(0.133, 0.201)
Men	Haematuria	Ever smoker	90	Prostate	0.052	(0.037, 0.073)
Men	Haematuria	Ever smoker	90	Haematological	0.004	(0.002, 0.010)
Men	Haematuria	Ever smoker	90	Other cancers	0.007	(0.002, 0.021)
Men	Haematuria	Ever smoker	90	Death w/o cancer	0.126	(0.108, 0.147)
Men	Haemoptysis	Never smoker	30	Other cancers	0.001	(<0.001, 0.002)
Men	Haemoptysis	Never smoker	30	Death w/o cancer	0.001	(<0.001, 0.003)
Men	Haemoptysis	Never smoker	40	Other cancers	0.001	(<0.001, 0.002)
Men	Haemoptysis	Never smoker	40	Death w/o cancer	0.003	(0.001, 0.007)
Men	Haemoptysis	Never smoker	50	Lung	0.005	(0.002, 0.010)
Men	Haemoptysis	Never smoker	50	Haematological	0.001	(<0.001, 0.003)
Men	Haemoptysis	Never smoker	50	Other cancers	0.002	(0.001, 0.006)
Men	Haemoptysis	Never smoker	50	Death w/o cancer	0.005	(0.003, 0.010)
Men	Haemoptysis	Never smoker	60	Lung	0.015	(0.009, 0.024)
Men	Haemoptysis	Never smoker	60	Upper GI	0.002	(0.001, 0.005)
Men	Haemoptysis	Never smoker	60	Lower GI	0.002	(<0.001, 0.004)

Sex	Index symptom	Smoking status	Age (years)	Outcome event	Probability of outcome	(95% CI)
Men	Haemoptysis	Never smoker	60	Prostate	0.003	(0.001, 0.007)
Men	Haemoptysis	Never smoker	60	Haematological	0.003	(0.002, 0.006)
Men	Haemoptysis	Never smoker	60	Other cancers	0.004	(0.002, 0.010)
Men	Haemoptysis	Never smoker	60	Death w/o cancer	0.011	(0.007, 0.019)
Men	Haemoptysis	Never smoker	70	Lung	0.034	(0.025, 0.047)
Men	Haemoptysis	Never smoker	70	Upper GI	0.005	(0.002, 0.013)
Men	Haemoptysis	Never smoker	70	Lower GI	0.004	(0.001, 0.009)
Men	Haemoptysis	Never smoker	70	Urological	0.002	(<0.001, 0.004)
Men	Haemoptysis	Never smoker	70	Prostate	0.008	(0.004, 0.016)
Men	Haemoptysis	Never smoker	70	Haematological	0.007	(0.003, 0.014)
Men	Haemoptysis	Never smoker	70	Other cancers	0.007	(0.003, 0.019)
Men	Haemoptysis	Never smoker	70	Death w/o cancer	0.028	(0.019, 0.042)
Men	Haemoptysis	Never smoker	80	Lung	0.044	(0.030, 0.065)
Men	Haemoptysis	Never smoker	80	Upper GI	0.005	(0.002, 0.010)
Men	Haemoptysis	Never smoker	80	Lower GI	0.004	(0.001, 0.014)
Men	Haemoptysis	Never smoker	80	Urological	0.002	(<0.001, 0.004)
Men	Haemoptysis	Never smoker	80	Prostate	0.009	(0.004, 0.020)
Men	Haemoptysis	Never smoker	80	Haematological	0.010	(0.005, 0.019)
Men	Haemoptysis	Never smoker	80	Other cancers	0.009	(0.004, 0.020)
Men	Haemoptysis	Never smoker	80	Death w/o cancer	0.078	(0.060, 0.100)
Men	Haemoptysis	Never smoker	90	Lung	0.048	(0.034, 0.067)
Men	Haemoptysis	Never smoker	90	Upper GI	0.005	(0.002, 0.011)
Men	Haemoptysis	Never smoker	90	Lower GI	0.004	(0.002, 0.013)
Men	Haemoptysis	Never smoker	90	Urological	0.002	(<0.001, 0.004)
Men	Haemoptysis	Never smoker	90	Prostate	0.009	(0.005, 0.015)
Men	Haemoptysis	Never smoker	90	Haematological	0.011	(0.005, 0.026)
Men	Haemoptysis	Never smoker	90	Other cancers	0.012	(0.005, 0.028)
Men	Haemoptysis	Never smoker	90	Death w/o cancer	0.217	(0.189, 0.248)
Men	Haemoptysis	Ever smoker	30	Other cancers	0.001	(<0.001, 0.002)
Men	Haemoptysis	Ever smoker	30	Death w/o cancer	0.001	(<0.001, 0.003)
Men	Haemoptysis	Ever smoker	40	Lung	0.002	(<0.001, 0.004)
Men	Haemoptysis	Ever smoker	40	Other cancers	0.002	(<0.001, 0.004)
Men	Haemoptysis	Ever smoker	40	Death w/o cancer	0.003	(0.002, 0.007)
Men	Haemoptysis	Ever smoker	50	Lung	0.017	(0.011, 0.026)
Men	Haemoptysis	Ever smoker	50	Upper GI	0.001	(<0.001, 0.002)
Men	Haemoptysis	Ever smoker	50	Haematological	0.002	(<0.001, 0.004)
Men	Haemoptysis	Ever smoker	50	Other cancers	0.003	(0.001, 0.006)
Men	Haemoptysis	Ever smoker	50	Death w/o cancer	0.006	(0.003, 0.012)
Men	Haemoptysis	Ever smoker	60	Lung	0.059	(0.043, 0.079)
Men	Haemoptysis	Ever smoker	60	Upper GI	0.003	(0.001, 0.007)
Men	Haemoptysis	Ever smoker	60	Lower GI	0.002	(<0.001, 0.003)
Men	Haemoptysis	Ever smoker	60	Urological	0.001	(<0.001, 0.003)
Men	Haemoptysis	Ever smoker	60	Prostate	0.003	(0.001, 0.007)
Men	Haemoptysis	Ever smoker	60	Haematological	0.003	(0.001, 0.010)
Men	Haemoptysis	Ever smoker	60	Other cancers	0.005	(0.003, 0.010)
Men	Haemoptysis	Ever smoker	60	Death w/o cancer	0.012	(0.008, 0.019)
Men	Haemoptysis	Ever smoker	70	Lung	0.129	(0.105, 0.157)
Men	Haemoptysis	Ever smoker	70	Upper GI	0.005	(0.002, 0.010)
Men	Haemoptysis	Ever smoker	70	Lower GI	0.003	(0.001, 0.009)
Men	Haemoptysis	Ever smoker	70	Urological	0.002	(<0.001, 0.004)
Men	Haemoptysis	Ever smoker	70	Prostate	0.006	(0.002, 0.015)
Men	Haemoptysis	Ever smoker	70	Haematological	0.006	(0.003, 0.013)
Men	Haemoptysis	Ever smoker	70	Other cancers	0.008	(0.004, 0.016)
Men	Haemoptysis	Ever smoker	70	Death w/o cancer	0.028	(0.019, 0.040)
Men	Haemoptysis	Ever smoker	80	Lung	0.164	(0.126, 0.210)
Men	Haemoptysis	Ever smoker	80	Upper GI	0.006	(0.002, 0.015)
Men	Haemoptysis	Ever smoker	80	Lower GI	0.004	(0.002, 0.013)
Men	Haemoptysis	Ever smoker	80	Urological	0.002	(<0.001, 0.004)
Men	Haemoptysis	Ever smoker	80	Prostate	0.007	(0.003, 0.016)
Men	Haemoptysis	Ever smoker	80	Haematological	0.008	(0.003, 0.022)
Men	Haemoptysis	Ever smoker	80	Other cancers	0.011	(0.006, 0.019)
Men	Haemoptysis	Ever smoker	80	Death w/o cancer	0.076	(0.058, 0.099)

Sex	Index symptom	Smoking status	Age (years)	Outcome event	Probability of outcome	(95% CI)
Men	Haemoptysis	Ever smoker	90	Lung	0.176	(0.147, 0.209)
Men	Haemoptysis	Ever smoker	90	Upper GI	0.006	(0.002, 0.021)
Men	Haemoptysis	Ever smoker	90	Lower GI	0.004	(0.001, 0.014)
Men	Haemoptysis	Ever smoker	90	Urological	0.002	(<0.001, 0.004)
Men	Haemoptysis	Ever smoker	90	Prostate	0.007	(0.004, 0.013)
Men	Haemoptysis	Ever smoker	90	Haematological	0.009	(0.004, 0.022)
Men	Haemoptysis	Ever smoker	90	Other cancers	0.013	(0.006, 0.026)
Men	Haemoptysis	Ever smoker	90	Death w/o cancer	0.208	(0.166, 0.256)
Men	Jaundice	Never smoker	30	Upper GI	0.002	(<0.001, 0.005)
Men	Jaundice	Never smoker	30	Other cancers	0.009	(0.002, 0.034)
Men	Jaundice	Never smoker	30	Death w/o cancer	0.005	(0.003, 0.010)
Men	Jaundice	Never smoker	40	Upper GI	0.010	(0.006, 0.019)
Men	Jaundice	Never smoker	40	Haematological	0.002	(<0.001, 0.004)
Men	Jaundice	Never smoker	40	Other cancers	0.012	(0.006, 0.022)
Men	Jaundice	Never smoker	40	Death w/o cancer	0.011	(0.007, 0.019)
Men	Jaundice	Never smoker	50	Upper GI	0.043	(0.030, 0.061)
Men	Jaundice	Never smoker	50	Lower GI	0.002	(<0.001, 0.004)
Men	Jaundice	Never smoker	50	Urological	0.002	(<0.001, 0.003)
Men	Jaundice	Never smoker	50	Haematological	0.003	(0.002, 0.008)
Men	Jaundice	Never smoker	50	Other cancers	0.025	(0.015, 0.039)
Men	Jaundice	Never smoker	50	Death w/o cancer	0.019	(0.012, 0.032)
Men	Jaundice	Never smoker	60	Lung	0.002	(<0.001, 0.005)
Men	Jaundice	Never smoker	60	Upper GI	0.115	(0.091, 0.144)
Men	Jaundice	Never smoker	60	Lower GI	0.004	(0.002, 0.007)
Men	Jaundice	Never smoker	60	Urological	0.004	(0.001, 0.014)
Men	Jaundice	Never smoker	60	Prostate	0.004	(0.001, 0.014)
Men	Jaundice	Never smoker	60	Haematological	0.007	(0.004, 0.014)
Men	Jaundice	Never smoker	60	Other cancers	0.041	(0.028, 0.060)
Men	Jaundice	Never smoker	60	Death w/o cancer	0.038	(0.028, 0.051)
Men	Jaundice	Never smoker	70	Lung	0.003	(0.001, 0.006)
Men	Jaundice	Never smoker	70	Upper GI	0.204	(0.158, 0.259)
Men	Jaundice	Never smoker	70	Lower GI	0.008	(0.003, 0.021)
Men	Jaundice	Never smoker	70	Urological	0.006	(0.002, 0.022)
Men	Jaundice	Never smoker	70	Prostate	0.008	(0.003, 0.028)
Men	Jaundice	Never smoker	70	Haematological	0.012	(0.006, 0.022)
Men	Jaundice	Never smoker	70	Other cancers	0.061	(0.039, 0.092)
Men	Jaundice	Never smoker	70	Death w/o cancer	0.085	(0.065, 0.111)
Men	Jaundice	Never smoker	80	Lung	0.003	(<0.001, 0.010)
Men	Jaundice	Never smoker	80	Upper GI	0.226	(0.187, 0.271)
Men	Jaundice	Never smoker	80	Lower GI	0.009	(0.004, 0.018)
Men	Jaundice	Never smoker	80	Urological	0.006	(0.002, 0.017)
Men	Jaundice	Never smoker	80	Prostate	0.009	(0.004, 0.020)
Men	Jaundice	Never smoker	80	Haematological	0.014	(0.007, 0.028)
Men	Jaundice	Never smoker	80	Other cancers	0.079	(0.055, 0.111)
Men	Jaundice	Never smoker	80	Death w/o cancer	0.210	(0.182, 0.242)
Men	Jaundice	Never smoker	90	Lung	0.003	(<0.001, 0.010)
Men	Jaundice	Never smoker	90	Upper GI	0.199	(0.161, 0.242)
Men	Jaundice	Never smoker	90	Lower GI	0.008	(0.003, 0.024)
Men	Jaundice	Never smoker	90	Urological	0.004	(0.002, 0.010)
Men	Jaundice	Never smoker	90	Prostate	0.007	(0.003, 0.017)
Men	Jaundice	Never smoker	90	Haematological	0.014	(0.007, 0.026)
Men	Jaundice	Never smoker	90	Other cancers	0.086	(0.062, 0.118)
Men	Jaundice	Never smoker	90	Death w/o cancer	0.472	(0.407, 0.537)
Men	Jaundice	Ever smoker	30	Upper GI	0.002	(<0.001, 0.006)
Men	Jaundice	Ever smoker	30	Other cancers	0.011	(0.006, 0.023)
Men	Jaundice	Ever smoker	30	Death w/o cancer	0.006	(0.003, 0.010)
Men	Jaundice	Ever smoker	40	Upper GI	0.012	(0.007, 0.021)
Men	Jaundice	Ever smoker	40	Haematological	0.002	(<0.001, 0.005)
Men	Jaundice	Ever smoker	40	Other cancers	0.014	(0.009, 0.022)
Men	Jaundice	Ever smoker	40	Death w/o cancer	0.013	(0.008, 0.022)
Men	Jaundice	Ever smoker	50	Lung	0.002	(<0.001, 0.005)
Men	Jaundice	Ever smoker	50	Upper GI	0.057	(0.042, 0.077)

Sex	Index symptom	Smoking status	Age (years)	Outcome event	Probability of outcome	(95% CI)
Men	Jaundice	Ever smoker	50	Lower GI	0.002	(<0.001, 0.003)
Men	Jaundice	Ever smoker	50	Urological	0.002	(0.001, 0.005)
Men	Jaundice	Ever smoker	50	Haematological	0.004	(0.001, 0.009)
Men	Jaundice	Ever smoker	50	Other cancers	0.028	(0.020, 0.041)
Men	Jaundice	Ever smoker	50	Death w/o cancer	0.021	(0.014, 0.033)
Men	Jaundice	Ever smoker	60	Lung	0.005	(0.002, 0.012)
Men	Jaundice	Ever smoker	60	Upper GI	0.148	(0.130, 0.169)
Men	Jaundice	Ever smoker	60	Lower GI	0.004	(0.001, 0.011)
Men	Jaundice	Ever smoker	60	Urological	0.005	(0.002, 0.016)
Men	Jaundice	Ever smoker	60	Prostate	0.003	(0.001, 0.008)
Men	Jaundice	Ever smoker	60	Haematological	0.007	(0.002, 0.022)
Men	Jaundice	Ever smoker	60	Other cancers	0.049	(0.031, 0.076)
Men	Jaundice	Ever smoker	60	Death w/o cancer	0.041	(0.028, 0.059)
Men	Jaundice	Ever smoker	70	Lung	0.010	(0.006, 0.018)
Men	Jaundice	Ever smoker	70	Upper GI	0.264	(0.215, 0.319)
Men	Jaundice	Ever smoker	70	Lower GI	0.007	(0.002, 0.023)
Men	Jaundice	Ever smoker	70	Urological	0.008	(0.002, 0.026)
Men	Jaundice	Ever smoker	70	Prostate	0.007	(0.003, 0.017)
Men	Jaundice	Ever smoker	70	Haematological	0.011	(0.005, 0.028)
Men	Jaundice	Ever smoker	70	Other cancers	0.071	(0.047, 0.105)
Men	Jaundice	Ever smoker	70	Death w/o cancer	0.084	(0.068, 0.105)
Men	Jaundice	Ever smoker	80	Lung	0.012	(0.006, 0.022)
Men	Jaundice	Ever smoker	80	Upper GI	0.285	(0.242, 0.331)
Men	Jaundice	Ever smoker	80	Lower GI	0.009	(0.003, 0.026)
Men	Jaundice	Ever smoker	80	Urological	0.007	(0.004, 0.015)
Men	Jaundice	Ever smoker	80	Prostate	0.008	(0.003, 0.019)
Men	Jaundice	Ever smoker	80	Haematological	0.013	(0.008, 0.023)
Men	Jaundice	Ever smoker	80	Other cancers	0.087	(0.067, 0.114)
Men	Jaundice	Ever smoker	80	Death w/o cancer	0.207	(0.175, 0.242)
Men	Jaundice	Ever smoker	90	Lung	0.010	(0.005, 0.018)
Men	Jaundice	Ever smoker	90	Upper GI	0.246	(0.202, 0.296)
Men	Jaundice	Ever smoker	90	Lower GI	0.008	(0.002, 0.027)
Men	Jaundice	Ever smoker	90	Urological	0.006	(0.002, 0.014)
Men	Jaundice	Ever smoker	90	Prostate	0.006	(0.001, 0.021)
Men	Jaundice	Ever smoker	90	Haematological	0.012	(0.007, 0.023)
Men	Jaundice	Ever smoker	90	Other cancers	0.095	(0.071, 0.125)
Men	Jaundice	Ever smoker	90	Death w/o cancer	0.456	(0.386, 0.528)
Men	Night sweats	Never smoker	40	Haematological	0.002	(<0.001, 0.003)
Men	Night sweats	Never smoker	50	Lower GI	0.001	(<0.001, 0.002)
Men	Night sweats	Never smoker	50	Urological	0.001	(<0.001, 0.002)
Men	Night sweats	Never smoker	50	Haematological	0.003	(0.001, 0.005)
Men	Night sweats	Never smoker	50	Other cancers	0.002	(<0.001, 0.004)
Men	Night sweats	Never smoker	50	Death w/o cancer	0.002	(<0.001, 0.004)
Men	Night sweats	Never smoker	60	Lung	0.002	(<0.001, 0.006)
Men	Night sweats	Never smoker	60	Lower GI	0.003	(0.001, 0.007)
Men	Night sweats	Never smoker	60	Urological	0.003	(0.001, 0.009)
Men	Night sweats	Never smoker	60	Prostate	0.007	(0.003, 0.015)
Men	Night sweats	Never smoker	60	Haematological	0.006	(0.002, 0.019)
Men	Night sweats	Never smoker	60	Other cancers	0.003	(0.001, 0.007)
Men	Night sweats	Never smoker	60	Death w/o cancer	0.004	(0.001, 0.011)
Men	Night sweats	Never smoker	70	Lung	0.005	(0.002, 0.013)
Men	Night sweats	Never smoker	70	Upper GI	0.001	(<0.001, 0.004)
Men	Night sweats	Never smoker	70	Lower GI	0.007	(0.002, 0.021)
Men	Night sweats	Never smoker	70	Urological	0.006	(0.003, 0.014)
Men	Night sweats	Never smoker	70	Prostate	0.018	(0.011, 0.029)
Men	Night sweats	Never smoker	70	Haematological	0.013	(0.006, 0.027)
Men	Night sweats	Never smoker	70	Other cancers	0.006	(0.002, 0.016)
Men	Night sweats	Never smoker	70	Death w/o cancer	0.009	(0.005, 0.016)
Men	Night sweats	Never smoker	80	Lung	0.007	(0.002, 0.020)
Men	Night sweats	Never smoker	80	Upper GI	0.001	(<0.001, 0.005)
Men	Night sweats	Never smoker	80	Lower GI	0.009	(0.004, 0.022)
Men	Night sweats	Never smoker	80	Urological	0.007	(0.002, 0.029)

Sex	Index symptom	Smoking status	Age (years)	Outcome event	Probability of outcome	(95% CI)
Men	Night sweats	Never smoker	80	Prostate	0.022	(0.014, 0.036)
Men	Night sweats	Never smoker	80	Haematological	0.016	(0.008, 0.034)
Men	Night sweats	Never smoker	80	Other cancers	0.007	(0.003, 0.015)
Men	Night sweats	Never smoker	80	Death w/o cancer	0.025	(0.015, 0.040)
Men	Night sweats	Never smoker	90	Lung	0.007	(0.003, 0.017)
Men	Night sweats	Never smoker	90	Upper GI	0.002	(<0.001, 0.005)
Men	Night sweats	Never smoker	90	Lower GI	0.011	(0.004, 0.026)
Men	Night sweats	Never smoker	90	Urological	0.008	(0.003, 0.018)
Men	Night sweats	Never smoker	90	Prostate	0.024	(0.015, 0.039)
Men	Night sweats	Never smoker	90	Haematological	0.021	(0.010, 0.044)
Men	Night sweats	Never smoker	90	Other cancers	0.010	(0.005, 0.021)
Men	Night sweats	Never smoker	90	Death w/o cancer	0.072	(0.045, 0.114)
Men	Night sweats	Ever smoker	40	Haematological	0.001	(<0.001, 0.003)
Men	Night sweats	Ever smoker	40	Other cancers	0.001	(<0.001, 0.002)
Men	Night sweats	Ever smoker	40	Death w/o cancer	0.001	(<0.001, 0.002)
Men	Night sweats	Ever smoker	50	Lung	0.002	(0.001, 0.004)
Men	Night sweats	Ever smoker	50	Lower GI	0.001	(<0.001, 0.003)
Men	Night sweats	Ever smoker	50	Urological	0.002	(<0.001, 0.005)
Men	Night sweats	Ever smoker	50	Haematological	0.002	(0.001, 0.006)
Men	Night sweats	Ever smoker	50	Other cancers	0.002	(<0.001, 0.006)
Men	Night sweats	Ever smoker	50	Death w/o cancer	0.002	(<0.001, 0.004)
Men	Night sweats	Ever smoker	60	Lung	0.009	(0.004, 0.023)
Men	Night sweats	Ever smoker	60	Lower GI	0.003	(0.001, 0.008)
Men	Night sweats	Ever smoker	60	Urological	0.005	(0.002, 0.016)
Men	Night sweats	Ever smoker	60	Prostate	0.006	(0.003, 0.013)
Men	Night sweats	Ever smoker	60	Haematological	0.007	(0.002, 0.018)
Men	Night sweats	Ever smoker	60	Other cancers	0.004	(0.002, 0.011)
Men	Night sweats	Ever smoker	60	Death w/o cancer	0.004	(0.002, 0.008)
Men	Night sweats	Ever smoker	70	Lung	0.020	(0.009, 0.042)
Men	Night sweats	Ever smoker	70	Upper GI	0.002	(<0.001, 0.004)
Men	Night sweats	Ever smoker	70	Lower GI	0.007	(0.002, 0.021)
Men	Night sweats	Ever smoker	70	Urological	0.008	(0.004, 0.018)
Men	Night sweats	Ever smoker	70	Prostate	0.015	(0.007, 0.031)
Men	Night sweats	Ever smoker	70	Haematological	0.012	(0.005, 0.025)
Men	Night sweats	Ever smoker	70	Other cancers	0.006	(0.002, 0.020)
Men	Night sweats	Ever smoker	70	Death w/o cancer	0.009	(0.005, 0.016)
Men	Night sweats	Ever smoker	80	Lung	0.026	(0.016, 0.041)
Men	Night sweats	Ever smoker	80	Upper GI	0.002	(<0.001, 0.006)
Men	Night sweats	Ever smoker	80	Lower GI	0.010	(0.004, 0.023)
Men	Night sweats	Ever smoker	80	Urological	0.010	(0.003, 0.031)
Men	Night sweats	Ever smoker	80	Prostate	0.019	(0.011, 0.032)
Men	Night sweats	Ever smoker	80	Haematological	0.017	(0.007, 0.037)
Men	Night sweats	Ever smoker	80	Other cancers	0.009	(0.005, 0.019)
Men	Night sweats	Ever smoker	80	Death w/o cancer	0.026	(0.016, 0.042)
Men	Night sweats	Ever smoker	90	Lung	0.028	(0.017, 0.046)
Men	Night sweats	Ever smoker	90	Upper GI	0.002	(<0.001, 0.005)
Men	Night sweats	Ever smoker	90	Lower GI	0.012	(0.005, 0.028)
Men	Night sweats	Ever smoker	90	Urological	0.011	(0.005, 0.026)
Men	Night sweats	Ever smoker	90	Prostate	0.021	(0.013, 0.032)
Men	Night sweats	Ever smoker	90	Haematological	0.021	(0.009, 0.044)
Men	Night sweats	Ever smoker	90	Other cancers	0.012	(0.006, 0.023)
Men	Night sweats	Ever smoker	90	Death w/o cancer	0.079	(0.048, 0.128)
Men	Rectal bleeding	Never smoker	30	Lower GI	0.002	(0.001, 0.003)
Men	Rectal bleeding	Never smoker	40	Lower GI	0.003	(0.001, 0.006)
Men	Rectal bleeding	Never smoker	40	Death w/o cancer	0.001	(<0.001, 0.003)
Men	Rectal bleeding	Never smoker	50	Lower GI	0.012	(0.007, 0.020)
Men	Rectal bleeding	Never smoker	50	Other cancers	0.002	(<0.001, 0.003)
Men	Rectal bleeding	Never smoker	50	Death w/o cancer	0.003	(0.001, 0.007)
Men	Rectal bleeding	Never smoker	60	Upper GI	0.002	(<0.001, 0.005)
Men	Rectal bleeding	Never smoker	60	Lower GI	0.030	(0.020, 0.044)
Men	Rectal bleeding	Never smoker	60	Urological	0.001	(<0.001, 0.003)
Men	Rectal bleeding	Never smoker	60	Prostate	0.006	(0.003, 0.015)

Sex	Index symptom	Smoking status	Age (years)	Outcome event	Probability of outcome	(95% CI)
Men	Rectal bleeding	Never smoker	60	Haematological	0.002	(<0.001, 0.003)
Men	Rectal bleeding	Never smoker	60	Other cancers	0.003	(0.001, 0.006)
Men	Rectal bleeding	Never smoker	60	Death w/o cancer	0.007	(0.003, 0.015)
Men	Rectal bleeding	Never smoker	70	Lung	0.001	(<0.001, 0.002)
Men	Rectal bleeding	Never smoker	70	Upper GI	0.004	(0.001, 0.009)
Men	Rectal bleeding	Never smoker	70	Lower GI	0.065	(0.041, 0.103)
Men	Rectal bleeding	Never smoker	70	Urological	0.003	(0.001, 0.006)
Men	Rectal bleeding	Never smoker	70	Prostate	0.014	(0.008, 0.025)
Men	Rectal bleeding	Never smoker	70	Haematological	0.003	(0.001, 0.007)
Men	Rectal bleeding	Never smoker	70	Other cancers	0.005	(0.002, 0.012)
Men	Rectal bleeding	Never smoker	70	Death w/o cancer	0.016	(0.010, 0.025)
Men	Rectal bleeding	Never smoker	80	Lung	0.001	(<0.001, 0.003)
Men	Rectal bleeding	Never smoker	80	Upper GI	0.005	(0.002, 0.009)
Men	Rectal bleeding	Never smoker	80	Lower GI	0.085	(0.070, 0.104)
Men	Rectal bleeding	Never smoker	80	Urological	0.003	(<0.001, 0.007)
Men	Rectal bleeding	Never smoker	80	Prostate	0.019	(0.013, 0.028)
Men	Rectal bleeding	Never smoker	80	Haematological	0.004	(0.002, 0.010)
Men	Rectal bleeding	Never smoker	80	Other cancers	0.006	(0.003, 0.015)
Men	Rectal bleeding	Never smoker	80	Death w/o cancer	0.046	(0.033, 0.065)
Men	Rectal bleeding	Never smoker	90	Lung	0.001	(<0.001, 0.003)
Men	Rectal bleeding	Never smoker	90	Upper GI	0.004	(0.002, 0.010)
Men	Rectal bleeding	Never smoker	90	Lower GI	0.096	(0.077, 0.119)
Men	Rectal bleeding	Never smoker	90	Urological	0.003	(0.001, 0.007)
Men	Rectal bleeding	Never smoker	90	Prostate	0.019	(0.012, 0.033)
Men	Rectal bleeding	Never smoker	90	Haematological	0.004	(0.002, 0.010)
Men	Rectal bleeding	Never smoker	90	Other cancers	0.008	(0.004, 0.016)
Men	Rectal bleeding	Never smoker	90	Death w/o cancer	0.129	(0.109, 0.152)
Men	Rectal bleeding	Ever smoker	30	Lower GI	0.002	(0.001, 0.003)
Men	Rectal bleeding	Ever smoker	30	Death w/o cancer	0.001	(<0.001, 0.002)
Men	Rectal bleeding	Ever smoker	40	Lower GI	0.003	(0.001, 0.008)
Men	Rectal bleeding	Ever smoker	40	Death w/o cancer	0.002	(<0.001, 0.004)
Men	Rectal bleeding	Ever smoker	50	Lower GI	0.012	(0.007, 0.020)
Men	Rectal bleeding	Ever smoker	50	Other cancers	0.002	(<0.001, 0.004)
Men	Rectal bleeding	Ever smoker	50	Death w/o cancer	0.003	(0.001, 0.010)
Men	Rectal bleeding	Ever smoker	60	Lung	0.002	(<0.001, 0.004)
Men	Rectal bleeding	Ever smoker	60	Upper GI	0.003	(0.001, 0.005)
Men	Rectal bleeding	Ever smoker	60	Lower GI	0.031	(0.021, 0.046)
Men	Rectal bleeding	Ever smoker	60	Urological	0.002	(0.001, 0.003)
Men	Rectal bleeding	Ever smoker	60	Prostate	0.006	(0.003, 0.011)
Men	Rectal bleeding	Ever smoker	60	Haematological	0.002	(<0.001, 0.004)
Men	Rectal bleeding	Ever smoker	60	Other cancers	0.003	(0.001, 0.009)
Men	Rectal bleeding	Ever smoker	60	Death w/o cancer	0.007	(0.003, 0.016)
Men	Rectal bleeding	Ever smoker	70	Lung	0.004	(0.001, 0.012)
Men	Rectal bleeding	Ever smoker	70	Upper GI	0.005	(0.002, 0.015)
Men	Rectal bleeding	Ever smoker	70	Lower GI	0.068	(0.050, 0.092)
Men	Rectal bleeding	Ever smoker	70	Urological	0.003	(0.001, 0.008)
Men	Rectal bleeding	Ever smoker	70	Prostate	0.013	(0.007, 0.023)
Men	Rectal bleeding	Ever smoker	70	Haematological	0.003	(0.001, 0.007)
Men	Rectal bleeding	Ever smoker	70	Other cancers	0.006	(0.002, 0.016)
Men	Rectal bleeding	Ever smoker	70	Death w/o cancer	0.018	(0.010, 0.032)
Men	Rectal bleeding	Ever smoker	80	Lung	0.005	(0.002, 0.010)
Men	Rectal bleeding	Ever smoker	80	Upper GI	0.006	(0.002, 0.016)
Men	Rectal bleeding	Ever smoker	80	Lower GI	0.091	(0.070, 0.118)
Men	Rectal bleeding	Ever smoker	80	Urological	0.004	(0.001, 0.011)
Men	Rectal bleeding	Ever smoker	80	Prostate	0.016	(0.008, 0.031)
Men	Rectal bleeding	Ever smoker	80	Haematological	0.004	(0.001, 0.010)
Men	Rectal bleeding	Ever smoker	80	Other cancers	0.008	(0.004, 0.013)
Men	Rectal bleeding	Ever smoker	80	Death w/o cancer	0.048	(0.036, 0.064)
Men	Rectal bleeding	Ever smoker	90	Lung	0.006	(0.002, 0.013)
Men	Rectal bleeding	Ever smoker	90	Upper GI	0.006	(0.003, 0.012)
Men	Rectal bleeding	Ever smoker	90	Lower GI	0.100	(0.078, 0.127)
Men	Rectal bleeding	Ever smoker	90	Urological	0.004	(0.002, 0.011)

Sex	Index symptom	Smoking status	Age (years)	Outcome event	Probability of outcome	(95% CI)
Men	Rectal bleeding	Ever smoker	90	Prostate	0.017	(0.011, 0.027)
Men	Rectal bleeding	Ever smoker	90	Haematological	0.004	(0.002, 0.011)
Men	Rectal bleeding	Ever smoker	90	Other cancers	0.010	(0.004, 0.021)
Men	Rectal bleeding	Ever smoker	90	Death w/o cancer	0.140	(0.117, 0.167)
Men	Weight loss	Never smoker	30	Other cancers	0.001	(<0.001, 0.002)
Men	Weight loss	Never smoker	30	Death w/o cancer	0.002	(<0.001, 0.004)
Men	Weight loss	Never smoker	40	Haematological	0.001	(<0.001, 0.004)
Men	Weight loss	Never smoker	40	Other cancers	0.002	(<0.001, 0.003)
Men	Weight loss	Never smoker	40	Death w/o cancer	0.004	(0.002, 0.008)
Men	Weight loss	Never smoker	50	Lung	0.001	(<0.001, 0.002)
Men	Weight loss	Never smoker	50	Upper GI	0.004	(0.002, 0.007)
Men	Weight loss	Never smoker	50	Lower GI	0.003	(0.001, 0.006)
Men	Weight loss	Never smoker	50	Urological	0.002	(<0.001, 0.004)
Men	Weight loss	Never smoker	50	Prostate	0.001	(<0.001, 0.003)
Men	Weight loss	Never smoker	50	Haematological	0.002	(<0.001, 0.006)
Men	Weight loss	Never smoker	50	Other cancers	0.004	(0.001, 0.010)
Men	Weight loss	Never smoker	50	Death w/o cancer	0.007	(0.004, 0.013)
Men	Weight loss	Never smoker	60	Lung	0.004	(0.001, 0.013)
Men	Weight loss	Never smoker	60	Upper GI	0.010	(0.006, 0.018)
Men	Weight loss	Never smoker	60	Lower GI	0.007	(0.003, 0.018)
Men	Weight loss	Never smoker	60	Urological	0.004	(0.001, 0.010)
Men	Weight loss	Never smoker	60	Prostate	0.011	(0.006, 0.018)
Men	Weight loss	Never smoker	60	Haematological	0.006	(0.003, 0.013)
Men	Weight loss	Never smoker	60	Other cancers	0.006	(0.003, 0.012)
Men	Weight loss	Never smoker	60	Death w/o cancer	0.015	(0.009, 0.026)
Men	Weight loss	Never smoker	70	Lung	0.009	(0.004, 0.019)
Men	Weight loss	Never smoker	70	Upper GI	0.020	(0.012, 0.035)
Men	Weight loss	Never smoker	70	Lower GI	0.016	(0.010, 0.027)
Men	Weight loss	Never smoker	70	Urological	0.006	(0.003, 0.013)
Men	Weight loss	Never smoker	70	Prostate	0.025	(0.016, 0.039)
Men	Weight loss	Never smoker	70	Haematological	0.011	(0.005, 0.023)
Men	Weight loss	Never smoker	70	Other cancers	0.011	(0.006, 0.020)
Men	Weight loss	Never smoker	70	Death w/o cancer	0.035	(0.024, 0.050)
Men	Weight loss	Never smoker	80	Lung	0.012	(0.006, 0.021)
Men	Weight loss	Never smoker	80	Upper GI	0.024	(0.016, 0.036)
Men	Weight loss	Never smoker	80	Lower GI	0.021	(0.014, 0.031)
Men	Weight loss	Never smoker	80	Urological	0.008	(0.002, 0.023)
Men	Weight loss	Never smoker	80	Prostate	0.031	(0.021, 0.046)
Men	Weight loss	Never smoker	80	Haematological	0.015	(0.008, 0.026)
Men	Weight loss	Never smoker	80	Other cancers	0.015	(0.008, 0.027)
Men	Weight loss	Never smoker	80	Death w/o cancer	0.099	(0.078, 0.124)
Men	Weight loss	Never smoker	90	Lung	0.012	(0.007, 0.020)
Men	Weight loss	Never smoker	90	Upper GI	0.023	(0.014, 0.037)
Men	Weight loss	Never smoker	90	Lower GI	0.023	(0.013, 0.040)
Men	Weight loss	Never smoker	90	Urological	0.007	(0.004, 0.015)
Men	Weight loss	Never smoker	90	Prostate	0.032	(0.022, 0.046)
Men	Weight loss	Never smoker	90	Haematological	0.017	(0.010, 0.029)
Men	Weight loss	Never smoker	90	Other cancers	0.018	(0.011, 0.030)
Men	Weight loss	Never smoker	90	Death w/o cancer	0.262	(0.229, 0.298)
Men	Weight loss	Ever smoker	30	Other cancers	0.002	(<0.001, 0.003)
Men	Weight loss	Ever smoker	30	Death w/o cancer	0.002	(0.001, 0.004)
Men	Weight loss	Ever smoker	40	Upper GI	0.001	(<0.001, 0.002)
Men	Weight loss	Ever smoker	40	Haematological	0.002	(<0.001, 0.003)
Men	Weight loss	Ever smoker	40	Other cancers	0.002	(0.001, 0.004)
Men	Weight loss	Ever smoker	40	Death w/o cancer	0.004	(0.002, 0.011)
Men	Weight loss	Ever smoker	50	Lung	0.004	(0.003, 0.007)
Men	Weight loss	Ever smoker	50	Upper GI	0.004	(0.002, 0.011)
Men	Weight loss	Ever smoker	50	Lower GI	0.003	(0.001, 0.007)
Men	Weight loss	Ever smoker	50	Urological	0.002	(<0.001, 0.005)
Men	Weight loss	Ever smoker	50	Prostate	0.001	(<0.001, 0.002)
Men	Weight loss	Ever smoker	50	Haematological	0.002	(0.001, 0.006)
Men	Weight loss	Ever smoker	50	Other cancers	0.004	(0.002, 0.009)

Sex	Index symptom	Smoking status	Age (years)	Outcome event	Probability of outcome	(95% CI)
Men	Weight loss	Ever smoker	50	Death w/o cancer	0.008	(0.004, 0.016)
Men	Weight loss	Ever smoker	60	Lung	0.016	(0.010, 0.025)
Men	Weight loss	Ever smoker	60	Upper GI	0.014	(0.008, 0.024)
Men	Weight loss	Ever smoker	60	Lower GI	0.007	(0.003, 0.018)
Men	Weight loss	Ever smoker	60	Urological	0.005	(0.002, 0.011)
Men	Weight loss	Ever smoker	60	Prostate	0.009	(0.004, 0.020)
Men	Weight loss	Ever smoker	60	Haematological	0.006	(0.002, 0.014)
Men	Weight loss	Ever smoker	60	Other cancers	0.008	(0.004, 0.017)
Men	Weight loss	Ever smoker	60	Death w/o cancer	0.016	(0.009, 0.027)
Men	Weight loss	Ever smoker	70	Lung	0.034	(0.026, 0.045)
Men	Weight loss	Ever smoker	70	Upper GI	0.027	(0.017, 0.044)
Men	Weight loss	Ever smoker	70	Lower GI	0.015	(0.007, 0.031)
Men	Weight loss	Ever smoker	70	Urological	0.009	(0.004, 0.019)
Men	Weight loss	Ever smoker	70	Prostate	0.021	(0.013, 0.033)
Men	Weight loss	Ever smoker	70	Haematological	0.011	(0.008, 0.017)
Men	Weight loss	Ever smoker	70	Other cancers	0.012	(0.007, 0.021)
Men	Weight loss	Ever smoker	70	Death w/o cancer	0.038	(0.026, 0.054)
Men	Weight loss	Ever smoker	80	Lung	0.044	(0.032, 0.059)
Men	Weight loss	Ever smoker	80	Upper GI	0.031	(0.020, 0.047)
Men	Weight loss	Ever smoker	80	Lower GI	0.021	(0.013, 0.035)
Men	Weight loss	Ever smoker	80	Urological	0.010	(0.005, 0.019)
Men	Weight loss	Ever smoker	80	Prostate	0.027	(0.016, 0.045)
Men	Weight loss	Ever smoker	80	Haematological	0.015	(0.009, 0.025)
Men	Weight loss	Ever smoker	80	Other cancers	0.016	(0.010, 0.028)
Men	Weight loss	Ever smoker	80	Death w/o cancer	0.102	(0.084, 0.122)
Men	Weight loss	Ever smoker	90	Lung	0.047	(0.031, 0.069)
Men	Weight loss	Ever smoker	90	Upper GI	0.031	(0.022, 0.044)
Men	Weight loss	Ever smoker	90	Lower GI	0.023	(0.015, 0.036)
Men	Weight loss	Ever smoker	90	Urological	0.010	(0.005, 0.019)
Men	Weight loss	Ever smoker	90	Prostate	0.026	(0.019, 0.036)
Men	Weight loss	Ever smoker	90	Haematological	0.017	(0.009, 0.030)
Men	Weight loss	Ever smoker	90	Other cancers	0.021	(0.014, 0.031)
Men	Weight loss	Ever smoker	90	Death w/o cancer	0.271	(0.238, 0.306)

Appendix 6. Additional detail on the risk of specific cancer sites.

Risk of breast cancer

Risk of breast cancer in women presenting with any of the studied symptoms other than breast lump was always under the 3% urgent referral threshold (Figures 4 and 5), while for those presenting with breast lump it reached this threshold from age 35 (smokers) / 40 (non-smokers). In women with breast lump risk appeared to increase rapidly with age, and exceeded 10% from age 50 and 20% from age 60. Breast cancer in men was grouped into the 'other cancers' category (see below).

Risk of gynaecological cancers

Risk of gynaecological cancer exceeded the 3% urgent referral threshold in women presenting with post-menopausal bleeding (Figures 4 and 5); post-menopausal bleeding at any age appeared associated with greater than 3% risk (although estimates for younger ages were based on very small sample sizes). While gynaecological cancer did not reach the 3% threshold for other 14 studied symptoms, there was notable risk of gynaecological cancer in women presenting with haematuria and abdominal bloating. For example, 40-year-old female non-smokers presenting with haematuria had a risk of gynaecological cancer of 0.6% (95% CI 0.2% to 1.5%, Figure 4, Appendix 5 Table 1), and with abdominal bloating a risk of 0.7% (95% CI 0.3% to 1.8%).

Risk of lung cancer

Lung cancer risk in non-smokers was generally minimal across the studied symptoms, except for haemoptysis. Male non-smokers presenting with haemoptysis had 3% risk of lung cancer from age 70 (Table 2, Figure 2). In smokers, there was notable lung cancer risk in patients presenting with haemoptysis (3% or higher risk from age 55 for male smokers, 60 for female smokers, Figures 3 and 5) and for male smokers presenting with weight loss (reaching 3% risk from age 70). For dyspnoea and all three non-specific symptoms studied (fatigue, night sweats, weight loss), there was elevated risk of lung cancer for male and female smokers presenting, though without reaching 3%.

Risk of upper GI cancers

Risk of upper GI cancers exceeded the 3% referral threshold for both men and women presenting with dysphagia (from age 60 in men, 70 in women) and jaundice (from age 50). Patients presenting with dyspepsia, abdominal pain, abdominal bloating and weight loss also had notable risk of upper GI cancer, but not reaching the 3% threshold except for male smokers presenting with weight loss, where the risk of upper GI cancer reached 3% from age 75. The specific types of upper GI cancer varied by index symptom. Pancreatic cancer was the most commonly diagnosed cancer following a presentation with jaundice (237 pancreatic cancers of 456 total cancers in patients with jaundice as index symptom, Appendix 2 Table 1), while dysphagia was more frequently associated with oesophageal cancer (558 oesophageal cancers of 1,036 total cancers).

Risk of lower GI cancers

Risk of lower GI cancers exceeded the 3% referral threshold for patients presenting with rectal bleeding or change in bowel habit from around age 60 in men and around age 70 in women. Patients presenting with abdominal pain and abdominal bloating, and also non-specific symptoms such as fatigue, night sweats, and weight loss, appeared to have a 1-2% risk of lower GI cancer from around age 60.

Risk of urological cancers

Risk of urological cancer was higher in smokers than non-smokers, and reached the 3% urgent referral threshold for patients presenting with haematuria from age 55 in men and age 60/65 in female smokers/non-smokers.

Risk of prostate cancer

Risk of prostate cancer reached 3% from around age 70 in men presenting with haematuria, and from age 80 in male non-smokers presenting with weight loss. Older men presenting with many of the studied symptoms had 1-2% risk of prostate cancer (including following presentation with abdominal pain and bloating, rectal bleeding, change in bowel habit, dyspepsia, fatigue, night sweats and weight loss).

Risk of haematological cancers

While many symptoms were associated with relatively large hazard ratios for haematological cancer, the risk of haematological cancers did not reach the 3% referral threshold for any of the studied symptoms. Night sweats in older male patients were associated with the highest risk of haematological cancers.

Risk of other cancers

Risk of other cancers, including breast cancer in men and all other cancers excluding non-melanoma skin cancer, reached the 3% urgent referral threshold from around age 55 for patients presenting with jaundice, and from around age 75 for men presenting with breast lump.

Appendix 7. Additional detail on the limitations of this study

This section expands on the brief summary of limitations given in the main text of the article.

First, the consideration of deaths in patients with cancer. In this analysis, only the first of the two outcomes (either diagnosis of cancer or death) was considered, and so deaths in patients with cancer were not examined in detail, beyond briefly in Table 1. Research to inform diagnostic strategies may additionally consider whether certain symptomatic presentations were associated with worse outcomes in patients diagnosed with cancer, but the focus of this study was cancer incidence alone.

Second, the assessment of smoking-status used in this study was crude, classifying patients as ‘ever-smokers’ if they ever had a positive smoking-related Read code, and ‘never-smokers’ otherwise. This will have led some patients with minimal or no history of smoking being classed as smokers, and did not allow any examination of possible dose-response relationships with measures such as pack-years of smoking [1,2]. A more refined stratification of risk related to exposure to smoking (e.g., including smoking intensity) may be attempted in future as this could further support referral decisions.

Third, each patient was included at most once in the analysis. Landmark methods with multiple observations per patient would have made better use of the longitudinal data from the electronic health record [3], and would have avoided the need to remove patients from the random sample from consideration for their symptom status. Using landmark approaches would have allowed consideration of symptom history (i.e., patients who have previously presented with a symptom and have returned again with that symptom may be different risk of cancer than those presenting for the first time). We considered adopting landmark methods in this analysis but decided against it due to challenges in accurately estimating variances, and the relatively limited precision benefits with an analysis cohort that already included 1.7 million patients.

Fourth, examination of risk of cancer in patients with multiple symptoms was limited. Analysis included all symptoms patients had at index when estimating cause-specific hazard ratios, but we did not consider possible interactions between index symptoms and we simulated cumulative incidence curves for single index symptoms only. Further simulations could be carried out to produce cumulative incidence curves for combinations of index symptoms, although given only a small fraction of patients have multiple index symptoms the value of such analyses may be limited in practice.

Fifth, analysis relies entirely on symptoms that were recorded in coded form in the electronic health record. We know that coded data does not capture all symptoms, or even all symptoms that are entered in free-text in the patient’s record [4,5], despite the proportion of cancer patients in CPRD who are symptomatic being similar to the proportion of patients in the National Cancer Diagnosis Audit for whom a symptom was judged to be relevant to their diagnosis [6,7]. This motivated the use of a random sample as a reference group rather than an apparently-symptom-free comparison group; we could be confident that patients with a recorded symptom

had that symptom, but it was harder to be certain that a patient without a recorded symptom did not have a symptom. It would be preferable to have access to free-text data to get better ascertainment of patients' symptom status, bearing in mind that weather the symptom was recorded using structured code or entered in free-text may in itself be informative of risk level. Yet data protection concerns mean that access to free-text data for research is limited at present.

Sixth, while we examine 15 different symptoms possibly related to an underlying cancer diagnosis, there are over 70 other symptoms that could have additionally been examined [8]. Further, certain comorbidities may be linked to both the presence of a symptom and underlying risk of cancer, for example COPD is linked to both dyspnoea and lung cancer risk [9]. The symptomatic presentation and diagnostic management of patients with cancer and prior comorbidities may vary to that observed in patients without pre-existing chronic conditions [10]. There are practical limits on how much can be examined in a multi-exposure multi-outcome descriptive study, and examining the complicated associations between comorbidities, symptoms, and individual cancer sites is likely best served by carefully designed single-outcome studies.

Citations in above text (different numbering from article main-text)

1 Hippisley-Cox J, Coupland C. Symptoms and risk factors to identify women with suspected cancer in primary care: derivation and validation of an algorithm. *The British Journal of General Practice*. 2013;63:e11.

2 Hippisley-Cox J, Coupland C. Symptoms and risk factors to identify men with suspected cancer in primary care: derivation and validation of an algorithm. *British Journal of General Practice*. 2013;63:e1–10.

3 Keogh RH, Seaman SR, Barrett JK, *et al.* Dynamic Prediction of Survival in Cystic Fibrosis: A Landmarking Analysis Using UK Patient Registry Data. *Epidemiology*. 2019;30:29–37.

4 Kostopoulou O, Tracey C, Delaney BC. Can decision support combat incompleteness and bias in routine primary care data? *Journal of the American Medical Informatics Association*. 2021;28:1461–7.

5 Price SJ, Stapley SA, Shephard E, *et al.* Is omission of free text records a possible source of data loss and bias in Clinical Practice Research Datalink studies? A case-control study. *BMJ open*. 2016;6. doi: 10.1136/BMJOPEN-2016-011664

6 Barclay M, Renzi C, Antoniou A, *et al.* Phenotypes and rates of cancer-relevant symptoms and tests in the year before cancer diagnosis in UK Biobank and CPRD Gold. *PLOS Digital Health*. 2023;2:e0000383.

7 Zakkak N, Barclay ME, Swann R, *et al.* The presenting symptom signatures of incident cancer: evidence from the English 2018 National Cancer Diagnosis Audit. *Br J Cancer*. 2023;1–11.

8 Moore SF, Price SJ, Chowienczyk S, *et al.* The impact of changing risk thresholds on the number of people in England eligible for urgent investigation for possible cancer: an observational cross-sectional study. *British Journal of Cancer*. 2021;125:1593–7.

9 Skillrud D, Offord K, Miller R. Higher Risk of Lung Cancer in Chronic Obstructive Pulmonary Disease. *Annals of Internal Medicine*. 1986;105:503.

10 Majano SB, Lyratzopoulos G, Rachet B, *et al.* Do presenting symptoms, use of pre-diagnostic endoscopy and risk of emergency cancer diagnosis vary by comorbidity burden and type in patients with colorectal cancer? *British Journal of Cancer 2021 126:4*. 2021;126:652–63.
